# Genome-wide analysis of flavonoid biosynthetic genes in Musaceae (*Ensete*, *Musella*, and *Musa* species) reveals amplification of flavonoid 3ʹ,5ʹ-hydroxylase

**DOI:** 10.1093/aobpla/plae049

**Published:** 2024-09-10

**Authors:** Dongli Cui, Gui Xiong, Lyuhan Ye, Richard Gornall, Ziwei Wang, Pat Heslop-Harrison, Qing Liu

**Affiliations:** Key Laboratory of National Forestry and Grassland Administration Plant Conservation and Utilization in Southern China/Guangdong Provincial Key Laboratory of Applied Botany, South China Botanical Garden, Chinese Academy of Sciences, Xingke Road 723, Tianhe District, Guangzhou 510650, China; South China National Botanical Garden, Xingke Road 723, Tianhe District, Guangzhou 510650, China; University of Chinese Academy of Sciences, Yuquan Road 19, Shijingshan District, Beijing 100049, China; Key Laboratory of National Forestry and Grassland Administration Plant Conservation and Utilization in Southern China/Guangdong Provincial Key Laboratory of Applied Botany, South China Botanical Garden, Chinese Academy of Sciences, Xingke Road 723, Tianhe District, Guangzhou 510650, China; South China National Botanical Garden, Xingke Road 723, Tianhe District, Guangzhou 510650, China; University of Chinese Academy of Sciences, Yuquan Road 19, Shijingshan District, Beijing 100049, China; Key Laboratory of National Forestry and Grassland Administration Plant Conservation and Utilization in Southern China/Guangdong Provincial Key Laboratory of Applied Botany, South China Botanical Garden, Chinese Academy of Sciences, Xingke Road 723, Tianhe District, Guangzhou 510650, China; South China National Botanical Garden, Xingke Road 723, Tianhe District, Guangzhou 510650, China; University of Chinese Academy of Sciences, Yuquan Road 19, Shijingshan District, Beijing 100049, China; University of Leicester, Department of Genetics and Genome Biology, Institute for Environmental Futures, University Road, Leicester LE1 7RH, UK; Henry Fok School of Biology and Agriculture, Shaoguan University, University Road 288, Zhenjiang District, Shaoguan 512005, China; Key Laboratory of National Forestry and Grassland Administration Plant Conservation and Utilization in Southern China/Guangdong Provincial Key Laboratory of Applied Botany, South China Botanical Garden, Chinese Academy of Sciences, Xingke Road 723, Tianhe District, Guangzhou 510650, China; University of Leicester, Department of Genetics and Genome Biology, Institute for Environmental Futures, University Road, Leicester LE1 7RH, UK; Key Laboratory of National Forestry and Grassland Administration Plant Conservation and Utilization in Southern China/Guangdong Provincial Key Laboratory of Applied Botany, South China Botanical Garden, Chinese Academy of Sciences, Xingke Road 723, Tianhe District, Guangzhou 510650, China; South China National Botanical Garden, Xingke Road 723, Tianhe District, Guangzhou 510650, China; State Key Laboratory of Plant Diversity and Specialty Crops, South China Botanical Garden, Chinese Academy of Sciences, Xingke Road 723, Tianhe District, Guangzhou 510650, China

**Keywords:** Anthocyanins, banana, diversification, F3ʹ5 ʹH, F3ʹH, flavonoids, genetics, genomics, ginger, monocotyledons, Musaceae

## Abstract

Flavonoids in Musaceae are involved in pigmentation and stress responses, including cold resistance, and are a component of the healthy human diet. Identification and analysis of the sequence and copy number of flavonoid biosynthetic genes are valuable for understanding the nature and diversity of flavonoid evolution in Musaceae species. In this study, we identified 71–80 flavonoid biosynthetic genes in chromosome-scale genome sequence assemblies of Musaceae, including those of *Ensete glaucum*, *Musella lasiocarpa*, *Musa beccarii*, *M. acuminata*, *M. balbisiana* and *M. schizocarpa,* checking annotations with BLAST and determining the presence of conserved domains. The number of genes increased through segmental duplication and tandem duplication. Orthologues of both structural and regulatory genes in the flavonoid biosynthetic pathway are highly conserved across Musaceae. The flavonoid 3ʹ,5ʹ-hydroxylase gene *F3ʹ5ʹH* was amplified in Musaceae and ginger compared with grasses (rice, Brachypodium, *Avena longiglumis,* and sorghum). One group of genes from this gene family amplified near the centromere of chromosome 2 in the *x* = 11 Musaceae species. Flavonoid biosynthetic genes displayed few consistent responses in the yellow and red bracts of *Musella lasiocarpa* when subjected to low temperatures. The expression levels of *MlDFR2/3* (dihydroflavonol reductase) increased while *MlLAR* (leucoanthocyanidin reductase) was reduced by half. Overall, the results establish the range of diversity in both sequence and copy number of flavonoid biosynthetic genes during evolution of Musaceae. The combination of allelic variants of genes, changes in their copy numbers, and variation in transcription factors with the modulation of expression under cold treatments and between genotypes with contrasting bract-colours suggests the variation may be exploited in plant breeding programmes, particularly for improvement of stress-resistance in the banana crop.

## Teaser

In this study, we identified 71–80 flavonoid biosynthetic genes in chromosome-scale genome assemblies of five Musaceae species, by checking annotations with BLAST and determining the presence of conserved domains. The number of genes increased through segmental duplication and tandem duplication. The flavonoid 3ʹ,5ʹ-hydroxylase gene F3ʹ5ʹH was amplified in Musaceae and ginger compared with four grass species (rice, Brachypodium, Avena longiglumis, and sorghum). Flavonoid biosynthetic genes displayed few consistent responses in the yellow and red bracts of Musella lasiocarpa when subjected to low temperatures (15 °C). The combination of allelic variants of genes, changes in their copy numbers, and variation in transcription factors with the modulation of expression under cold treatments and between genotypes with contrasting bract-colours suggests the variation may be exploited in plant breeding programs, particularly for improvement of stress-resistance in the banana crop.

## Introduction

The Musaceae comprises three genera, *Ensete* (7 spp.), *Musella* (a monotypic genus) and *Musa* (c. 83 spp.) (Cheesman 1947; Häkkinen 2013), distributed across Southeastern Asia and Africa (Janssens *et al.* 2016). *Musa* and *Ensete* are important crops (Christelová *et al.* 2011; Amah *et al.* 2020) that provide a source of carbohydrates and livelihoods to tropical communities. Cold resistance occurs especially in the Asian species *Musella lasiocarpa* and *Ensete glaucum*, which grow at 1500–2500 m and 800–1100 m above sea level, respectively (Li 1978). The worldwide production of bananas and plantains reached 179 263 140 tons in 2022 (https://www.fao.org/faostat/, accessed 30 January 2024). The basic chromosome number in *Ensete* and *Musella* is considered to be *x* = 9, with all the species being diploid (Cheesman 1947; Li 1978, 1979; Zhang *et al.* 2018; Wang *et al.* 2022, 2023). *Musa* has *x* = 9, 10 and 11 species, including diploids, with triploids and tetraploids present in some species and as hybrids (Wang *et al.* 2019). The wild species are diploids with a signature of ancient genome triplication, while most cultivated lineages of banana and plantains are sterile parthenocarpic triploids (Heslop-Harrison *et al.* 2007; Passos *et al.* 2012). Genetic diversity has been exploited in all three genera through selection for cultivation as food (banana and starch ensete) (Neumann and Hildebrand 2009; Borrell *et al.* 2019), potential medicines (Ranjha *et al.* 2022), and horticulture (Nugraha *et al.* 2023).

Flavonoids occur in all photosynthetic lineages, from cyanobacteria, haptophytes, ochrophytes, rhodophytes and chlorophytes to bryophytes and vascular plants (Goiris *et al.* 2014). These compounds are synthesized in the cytosol and transported into the vacuole, where they can function as bioactive molecules (Rausher 2006; Zhao 2015; Davies *et al.* 2020). Flavonoids are important to plants for the production of colours, especially flowers and fruits, in the context of pollination and seed dispersal (Giusti *et al.* 2023); pollen, spore and seed germination; and seedling development (Wang *et al.* 2020). Flavonoids protect plants from different abiotic and biotic stresses, including by protection against UV damage in plant seedlings and playing roles in frost hardiness and drought resistance (Samanta *et al.* 2011; Ferreyra *et al.* 2021). They are important in defence against pathogens, pests and herbivores (Mahajan *et al.* 2011; van der Kooi *et al.* 2019; Tiku *et al.* 2020). Attention has been given to the potential nutraceutical, pharmaceutical, medicinal and cosmetic applications and benefits of flavonoids due to their antioxidative, antiviral, antimicrobial, inflammatory, mutagenic and anticarcinogenic properties (Someya *et al.* 2002; Panche *et al.* 2016).

**Figure 1.**
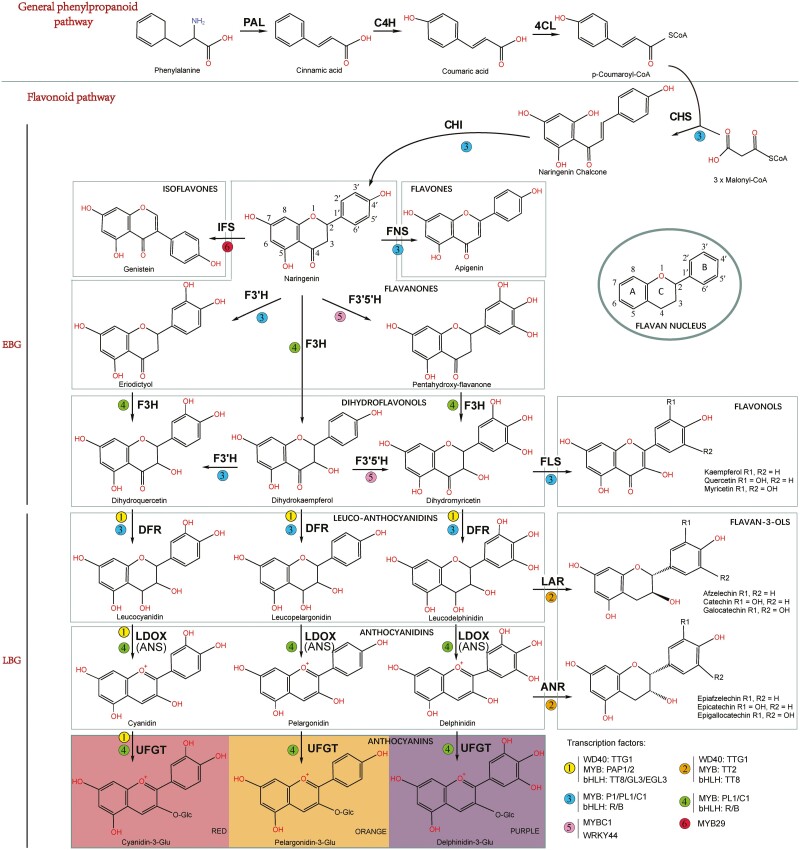
The part of the flavonoid biosynthetic pathway analysed here, with regulators of the genes, in plant cells (based on maize, Arabidopsis, *Brassica*, *Musa*, kiwifruit and soybean). Structural genes encoding enzymes are: PAL, phenylalanine ammonia lyase; C4H, cinnamic acid 4-hydroxylase; 4CL, 4-coumarate CoA ligase; CHS, chalcone synthase; CHI, chalcone isomerase; FNS, flavone synthase; IFS, isoflavone synthase; F3H, flavanone 3-hydroxylase; F3’H, flavanone 3’-hydroxylase; F3’5’H, flavanone 3’5’-hydroxylase; DFR, dihydroflavonol reductase; FLS, flavonol synthase; ANS/LDOX, anthocyanidin synthase/leucoanthocyanidin dioxygenase; UGP/UFGT, UDP-flavonoid glucosyl transferase; ANR, anthocyanidin reductase; LAR, leucoanthocyanidin reductase. EBGs: early biosynthetic genes; LBG: late biosynthetic genes. Regulatory transcription factors MYB (P1/PL1/C1 or PL1/C1) and bHLH (R/B) are from maize; WD40 (TTG1), MYB (TT2/PAP1/PAP2), and bHLH (TT8/GL3/EGL3) are from Arabidopsis; MYBC1 and WRKY44 are from kiwifruit; and MYB29 is from soybean.

Flavonoids are the largest class of polyphenols in the plant kingdom, with some 9000 different molecules (Bate-Smith 1954; Williams *et al.* 2004) bearing a common diphenylpropane (C6–C3–C6) backbone in which two aromatic rings are linked via a three-carbon chain (Saito *et al.* 2013) (Fig. 1; inset flavan nucleus). Flavonoids consist of a benzene ring (A) linked to a pyrone ring (C), which at the 2 or 3 position takes a phenyl ring (B) as a substitute (Fig. 1); based on the oxygenation pattern of the heterocyclic C ring, they can be sub-classified into six groups, namely flavones, flavonols, flavanones, flavanols, anthocyanidins and isoflavones (Alseekh *et al.* 2020; Wen *et al.* 2020). Flavonoid biosynthesis has been studied extensively and the pathways to the various compounds are well-established (Shirley 1996; Ng and Smith 2016; Chu *et al.* 2017; Zhao *et al.* 2020; Manzoor *et al.* 2023). The genes controlling the individual processes have been characterized in detail (Fig. 1; Falcone Ferreyra *et al.* 2012; Peng *et al.* 2020). The biosynthetic pathway starts with phenylalanine via the general phenylpropanoid pathway (Wei *et al.* 2023). Three major gene families, PAL, C4H and 4CL, are involved in the production of p-coumaroyl-CoA via cinnamic acid (precursor to salicylic acid). The resulting structural genes can be divided into two groups (Guo *et al.* 2014). Early biosynthetic genes (EBGs), which include a group of hydroxylases and synthases and late biosynthetic genes (LBGs), which include reductases (Lepiniec *et al.* 2006; Petroni and Tonelli 2011; Pucker *et al.* 2020). In the LBG group, glycosyl transferase genes (UFGT) add the finishing modifications to the molecules. The structural genes have been identified in many species, including Musaceae (Pucker *et al.* 2020; Rijzaani *et al.* 2022; Busche *et al.* 2023).

Three transcription factor (TF) families, R2R3-MYB, bHLH and WD40, comprise the ternary MBW complexes which regulate different branches of the pathway by activating a subset of biosynthetic genes (Wilkins *et al*. 2009; Zheng *et al.* 2019; Pucker 2022). The expression of several genes has been studied; for example, the expression of the *Musa F3ʹ5ʹH-1* gene is strongly affected by abiotic stress conditions and following application of phytohormones to banana plants (Negi *et al.* 2023). Transcriptome expression profiling, e.g. in *Musa* (Liu *et al.* 2023; Mingmanit *et al.* 2023) is increasingly used to complement studies of gene alleles and copy number variations.

Both structural and regulatory genes in the flavonoid pathway have served as model systems for understanding a variety of evolutionary processes, such as gene duplication, evolutionary rate variation among genes, and the relative importance of structural and regulatory genes in the evolution of ecologically important characters (Rausher 2006), including studies of *Musa acuminata* (Pandey *et al.* 2016), *Oryza sativa* (rice) (Xia *et al.* 2021), *Arabidopsis thaliana* (Arabidopsis) and *Brassica rapa* (Guo *et al.* 2014). Analysis of their evolution in both the sequence and copy number of these genes is valuable for phylogenetic reconstruction.

In the present study, we aimed to perform a comparative analysis of flavonoid biosynthetic genes in Musaceae based on recent publications of chromosome-scale genome assemblies and transcriptome data (Droc *et al.* 2022). Interspecific variations in gene copy number, structural organization, genomic location, and the nature and expression of key structural and regulatory genes were investigated, especially in the context of response to low temperatures.

## Materials and Methods

### Identification of putative flavonoid biosynthetic genes

The genomic DNA and inferred protein sequences of *Ensete glaucum*, *Musella lasiocarpa*, *Musa beccarii*, *M. acuminata*, *M. balbisiana* and *M. schizocarpa* were downloaded from the Banana Genome Hub (https://banana-genome-hub.southgreen.fr/content/download) (Droc *et al.* 2022); *Oryza sativa* (PRJDB2223), *Sorghum bicolor* (PRJNA13876), *Zingiber officinale* (PRJNA647255) and *Brachypodium distachyon* (PRJNA32607) protein sequences were downloaded from NCBI (https://www.ncbi.nlm.nih.gov); and *Avena longiglumis* (PRJNA956334) protein sequences were downloaded from Liu *et al.* (2024) to build 11 local protein databases.

Hidden Markov Model (HMM) profiles of the characterized and conserved domains of flavonoid biosynthetic proteins (see Supporting Information—Table S1) were retrieved from the protein family database (https://www.ebi.ac.uk/interpro/) (Mistry *et al.* 2021; Paysan-Lafosse *et al.* 2023) to search for these gene families in the Musaceae. The flavonoid biosynthetic protein sequences were identified based on the HMM profiles using HMMER software with default parameters optimized by the original authors (Finn *et al.* 2011) and a cut-off value of 0.01 (Xie *et al.* 2018). To ensure the accuracy of the identification of flavonoid biosynthetic proteins, we further blasted the flavonoid protein sequences of Arabidopsis, rice and Brachypodium (Qian *et al.* 2011) against the *E. glaucum*, *Musella lasiocarpa*, *Musa beccarii*, *M. acuminata*, *M. balbisiana* and *M. schizocarpa* genome databases. The conserved domains of all the candidate proteins were characterized using the Pfam and Simple Modular Architecture Research Tool (SMART, http://smart.embl-heidelberg.de/) programs. The physico-chemical properties (sequence length, molecular weight and isoelectric point) of the flavonoid biosynthetic proteins were predicted using protparam tool from ExPasy (https://web.expasy.org/protparam/).

Artefacts arise from automated genome-wide annotation, with Bartas *et al.* (2018) noting an apparent triplication of the CHS domain in *M. balbisiana*. Here, we found one (*M. schizocarpa F3ʹH*; *M. balbisiana F3ʹ5ʹH*, and *DFR*) or two (*M. schizocarpa F3ʹ5ʹH*) annotated genes which could not be validated and Table 1 shows the revised gene numbers.

**Table 1:** Number of flavonoid genes in four species of *Musa, Musella* and one each of genera *Ensete*, *Zingiber*, *Avena*, *Brachypodium*, *Oryza*, *Sorghum*, and *Arabidopsis.*

Gene family	*Musa acuminata* sect. *Musa* (Musaceae)	*Musa balbisiana* sect. *Musa* (Musaceae)	*Musa schizocarpa* sect. *Musa* (Musaceae)	*Musa beccarii* sect. *Callimusa* (Musaceae)	*Musella lasiocarpa* (Musaceae)	*Ensete glaucum* (Musaceae)	*Zingiber officinale* (Zingiber-aceae)	*Avena longiglumis* (Poaceae)	*Brachypodium distachyon* (Poaceae)	*Oryza sativa* (Poaceae)	*Sorghum bicolor* (Poaceae)	*Arabidopsis thaliana* (Brassicaceae)
	2*n* = 2*x* = 22	2*n* = 2*x* = 22	2n = 2x = 22	2*n* = 2*x* = 18	2*n* = 2*x* = 18	2*n* = 2*x* = 18	2*n* = 2*x* = 22	2*n* = 2*x* = 14	2*n* = 2*x* = 10	2*n* = 2*x* = 24	2*n* = 2*x* = 20	2*n* = 2*x* = 10
*Structural genes*												
PAL^a^	8	7	8	8	9	9	6	26	8	12	8	4
C4H^a^	4	4	3	4	4	4	3	5	2	1	2	1
4CL^a^	18	16	18	18	18	16	24	24	14	16	17	4
CHS^b^	6	8	5	5	4	6	7	1	1	2	10	1
CHI^b^	2	2	2	2	2	2	2	2	2	3	2	1
F3H^b^	2	2	(1)2	2	2	2	1	1	1	1	2	1
F3’H^b^	1	1	1	1	1	1	2	2	4	2	5	1
F3’5’H^b^	13	(13)14	(8)10	10	12	10	8	1	2	1	2	7
FLS^b^	2	5	3	4	5	4	3	3	1	1	2	6
DFR^c^	3	(2)3	3	3	3	3	1	1	2	1	4	1
LDOX^c^	1	1	1	1	1	1	1	2	0	2	1	1
LAR^c^	1	1	1	1	1	1	0	1	1	1	0	0
ANR^c^	5	1	3	1	4	2	4	19	8	5	2	1
UGT78D2^c^	3	3	3	3	3	3	5	1	2	2	2	1
UGT75C1^c^	4	3	4	3	3	3	7	9	9	6	9	1
UGT79B1^c^	1	(0)1	1	1	1	1	1	1	2	1	3	1
*Regulatory genes (transcription factors)*												
bHLH: TT8 (R1)	2	2	2	2	2	2	1	1	3	1	1	1
WD40: TTG1 (PAC1)	2	2	2	2	2	2	3	1	1	1	1	1
MYB: C1	2	3	2	3	3	2	5	2	1	1	1	0
Total	80	76	71	74	80	74	84	103	64	60	74	34

^a^Flavonoid precursor;

^b^early-pathway;

^c^late-pathway.

The red number in parentheses is the original published annotated gene copy number, with the corrected annotation number next to it (c.f. Bartas *et al.* 2018).

### *In-silico* analysis of promoter *cis*-acting elements

A 1500-bp (base pair) upstream region from the initial codon of each candidate flavonoid biosynthetic gene was used to search *cis*-elements with PlantCARE (http://bioinformatics.psb.ugent.be/webtools/plantcare/html/; accessed November 2023) with default settings (Lescot *et al.* 2002).

### Chromosomal localization and phylogenetic analysis

The physical locations of flavonoid biosynthetic genes were determined in *E. glaucum, Musella lasiocarpa, Musa beccarii, M. acuminata, M. balbisiana* and *M. schizocarpa* genome databases and displayed in MapChart (Voorrips *et al.* 2022). Multiple sequence alignments with the respective protein families in Arabidopsis, *B. distachyon*, *A. longiglumis*, *O. sativa*, *S. bicolor*, *Z. officinale*, *E. glaucum*, *M. lasiocarpa*, *M. beccarii*, *M. acuminata*, *M. balbisiana* and *M. schizocarpa* were performed using ClustalW in MEGA 7.0 (Kumar *et al.* 2018). Phylogenetic trees were constructed using a maximum likelihood (ML) method with 1000 bootstrap replications.

### Analysis of collinearity and duplication

Gene duplication events and collinearity (conserved synteny) were analysed using the Multiple Collinearity Scan toolkit (MCScanX). Amino acid sequences were analysed using BLASTP with an e-value of 1.0 × 10^−5^. The collinear blocks were detected by submitting a whole-genome gff file (downloaded from Banana Genome Hub) and BLASTP results to MCScanX v1.5.1 with default settings. The positions of gene pairs of flavonoid biosynthetic genes were mapped using Circos v0.69 tool (Krzywinski *et al.* 2009).

### Evolutionary analysis of FBGs

Coding sequences (CDSs) of orthologous flavonoid biosynthetic gene pairs between *E. glaucum*, *Musella lasiocarpa*, *Musa beccarii*, *M. acuminata*, *M. balbisiana* and *M. schizocarpa* were aligned using ClustalW. The synonymous (Ks) and non-synonymous (Ka) substitution rates were calculated using the Ka/Ks_calculator 2.0 (Wang *et al.* 2010). The divergence time (*T*) was estimated using the equation *T* = Ks/2*r*, where ‘*r*’ represents the synonymous substitution rate (4.5 per 10^9^ years) (Lescot *et al.* 2008).

### Plant materials and temperature stress treatments

Four-year-old *Musella lasiocarpa* var. *lasiocarpa* (yellow bracts) and *M. lasiocarpa* var. *rubribracteata* (red bracts) were used in this study, which originated from Puge city (E 102.68°, N 23.45°), Liangshan Yi Autonomous Prefecture, Sichuan Province, China. The plants were subsequently grown in a growth cabinet at the South China Botanical Garden for one week at 25 °C with 16 h/8 h (day/night) and 70% relative humidity, and then placed under two temperature regimes: 15 °C in the low-temperature-treated group and 25 °C in the control group (Ravi and Vaganan 2016). After 48 h of treatment, the bracts were collected. Three replicates were conducted for each species and two treatments. The specimens were frozen in liquid nitrogen for RNA extraction and kept at 80 °C.

### RNA-seq-based gene expression analysis

RNA was extracted from the bracts of *Musella lasiocarpa* using an ENZA Plant RNA Kit (R6827, Omega, Guangzhou). The library was constructed using the MGIEasy RNA Library Prep Kit v.3.0 (BGI, Shenzhen). Transcriptome sequencing was performed by cPAS (Combinatorial probe-anchor synthesis) using the high-throughput sequencing platform DNBSEQ-T7 (MGI) and the RNA-seq data can be obtained from the Genome Sequence Archive of the China National Center for Bioinformation project PRJCA023025. Low-quality sequences and splice sequences in raw reads were filtered using the software fastp.v.0.21.0 (Chen *et al.* 2018) to obtain clean reads. The filtered reads were aligned to the *Musella lasiocarpa* genome (https://banana-genome-hub.southgreen.fr/node/50/942) by STAR (Dobin *et al.* 2015), and the raw number of aligned reads was calculated using the feature Counts software (Liao *et al.* 2014). The TPM (transcripts per million) value was calculated using the edgeR package (Robinson *et al.* 2010) and normalized using the TMM (trimmed mean of M-values) method. A heat map of the flavonoid biosynthetic gene expression pattern was constructed based on log_2_ (TPM + 1) values. DEGexp function with the MARS method in the R package DESeq2 v.1.54.0 (Love *et al.* 2014) was used for the differential expression analysis of the flavonoid biosynthetic genes in response to low-temperature stress, a threshold of adjusted *P* value ≤ 0.05 and an absolute value of log2 |fold-change| ≥ 1 were used to define differentially expressed genes (DEGs).

## Results

### Identification and number of flavonoid biosynthetic genes

To identify the flavonoid biosynthetic structural and regulatory (transcription factors) genes in Musaceae, we gathered the conserved domains of these gene families and used HMMER software (Pucker *et al.* 2020) to search for corresponding domains in genome assemblies (removing any allelic variants at single loci) from six Musaceae species (*Ensete glaucum, Musella lasiocarpa*, *Musa beccarii*, *M. acuminata*, *M. balbisiana* and *M. schizocarpa*). For comparisons, we also used flavonoid genes identified in the genome assemblies of Zingiberaceae (*Zingiber officinale,* ginger), four species of Poaceae (*Oryza sativa*, rice; *Brachypodium distachyon*, Brachypodium; *Avena longiglumis*; *Sorghum bicolor*, sorghum; and the dicot *Arabidopsis thaliana,* Arabidopsis, all diploid species. Almost all genes and transcription factors were found in all analysed plant families (Table 1). The number of biosynthetic structural genes in the Musaceae ranged from 65 to 74 and the number of regulatory genes ranged from 6 to 7, for a total of 71–80 flavonoid biosynthetic genes and transcription factors. These numbers are broadly similar to the numbers found in *Zingiber* (84) and Poaceae (64–103) species but two to three times greater than the number found in Arabidopsis (Tables 1, **see Supporting Information—Table S1**; detailed characteristics, including protein properties and subcellular localization prediction, are provided in **see Supporting Information—Table S3**). There were more early- than late-stage biosynthetic genes in each Musaceae species, with early/late ratios ranging from 1.25 to 2.82 (Table 1). This finding is similar to the situation in Arabidopsis. In contrast, ginger and three of the four Poaceae species have fewer EBGs than late ones, with early/late ratios of 0.55 in ginger and 0.29–0.56 in the three grasses. The fourth grass species, sorghum, is an exception, with a ratio of 1.10.

Although the total numbers of genes were similar across the monocot species represented, there were marked differences in the copy numbers of individual genes; this phenomenon was especially pronounced among the structural genes but less pronounced among the regulatory genes. Although there has been extensive multiplication of PAL and ANR in *Avena longiglumis* (Table 1), within the Musaceae, although the copy numbers of each gene are relatively constant and even invariant among the taxa, there is evidence of multiplication in the CHS and especially F3ʹ,5ʹH gene families when compared with the Poaceae. In contrast, there are fewer copies of the F3ʹH and UGT75C1 families in Poaceae than in Zingiberales. Among the transcription factor families, Musaceae had two or three members, while Poaceae usually had fewer. LAR and MYB (C1) have not been reported in Arabidopsis (Table 1).

### Chromosomal distribution of flavonoid biosynthetic genes

The flavonoid biosynthetic genes are distributed across all chromosomes in the six Musaceae species (**see Supporting Information—Fig. S1**), with some tandem duplications (shown in boxes in **see Supporting Information—Fig. S1**; in some cases, the genes were adjacent but not recognized as tandem duplications with flanking regions). Most of the flavonoid biosynthetic genes were distributed near the ends of the chromosome arms, except for the F3ʹ5ʹH genes. There is a cluster of F3ʹ5ʹH genes near the centromere of chromosome 2 in the *x* = 11 *Musa* species, *M. acuminata*, *M. balbisiana* and *M. schizocarpa*.

### Phylogenetic analysis of flavonoid biosynthetic genes in Musaceae and outgroups

**Figure 2.**
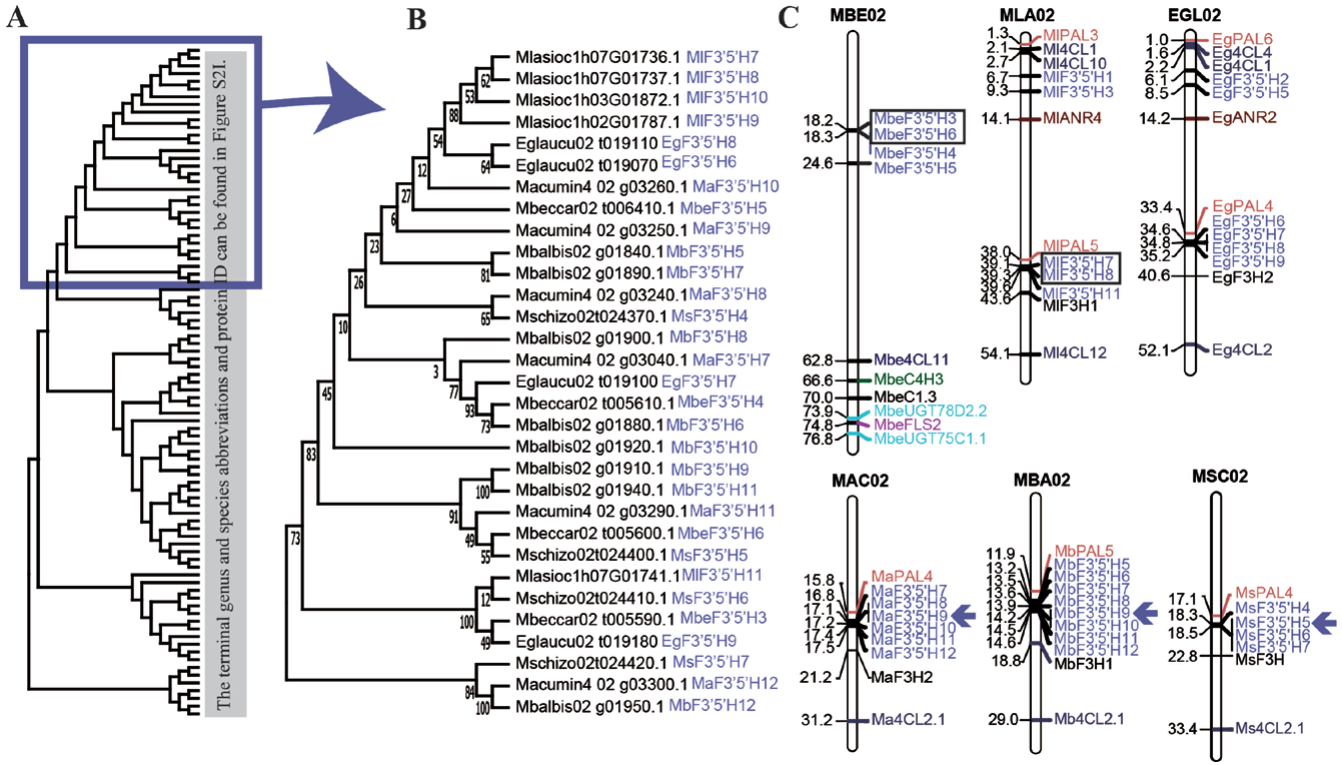
Phylogenetic tree and chromosomal location of *F3ʹ5ʹHs*. (A) Phylogenetic tree of the F3ʹ5ʹH gene family of Musaceae plus rice, Brachypodium, *Avena longiglumis*, sorghum, ginger and Arabidopsis as outgroups. (B) One clade of the F3ʹ5ʹH phylogenetic tree terminal with genus (initial letter) and species (six letter) abbreviations and (**C**) Chromosome 2 gene location maps in Musaceae. Genes from different families are shown in different colours (F3ʹ5ʹH in purple). Horizontal bars represent the gene locations (Mb, megabases) on syntenic chromosome 2 in Fig. 2C. Genes with tandem duplications are framed by black boxes; F3ʹ5ʹH clusters near the chromosome 2 centromere are indicated by arrows. MBE, Mbeccar, *M. beccarii*; MLA, Mlasioc, *M. lasiocarpa*; EGL, Eglaucu, *E. glaucum*; MAC, Macumin, *M. acuminata*; MBA, Mbalbis, *M. balbisiana*; MSC, Mschizo, *M. schizocarpa. *A colour version of this figure appears in the online version of this article.

Phylogenetic trees were constructed using the amino acid sequences of 17 flavonoid biosynthetic genes in six Musaceae species, ginger, four grasses and Arabidopsis (Fig. 2 and **see Supporting Information—Fig. S1**). For Musaceae, 50 out of 72 terminal branches had a gene from all six species, showing multi-copy gene duplication and that some diversification occurred before speciation. The phylogeny of the *F3ʹ5’H* gene family (amplified in Musaceae; Table 1) was more complex, revealing a new clade of *Musella lasiocarpa* genes with tandem duplication of the genes (Fig. 2). Around this clade, there were notable clusters of *F3’5’H* around the chromosome 2 centromere in *Musa acuminata*, *M. balbisiana* and *M. schizocarpa* (Fig. 2).

### Identification of duplicated (paralogous) gene pairs of flavonoid biosynthetic genes in Musaceae

**Figure 3.**
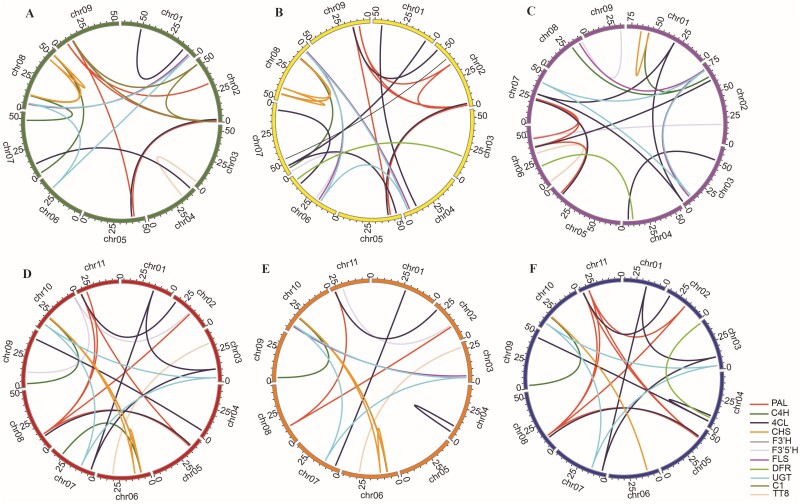
Circos plot of flavonoid biosynthetic gene paralogue locations on the chromosomes of six Musaceae species. (A) *E. glaucum* (green). (B) *M. lasiocarpa* (yellow). (C) *M. beccarii* (purple). (D) *M. acuminata* (red). (E) *M. balbisiana* (orange). (F) *M. schizocarpa* (blue). Different gene families are represented by different coloured lines. A colour version of this figure appears in the online version of this article.

The Musaceae family shares ancestral whole-genome duplication events, with three copies of most of the genes being found (D’Hont *et al.* 2012; Wang *et al.* 2022). We analysed the locations and changes in the copy numbers of paralogous flavonoid biosynthetic genes with respect to genome reorganization and chromosome recombination and identified 17–25 segmental duplicated gene pairs and 3–5 tandemly duplicated gene pairs in the *E. glaucum, M. lasiocarpa, M. beccarii*, *M. acuminata*, *M. balbisian* and *M. schizocarpa* genomes (Fig. 3 and **see Supporting Information—Table S5**). In these six species, genes in the PAL, C4H, 4CL, CHS and UFGT families always exhibited segmental duplication events. Tandem duplication events also played a part in UFGT amplification in Musaceae.

### Identification of orthologous gene pairs of flavonoid biosynthetic genes in Musaceae

On average, 77 orthologous gene pairs were detected between the Musaceae species (**see Supporting Information—Fig. S3** and Table 1). There is well characterized and extensive chromosomal recombination (translocations, inversions and fusion/fission events) between the Musaceae species with chromosome basic number *x* = 9 and 11 (Droc *et al.* 2022; Wang *et al.* 2022): the genes for the flavonoid biosynthetic pathway are within syntenic blocks, and their rearrangements across species follow the pattern of other genes [red lines linking flavonoid pathway genes; the synteny (collinearity) of 27 021 genes is shown by the grey underlying lines].

**Figure 4.**
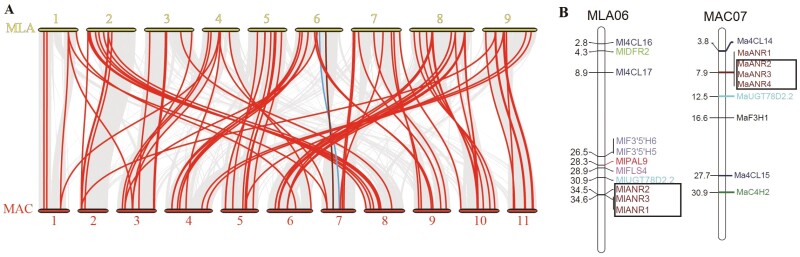
Synteny analysis and chromosomal location of *ANRs.* (A) Synteny analysis of flavonoid biosynthetic genes identified between MLA (*M. lasiocarpa*) and MAC (*M. acuminata*). The grey lines indicate all collinear gene blocks, while the red lines highlight the syntenic flavonoid biosynthetic gene pairs. The brown and blue lines indicate the ANR and UGT syntenic blocks, respectively. (B) Chromosome localization of flavonoid biosynthetic genes identified in *M. lasiocarpa* and *M. acuminata*. Genes from different families are shown in different colours. The chromosome number is shown at the top of each bar. The horizontal bars represent the gene locations on each chromosome (Mb). *ANR* genes with tandem duplications are framed by black boxes. A colour version of this figure appears in the online version of this article.

The *ANR* gene family has higher copy numbers (4 or 5) in *Musella lasiocarpa* and *Musa acuminata* than in other Musaceae species due to tandem triplication on chromosome 6 and chromosome 7 (Fig. 4, **see Supporting Information—Fig. S1**, and Table 1). Interestingly, this triplicated collinear block identified in *M. lasiocarpa* and *M. acuminata* was not found in other Musaceae species, suggesting that the tandem triplication event may have occurred independently in *M. lasiocarpa* and *M. acuminata* (Fig. 4B).

For Musaceae, we calculated the Ka/Ks ratios and divergence times for orthologous genes (**see Supporting Information—Table S4**). The results showed that most (1166 out of 1169 comparisons) of the Ka/Ks ratios were less than 1, consistent with ongoing stabilizing or purifying selection with no positive selection in any of the Musaceae. The Ka/Ks ratios of three gene pairs in the UGT gene family were greater than 1 (*MaUGT75C1* vs. *MbUGT75C1*, *MaUGT79B1* vs. *MsUGT79B1* and *MbUGT75C1* vs. *MsUGT75C1.4*), perhaps indicating slight (beneficial) positive selection pressure.

### Prediction of *cis*-acting elements in the putative gene promoters of flavonoid biosynthetic genes in Musaceae

Sequences (1.5 kb) upstream of the translation initiation codon for the flavonoid biosynthetic genes were examined for the presence of *cis*-acting elements using the PlantCARE online database with default settings. In addition to *cis*-acting elements that are characteristic of eukaryotic promoters, various *cis*-acting elements, including those associated with phytohormones, light responses, plant growth, development and stress responses (Alam *et al.* 2019), have been found among flavonoid biosynthetic genes. The *cis*-acting elements in the promoter region associated with stress responses (MYB and MYC elements), light responses (Box 4 and G-box), and phytohormones (ABRE, CGTCA-motif and TGACG-motif) were present in almost all the flavonoid biosynthetic genes of Musaceae (**see Supporting Information—Fig. S4**). In addition to the *4CL* genes, all *CHS* gene promoters contain candidate ABREs and G-box elements, namely, the stress responsive elements (Xia *et al.* 2021).

### Expression of flavonoid biosynthetic genes under low temperature

**Figure 5.**
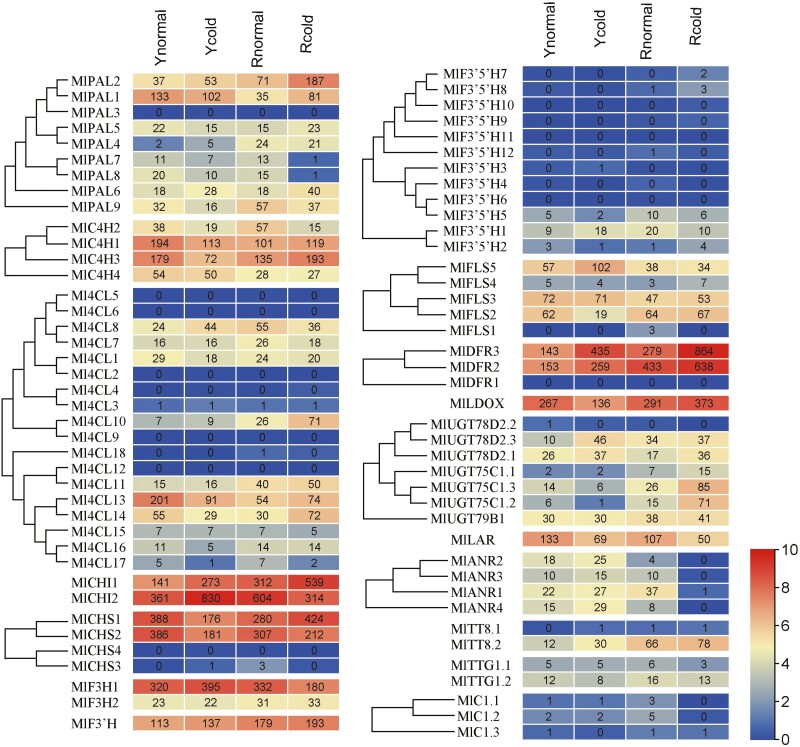
The expression of flavonoid biosynthetic genes in *Musella lasiocarpa* var. *lasiocarpa* (circles labelled Y) and *M. lasiocarpa* var. *rubribracteata* (labelled R); 15 °C-treated groups labelled with ‘cold’, and control groups marked with ‘normal’. The flavonoid biosynthetic gene expression levels were calculated as transcripts per million (TPM) values. Different log_2_(TPM + 1) values labelled with different colours, red to blue colours indicate expression levels from high to low. A colour version of this figure appears in the online version of this article.

The expression levels of flavonoid biosynthetic genes were analysed in *Musella lasiocarpa* var. *lasiocarpa* (yellow bracts) and *M. lasiocarpa* var. *rubribracteata* (red bracts) grown under low-temperature treatment (15 °C) for 48 h and at control temperature (25 °C). Among the 80 flavonoid biosynthetic genes, under both conditions, 18 were expressed at very low levels (transcripts per million TPM value < 1) in both the yellow- and red-bracted varieties and all *F3ʹ5ʹH* genes and the transcription factors TTG and C1 had low TPMs (<21), while 11 genes (*CHI*, *C4H*, *F3H*, *F3’H*, *DFR* and *LDOX* genes) were more strongly expressed (most with TPM value > 100) (Fig. 5 and **see Supporting Information—Table S5**). In other gene families, some members were less expressed than others in all four samples, with some being more strongly expressed in var. *lasiocarpa* (Y) than in var. *rubribraceata* (R) (eg. *PAL1*, *4CL13*, *FLS5*), or vice-versa (e.g. *PAL2*, *PAL9*, *4CL10*, *C1.3*, and most strongly, *TT8.2*). There were 13 and 26 flavonoid biosynthetic genes differentially expressed in yellow and red bracts after low-temperature treatment, respectively (**see Supporting Information—Table S7**). In particular, the expression levels of *MlDFR 3* significantly increased in both yellow and red bracts.

## Discussion

### Flavonoid biosynthetic genes and copy number changes in Musaceae

Musaceae, with well-defined species in three genera with Asian and African natives and high-quality chromosome-level genome assemblies, is a good model taxon to understand the evolution of flavonoid gene diversity. All the structural gene families annotated in Poaceae and Arabidopsis are found in Musaceae (Table 1). Based on a syntenic homology analysis, 74, 80, 74, 80, 76 and 71 flavonoid biosynthetic genes were identified in *E. glaucum, Musella lasiocarpa, Musa beccarii, M. acuminata*, *M. balbisiana* and *M. schizocarpa*, respectively, representing homologues of 34 out of the 40 flavonoid biosynthetic genes in Arabidopsis (Fig. 1, Table 1). The analysis of both structural and regulatory genes in the flavonoid pathway enables us to understand a variety of evolutionary and selective processes. The role of gene duplication, the nature of evolutionary rate variation among genes, the relative importance of structural and regulatory genes in the evolution of ecologically important characters (Rausher 2006), and the consequences of structural organization changes in the gene locations between species are important evolutionary questions. On average, 69 (65–74) structural genes encoding flavonoid biosynthetic enzymes and 7 (6–7) regulatory genes encoding transcription factors were identified in each species in the Musaceae.

In the Musaceae, we identified an average of 29 (25–34) flavonoid biosynthetic genes with segmental duplications (with substantial chromosomal regions) and 9 (6–13) flavonoid biosynthetic genes with tandem events (copies contiguous to each other; Fig. 3, **see Supporting Information—Table S5**). Only two TT8 bHLH transcription factors, which are required for the proper expression of several flavonoid biosynthetic genes, were duplicated in *E. glaucum, M. beccarii, M. acuminata* and *M. balbisiana*. Both tandem duplication and segmental duplication contribute to the amplification of structural gene number (Fig. 3), while segmental duplication contributes to the amplification of key regulatory gene number (Fig. 3: TT8 and C1). Our evaluation of evolutionary changes in gene copy number, complementing sequence evolution studies, has implications for taxonomy and phylogeny (Hollingsworth *et al.* 1999), evolutionary mechanisms, and characterization of genes under selective forces.

The copy number of flavonoid biosynthetic genes has expanded in Musaceae species compared with Arabidopsis, Brachypodium and rice. In Arabidopsis, 32 structural genes that encode flavonoid biosynthetic enzymes and 2 regulatory genes encoding transcription factors have been identified as homologous to genes in Musaceae (Guo *et al.* 2014; Li *et al.* 2019). The greater number of flavonoid biosynthetic genes in Musaceae than in Arabidopsis presumably reflects the ancient genome triplication that occurred in the family (D’Hont *et al.* 2012; Zhang *et al.* 2020): There were three chromosomal clusters of tandem duplication/triplication events in every Musaceae species (Figs. 2, **see Supporting Information—Fig. S1** and **see Supporting Information—Table S4**). Compared with those of Musaceae, Arabidopsis, sorghum and ginger lack a reported functional LAR gene (Table 1); consequently, proanthocyanin synthesis only results in the production of epicatechin (2,3-*cis*-flavan-3-ol), which is found in the seed coat and catalysed by ANR (encoded by the BANYULS gene) (Xie *et al.* 2003; Dixon *et al.* 2005; Li *et al.* 2019). Sequence and copy number evolution of F3ʹ5 ʹH are discussed below.

The phylogenetic trees (**see Supporting Information—Fig. S2**) show flavonoid biosynthetic genes in all Musaceae species analysed are usually clustered together, indicating that the duplications leading to their amplification occurred before the divergence of the species. Notably, the UGT genes of Arabidopsis, Poaceae, and ginger were clustered into UGT75C1, UGT78D2 and UGT79B1 groups (**see Supporting Information—Fig. S2**), implying that the duplications leading to the amplification of the UGT75C1, UGT78D2 and UGT79B1 genes were ancient events, pre-dating the separation of Musaceae from the outgroups.

### Sequence variation, phylogeny and adaptation of flavonoid biosynthetic genes in Musaceae

Analysis of nucleotide variation of flavonoid genes (**see Supporting Information—Table S4**) shows that most (1166 out of 1169 comparisons) of the non-synonymous to synonymous (Ka/Ks) ratios were less than 1, consistent with ongoing stabilizing or purifying selection with no positive selection at sequence level in Musaceae. Here, most of the diploid Musaceae accessions are non-domesticated wild species. Do the results (Table 1) show evidence that the species selected as crops (that is, *M. acuminata* as the desert banana, A genome, and *M. balbisiana*, B genome, often in hybrids with *M. acuminata*, for starchy fruits), show pre-adaptation, before domestication? An increase in flavonoid pathway genes might reflect increased protection against biotic and abiotic stresses (Zhang *et al.* 2019), and colouration might be a quality attribute. Cenci *et al.* (2019) suggested that the presence of the B genome was associated with increased expression of genes related to flavonoid biosynthesis, so this may be identified here (cf Fig. 5, rather than in gene copy number). The extreme plasticity of transcriptional regulation, leading to novel colouration phenotypes (Nair *et al.* 2005), has been shown by Recinos and Pucker (2024), so variation in genes and regulation is likely to be more important than gene copy number. Nevertheless, the 5- to 10-fold increase in the copy number of *F3ʹ5 ʹH* genes in Zingiberales compared with that in Poaceae suggested the selection of multiple copies in monocots, as proven by the greater number of 4CL gene copies than other genes in the pathway. In a genome-wide study, Rijzaani *et al.* (2022) proposed that a selective sweep through certain genomic regions might explain the genetic differentiation between *Ensete* and *Musa*, occurring at coding sequence and expression levels rather than copy number (Table 1, Fig. 3). For example, some genes (GO terms) in highly differentiated regions are related to flavonoid biosynthesis and defense responses.

### Early biosynthetic genes were more conserved than LBGs among Musaceae species

Changes in biosynthesis of flavonoids, as seen in Fig. 5, would be expected to alter the fitness of the plant (Guo *et al.* 2014). Products of the flavonoid pathway (Fig. 1) are involved in multiple plant characters, and pleiotropic effects from the simultaneous expression of unrelated genes controlled by shared transcription factors may also be involved (Dwivedi *et al.* 2016, 2024), and some researchers reported that transcription factors evolved faster than their structural genes in the flavonoid pigment pathway (Rausher *et al.* 1999; Wheeler *et al.* 2022).

The flavonoid gene copy number ratio of Musaceae/Poaceae is 1.16 for the EBGs and 0.74 for the late biosynthetic genes (using data from Table 1; average early/late biosynthetic gene copy number in Musaceae divided by that of Poaceae, largely because of the high copy number of the *F3’5’H* gene). Upstream genes (phenylpropanoid pathway and EBGs; Table 1) in the flavonoid pathway have evolved more slowly (**see Supporting Information—Fig. S2**) than the late biosynthetic genes, and these different evolutionary rates may be caused by upstream genes being more conserved due to involvement in multiple biochemical pathways (Fig. 1; Nair *et al.* 2005).

### Flavonoid 3ʹ,4ʹ,5ʹ-hydroxylase *F3*ʹ*5*ʹ*H* genes show amplification in monocots

F3ʹ5ʹH is a key branch-point in the flavonoid biosynthetic pathway (Fig. 1), generating 3ʹ,4ʹ,5ʹ-hydroxylated flavonoids (Wang *et al.* 2014), which are regarded as giving quality attributes, for example to tea (Jin *et al.* 2017). F3ʹH and F3ʹ5ʹH are members of the cytochrome P450 enzyme family (Park *et al.* 2021; Zhang *et al.* 2022). McClean *et al.* (2022) reported a phylogenetic analysis suggesting F3ʹ5ʹH first appeared in the Streptophyta, as it is present in only 41% of angiosperm genomes. In contrast, F3ʹH is found universally in angiosperms, due to its critical role in abiotic stress, and *F3*ʹ*5* ʹ*H* was reported as a major gene for the flower and seed coat colour regulation in plants (McClean *et al.* 2022), with MYB and MYC elements in the promoter region. Nevertheless, multiple copies of the F3ʹ5ʹH family genes were found in Arabidopsis, Brachypodium, sorghum, ginger, *E. glaucum, Musella lasiocarpa, Musa beccarii, M. acuminata, M. balbisiana* and *M. schizocarpa* (**see Supporting Information—Fig. S2**; Table 1), with amplification in monocots before separation of Poales and Zingiberales. The ratio of gene number of *F3ʹ5ʹH* (average Musaceae/average Poaceae) was 7.33. We identify that further proliferation of F3ʹ5ʹH genes occurred at one of the loci after the separation of Musaceae and gingers; one clade of *F3ʹ5ʹH* genes was specific to Musaceae (Fig. 2). The centromeric locations of duplicated *MaF3ʹ5ʹH*, *MbF3ʹ5ʹH*, and *MsF3ʹ5ʹH* on chromosome 2 suggest tandem duplication by unequal crossing over (Fig. 2): clusters of *F3ʹ5ʹH* are near the chromosome 2 centromere of *M. acuminata, M. balbisiana* and *M. schizocarpa*. The non-centromeric *F3ʹ5ʹH* genes in Musaceae are close to those of species in other families, while the centromeric *F3ʹ5ʹH* genes in Musaceae are relatively distant from the others in the phylogeny (Fig. 2), perhaps suggesting different origins of the centromeric *F3ʹ5ʹH* genes. Seitz *et al.* (2006 and 2015) reported multiple origins of *F3ʹ5ʹH* from *F3ʹH* precursors at least four times in dicotyledonous plants, while Negi *et al.* (2023) reported that the functional divergence of the 5ʹ regulator region of *F3ʹ5ʹH* related to stress response flavonoid phenotype of the Musaceae is underexplored, particularly with respect to non-anthocyanin flavonoids, and very little can be said about any relationship between *F3ʹ5ʹH* copy number and 3ʹ5ʹ-O flavonoid diversity. Available data show that B-ring 3ʹ5ʹ-oxygenation (as myricetin or delphinidin and its derivatives) occurs in two species of *Ensete* and nine species of *Musa*, most of which have not been investigated (Gornall unpublished).

### Flavonoid biosynthetic genes were expressed at different levels in the bract tissues of Musella lasiocarpa

Similar to the gene expression levels in *Musa acuminata* bracts (Pandey *et al.* 2016), *MlF3H1*, *MlF3’H*, *MlCHI1/2*, *MlFLS2/3/5* and *MlLDOX* genes were expressed at high levels in the bracts of *M. lasiocarpa* (Fig. 5). In particular, the *LDOX* (one copy only) was expressed at a high level (TPM values of 135.6–373.1) (Fig. 5): the spatial correlation between *LDOX* gene expression and anthocyanin metabolites in banana (cv. Grand Naine) bract tissue supports the importance of LDOX enzyme in the conversion of leucoanthocyanidins to anthocyanidins (Busche *et al.* 2021). Because of their powerful antioxidant activity, accumulation of flavonoids is likely to counteract oxidative damage induced by various stresses (Reginata *et al.* 2014). The accumulation of anthocyanins gives the bracts of Musaceae yellow or red colours (Pazmiño-Durán *et al.* 2001), with relatively high expression of *MlF3H1* and *MlF3ʹH* (TPM > 110, Fig. 5) regulating synthesis of orange-yellow pelargonidin and the purplish-red cyanidin (Fig. 1) pigment. Cis-acting regulatory elements are present in the promoter region of many genes, and PlantCARE (Lescot *et al.* 2002) was able to associate elements with nearly all the flavonoid biosynthetic genes of Musaceae (**see Supporting Information—Fig. S4**). After low-temperature treatment, the expression level of *MlDFR2*/*3*, *MlLDOX and MlUGT75C1.1/78D2.1/78D2.3* increased, while *MlLAR* expression level decreased under cold conditions (Fig. 1). It suggests increased leucoanthocyanidins were synthesized from DFR in the bracts, and most of them were synthesized into anthocyanins by LDOX and UGT rather than proanthocyanidins by LAR (Figs. 1 and 5). The expression levels of the *C1* genes from the MYB family, a positive regulator of the flavonoid biosynthetic pathway, were always decreased or unchanged in the yellow and red bract tissues of *M. lasiocarpa* (Zhang *et al.* 2015).

### Flavonoid pathway in evolution and crop improvement

Musaceae has three well-defined genera and is a concise model for understanding the range and nature of flavonoid gene diversity, exploiting uniformly-curated chromosome-level genome assemblies in the Banana Genome Hub (Droc *et al.* 2022). Knowledge of the range of diversity of genes in the flavonoid pathway across species has implications for taxonomy and phylogeny, and may help identify genes of importance for adaptation in the wild, or for exploitation in crops. Although the mechanisms of action of flavonoids in plant stress resistance, and in nutrition or medicine, are not fully understood (Panche *et al.* 2016; Dissanayake *et al.* 2020; Ullah *et al.* 2020; Nuraga *et al.* 2022), the diversity of flavonoid genes is valuable for functional analysis across all angiosperm species, and can be used for the development of approaches to modify flavonoid contents.

## Supporting Information

The following additional information is available in the online version of this article –

**Figure S1.** Chromosome localization of flavonoid biosynthetic genes identified in Musaceae.

**Figure S2.** Phylogenetic trees (Maximum-likelihood method) showing the genetic relationship of flavonoid biosynthetic genes in Arabidopsis, *B. distachyon*, *A. longiglumis*, *O. sativa*, *S. bicolor*, *Z. officinale*, *E. glaucum*, *Musella lasiocarpa*, *Musa beccarii*, *M. acuminata*, *M. balbisiana*, and *M. schizocarpa*.

**Figure S3.** Synteny analysis of FBGs between six Musaceae species.

**Figure S4.** *Cis*-acting elements in flavonoid biosynthetic genes of Musaceae.

**Table S1.** PFAM accession of the conserved domain of flavonoid biosynthetic protein.

**Table S2.** Flavonoid biosynthetic genes (FBGs) in *A. thaliana*, *B. distachyon*, *O. sativa*, *S. bicolor*, *Z. officinale*, *E. glaucum*, *Musella lasiocarpa, Musa beccarii, M. acuminata*, *M. balbisiana* and *M. schizocharpa.*

**Table S3.** Information on flavonoid biosynthetic proteins identified in Musaceae.

**Table S4.** Divergence time, Ka, Ks, and Ka/Ks values of orthologous flavonoid biosynthetic genes pairs between Musaceae.

**Table S5.** Intra-species pairs of duplicated genes.

**Table S6.** Expression of several flavonoid biosynthetic genes in *Musella lasiocarpa*.

**Table S7.** Expression of flavonoid biosynthetic gene in *Musella lasiocarpa*.

plae049_suppl_Supplementary_Materials

## Data Availability

The genomic DNA and inferred protein sequences of *Ensete glaucum*, *Musella lasiocarpa*, *Musa beccarii*, *M. acuminata*, *M. balbisiana*, and *M. schizocarpa* were downloaded from the Banana Genome Hub (https://banana-genome-hub.southgreen.fr/content/download); *Oryza sativa* (PRJDB2223), *Sorghum bicolor* (PRJNA13876), *Zingiber officinale* (PRJNA647255), and *Brachypodium distachyon* (PRJNA32607) protein sequences were downloaded from NCBI (https://www.ncbi.nlm.nih.gov); and *Avena longiglumis* (PRJNA956334) protein sequences were downloaded from Liu *et al.* (2024). The RNA-seq data of *Musella* bracts can be obtained in the Genome Sequence Archive for project PRJCA023025 (https://ngdc.cncb.ac.cn/gsa/browse/CRA014572). Hidden Markov Model (HMM) profiles of the characterized and conserved domains of flavonoid biosynthetic proteins (**see Supporting Information—Table S1**) were retrieved from the protein family database (https://www.ebi.ac.uk/interpro/). All data associated with this article are available in order to meet the publication standard. Any required links or identitfiers for data are present in this article as described.
