# Supplementary material for: Genome-wide analysis of flavonoid biosynthetic genes in Musaceae (Ensete, Musella, and Musa species) reveals amplification of flavonoid 3ʹ,5ʹ-hydroxylase
Source: AoB Plants. 2024 Sep 10;16(5):plae049. doi: 10.1093/aobpla/plae049 (PMC11500454; doi:10.1093/aobpla/plae049)
Supplement: plae049_suppl_Supplementary_Materials [file plae049_suppl_supplementary_materials.pdf]

Type of the Paper*:* Original Research Article

Genome-wide analysis of flavonoid biosynthetic genes in Musaceae (*Ensete*, *Musella*, and *Musa* species) reveals amplification of flavonoid 3',5'-hydroxylase

Dongli Cui[1,2,3], Gui Xiong[1,2,3], Lyuhan Ye[1,2,3], Richard Gornall[4], Ziwei Wang[5], Pat Heslop-Harrison[1,4]*, Qing Liu[1,2,6]*

[1] Key Laboratory of National Forestry and Grassland Administrationon Plant Conservation and Utilization in Southern China / Guangdong Provincial Key Laboratory of Applied Botany, South China Botanical Garden, Chinese Academy of Sciences, Guangzhou 510650, China

[2] South China National Botanical Garden, Guangzhou 510650, China

[3] University of Chinese Academy of Sciences, Beijing 100049, China

[4] University of Leicester, Department of Genetics and Genome Biology, Institute for Environmental Futures, Leicester LE1 7RH, UK

[5] Henry Fok School of Biology and Agriculture, Shaoguan University, Shaoguan 512005, China

[6] State Key Laboratory of Plant Diversity and Specialty Crops, South China Botanical Garden, Chinese Academy of Sciences, Guangzhou 510650, China

* Correspondence: phh4@le.ac.uk (PHH); liuqing@scib.ac.cn (QL)

A *M. acuminata*

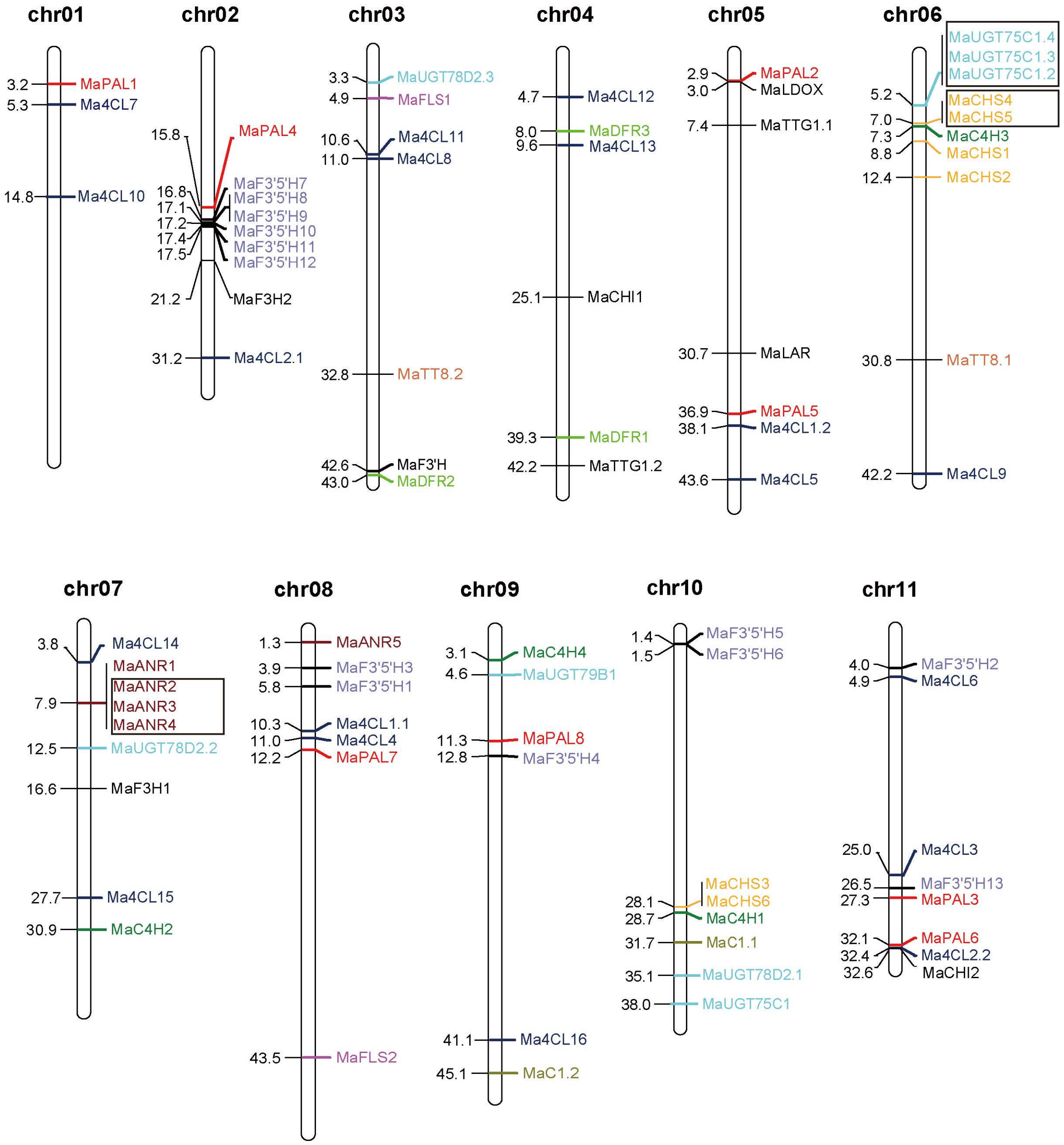

B *M. balbisiana*

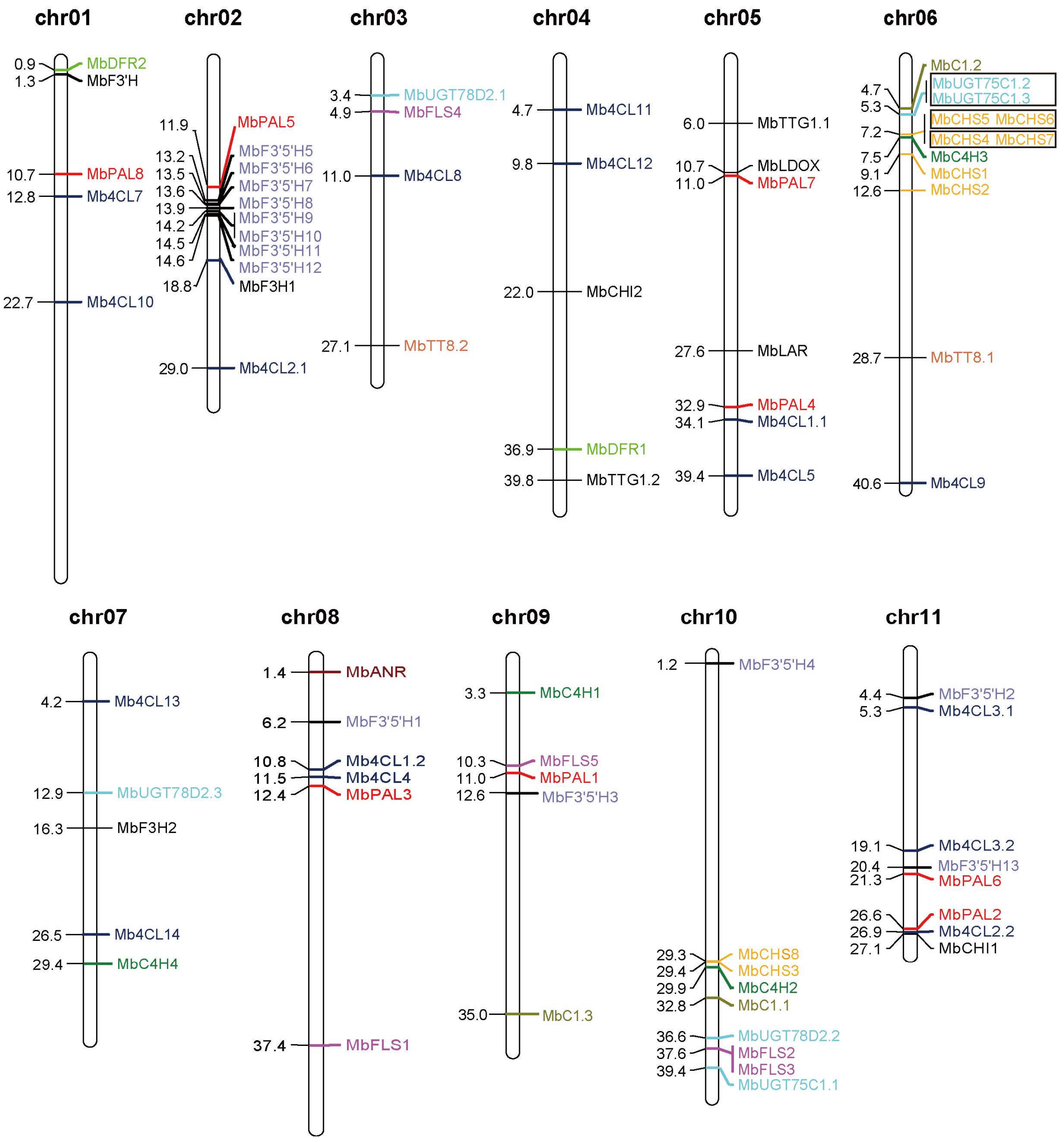

Cui *et al*. Flavonoid biosynthetic genes in Musaceae. AoB Plants 2024.

C *M. schizocharpa*

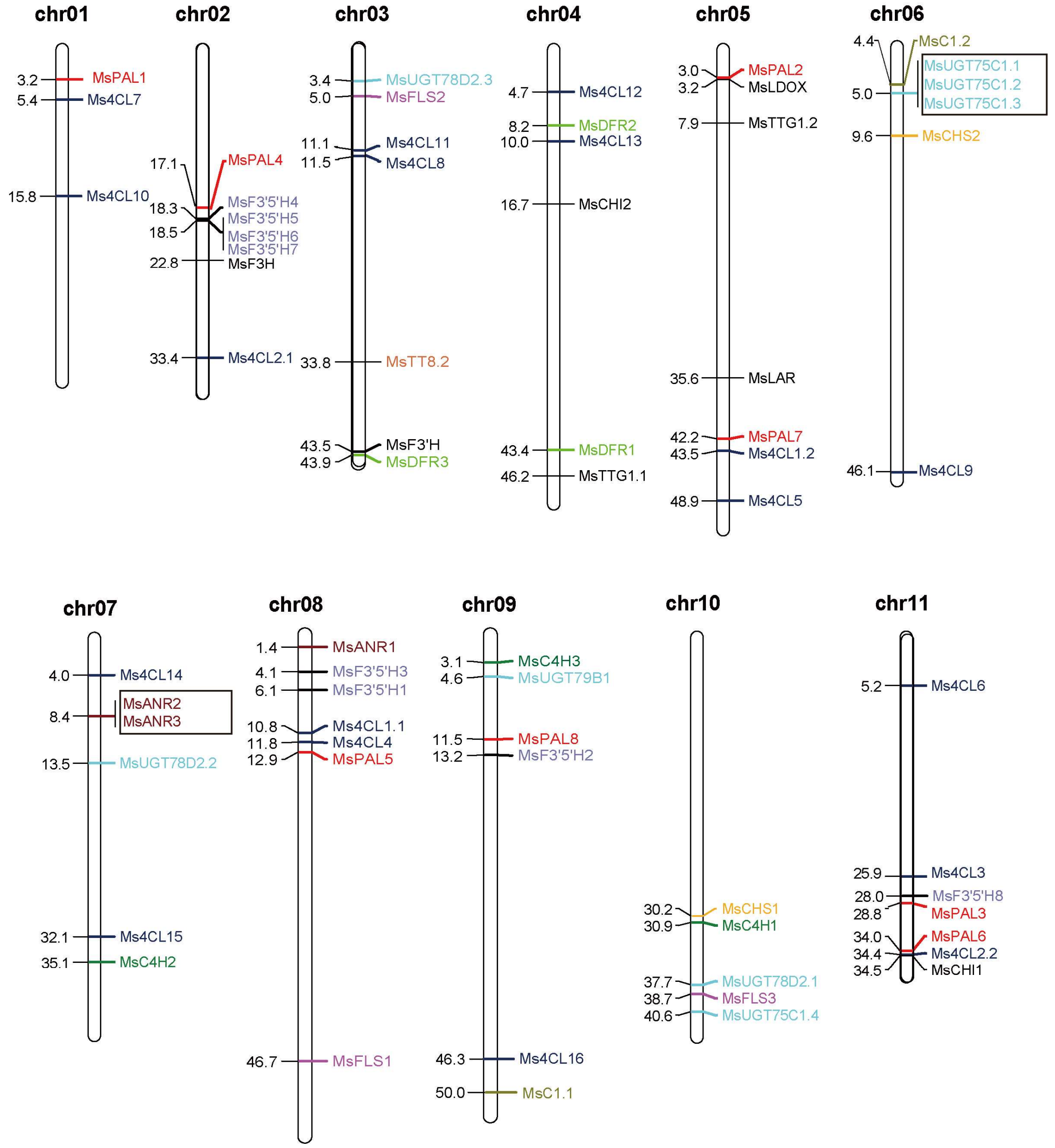

D *M. beccarii*

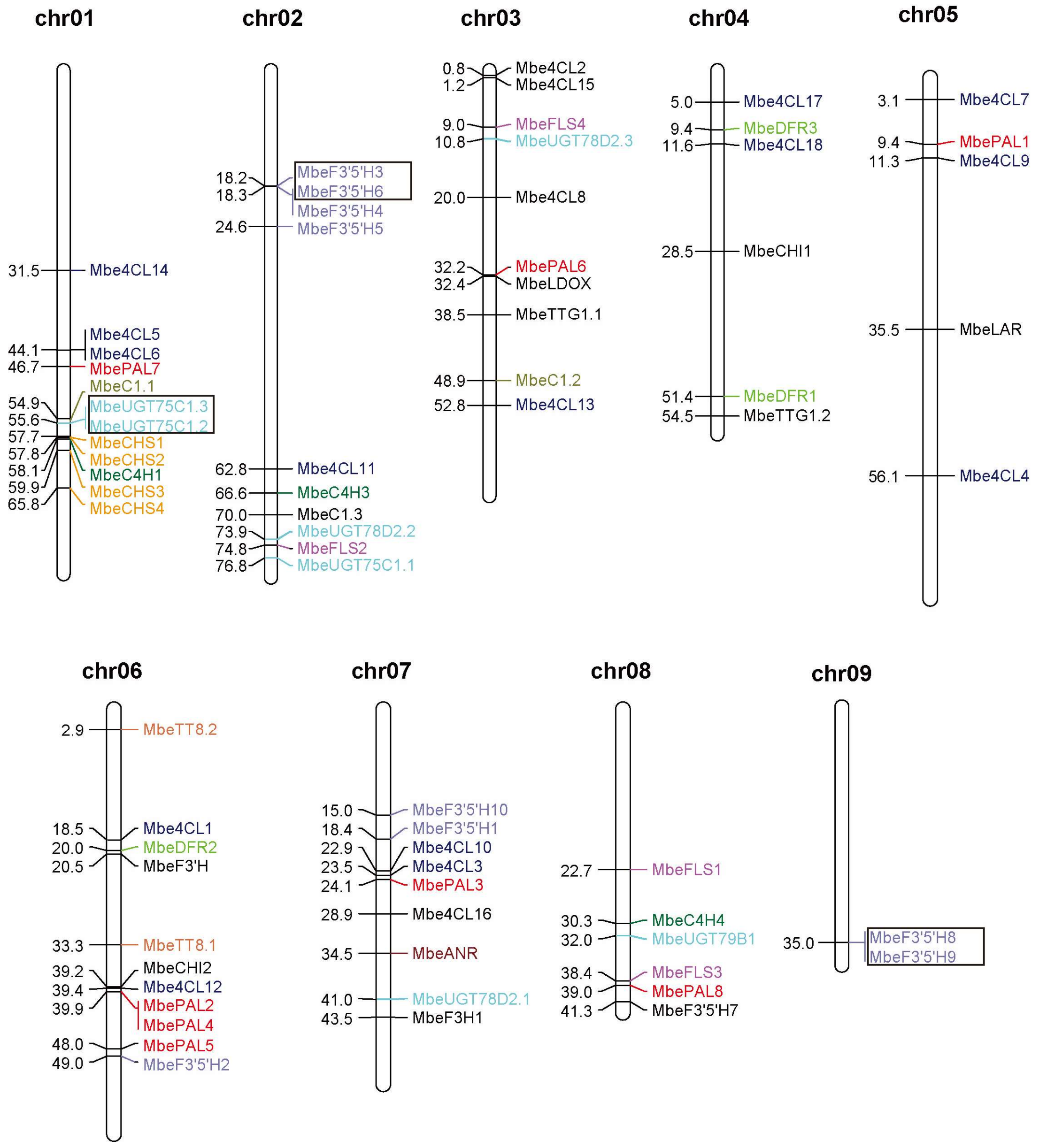
chr01
31.5 Mbe4CL14
Mbe4CL5
44.1 Mbe4CL6
46.7 MbePAL7
MbeC1.1
54.9 MbeUGT75C1.3
55.6 MbeUGT75C1.2
57.7 MbeCHS1
57.8 MbeCHS2
58.1 MbeC4H1
59.9 MbeCHS3
65.8 MbeCHS4
chr02
18.2 MbeF3'5'H3
18.3 MbeF3'5'H6
MbeF3'5'H4
24.6 MbeF3'5'H5
62.8 Mbe4CL11
66.6 MbeC4H3
70.0 MbeC1.3
73.9 MbeUGT78D2.2
74.8 MbeFLS2
76.8 MbeUGT75C1.1
chr03
0.8 Mbe4CL2
1.2 Mbe4CL15
9.0 MbeFLS4
10.8 MbeUGT78D2.3
20.0 Mbe4CL8
32.2 MbePAL6
32.4 MbeLDOX
38.5 MbeTTG1.1
48.9 MbeC1.2
52.8 Mbe4CL13
chr04
5.0 Mbe4CL17
9.4 MbeDFR3
11.6 Mbe4CL18
28.5 MbeCHI1
51.4 MbeDFR1
54.5 MbeTTG1.2
chr05
3.1 Mbe4CL7
9.4 MbePAL1
11.3 Mbe4CL9
35.5 MbeLAR
56.1 Mbe4CL4
chr06
2.9 MbeTT8.2
18.5 Mbe4CL1
20.0 MbeDFR2
20.5 MbeF3'H
33.3 MbeTT8.1
39.2 MbeCHI2
39.4 Mbe4CL12
39.9 MbePAL2
MbePAL4
48.0 MbePAL5
49.0 MbeF3'5'H2
chr07
15.0 MbeF3'5'H10
18.4 MbeF3'5'H1
22.9 Mbe4CL10
23.5 Mbe4CL3
24.1 MbePAL3
28.9 Mbe4CL16
34.5 MbeANR
41.0 MbeUGT78D2.1
43.5 MbeF3H1
chr08
22.7 MbeFLS1
30.3 MbeC4H4
32.0 MbeUGT79B1
38.4 MbeFLS3
39.0 MbePAL8
41.3 MbeF3'5'H7
chr09
35.0 MbeF3'5'H8
MbeF3'5'H9

E *M. lasiocarpa*

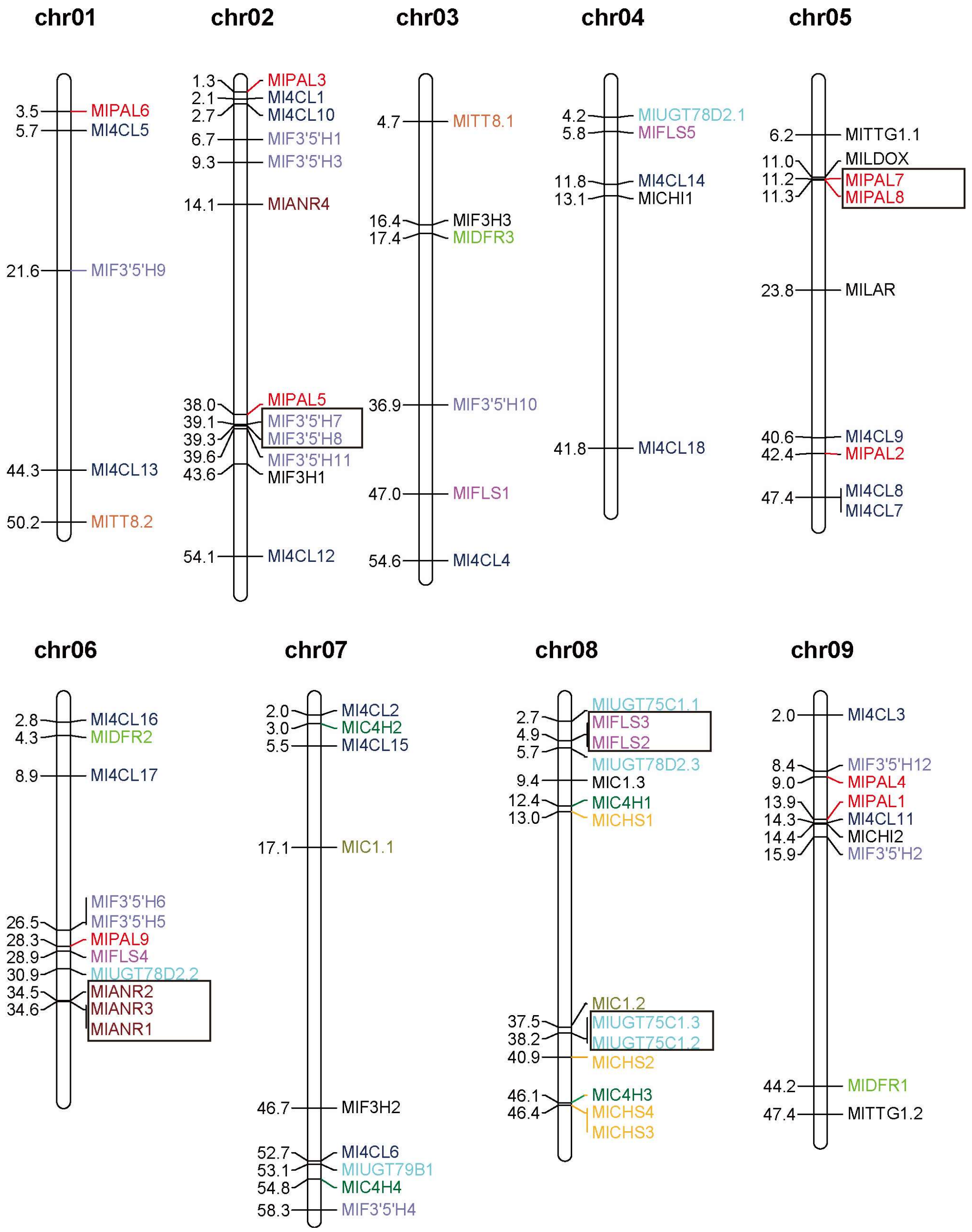

F *E, glaucum*

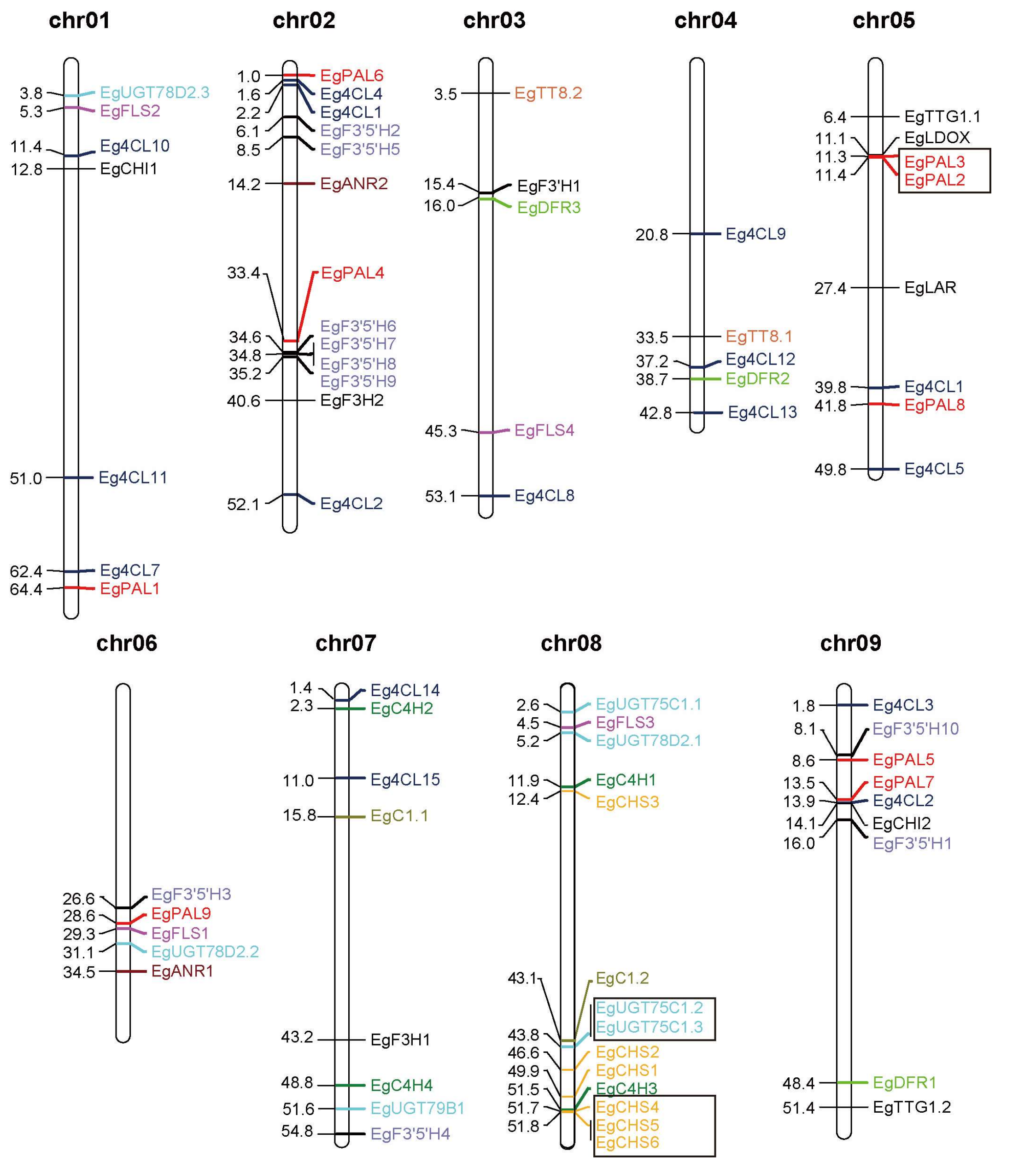

Figure S1. Chromosome localization of flavonoid biosynthetic genes (FBGs) identified in Musaceae. (**A**) FBGs in *Musa acuminata* (*x*=11). (**B**) FBGs in *M. balbisiana* (*x*=11). (**C**) FBGs in *M. schizocarpa* (*x*=11). (**D**) FBGs in *M. beccarii* (*x*=9). (**E**) FBGs in *Musella lasiocarpa* (*x*=9). (**F**) FBGs in *Ensete glaucum* (*x*=9).

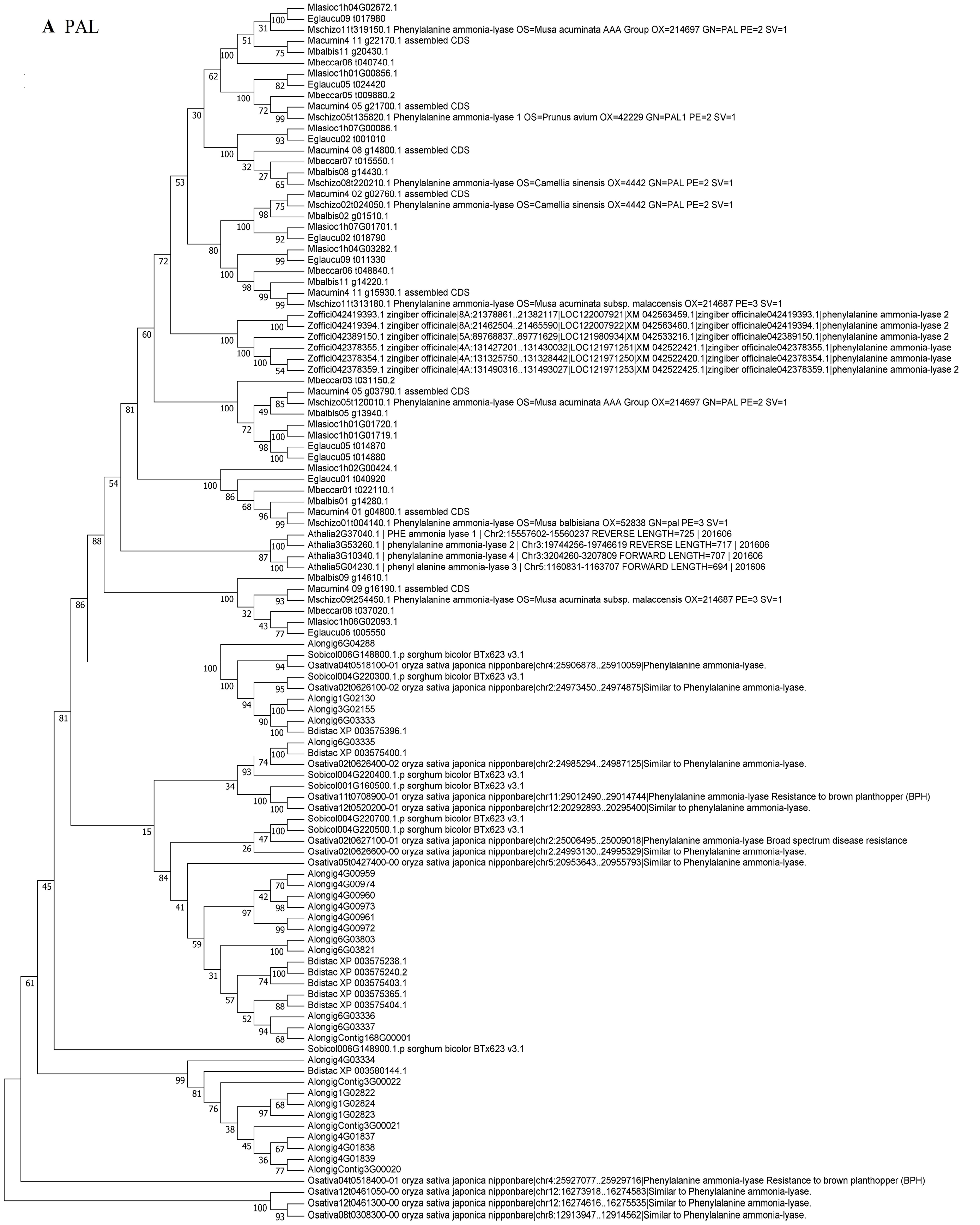
A PAL
Mlasioc1h04G02672.1
Eglaucu09 t017980
Mschizo11t319150.1 Phenylalanine ammonia-lyase OS=Musa acuminata AAA Group OX=214697 GN=PAL PE=2 SV=1
Macumin4 11 g22170.1 assembled CDS
Mbalbis11 g20430.1
Mbeccar06 t040740.1
Mlasioc1h01G00856.1
Eglaucu05 t024420
Mbeccar05 t009880.2
Macumin4 05 g21700.1 assembled CDS
Mschizo05t135820.1 Phenylalanine ammonia-lyase 1 OS=Prunus avium OX=42229 GN=PAL1 PE=2 SV=1
Mlasioc1h07G00086.1
Eglaucu02 t001010
Macumin4 08 g14800.1 assembled CDS
Mbeccar07 t015550.1
Mbalbis08 g14430.1
Mschizo08t220210.1 Phenylalanine ammonia-lyase OS=Camellia sinensis OX=4442 GN=PAL PE=2 SV=1
Macumin4 02 g02760.1 assembled CDS
Mschizo02t024050.1 Phenylalanine ammonia-lyase OS=Camellia sinensis OX=4442 GN=PAL PE=2 SV=1
Mbalbis02 g01510.1
Mlasioc1h07G01701.1
Eglaucu02 t018790
Mlasioc1h04G03282.1
Eglaucu09 t011330
Mbeccar06 t048840.1
Mbalbis11 g14220.1
Macumin4 11 g15930.1 assembled CDS
Mschizo11t313180.1 Phenylalanine ammonia-lyase OS=Musa acuminata subsp. malaccensis OX=214687 PE=3 SV=1
Zoffici042419393.1 zingiber officinale|8A:21378861..21382117|LOC122007921|XM 042563459.1|zingiber officinale042419393.1|phenylalanine ammonia-lyase 2
Zoffici042419394.1 zingiber officinale|8A:21462504..21465590|LOC122007922|XM 042563460.1|zingiber officinale042419394.1|phenylalanine ammonia-lyase 2
Zoffici042389150.1 zingiber officinale|5A:89768837..89771629|LOC121980934|XM 042533216.1|zingiber officinale042389150.1|phenylalanine ammonia-lyase 2
Zoffici042378355.1 zingiber officinale|4A:131427201..131430032|LOC121971251|XM 042522421.1|zingiber officinale042378355.1|phenylalanine ammonia-lyase
Zoffici042378354.1 zingiber officinale|4A:131325750..131328442|LOC121971250|XM 042522420.1|zingiber officinale042378354.1|phenylalanine ammonia-lyase
Zoffici042378359.1 zingiber officinale|4A:131490316..131493027|LOC121971253|XM 042522425.1|zingiber officinale042378359.1|phenylalanine ammonia-lyase 2
Mbeccar03 t031150.2
Macumin4 05 g03790.1 assembled CDS
Mschizo05t120010.1 Phenylalanine ammonia-lyase OS=Musa acuminata AAA Group OX=214697 GN=PAL PE=2 SV=1
Mbalbis05 g13940.1
Mlasioc1h01G01720.1
Mlasioc1h01G01719.1
Eglaucu05 t014870
Eglaucu05 t014880
Mlasioc1h02G00424.1
Eglaucu01 t040920
Mbeccar01 t022110.1
Mbalbis01 g14280.1
Macumin4 01 g04800.1 assembled CDS
Mschizo01t004140.1 Phenylalanine ammonia-lyase OS=Musa balbisiana OX=52838 GN=pal PE=3 SV=1
Athalia2G37040.1 | PHE ammonia lyase 1 | Chr2:15557602-15560237 REVERSE LENGTH=725 | 201606
Athalia3G53260.1 | phenylalanine ammonia-lyase 2 | Chr3:19744256-19746619 REVERSE LENGTH=717 | 201606
Athalia3G10340.1 | phenylalanine ammonia-lyase 4 | Chr3:3204260-3207809 FORWARD LENGTH=707 | 201606
Athalia5G04230.1 | phenyl alanine ammonia-lyase 3 | Chr5:1160831-1163707 FORWARD LENGTH=694 | 201606
Mbalbis09 g14610.1
Macumin4 09 g16190.1 assembled CDS
Mschizo09t254450.1 Phenylalanine ammonia-lyase OS=Musa acuminata subsp. malaccensis OX=214687 PE=3 SV=1
Mbeccar08 t037020.1
Mlasioc1h06G02093.1
Eglaucu06 t005550
Alongig6G04288
Sobicol006G148800.1.p sorghum bicolor BTx623 v3.1
Osativa04t0518100-01 oryza sativa japonica nipponbare|chr4:25906878..25910059|Phenylalanine ammonia-lyase.
Sobicol004G220300.1.p sorghum bicolor BTx623 v3.1
Osativa02t0626100-02 oryza sativa japonica nipponbare|chr2:24973450..24974875|Similar to Phenylalanine ammonia-lyase.
Alongig1G02130
Alongig3G02155
Alongig6G03333
Bdistac XP 003575396.1
Alongig6G03335
Bdistac XP 003575400.1
Osativa02t0626400-02 oryza sativa japonica nipponbare|chr2:24985294..24987125|Similar to Phenylalanine ammonia-lyase.
Sobicol004G220400.1.p sorghum bicolor BTx623 v3.1
Sobicol001G160500.1.p sorghum bicolor BTx623 v3.1
Osativa11t0708900-01 oryza sativa japonica nipponbare|chr11:29012490..29014744|Phenylalanine ammonia-lyase Resistance to brown planthopper (BPH)
Osativa12t0520200-01 oryza sativa japonica nipponbare|chr12:20292893..20295400|Similar to phenylalanine ammonia-lyase.
Sobicol004G220700.1.p sorghum bicolor BTx623 v3.1
Sobicol004G220500.1.p sorghum bicolor BTx623 v3.1
Osativa02t0627100-01 oryza sativa japonica nipponbare|chr2:25006495..25009018|Phenylalanine ammonia-lyase Broad spectrum disease resistance
Osativa02t0626600-00 oryza sativa japonica nipponbare|chr2:24993130..24995329|Similar to Phenylalanine ammonia-lyase.
Osativa05t0427400-00 oryza sativa japonica nipponbare|chr5:20953643..20955793|Similar to Phenylalanine ammonia-lyase.
Alongig4G00959
Alongig4G00974
Alongig4G00960
Alongig4G00973
Alongig4G00961
Alongig4G00972
Alongig6G03803
Alongig6G03821
Bdistac XP 003575238.1
Bdistac XP 003575240.2
Bdistac XP 003575403.1
Bdistac XP 003575365.1
Bdistac XP 003575404.1
Alongig6G03336
Alongig6G03337
AlongigContig168G00001
Sobicol006G148900.1.p sorghum bicolor BTx623 v3.1
Alongig4G03334
Bdistac XP 003580144.1
AlongigContig3G00022
Alongig1G02822
Alongig1G02824
Alongig1G02823
AlongigContig3G00021
Alongig4G01837
Alongig4G01838
Alongig4G01839
AlongigContig3G00020
Osativa04t0518400-01 oryza sativa japonica nipponbare|chr4:25927077..25929716|Phenylalanine ammonia-lyase Resistance to brown planthopper (BPH)
Osativa12t0461050-00 oryza sativa japonica nipponbare|chr12:16273918..16274583|Similar to Phenylalanine ammonia-lyase.
Osativa12t0461300-00 oryza sativa japonica nipponbare|chr12:16274616..16275535|Similar to Phenylalanine ammonia-lyase.
Osativa08t0308300-00 oryza sativa japonica nipponbare|chr8:12913947..12914562|Similar to Phenylalanine ammonia-lyase.

B 4CL

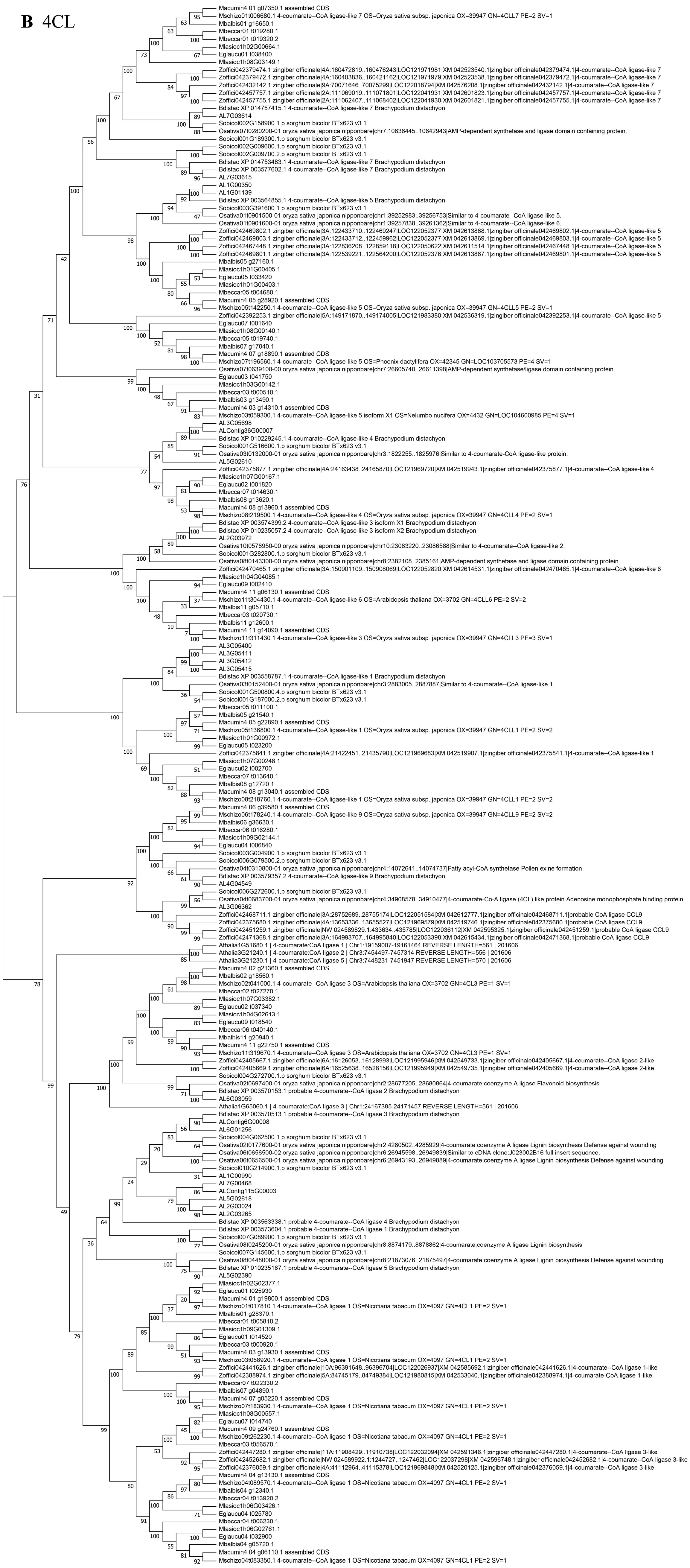

Cui *et al*. Flavonoid biosynthetic genes in Musaceae. AoB Plants 2024.

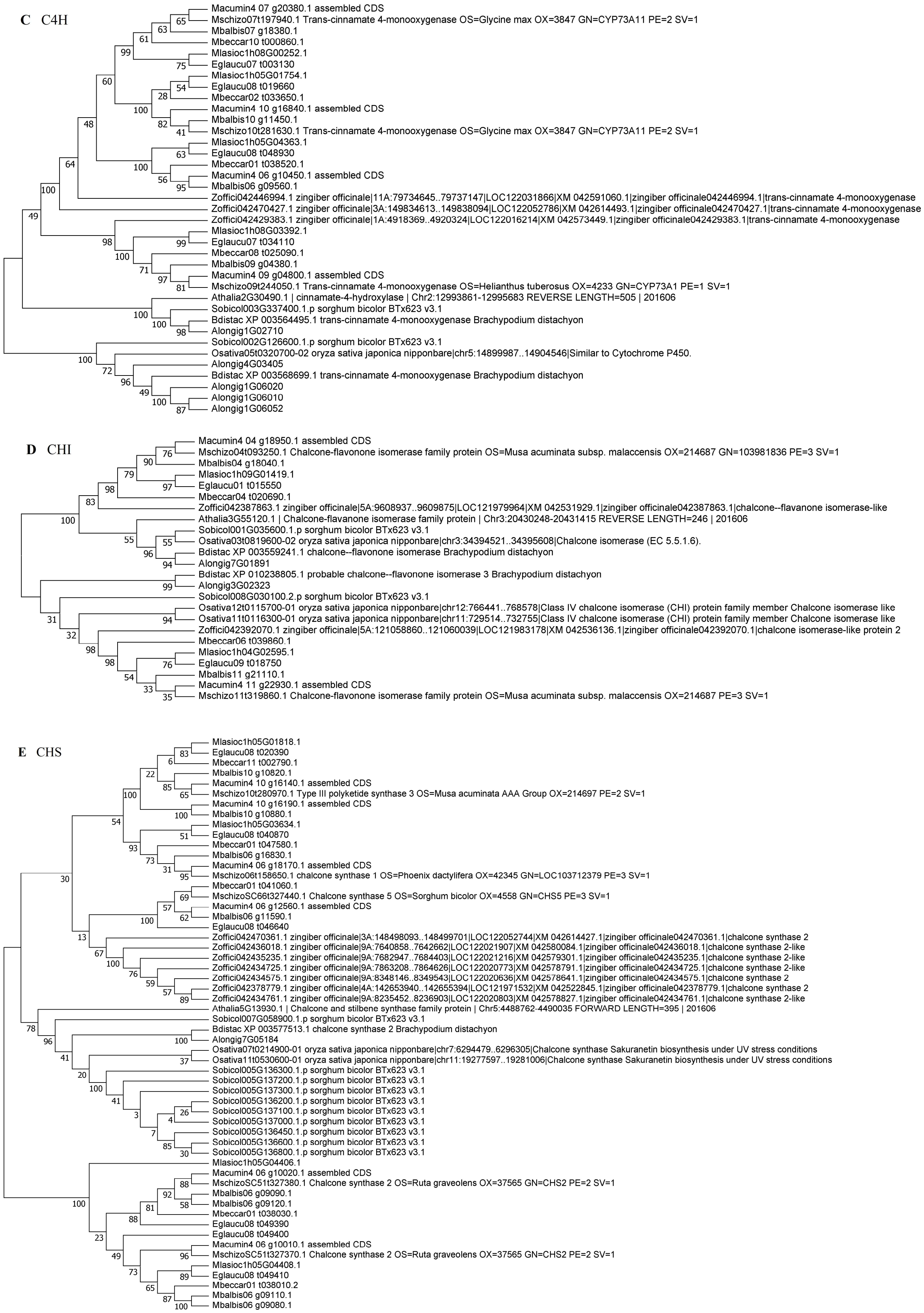
C C4H
Macumin4 07 g20380.1 assembled CDS
Mschizo07t197940.1 Trans-cinnamate 4-monooxygenase OS=Glycine max OX=3847 GN=CYP73A11 PE=2 SV=1
Mbalbis07 g18380.1
Mbeccar10 t000860.1
Mlasioc1h08G00252.1
Eglaucu07 t003130
Mlasioc1h05G01754.1
Eglaucu08 t019660
Mbeccar02 t033650.1
Macumin4 10 g16840.1 assembled CDS
Mbalbis10 g11450.1
Mschizo10t281630.1 Trans-cinnamate 4-monooxygenase OS=Glycine max OX=3847 GN=CYP73A11 PE=2 SV=1
Mlasioc1h05G04363.1
Eglaucu08 t048930
Mbeccar01 t038520.1
Macumin4 06 g10450.1 assembled CDS
Mbalbis06 g09560.1
Zoffici042446994.1 zingiber officinale|11A:79734645..79737147|LOC122031866|XM 042591060.1|zingiber officinale042446994.1|trans-cinnamate 4-monooxygenase
Zoffici042470427.1 zingiber officinale|3A:149834613..149838094|LOC122052786|XM 042614493.1|zingiber officinale042470427.1|trans-cinnamate 4-monooxygenase
Zoffici042429383.1 zingiber officinale|1A:4918369..4920324|LOC122016214|XM 042573449.1|zingiber officinale042429383.1|trans-cinnamate 4-monooxygenase
Mlasioc1h08G03392.1
Eglaucu07 t034110
Mbeccar08 t025090.1
Mbalbis09 g04380.1
Macumin4 09 g04800.1 assembled CDS
Mschizo09t244050.1 Trans-cinnamate 4-monooxygenase OS=Helianthus tuberosus OX=4233 GN=CYP73A1 PE=1 SV=1
Athalia2G30490.1 | cinnamate-4-hydroxylase | Chr2:12993861-12995683 REVERSE LENGTH=505 | 201606
Sobicol003G337400.1.p sorghum bicolor BTx623 v3.1
Bdistac XP 003564495.1 trans-cinnamate 4-monooxygenase Brachypodium distachyon
Alongig1G02710
Sobicol002G126600.1.p sorghum bicolor BTx623 v3.1
Osativa05t0320700-02 oryza sativa japonica nipponbare|chr5:14899987..14904546|Similar to Cytochrome P450.
Alongig4G03405
Bdistac XP 003568699.1 trans-cinnamate 4-monooxygenase Brachypodium distachyon
Alongig1G06020
Alongig1G06010
Alongig1G06052
D CHI
Macumin4 04 g18950.1 assembled CDS
Mschizo04t093250.1 Chalcone-flavonone isomerase family protein OS=Musa acuminata subsp. malaccensis OX=214687 GN=103981836 PE=3 SV=1
Mbalbis04 g18040.1
Mlasioc1h09G01419.1
Eglaucu01 t015550
Mbeccar04 t020690.1
Zoffici042387863.1 zingiber officinale|5A:9608937..9609875|LOC121979964|XM 042531929.1|zingiber officinale042387863.1|chalcone--flavanone isomerase-like
Athalia3G55120.1 | Chalcone-flavanone isomerase family protein | Chr3:20430248-20431415 REVERSE LENGTH=246 | 201606
Sobicol001G035600.1.p sorghum bicolor BTx623 v3.1
Osativa03t0819600-02 oryza sativa japonica nipponbare|chr3:34394521..34395608|Chalcone isomerase (EC 5.5.1.6).
Bdistac XP 003559241.1 chalcone--flavonone isomerase Brachypodium distachyon
Alongig7G01891
Bdistac XP 010238805.1 probable chalcone--flavonone isomerase 3 Brachypodium distachyon
Alongig3G02323
Sobicol008G030100.2.p sorghum bicolor BTx623 v3.1
Osativa12t0115700-01 oryza sativa japonica nipponbare|chr12:766441..768578|Class IV chalcone isomerase (CHI) protein family member Chalcone isomerase like
Osativa11t0116300-01 oryza sativa japonica nipponbare|chr11:729514..732755|Class IV chalcone isomerase (CHI) protein family member Chalcone isomerase like
Zoffici042392070.1 zingiber officinale|5A:121058860..121060039|LOC121983178|XM 042536136.1|zingiber officinale042392070.1|chalcone isomerase-like protein 2
Mbeccar06 t039860.1
Mlasioc1h04G02595.1
Eglaucu09 t018750
Mbalbis11 g21110.1
Macumin4 11 g22930.1 assembled CDS
Mschizo11t319860.1 Chalcone-flavonone isomerase family protein OS=Musa acuminata subsp. malaccensis OX=214687 PE=3 SV=1
E CHS
Mlasioc1h05G01818.1
Eglaucu08 t020390
Mbeccar11 t002790.1
Mbalbis10 g10820.1
Macumin4 10 g16140.1 assembled CDS
Mschizo10t280970.1 Type III polyketide synthase 3 OS=Musa acuminata AAA Group OX=214697 PE=2 SV=1
Macumin4 10 g16190.1 assembled CDS
Mbalbis10 g10880.1
Mlasioc1h05G03634.1
Eglaucu08 t040870
Mbeccar01 t047580.1
Mbalbis06 g16830.1
Macumin4 06 g18170.1 assembled CDS
Mschizo06t158650.1 chalcone synthase 1 OS=Phoenix dactylifera OX=42345 GN=LOC103712379 PE=3 SV=1
Mbeccar01 t041060.1
MschizoSC66t327440.1 Chalcone synthase 5 OS=Sorghum bicolor OX=4558 GN=CHS5 PE=3 SV=1
Macumin4 06 g12560.1 assembled CDS
Mbalbis06 g11590.1
Eglaucu08 t046640
Zoffici042470361.1 zingiber officinale|3A:148498093..148499701|LOC122052744|XM 042614427.1|zingiber officinale042470361.1|chalcone synthase 2
Zoffici042436018.1 zingiber officinale|9A:7640858..7642662|LOC122021907|XM 042580084.1|zingiber officinale042436018.1|chalcone synthase 2-like
Zoffici042435235.1 zingiber officinale|9A:7682947..7684403|LOC122021216|XM 042579301.1|zingiber officinale042435235.1|chalcone synthase 2-like
Zoffici042434725.1 zingiber officinale|9A:7863208..7864626|LOC122020773|XM 042578791.1|zingiber officinale042434725.1|chalcone synthase 2-like
Zoffici042434575.1 zingiber officinale|9A:8348146..8349543|LOC122020636|XM 042578641.1|zingiber officinale042434575.1|chalcone synthase 2
Zoffici042378779.1 zingiber officinale|4A:142653940..142655394|LOC121971532|XM 042522845.1|zingiber officinale042378779.1|chalcone synthase 2
Zoffici042434761.1 zingiber officinale|9A:8235452..8236903|LOC122020803|XM 042578827.1|zingiber officinale042434761.1|chalcone synthase 2-like
Athalia5G13930.1 | Chalcone and stilbene synthase family protein | Chr5:4488762-4490035 FORWARD LENGTH=395 | 201606
Sobicol007G058900.1.p sorghum bicolor BTx623 v3.1
Bdistac XP 003577513.1 chalcone synthase 2 Brachypodium distachyon
Alongig7G05184
Osativa07t0214900-01 oryza sativa japonica nipponbare|chr7:6294479..6296305|Chalcone synthase Sakuranetin biosynthesis under UV stress conditions
Osativa11t0530600-01 oryza sativa japonica nipponbare|chr11:19277597..19281006|Chalcone synthase Sakuranetin biosynthesis under UV stress conditions
Sobicol005G136300.1.p sorghum bicolor BTx623 v3.1
Sobicol005G137200.1.p sorghum bicolor BTx623 v3.1
Sobicol005G137300.1.p sorghum bicolor BTx623 v3.1
Sobicol005G136200.1.p sorghum bicolor BTx623 v3.1
Sobicol005G137100.1.p sorghum bicolor BTx623 v3.1
Sobicol005G137000.1.p sorghum bicolor BTx623 v3.1
Sobicol005G136450.1.p sorghum bicolor BTx623 v3.1
Sobicol005G136600.1.p sorghum bicolor BTx623 v3.1
Sobicol005G136800.1.p sorghum bicolor BTx623 v3.1
Mlasioc1h05G04406.1
Macumin4 06 g10020.1 assembled CDS
MschizoSC51t327380.1 Chalcone synthase 2 OS=Ruta graveolens OX=37565 GN=CHS2 PE=2 SV=1
Mbalbis06 g09090.1
Mbalbis06 g09120.1
Mbeccar01 t038030.1
Eglaucu08 t049390
Eglaucu08 t049400
Macumin4 06 g10010.1 assembled CDS
MschizoSC51t327370.1 Chalcone synthase 2 OS=Ruta graveolens OX=37565 GN=CHS2 PE=2 SV=1
Mlasioc1h05G04408.1
Eglaucu08 t049410
Mbeccar01 t038010.2
Mbalbis06 g09110.1
Mbalbis06 g09080.1

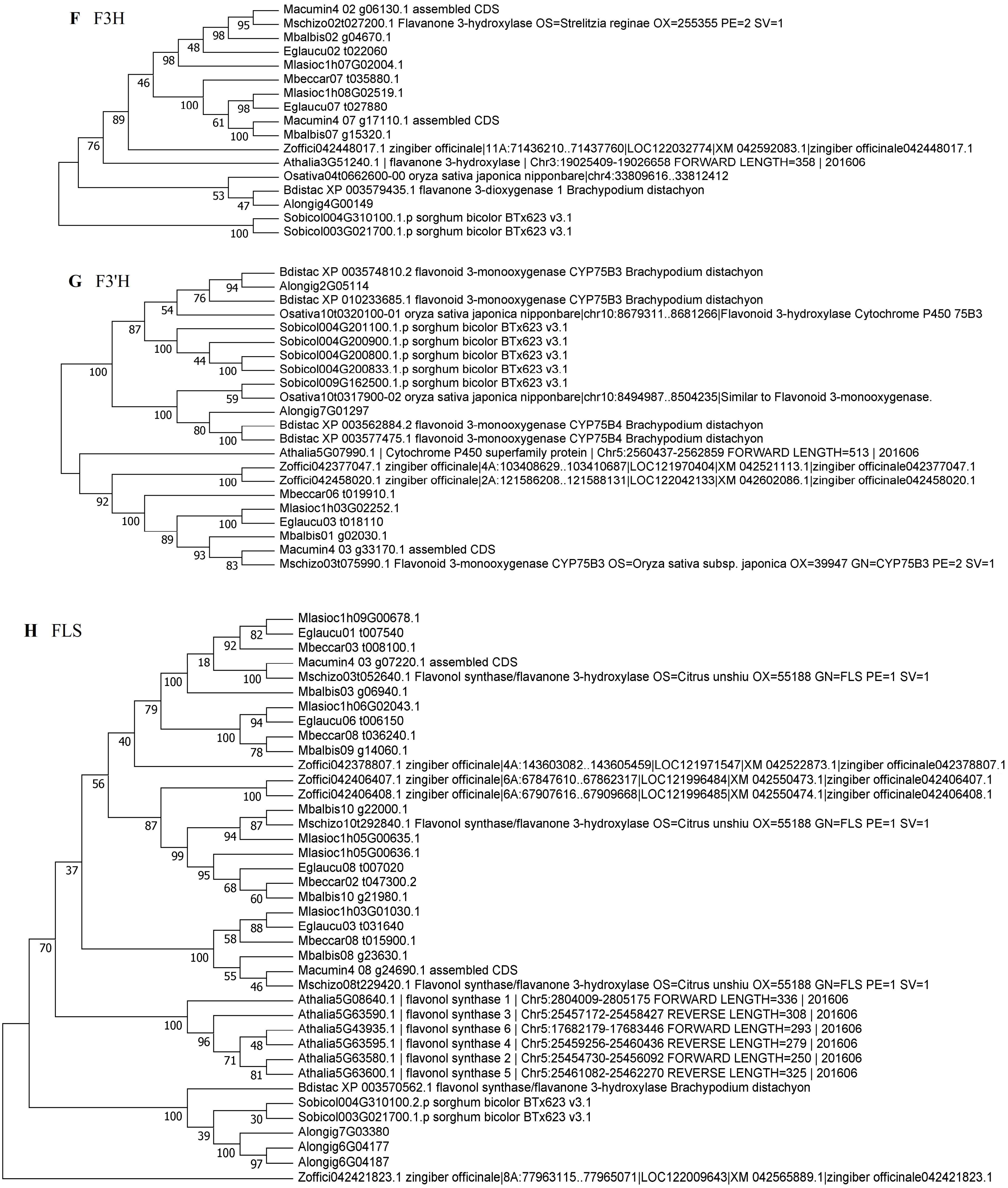
F F3H
Macumin4 02 g06130.1 assembled CDS
Mschizo02t027200.1 Flavanone 3-hydroxylase OS=Strelitzia reginae OX=255355 PE=2 SV=1
Mbalbis02 g04670.1
Eglaucu02 t022060
Mlasioc1h07G02004.1
Mbeccar07 t035880.1
Mlasioc1h08G02519.1
Eglaucu07 t027880
Macumin4 07 g17110.1 assembled CDS
Mbalbis07 g15320.1
Zoffici042448017.1 zingiber officinale|11A:71436210..71437760|LOC122032774|XM 042592083.1|zingiber officinale042448017.1
Athalia3G51240.1 | flavanone 3-hydroxylase | Chr3:19025409-19026658 FORWARD LENGTH=358 | 201606
Osativa04t0662600-00 oryza sativa japonica nipponbare|chr4:33809616..33812412
Bdistac XP 003579435.1 flavanone 3-dioxygenase 1 Brachypodium distachyon
Alongig4G00149
Sobicol004G310100.1.p sorghum bicolor BTx623 v3.1
Sobicol003G021700.1.p sorghum bicolor BTx623 v3.1
G F3'H
Bdistac XP 003574810.2 flavonoid 3-monooxygenase CYP75B3 Brachypodium distachyon
Alongig2G05114
Bdistac XP 010233685.1 flavonoid 3-monooxygenase CYP75B3 Brachypodium distachyon
Osativa10t0320100-01 oryza sativa japonica nipponbare|chr10:8679311..8681266|Flavonoid 3-hydroxylase Cytochrome P450 75B3
Sobicol004G201100.1.p sorghum bicolor BTx623 v3.1
Sobicol004G200900.1.p sorghum bicolor BTx623 v3.1
Sobicol004G200800.1.p sorghum bicolor BTx623 v3.1
Sobicol004G200833.1.p sorghum bicolor BTx623 v3.1
Sobicol009G162500.1.p sorghum bicolor BTx623 v3.1
Osativa10t0317900-02 oryza sativa japonica nipponbare|chr10:8494987..8504235|Similar to Flavonoid 3-monooxygenase.
Alongig7G01297
Bdistac XP 003562884.2 flavonoid 3-monooxygenase CYP75B4 Brachypodium distachyon
Bdistac XP 003577475.1 flavonoid 3-monooxygenase CYP75B4 Brachypodium distachyon
Athalia5G07990.1 | Cytochrome P450 superfamily protein | Chr5:2560437-2562859 FORWARD LENGTH=513 | 201606
Zoffici042377047.1 zingiber officinale|4A:103408629..103410687|LOC121970404|XM 042521113.1|zingiber officinale042377047.1
Zoffici042458020.1 zingiber officinale|2A:121586208..121588131|LOC122042133|XM 042602086.1|zingiber officinale042458020.1
Mbeccar06 t019910.1
Mlasioc1h03G02252.1
Eglaucu03 t018110
Mbalbis01 g02030.1
Macumin4 03 g33170.1 assembled CDS
Mschizo03t075990.1 Flavonoid 3-monooxygenase CYP75B3 OS=Oryza sativa subsp. japonica OX=39947 GN=CYP75B3 PE=2 SV=1
H FLS
Mlasioc1h09G00678.1
Eglaucu01 t007540
Mbeccar03 t008100.1
Macumin4 03 g07220.1 assembled CDS
Mschizo03t052640.1 Flavonol synthase/flavanone 3-hydroxylase OS=Citrus unshiu OX=55188 GN=FLS PE=1 SV=1
Mbalbis03 g06940.1
Mlasioc1h06G02043.1
Eglaucu06 t006150
Mbeccar08 t036240.1
Mbalbis09 g14060.1
Zoffici042378807.1 zingiber officinale|4A:143603082..143605459|LOC121971547|XM 042522873.1|zingiber officinale042378807.1
Zoffici042406407.1 zingiber officinale|6A:67847610..67862317|LOC121996484|XM 042550473.1|zingiber officinale042406407.1
Zoffici042406408.1 zingiber officinale|6A:67907616..67909668|LOC121996485|XM 042550474.1|zingiber officinale042406408.1
Mbalbis10 g22000.1
Mschizo10t292840.1 Flavonol synthase/flavanone 3-hydroxylase OS=Citrus unshiu OX=55188 GN=FLS PE=1 SV=1
Mlasioc1h05G00635.1
Mlasioc1h05G00636.1
Eglaucu08 t007020
Mbeccar02 t047300.2
Mbalbis10 g21980.1
Mlasioc1h03G01030.1
Eglaucu03 t031640
Mbeccar08 t015900.1
Mbalbis08 g23630.1
Macumin4 08 g24690.1 assembled CDS
Mschizo08t229420.1 Flavonol synthase/flavanone 3-hydroxylase OS=Citrus unshiu OX=55188 GN=FLS PE=1 SV=1
Athalia5G08640.1 | flavonol synthase 1 | Chr5:2804009-2805175 FORWARD LENGTH=336 | 201606
Athalia5G63590.1 | flavonol synthase 3 | Chr5:25457172-25458427 REVERSE LENGTH=308 | 201606
Athalia5G43935.1 | flavonol synthase 6 | Chr5:17682179-17683446 FORWARD LENGTH=293 | 201606
Athalia5G63595.1 | flavonol synthase 4 | Chr5:25459256-25460436 REVERSE LENGTH=279 | 201606
Athalia5G63580.1 | flavonol synthase 2 | Chr5:25454730-25456092 FORWARD LENGTH=250 | 201606
Athalia5G63600.1 | flavonol synthase 5 | Chr5:25461082-25462270 REVERSE LENGTH=325 | 201606
Bdistac XP 003570562.1 flavonol synthase/flavanone 3-hydroxylase Brachypodium distachyon
Sobicol004G310100.2.p sorghum bicolor BTx623 v3.1
Sobicol003G021700.1.p sorghum bicolor BTx623 v3.1
Alongig7G03380
Alongig6G04177
Alongig6G04187
Zoffici042421823.1 zingiber officinale|8A:77963115..77965071|LOC122009643|XM 042565889.1|zingiber officinale042421823.1

I F3'5'H

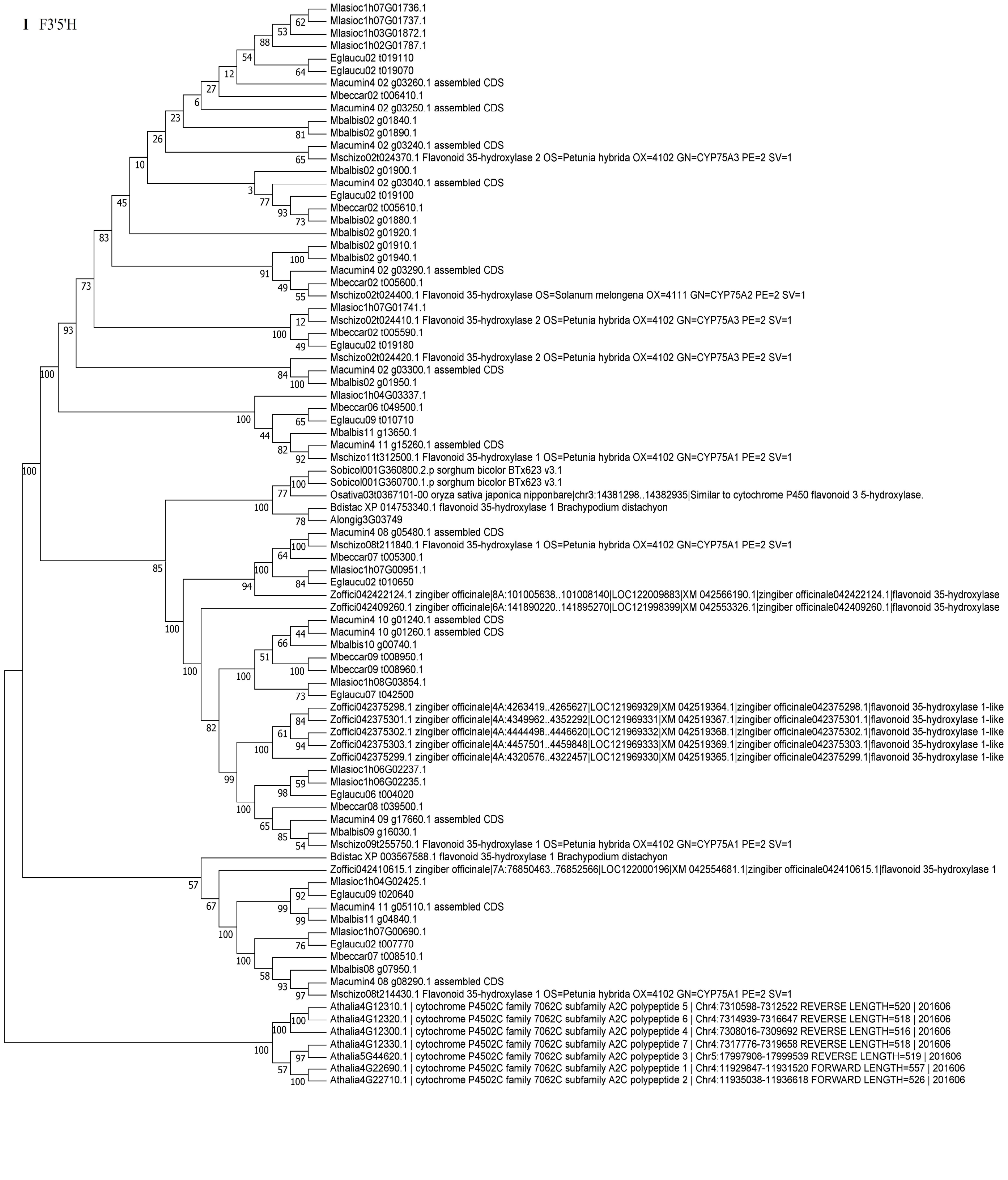
Mlasioc1h07G01736.1
Mlasioc1h07G01737.1
Mlasioc1h03G01872.1
Mlasioc1h02G01787.1
Eglaucu02 t019110
Eglaucu02 t019070
Macumin4 02 g03260.1 assembled CDS
Mbeccar02 t006410.1
Macumin4 02 g03250.1 assembled CDS
Mbalbis02 g01840.1
Mbalbis02 g01890.1
Macumin4 02 g03240.1 assembled CDS
Mschizo02t024370.1 Flavonoid 35-hydroxylase 2 OS=Petunia hybrida OX=4102 GN=CYP75A3 PE=2 SV=1
Mbalbis02 g01900.1
Macumin4 02 g03040.1 assembled CDS
Eglaucu02 t019100
Mbeccar02 t005610.1
Mbalbis02 g01880.1
Mbalbis02 g01920.1
Mbalbis02 g01910.1
Mbalbis02 g01940.1
Macumin4 02 g03290.1 assembled CDS
Mbeccar02 t005600.1
Mschizo02t024400.1 Flavonoid 35-hydroxylase OS=Solanum melongena OX=4111 GN=CYP75A2 PE=2 SV=1
Mlasioc1h07G01741.1
Mschizo02t024410.1 Flavonoid 35-hydroxylase 2 OS=Petunia hybrida OX=4102 GN=CYP75A3 PE=2 SV=1
Mbeccar02 t005590.1
Eglaucu02 t019180
Mschizo02t024420.1 Flavonoid 35-hydroxylase 2 OS=Petunia hybrida OX=4102 GN=CYP75A3 PE=2 SV=1
Macumin4 02 g03300.1 assembled CDS
Mbalbis02 g01950.1
Mlasioc1h04G03337.1
Mbeccar06 t049500.1
Eglaucu09 t010710
Mbalbis11 g13650.1
Macumin4 11 g15260.1 assembled CDS
Mschizo11t312500.1 Flavonoid 35-hydroxylase 1 OS=Petunia hybrida OX=4102 GN=CYP75A1 PE=2 SV=1
Sobicol001G360800.2.p sorghum bicolor BTx623 v3.1
Sobicol001G360700.1.p sorghum bicolor BTx623 v3.1
Osativa03t0367101-00 oryza sativa japonica nipponbare|chr3:14381298..14382935|Similar to cytochrome P450 flavonoid 3 5-hydroxylase.
Bdistac XP 014753340.1 flavonoid 35-hydroxylase 1 Brachypodium distachyon
Alongig3G03749
Macumin4 08 g05480.1 assembled CDS
Mschizo08t211840.1 Flavonoid 35-hydroxylase 1 OS=Petunia hybrida OX=4102 GN=CYP75A1 PE=2 SV=1
Mbeccar07 t005300.1
Mlasioc1h07G00951.1
Eglaucu02 t010650
Zoffici042422124.1 zingiber officinale|8A:101005638..101008140|LOC122009883|XM 042566190.1|zingiber officinale042422124.1|flavonoid 35-hydroxylase
Zoffici042409260.1 zingiber officinale|6A:141890220..141895270|LOC121998399|XM 042553326.1|zingiber officinale042409260.1|flavonoid 35-hydroxylase
Macumin4 10 g01240.1 assembled CDS
Macumin4 10 g01260.1 assembled CDS
Mbalbis10 g00740.1
Mbeccar09 t008950.1
Mbeccar09 t008960.1
Mlasioc1h08G03854.1
Eglaucu07 t042500
Zoffici042375298.1 zingiber officinale|4A:4263419..4265627|LOC121969329|XM 042519364.1|zingiber officinale042375298.1|flavonoid 35-hydroxylase 1-like
Zoffici042375301.1 zingiber officinale|4A:4349962..4352292|LOC121969331|XM 042519367.1|zingiber officinale042375301.1|flavonoid 35-hydroxylase 1-like
Zoffici042375302.1 zingiber officinale|4A:4444498..4446620|LOC121969332|XM 042519368.1|zingiber officinale042375302.1|flavonoid 35-hydroxylase 1-like
Zoffici042375303.1 zingiber officinale|4A:4457501..4459848|LOC121969333|XM 042519369.1|zingiber officinale042375303.1|flavonoid 35-hydroxylase 1-like
Zoffici042375299.1 zingiber officinale|4A:4320576..4322457|LOC121969330|XM 042519365.1|zingiber officinale042375299.1|flavonoid 35-hydroxylase 1-like
Mlasioc1h06G02237.1
Mlasioc1h06G02235.1
Eglaucu06 t004020
Mbeccar08 t039500.1
Macumin4 09 g17660.1 assembled CDS
Mbalbis09 g16030.1
Mschizo09t255750.1 Flavonoid 35-hydroxylase 1 OS=Petunia hybrida OX=4102 GN=CYP75A1 PE=2 SV=1
Bdistac XP 003567588.1 flavonoid 35-hydroxylase 1 Brachypodium distachyon
Zoffici042410615.1 zingiber officinale|7A:76850463..76852566|LOC122000196|XM 042554681.1|zingiber officinale042410615.1|flavonoid 35-hydroxylase 1
Mlasioc1h04G02425.1
Eglaucu09 t020640
Macumin4 11 g05110.1 assembled CDS
Mbalbis11 g04840.1
Mlasioc1h07G00690.1
Eglaucu02 t007770
Mbeccar07 t008510.1
Mbalbis08 g07950.1
Macumin4 08 g08290.1 assembled CDS
Mschizo08t214430.1 Flavonoid 35-hydroxylase 1 OS=Petunia hybrida OX=4102 GN=CYP75A1 PE=2 SV=1
Athalia4G12310.1 | cytochrome P4502C family 7062C subfamily A2C polypeptide 5 | Chr4:7310598-7312522 REVERSE LENGTH=520 | 201606
Athalia4G12320.1 | cytochrome P4502C family 7062C subfamily A2C polypeptide 6 | Chr4:7314939-7316647 REVERSE LENGTH=518 | 201606
Athalia4G12300.1 | cytochrome P4502C family 7062C subfamily A2C polypeptide 4 | Chr4:7308016-7309692 REVERSE LENGTH=516 | 201606
Athalia4G12330.1 | cytochrome P4502C family 7062C subfamily A2C polypeptide 7 | Chr4:7317776-7319658 REVERSE LENGTH=518 | 201606
Athalia5G44620.1 | cytochrome P4502C family 7062C subfamily A2C polypeptide 3 | Chr5:17997908-17999539 REVERSE LENGTH=519 | 201606
Athalia4G22690.1 | cytochrome P4502C family 7062C subfamily A2C polypeptide 1 | Chr4:11929847-11931520 FORWARD LENGTH=557 | 201606
Athalia4G22710.1 | cytochrome P4502C family 7062C subfamily A2C polypeptide 2 | Chr4:11935038-11936618 FORWARD LENGTH=526 | 201606

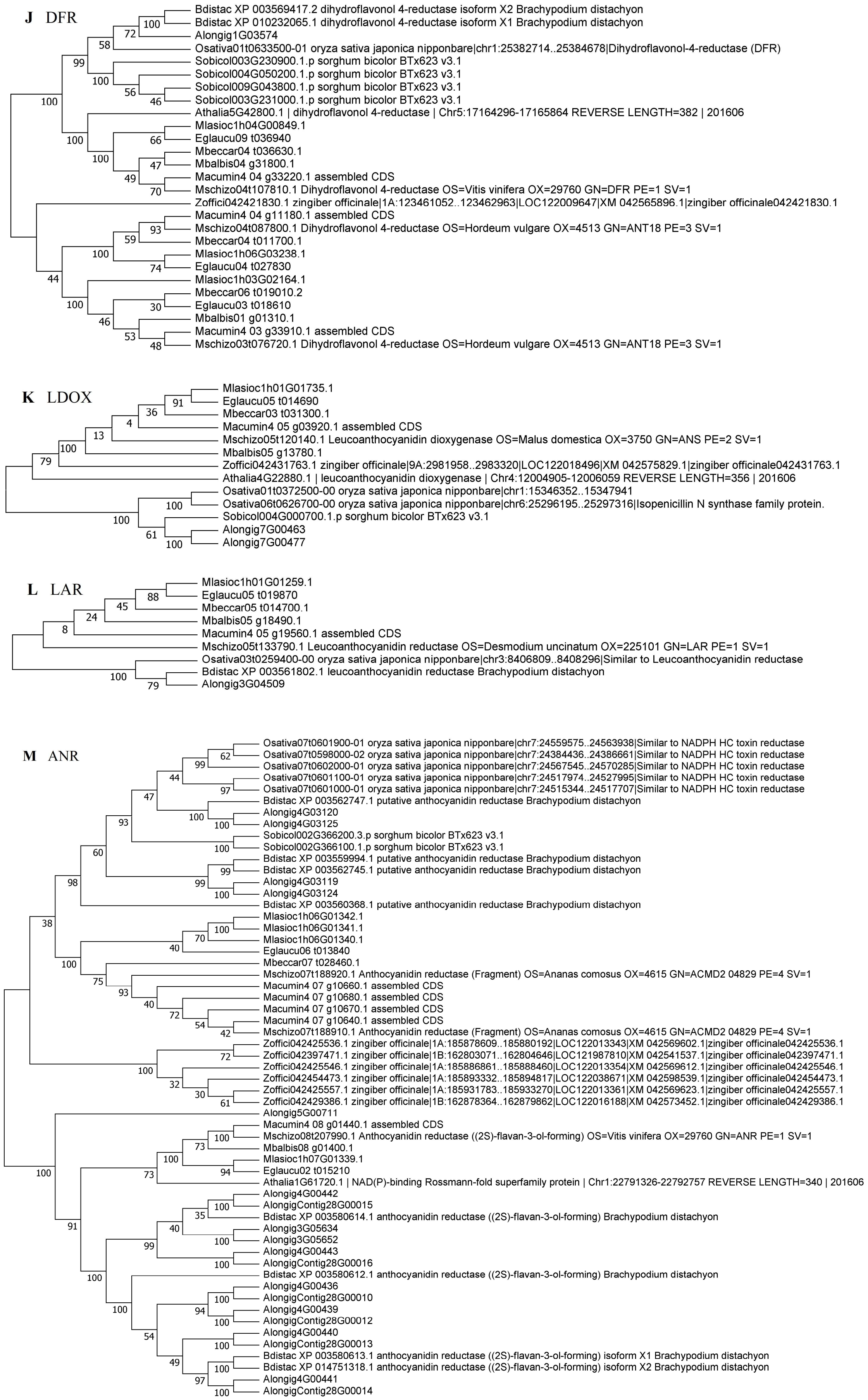
J DFR
Bdistac XP 003569417.2 dihydroflavonol 4-reductase isoform X2 Brachypodium distachyon
Bdistac XP 010232065.1 dihydroflavonol 4-reductase isoform X1 Brachypodium distachyon
Alongig1G03574
Osativa01t0633500-01 oryza sativa japonica nipponbare|chr1:25382714..25384678|Dihydroflavonol-4-reductase (DFR)
Sobicol003G230900.1.p sorghum bicolor BTx623 v3.1
Sobicol004G050200.1.p sorghum bicolor BTx623 v3.1
Sobicol009G043800.1.p sorghum bicolor BTx623 v3.1
Sobicol003G231000.1.p sorghum bicolor BTx623 v3.1
Athalia5G42800.1 | dihydroflavonol 4-reductase | Chr5:17164296-17165864 REVERSE LENGTH=382 | 201606
Mlasioc1h04G00849.1
Eglaucu09 t036940
Mbeccar04 t036630.1
Mbalbis04 g31800.1
Macumin4 04 g33220.1 assembled CDS
Mschizo04t107810.1 Dihydroflavonol 4-reductase OS=Vitis vinifera OX=29760 GN=DFR PE=1 SV=1
Zoffici042421830.1 zingiber officinale|1A:123461052..123462963|LOC122009647|XM 042565896.1|zingiber officinale042421830.1
Macumin4 04 g11180.1 assembled CDS
Mschizo04t087800.1 Dihydroflavonol 4-reductase OS=Hordeum vulgare OX=4513 GN=ANT18 PE=3 SV=1
Mbeccar04 t011700.1
Mlasioc1h06G03238.1
Eglaucu04 t027830
Mlasioc1h03G02164.1
Mbeccar06 t019010.2
Eglaucu03 t018610
Mbalbis01 g01310.1
Macumin4 03 g33910.1 assembled CDS
Mschizo03t076720.1 Dihydroflavonol 4-reductase OS=Hordeum vulgare OX=4513 GN=ANT18 PE=3 SV=1
K LDOX
Mlasioc1h01G01735.1
Eglaucu05 t014690
Mbeccar03 t031300.1
Macumin4 05 g03920.1 assembled CDS
Mschizo05t120140.1 Leucoanthocyanidin dioxygenase OS=Malus domestica OX=3750 GN=ANS PE=2 SV=1
Mbalbis05 g13780.1
Zoffici042431763.1 zingiber officinale|9A:2981958..2983320|LOC122018496|XM 042575829.1|zingiber officinale042431763.1
Athalia4G22880.1 | leucoanthocyanidin dioxygenase | Chr4:12004905-12006059 REVERSE LENGTH=356 | 201606
Osativa01t0372500-00 oryza sativa japonica nipponbare|chr1:15346352..15347941
Osativa06t0626700-00 oryza sativa japonica nipponbare|chr6:25296195..25297316|Isopenicillin N synthase family protein.
Sobicol004G000700.1.p sorghum bicolor BTx623 v3.1
Alongig7G00463
Alongig7G00477
L LAR
Mlasioc1h01G01259.1
Eglaucu05 t019870
Mbeccar05 t014700.1
Mbalbis05 g18490.1
Macumin4 05 g19560.1 assembled CDS
Mschizo05t133790.1 Leucoanthocyanidin reductase OS=Desmodium uncinatum OX=225101 GN=LAR PE=1 SV=1
Osativa03t0259400-00 oryza sativa japonica nipponbare|chr3:8406809..8408296|Similar to Leucoanthocyanidin reductase
Bdistac XP 003561802.1 leucoanthocyanidin reductase Brachypodium distachyon
Alongig3G04509
M ANR
Osativa07t0601900-01 oryza sativa japonica nipponbare|chr7:24559575..24563938|Similar to NADPH HC toxin reductase
Osativa07t0598000-02 oryza sativa japonica nipponbare|chr7:24384436..24386661|Similar to NADPH HC toxin reductase
Osativa07t0602000-01 oryza sativa japonica nipponbare|chr7:24567545..24570285|Similar to NADPH HC toxin reductase
Osativa07t0601100-01 oryza sativa japonica nipponbare|chr7:24517974..24527995|Similar to NADPH HC toxin reductase
Osativa07t0601000-01 oryza sativa japonica nipponbare|chr7:24515344..24517707|Similar to NADPH HC toxin reductase
Bdistac XP 003562747.1 putative anthocyanidin reductase Brachypodium distachyon
Alongig4G03120
Alongig4G03125
Sobicol002G366200.3.p sorghum bicolor BTx623 v3.1
Sobicol002G366100.1.p sorghum bicolor BTx623 v3.1
Bdistac XP 003559994.1 putative anthocyanidin reductase Brachypodium distachyon
Bdistac XP 003562745.1 putative anthocyanidin reductase Brachypodium distachyon
Alongig4G03119
Alongig4G03124
Bdistac XP 003560368.1 putative anthocyanidin reductase Brachypodium distachyon
Mlasioc1h06G01342.1
Mlasioc1h06G01341.1
Mlasioc1h06G01340.1
Eglaucu06 t013840
Mbeccar07 t028460.1
Mschizo07t188920.1 Anthocyanidin reductase (Fragment) OS=Ananas comosus OX=4615 GN=ACMD2 04829 PE=4 SV=1
Macumin4 07 g10660.1 assembled CDS
Macumin4 07 g10680.1 assembled CDS
Macumin4 07 g10670.1 assembled CDS
Macumin4 07 g10640.1 assembled CDS
Mschizo07t188910.1 Anthocyanidin reductase (Fragment) OS=Ananas comosus OX=4615 GN=ACMD2 04829 PE=4 SV=1
Zoffici042425536.1 zingiber officinale|1A:185878609..185880192|LOC122013343|XM 042569602.1|zingiber officinale042425536.1
Zoffici042397471.1 zingiber officinale|1B:162803071..162804646|LOC121987810|XM 042541537.1|zingiber officinale042397471.1
Zoffici042425546.1 zingiber officinale|1A:185886861..185888460|LOC122013354|XM 042569612.1|zingiber officinale042425546.1
Zoffici042454473.1 zingiber officinale|1A:185893332..185894817|LOC122038671|XM 042598539.1|zingiber officinale042454473.1
Zoffici042425557.1 zingiber officinale|1A:185931783..185933270|LOC122013361|XM 042569623.1|zingiber officinale042425557.1
Zoffici042429386.1 zingiber officinale|1B:162878364..162879862|LOC122016188|XM 042573452.1|zingiber officinale042429386.1
Alongig5G00711
Macumin4 08 g01440.1 assembled CDS
Mschizo08t207990.1 Anthocyanidin reductase ((2S)-flavan-3-ol-forming) OS=Vitis vinifera OX=29760 GN=ANR PE=1 SV=1
Mbalbis08 g01400.1
Mlasioc1h07G01339.1
Eglaucu02 t015210
Athalia1G61720.1 | NAD(P)-binding Rossmann-fold superfamily protein | Chr1:22791326-22792757 REVERSE LENGTH=340 | 201606
Alongig4G00442
AlongigContig28G00015
Bdistac XP 003580614.1 anthocyanidin reductase ((2S)-flavan-3-ol-forming) Brachypodium distachyon
Alongig3G05634
Alongig3G05652
Alongig4G00443
AlongigContig28G00016
Bdistac XP 003580612.1 anthocyanidin reductase ((2S)-flavan-3-ol-forming) Brachypodium distachyon
Alongig4G00436
AlongigContig28G00010
Alongig4G00439
AlongigContig28G00012
Alongig4G00440
AlongigContig28G00013
Bdistac XP 003580613.1 anthocyanidin reductase ((2S)-flavan-3-ol-forming) isoform X1 Brachypodium distachyon
Bdistac XP 014751318.1 anthocyanidin reductase ((2S)-flavan-3-ol-forming) isoform X2 Brachypodium distachyon
Alongig4G00441
AlongigContig28G00014

N UGT

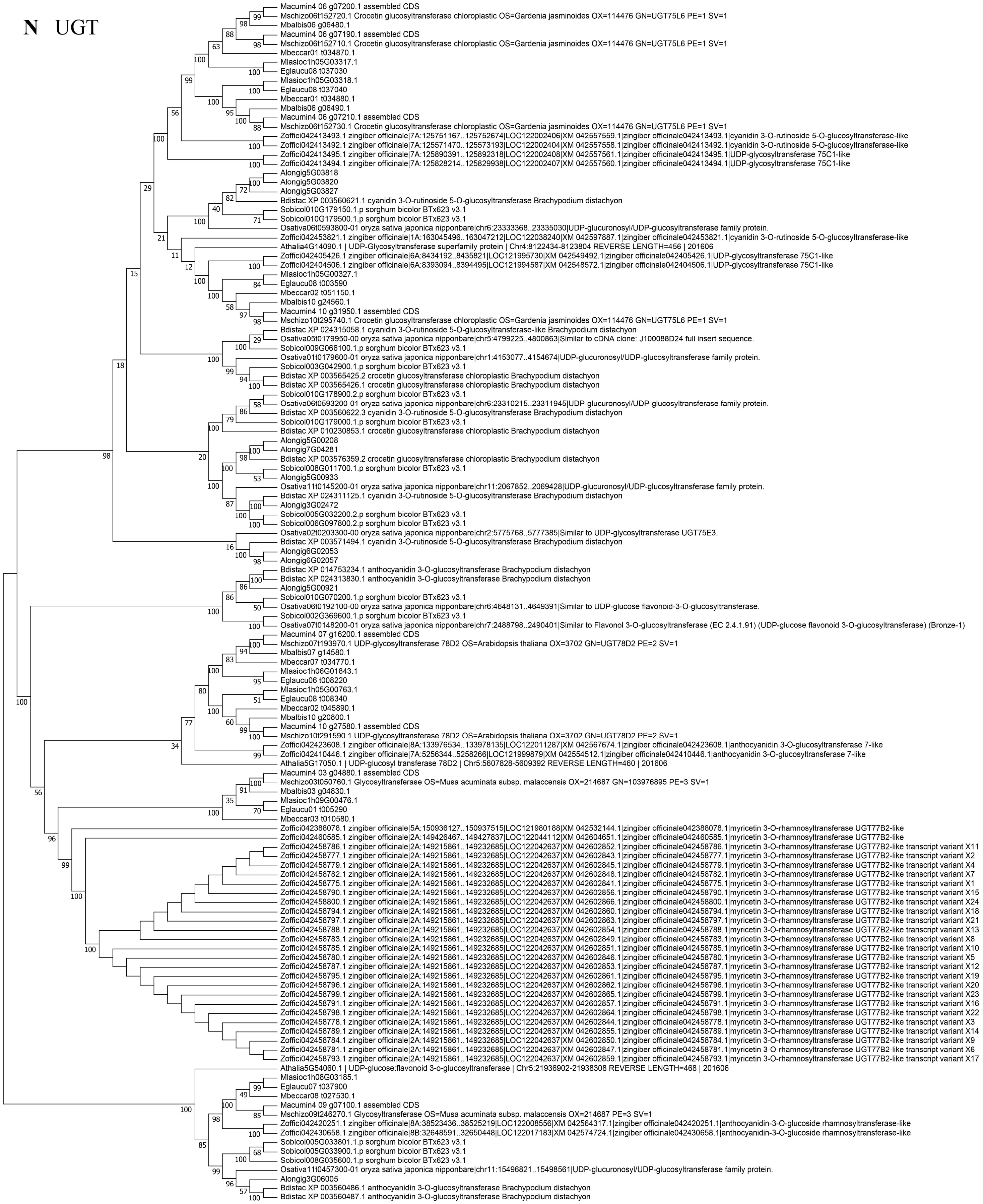

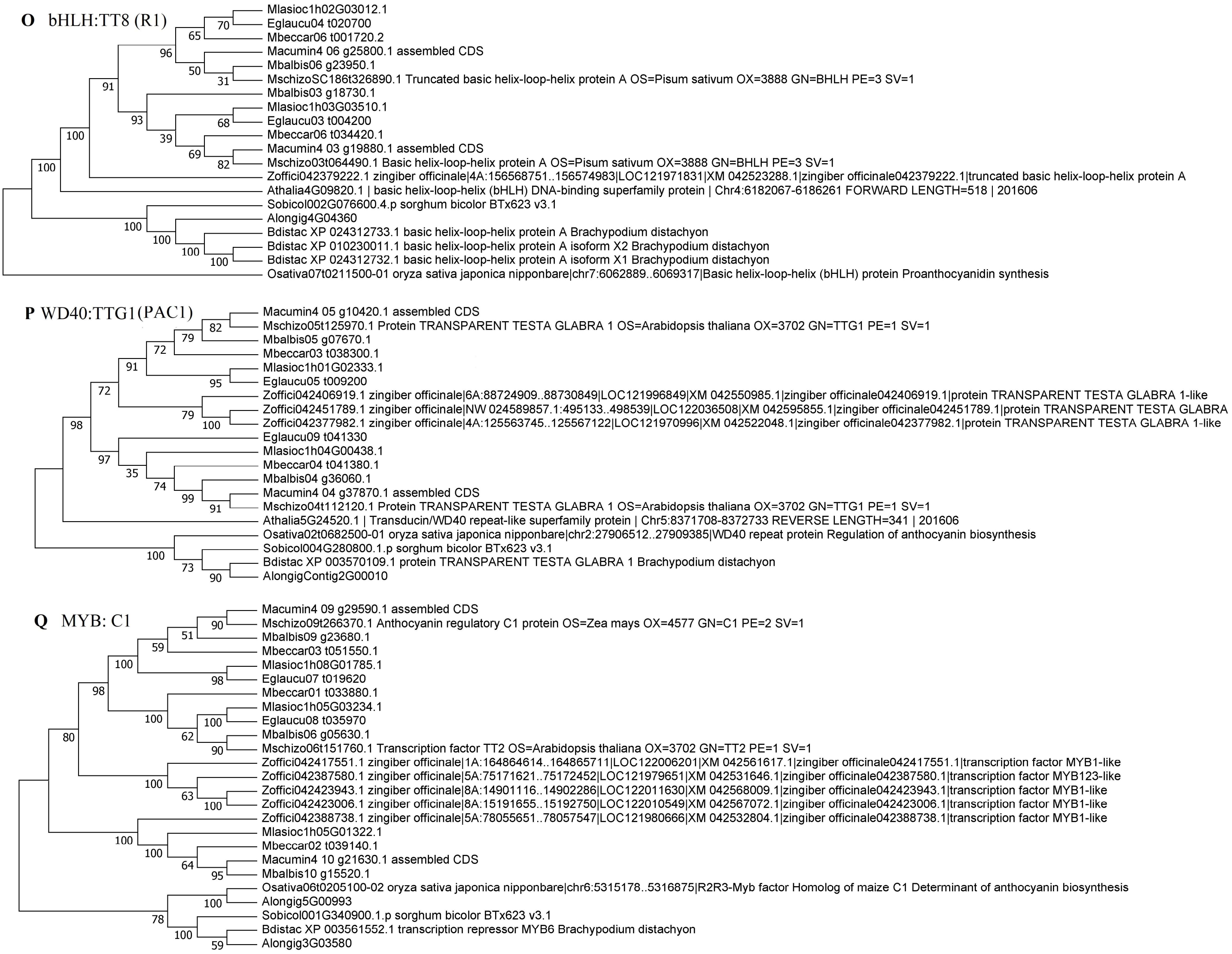

Figure S2 Phylogenetic trees shows the genetic relationship of flavonoid biosynthetic genes in *Arabidopsis*, *B. distachyon*, *A. longiglumis*, *O. sativa*, *S. bicolor*, *Z. officinale*, *E. glaucum*, *M. lasiocarpa*, *M. beccarii*, *M. acuminata*, *M. balbisiana*, and *M. schizocarpa.* (**A**) PAL family. (**B**) 4CL family. (**C**) C4H family. (**D**) CHI family. (**E**) CHS family. (**F**) F3H family. (**G**) F3'H family. (**H**) FLS family. (**I**) F3'5'H family. (**J**) DFR family. (**K**) LDOX family. (**L**) LAR family. (**M**) ANR family. (**N**) UGT family. (**O**) bHLH: TT8 (R1) family. (**P**) WD40: TTG1 (PAC1) family. (**Q**) MYB: C1 family.

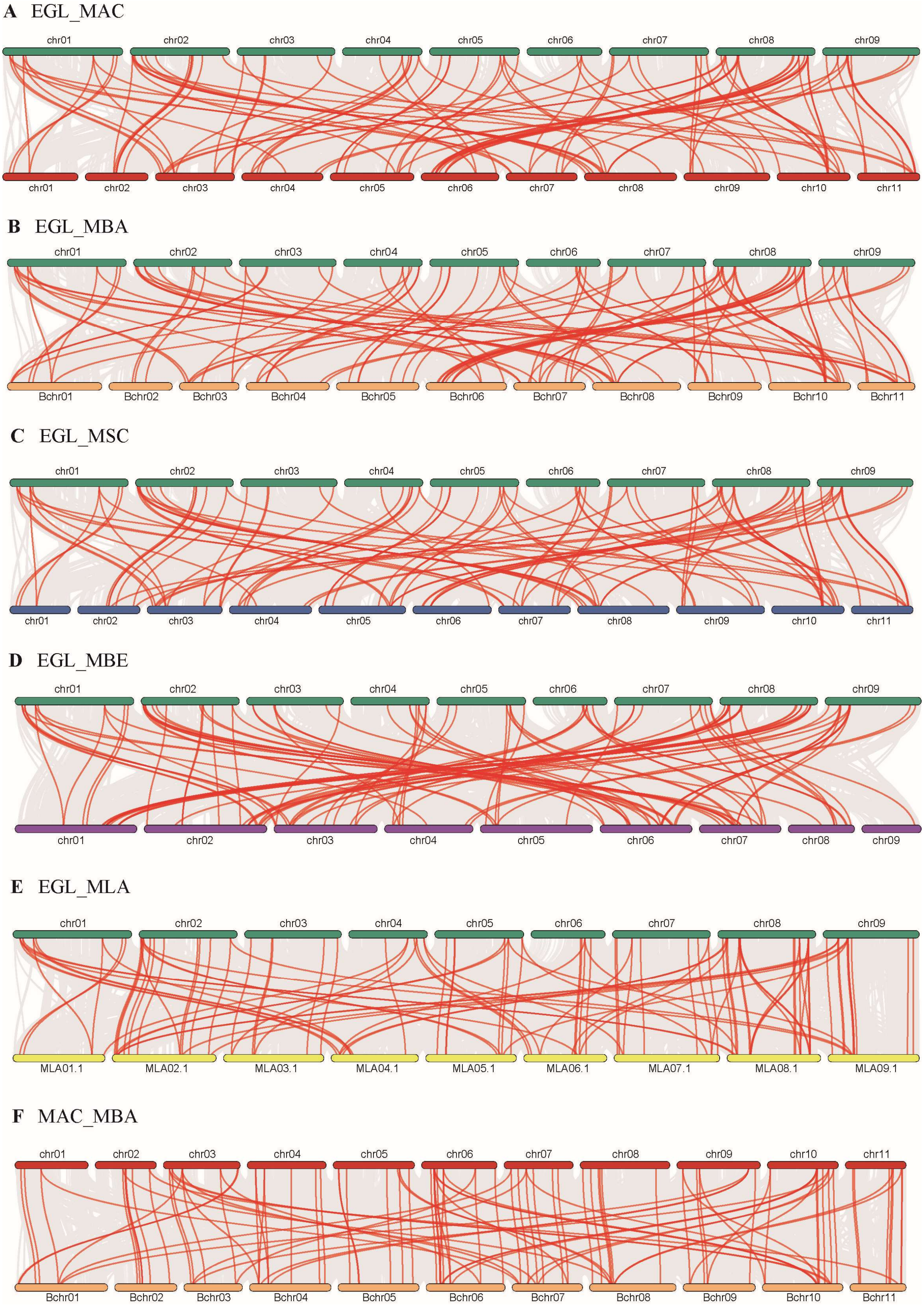

A EGL_MAC
chr01 chr02 chr03 chr04 chr05 chr06 chr07 chr08 chr09
chr01 chr02 chr03 chr04 chr05 chr06 chr07 chr08 chr09 chr10 chr11
B EGL_MBA
chr01 chr02 chr03 chr04 chr05 chr06 chr07 chr08 chr09
Bchr01 Bchr02 Bchr03 Bchr04 Bchr05 Bchr06 Bchr07 Bchr08 Bchr09 Bchr10 Bchr11
C EGL_MSC
chr01 chr02 chr03 chr04 chr05 chr06 chr07 chr08 chr09
chr01 chr02 chr03 chr04 chr05 chr06 chr07 chr08 chr09 chr10 chr11
D EGL_MBE
chr01 chr02 chr03 chr04 chr05 chr06 chr07 chr08 chr09
chr01 chr02 chr03 chr04 chr05 chr06 chr07 chr08 chr09
E EGL_MLA
chr01 chr02 chr03 chr04 chr05 chr06 chr07 chr08 chr09
MLA01.1 MLA02.1 MLA03.1 MLA04.1 MLA05.1 MLA06.1 MLA07.1 MLA08.1 MLA09.1
F MAC_MBA
chr01 chr02 chr03 chr04 chr05 chr06 chr07 chr08 chr09 chr10 chr11
Bchr01 Bchr02 Bchr03 Bchr04 Bchr05 Bchr06 Bchr07 Bchr08 Bchr09 Bchr10 Bchr11

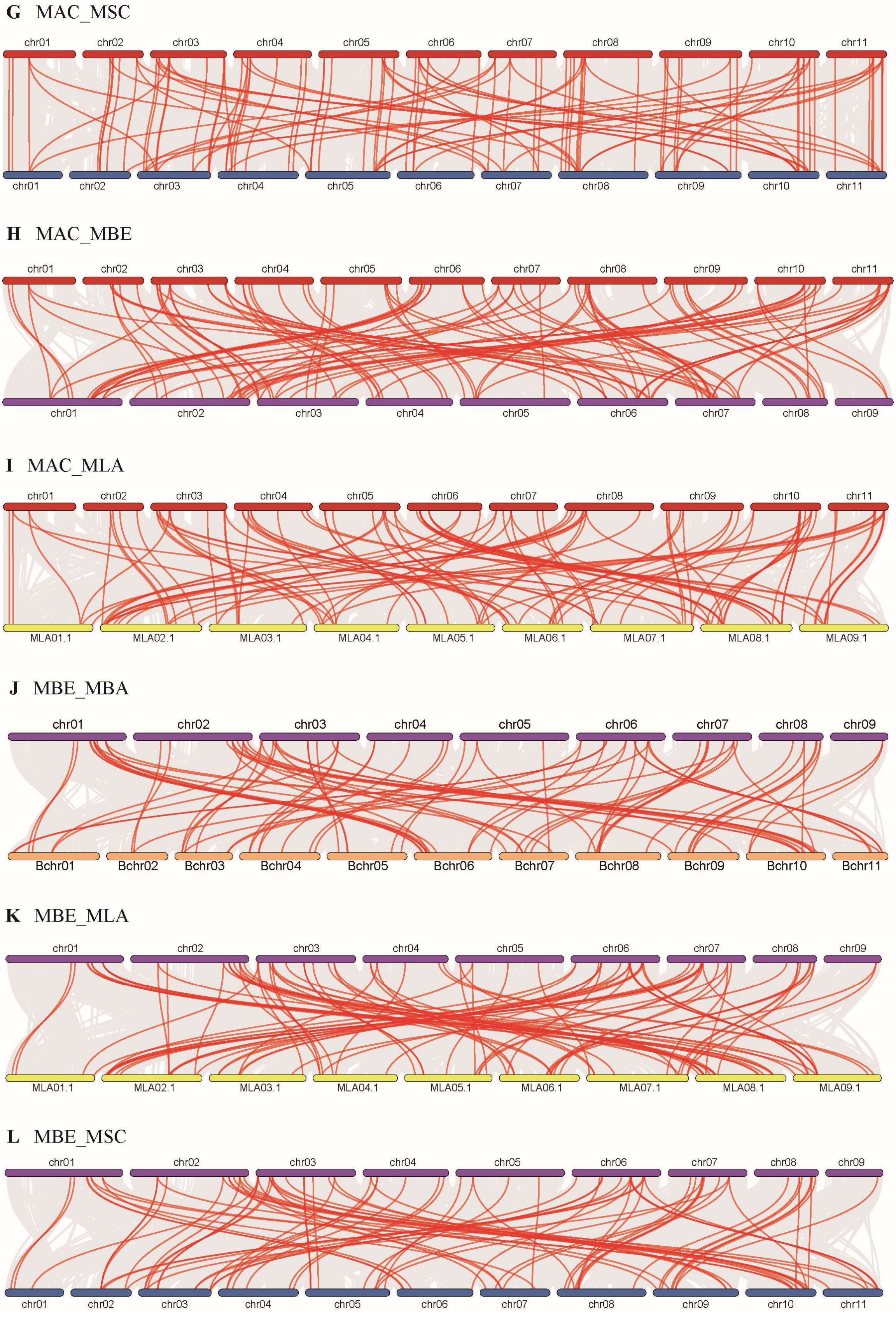
G MAC_MSC
chr01 chr02 chr03 chr04 chr05 chr06 chr07 chr08 chr09 chr10 chr11
chr01 chr02 chr03 chr04 chr05 chr06 chr07 chr08 chr09 chr10 chr11
H MAC_MBE
chr01 chr02 chr03 chr04 chr05 chr06 chr07 chr08 chr09 chr10 chr11
chr01 chr02 chr03 chr04 chr05 chr06 chr07 chr08 chr09
I MAC_MLA
chr01 chr02 chr03 chr04 chr05 chr06 chr07 chr08 chr09 chr10 chr11
MLA01.1 MLA02.1 MLA03.1 MLA04.1 MLA05.1 MLA06.1 MLA07.1 MLA08.1 MLA09.1
J MBE_MBA
chr01 chr02 chr03 chr04 chr05 chr06 chr07 chr08 chr09
Bchr01 Bchr02 Bchr03 Bchr04 Bchr05 Bchr06 Bchr07 Bchr08 Bchr09 Bchr10 Bchr11
K MBE_MLA
chr01 chr02 chr03 chr04 chr05 chr06 chr07 chr08 chr09
MLA01.1 MLA02.1 MLA03.1 MLA04.1 MLA05.1 MLA06.1 MLA07.1 MLA08.1 MLA09.1
L MBE_MSC
chr01 chr02 chr03 chr04 chr05 chr06 chr07 chr08 chr09
chr01 chr02 chr03 chr04 chr05 chr06 chr07 chr08 chr09 chr10 chr11

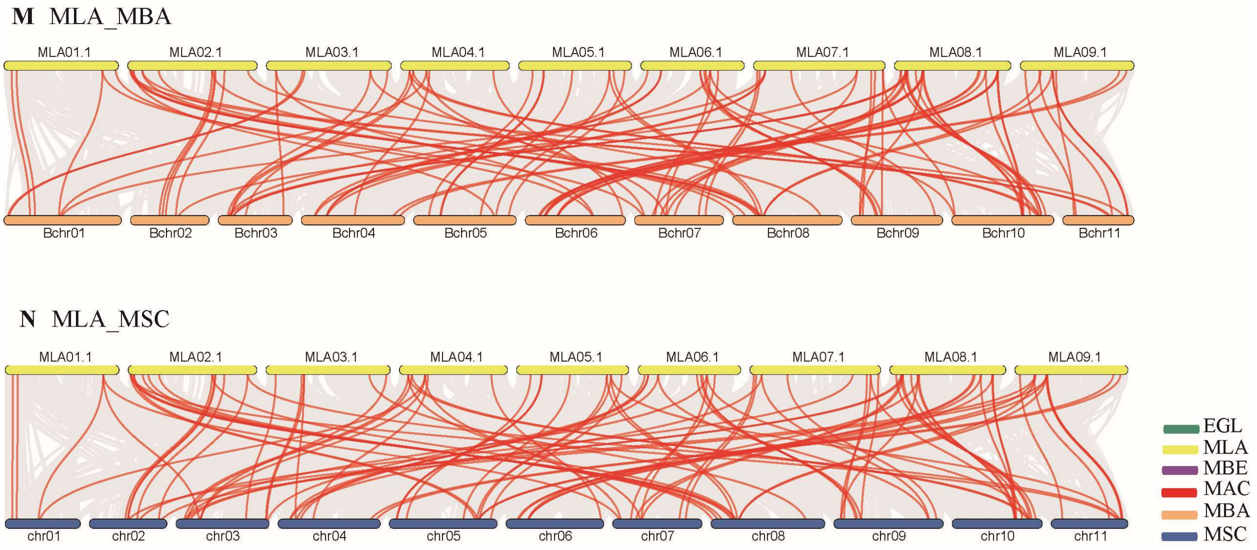

Figure S3. Synteny analysis of FBGs between six Musaceae species. Gray lines in the background indicate the collinear blocks within Musaceae genomes, while the red lines highlight the syntenic FBG pairs. The specie names "EGL", "MLA", "MBE", "MAC", "MBA", and "MSC" indicate *E. glaucum*, *M. lasiocarpa*, *M. beccarii, M. acuminata*, *M. balbisiana*, and *M. schizocarpa*. (**A**) *E. glaucum* with *M. acuminata*. (**B**) *E. glaucum* with *M. balbisiana*. (**C**) *E. glaucum* with *M. schizocarpa*. (**D**) *E. glaucum* with *M. beccarii*. (**E**) *E. glaucum* with *M. lasiocarpa*. (**F**) *M. acuminata* with *M. balbisiana*. (**G**) *M. acuminata* with *M. schizocarpa*. (**H**) *M. acuminata* with *M. beccarii.* (**I**) *M. acuminata.* with *M. lasiocarpa* (**J**) *M. beccarii* with *M. balbisiana* (**K**) *M. beccarii* with *M. lasiocarpa.* (**L**) *M. beccarii* with *M. schizocarpa* (**M**) *M. lasiocarpa* with *M. balbisiana* (**N**) *M. lasiocarpa* with *M. schizocarp*.

A

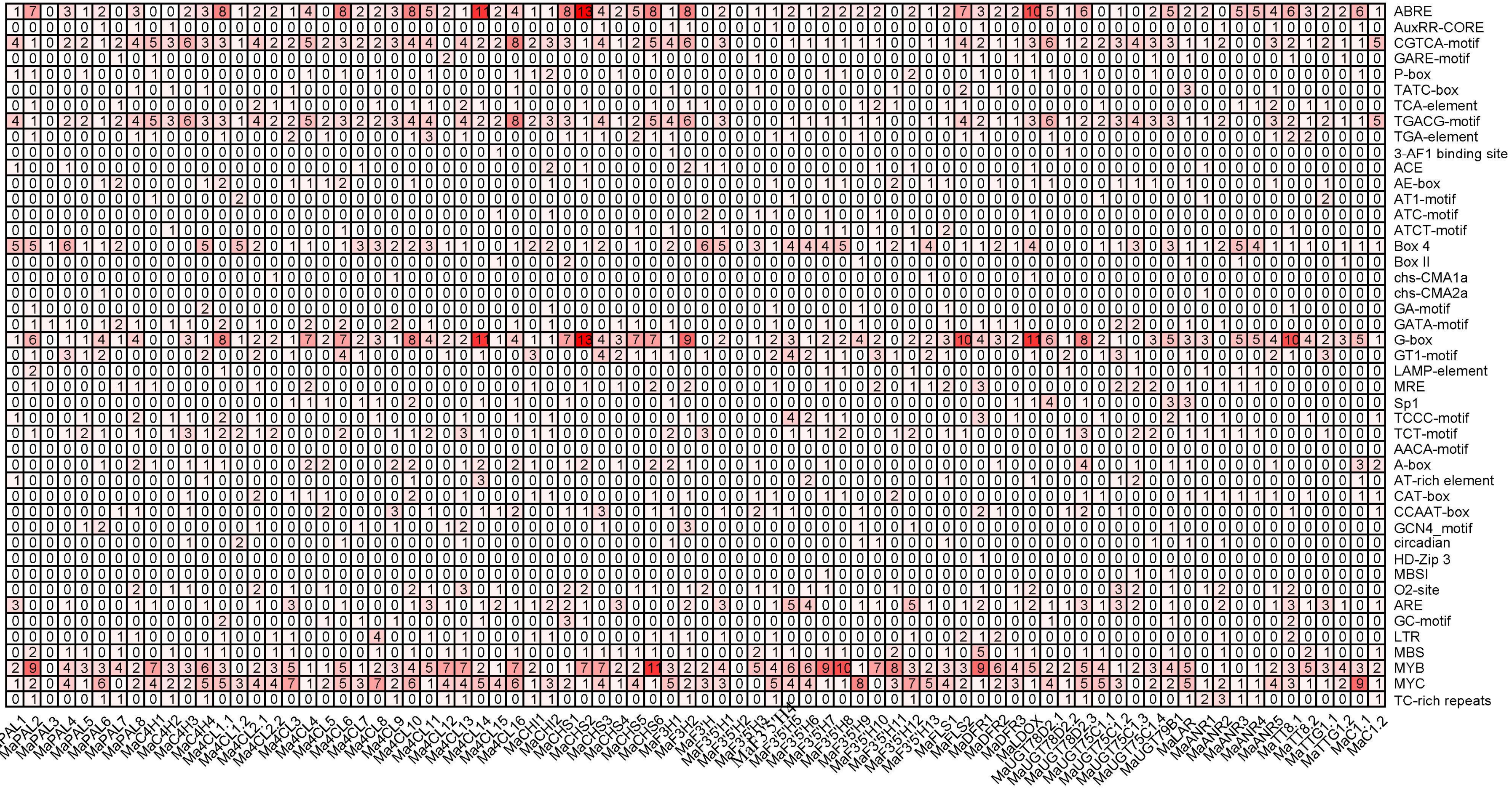

Cui *et al*. Flavonoid biosynthetic genes in Musaceae. AoB Plants 2024.

B

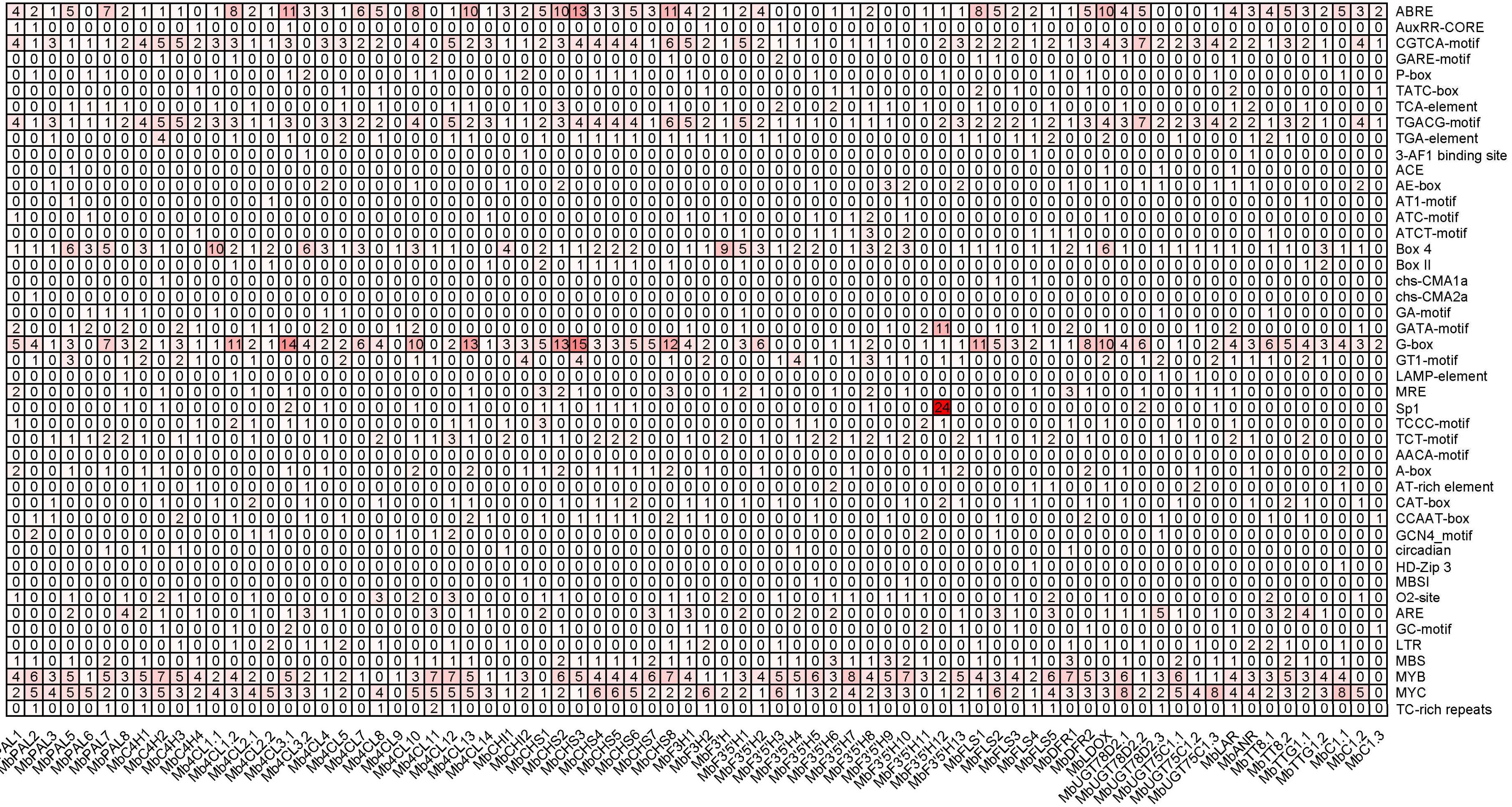
ABRE
AuxRR-CORE
CGTCA-motif
GARE-motif
P-box
TATC-box
TCA-element
TGACG-motif
TGA-element
3-AF1 binding site
ACE
AE-box
AT1-motif
ATC-motif
ATCT-motif
Box 4
Box II
chs-CMA1a
chs-CMA2a
GA-motif
GATA-motif
G-box
GT1-motif
LAMP-element
MRE
Sp1
TCCC-motif
TCT-motif
AACA-motif
A-box
AT-rich element
CAT-box
CCAAT-box
GCN4_motif
circadian
HD-Zip 3
MBSI
O2-site
ARE
GC-motif
LTR
MBS
MYB
MYC
TC-rich repeats
MbPAL1
MbPAL2
MbPAL3
MbPAL5
MbPAL6
MbPAL7
MbPAL8
MbC4H1
MbC4H2
MbC4H3
MbC4H4
Mb4CL1.1
Mb4CL1.2
Mb4CL2.1
Mb4CL2.2
Mb4CL3.1
Mb4CL3.2
Mb4CL4
Mb4CL5
Mb4CL7
Mb4CL8
Mb4CL9
Mb4CL10
Mb4CL11
Mb4CL12
Mb4CL13
Mb4CL14
MbCHI1
MbCHI2
MbCHS1
MbCHS2
MbCHS3
MbCHS4
MbCHS5
MbCHS6
MbCHS7
MbCHS8
MbF3H1
MbF3H2
MbF3'H
MbF3'5'H1
MbF3'5'H2
MbF3'5'H3
MbF3'5'H4
MbF3'5'H5
MbF3'5'H6
MbF3'5'H7
MbF3'5'H8
MbF3'5'H9
MbF3'5'H10
MbF3'5'H11
MbF3'5'H12
MbF3'5'H13
MbFLS1
MbFLS2
MbFLS3
MbFLS4
MbFLS5
MbDFR1
MbDFR2
MbLDOX
MbUGT78D2.1
MbUGT78D2.2
MbUGT78D2.3
MbUGT75C1.1
MbUGT75C1.2
MbUGT75C1.3
MbLAR
MbANR
MbTT8.1
MbTT8.2
MbTTG1.1
MbTTG1.2
MbC1.1
MbC1.2
MbC1.3

C

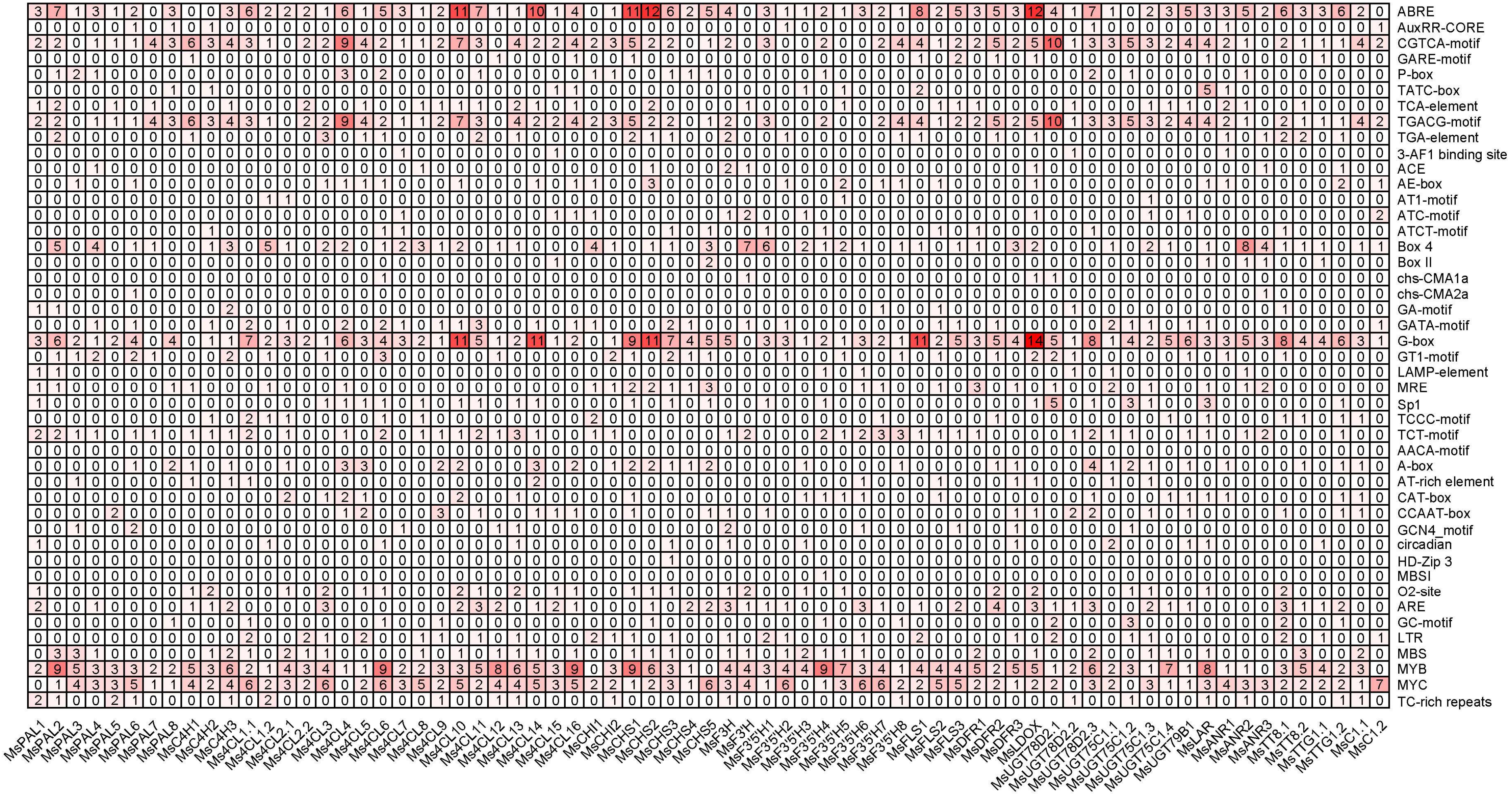

Cui *et al*. Flavonoid biosynthetic genes in Musaceae. AoB Plants 2024.

D

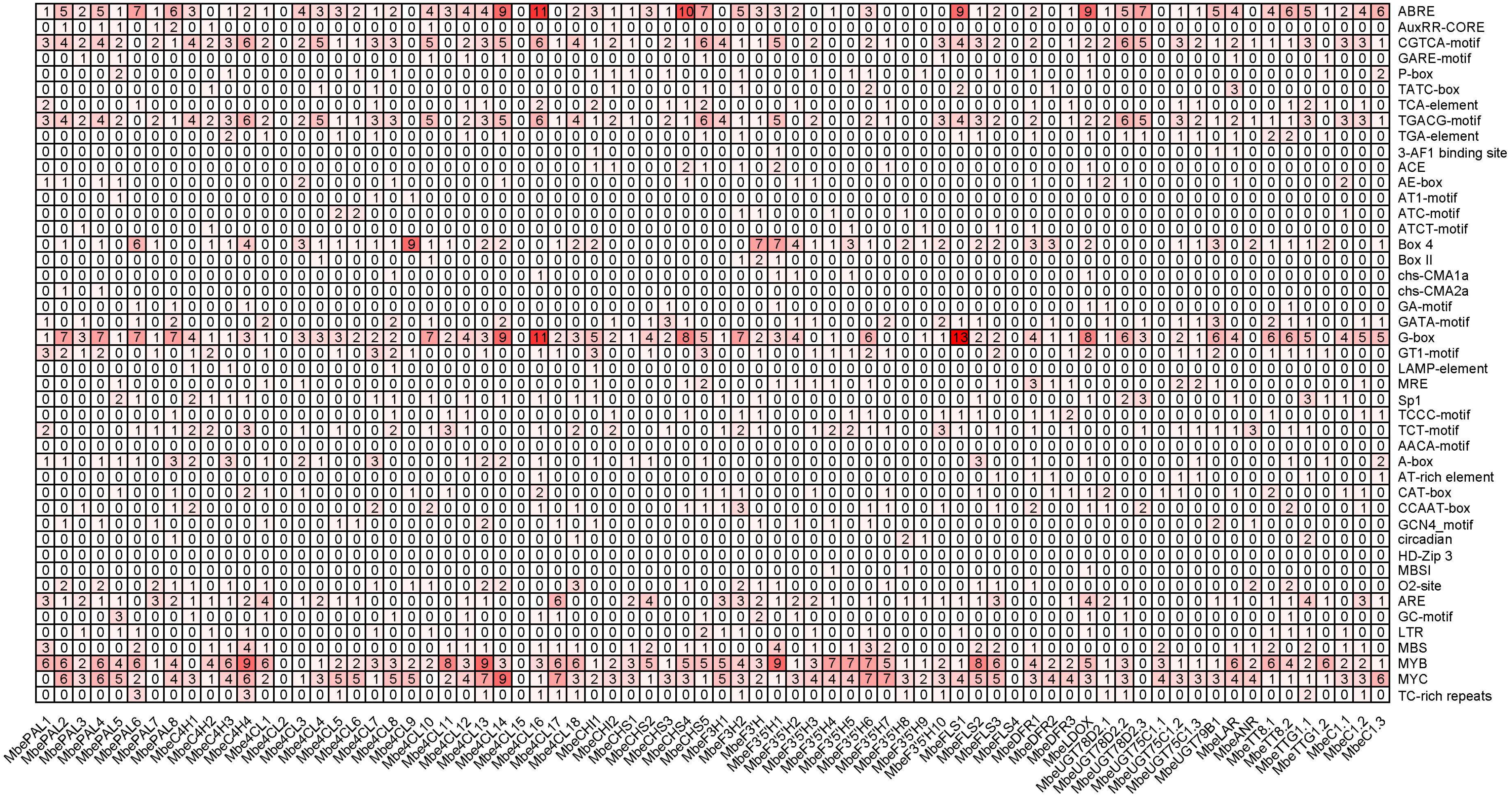

ABRE
AuxRR-CORE
CGTCA-motif
GARE-motif
P-box
TATC-box
TCA-element
TGACG-motif
TGA-element
3-AF1 binding site
ACE
AE-box
AT1-motif
ATC-motif
ATCT-motif
Box 4
Box II
chs-CMA1a
chs-CMA2a
GA-motif
GATA-motif
G-box
GT1-motif
LAMP-element
MRE
Sp1
TCCC-motif
TCT-motif
AACA-motif
A-box
AT-rich element
CAT-box
CCAAT-box
GCN4_motif
circadian
HD-Zip 3
MBSI
O2-site
ARE
GC-motif
LTR
MBS
MYB
MYC
TC-rich repeats
MbePAL1
MbePAL2
MbePAL3
MbePAL4
MbePAL5
MbePAL6
MbePAL7
MbePAL8
MbeC4H1
MbeC4H2
MbeC4H3
MbeC4H4
Mbe4CL1
Mbe4CL2
Mbe4CL3
Mbe4CL4
Mbe4CL5
Mbe4CL6
Mbe4CL7
Mbe4CL8
Mbe4CL9
Mbe4CL10
Mbe4CL11
Mbe4CL12
Mbe4CL13
Mbe4CL14
Mbe4CL15
Mbe4CL16
Mbe4CL17
Mbe4CL18
MbeCHI1
MbeCHI2
MbeCHS1
MbeCHS2
MbeCHS3
MbeCHS4
MbeCHS5
MbeF3H1
MbeF3H2
MbeF3'H
MbeF3'5'H1
MbeF3'5'H2
MbeF3'5'H3
MbeF3'5'H4
MbeF3'5'H5
MbeF3'5'H6
MbeF3'5'H7
MbeF3'5'H8
MbeF3'5'H9
MbeF3'5'H10
MbeFLS1
MbeFLS2
MbeFLS3
MbeFLS4
MbeDFR1
MbeDFR2
MbeDFR3
MbeLDOX
MbeUGT78D2.1
MbeUGT78D2.2
MbeUGT78D2.3
MbeUGT75C1.1
MbeUGT75C1.2
MbeUGT75C1.3
MbeUGT79B1
MbeLAR
MbeANR
MbeTT8.1
MbeTT8.2
MbeTTG1.1
MbeTTG1.2
MbeC1.1
MbeC1.2
MbeC1.3

E

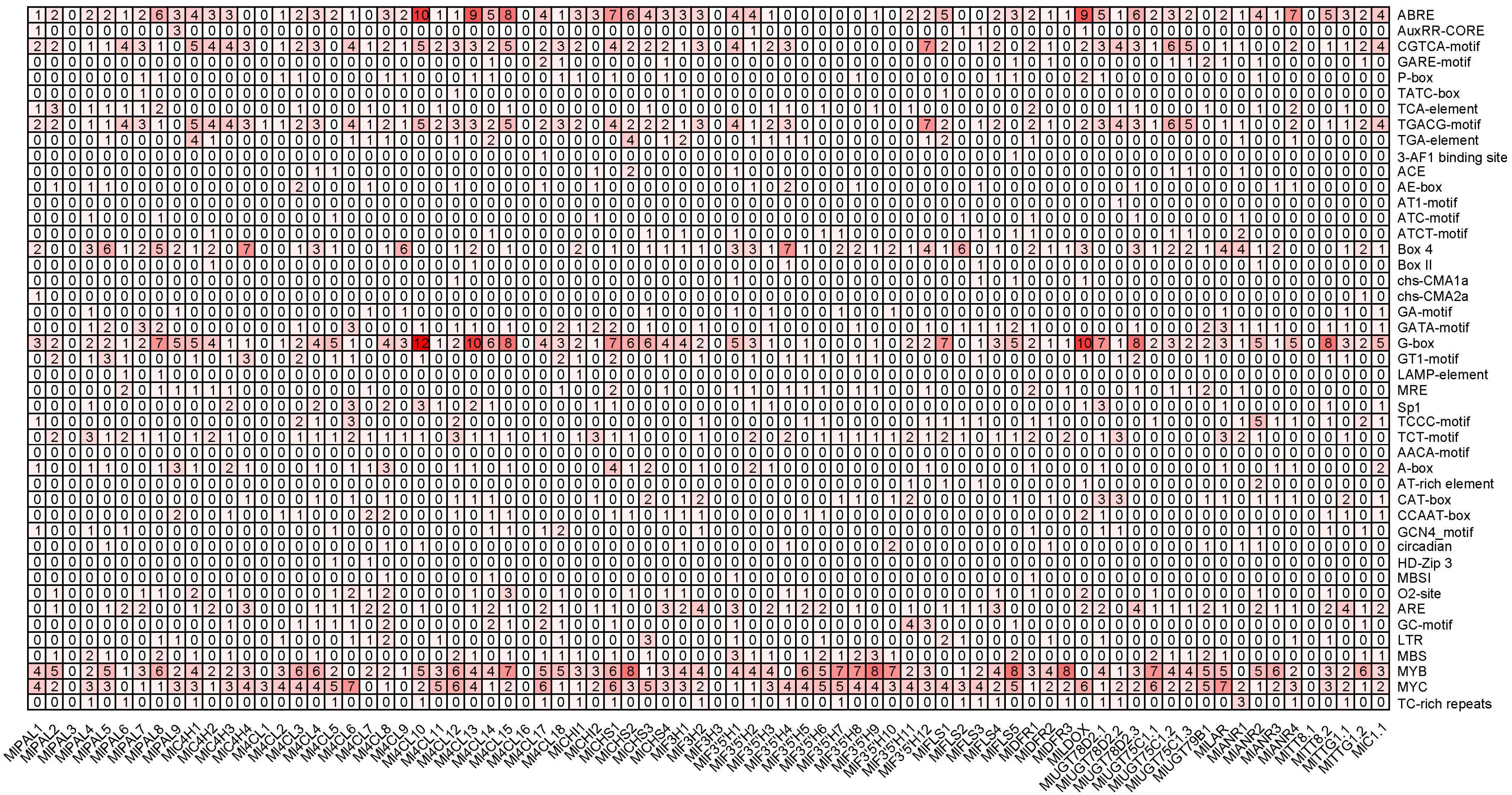

Cui *et al*. Flavonoid biosynthetic genes in Musaceae. AoB Plants 2024.

F

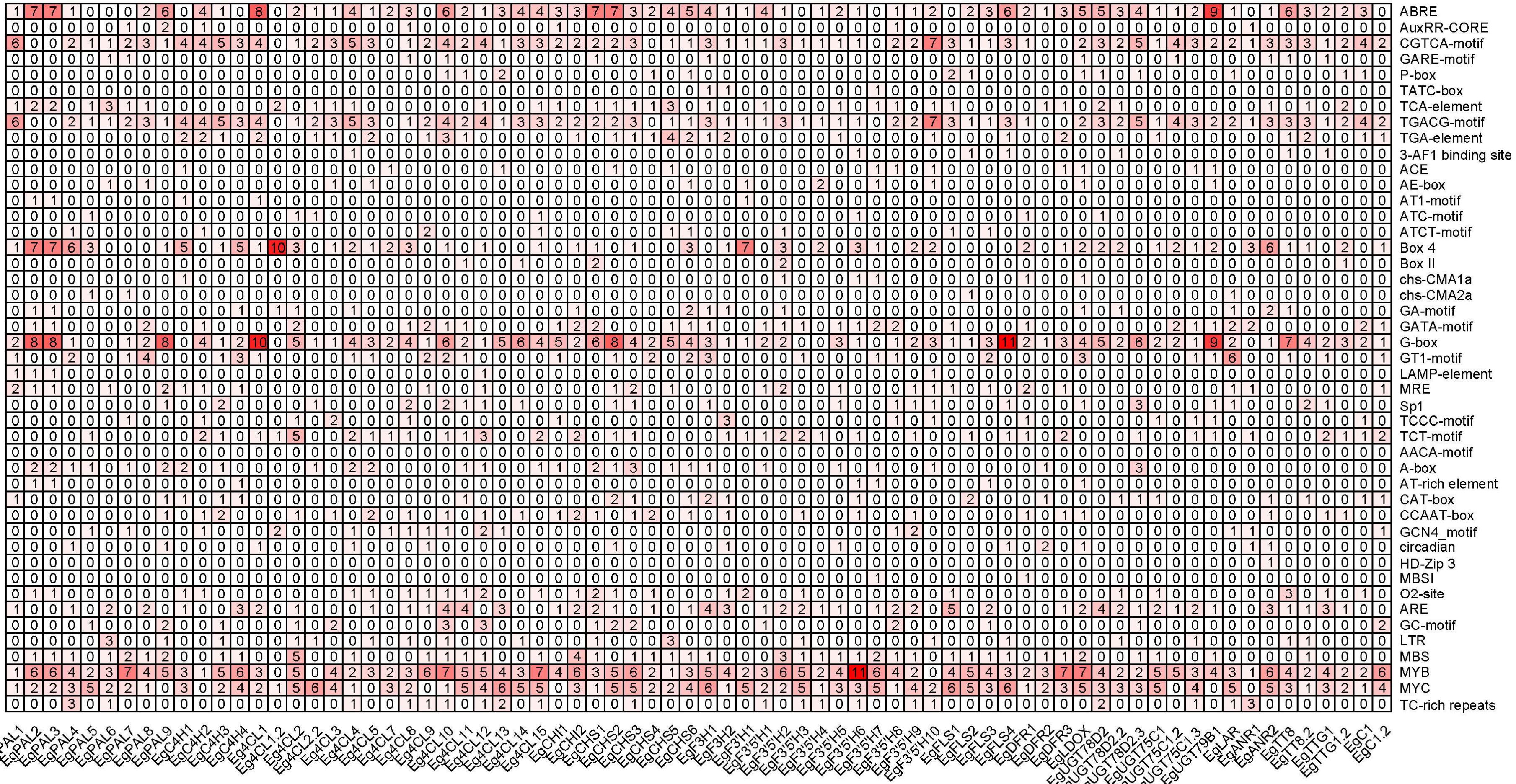

Figure S4. Cis-acting elements in flavonoid biosynthetic genes of Musaceae. **(A)** *Musa acuminata* (*x*=11). **(B)** *M. balbisiana* (*x*=11). **(C)** *M. schizocarpa* (*x*=11). **(D)** *M. beccarii* (*x*=9). **(E)** *Musella lasiocarpa* (*x*=9). **(F)** *Ensete glaucum* (*x*=9)

Table S1. PFAM accession of the conserved domain of flavonoid biosynthetic protein

Protein	HMM1	HMM2	HMM3	HMM4	HMM5	HMM6
PAL	PF00221					
C4H	PF00067					
4CL	PF00501					
CHI	PF02431	PF16035	PF16036			
CHS	PF02797	PF00195				
F3H	PF03171	PF14226				
F3'H	PF00067					
F3'5'H	PF00067					
FLS	PF03171	PF14226				
DFR	PF01370	PF07993	PF13460	PF05368		
LDOX	PF03171	PF14226				
LAR	PF01370	PF07993	PF05368			
ANR	PF01073	PF01370	PF16363	PF07993	PF13460	PF05368
UFGT	PF00201					
bHLH	PF14215					
C1	PF13921					
WD40	PF00400					

Table S2. Flavonoid biosynthetic genes (FBGs) in *A. thaliana*, *B. distachyon*, *O. sativa*, *S. bicolor*, *Z. officinale*, *M. beccarii, M. lasiocarpa*, *E. glaucum*, *M. acuminata*, *M. balbisiana*, and *M. schizocharpa*

	A. thaliana	***B. distachyon***	***A. longiglumis***	***O. sativa***	***S. bicolor***	***Z. officinale***	***E. glaucum***	***M. lasiocarpa***	***M. beccarii***	***M. acuminata***	***M. balbisiana***	***M. schizocharpa***
**PAL**	AT2G37040	XP_003575396.1	AL6G04288	Os04t0518100-01	Sobic.006G148800.1.p	zingiber_officinale042419393.1	Eg01_t040920	ML1h04G02672.1	Mbe05_t009880.2	Macma4_01_g04800.1	Mba09_g14610	Ms01t004140
	AT3G53260	XP_003575400.1	AL1G02130	Os02t0626100-02	Sobic.004G220300.1.p	zingiber_officinale042419394.1	Eg05_t014880	ML1h01G00856.1	Mbe06_t040740.1	Macma4_05_g03790.1	Mba11_g20430	Ms05t120010
	AT5G04230	XP_003580144.1	AL3G02155	Os11t0708900-01	Sobic.001G160500.1.p	zingiber_officinale042389150.1	Eg05_t014870	ML1h07G00086.1	Mbe07_t015550.1	Macma4_11_g15930.1	Mba08_g14430	Ms11t313180
	AT3G10340	XP_003575404.1	AL6G03333	Os12t0520200-01	Sobic.006G148900.1.p	zingiber_officinale042378355.1	Eg02_t018790	ML1h04G03282.1	Mbe06_t040740.2	Macma4_02_g02760.1	Mba02_g01510	Ms02t024050
		XP_003575365.1	AL6G03335	Os04t0518400-01	Sobic.004G220700.1.p	zingiber_officinale042378354.1	Eg09_t011330	ML1h07G01701.1	Mbe06_t048840.1	Macma4_05_g21700.1	Mba11_g14220	Ms08t220210
		XP_003575403.1	AL6G03803	Os05t0427400-00	Sobic.004G220600.1.p	zingiber_officinale042378359.1	Eg02_t001010	ML1h02G00424.1	Mbe03_t031150.2	Macma4_11_g22170.1	Mba05_g13940	Ms11t319150
		XP_003575240.2	AL6G03821	Os02t0626600-00	Sobic.004G220500.1.p		Eg09_t017980	ML1h01G01720.1	Mbe01_t022110.1	Macma4_08_g14800.1	Mba01_g14280	Ms05t135820
		XP_003575238.1	AL6G03336	Os02t0627100-01	Sobic.004G220400.1.p		Eg05_t024420	ML1h01G01719.1	Mbe08_t037020.1	Macma4_09_g16190.1		Ms09t254450

**PAL**	ALContig168G00001	Os02t0626400-02	Eg06_t005550	ML1h06G02093.1
	AL4G00959	Os12t0461300-00		
	AL4G00972	Os12t0461050-00		
	AL4G00961	Os08t0308300-00		
	AL4G00973			
	AL4G00960			
	AL4G00974			
	AL4G03334			
	ALContig3G00022			
	AL1G02823			
	AL1G02824			
	AL1G02822			
	AL4G01837			
	AL4G01838			
	ALContig3G00021			
	AL4G01839			
	ALContig3G00020			
	AL6G03337			

**C4H**	AT2G30490	XP_003564495.1	AL1G02710	Os05t0320700-02	Sobic.003G337400.1.p	zingiber_officinale042446994.1	Eg08_t019660	ML1h05G01754.1	Mbe01_t038520.1	Macma4_10_g16840.1	Mba09_g04380	Ms10t281630
		XP_003568699.1	AL4G03405		Sobic.002G126600.1.p	zingiber_officinale042470427.1	Eg07_t003130	ML1h08G00252.1	Mbe10_t000860.1	Macma4_07_g20380.1	Mba10_g11450	Ms07t197940
			AL1G06020			zingiber_officinale042429383.1	Eg08_t048930	ML1h05G04363.1	Mbe02_t033650.1	Macma4_06_g10450.1	Mba06_g09560	Ms09t244050
			AL1G06010				Eg07_t034110	ML1h08G03392.1	Mbe08_t025090.1	Macma4_09_g04800.1	Mba07_g18380	
			AL1G06052									
**4CL**	AT1G51680	XP_010229245.1	AL7G03614	Os04t0310800-01	Sobic.003G004900.1.p	zingiber_officinale042379474.1	Eg02_t002700	ML1h07G00167.1	Mbe06_t016280.1	Macma4_08_g13040.1	Mba05_g21540	Ms08t218760
	AT3G21240	XP_003574399.2	AL1G00350	Os03t0132000-01	Sobic.006G079500.2.p	zingiber_officinale042379472.1	Eg05_t023200	ML1h08G00140.1	Mbe03_t000510.1	Macma4_05_g22890.1	Mba08_g12720	Ms05t136800
	AT1G65060	XP_010235057.2	AL1G01139	Os07t0280200-01	Sobic.001G516600.1.p	zingiber_officinale042432142.1	Eg02_t037340	ML1h04G04085.1	Mbe07_t014630.1	Macma4_02_g21360.1	Mba02_g18560	Ms02t041000
	AT3G21230	XP_014757415.1	AL7G03615	Os01t0901600-01	Sobic.002G158900.1.p	zingiber_officinale042457757.1	Eg09_t018540	ML1h03G00142.1	Mbe05_t019740.1	Macma4_11_g22750.1	Mba11_g20940	Ms11t319670
		XP_003577602.1	AL5G02610	Os01t0901500-01	Sobic.001G189300.1.p	zingiber_officinale042457755.1	Eg09_t002410	ML1h02G00664.1	Mbe01_t019280.1	Macma4_11_g14090.1	Mba11_g05710	Ms11t311430
		XP_014753483.1	AL3G05698	Os10t0578950-00	Sobic.003G391600.1.p	zingiber_officinale042469802.1	Eg02_t001820	ML1h08G03149.1	Mbe01_t019320.2	Macma4_08_g13960.1	Mba11_g12600	Ms08t219500
		XP_003564855.1	ALContig36G00007	Os08t0143300-00	Sobic.002G009600.1.p	zingiber_officinale042469803.1	Eg05_t033420	ML1h01G00403.1	Mbe05_t004680.1	Macma4_05_g28920.1	Mba08_g13620	Ms05t142250
		XP_003558787.1	AL2G03972	Os03t0152400-01	Sobic.002G009700.2.p	zingiber_officinale042467448.1	Eg01_t038400	ML1h01G00405.1	Mbe03_t020730.1	Macma4_11_g06130.1	Mba05_g27160	Ms11t304430

**4CL**	XP_003570153.1	AL3G05400	Os02t0697400-01	Sobic.001G282800.1.p	zingiber_officinale042469801.1	Eg03_t041750	ML1h01G00972.1	Mbe05_t011100.1	Macma4_01_g07350.1	Mba01_g16650	Ms01t006680
	XP_010235187.1	AL3G05411	Os06t0656500-01	Sobic.001G187000.2.p	zingiber_officinale042392253.1	Eg04_t006840	ML1h07G00248.1	Mbe07_t013640.1	Macma4_03_g14310.1	Mba03_g13490	Ms03t059300
	XP_003570513.1	AL3G05412	Os06t0656500-02	Sobic.001G500800.4.p	zingiber_officinale042375877.1	Eg01_t014520	ML1h04G02613.1	Mbe02_t027270.1	Macma4_06_g39580.1	Mba06_g36630	Ms06t178240
	XP_003563338.1	AL3G05415	Os02t0177600-01	Sobic.004G272700.1.p	zingiber_officinale042470465.1	Eg01_t025930	ML1h07G03382.1	Mbe06_t040140.1	Macma4_01_g19800.1	Mba01_g28370.1	Ms01t017810.1
	XP_003573604.1	AL4G04549	Os08t0245200-01	Sobic.010G214900.1.p	zingiber_officinale042375841.1	Eg04_t025780	ML1h02G02377.1	Mbe03_t056570.1	Macma4_03_g13930.1	Mba04_g05720.1	Ms03t058920.1
	XP_003579357.2	AL3G06362	Os08t0448000-01	Sobic.004G062500.1.p	zingiber_officinale042468711.1	Eg04_t032900	ML1h09G01309.1	Mbe01_t005810.2	Macma4_04_g06110.1	Mba04_g12340.1	Ms04t083350.1
		AL6G03059	Os04t0683700-01	Sobic.007G089900.1.p	zingiber_officinale042375680.1	Eg07_t001640	ML1h08G00557.1	Mbe03_t000920.1	Macma4_04_g13130.1	Mba07_g04890.1	Ms04t089570.1
		AL5G02390	Os07t0639100-00	Sobic.007G145600.1.p	zingiber_officinale042451259.1	Eg07_t014740	ML1h06G03426.1	Mbe07_t022330.2	Macma4_07_g05220.1	Mba07_g17040.1	Ms07t183930.1
		AL2G03024		Sobic.006G272600.1.p	zingiber_officinale042471368.1		ML1h06G02761.1	Mbe04_t006230.1	Macma4_07_g18890.1		Ms07t196560.1
		AL2G03265			zingiber_officinale042405667.1		ML1h09G02144.1	Mbe04_t013920.2	Macma4_09_g24760.1		Ms09t262230.1
		AL5G02618			zingiber_officinale042405669.1						
		AL7G00468			zingiber_officinale042441626.1						

**4CL**			ALContig115G00003			zingiber_officinale042388974.1						
			AL1G00990			zingiber_officinale042452682.1						
			AL6G01256			zingiber_officinale042376059.1						
			ALContig6G00008			zingiber_officinale042447280.1						
**CHI**	AT3G55120	XP_003559241.1	AL3G02323	Os03t0819600-02	Sobic.001G035600.1.p	zingiber_officinale042387863.1	Eg01_t015550	ML1h09G01419.1	Mbe04_t020690.1	Macma4_04_g18950.1	Mba11_g21110	Ms11t319860
		XP_010238805.1	AL7G01891	Os12t0115700-01	Sobic.008G030100.2.p	zingiber_officinale042392070.1	Eg09_t018750	ML1h04G02595.1	Mbe06_t039860.1	Macma4_11_g22930.1	Mba04_g18040	Ms04t093250
				Os11t0116300-01								
**CHS**	AT5G13930	XP_003577513.1	AL7G05184	Os07t0214900-01	Sobic.007G058900.1.p	zingiber_officinale042470361.1	Eg08_t046640	ML1h05G01818.1	Mbe01_t038010.2	Macma4_06_g12560.1	Mba06_g11590	Ms10t280970
				Os11t0530600-01	Sobic.005G137200.1.p	zingiber_officinale042436018.1	Eg08_t040870	ML1h05G03634.1	Mbe01_t038030.1	Macma4_06_g18170.1	Mba06_g16830	Ms06t158650
					Sobic.005G137000.1.p	zingiber_officinale042434575.1	Eg08_t020390	ML1h05G04408.1	Mbe01_t041060.1	Macma4_10_g16190.1	Mba10_g10880	MsSC51t327370
					Sobic.005G136450.1.p	zingiber_officinale042434725.1	Eg08_t049390	ML1h05G04406.1	Mbe01_t047580.1	Macma4_06_g10020.1	Mba06_g09110	MsSC51t327380
					Sobic.005G136600.1.p	zingiber_officinale042435235.1	Eg08_t049400		Mbe11_t002790.1	Macma4_06_g10010.1	Mba06_g09080	MsSC66t327440

**CHS**					Sobic.005G136800.1.p	zingiber_officinale042378779.1	Eg08_t049410			Macma4_10_g16140.1	Mba06_g09090	
					Sobic.005G137100.1.p	zingiber_officinale042434761.1					Mba06_g09120	
					Sobic.005G136200.1.p						Mba10_g10820	
					Sobic.005G137300.1.p							
					Sobic.005G136300.1.p							
**F3H**	AT3G51240	XP_003579435.1	AL4G00149	Os04t0662600-00	Sobic.003G021700.1.p	zingiber_officinale042448017.1	Eg07_t027880	ML1h07G02004.1	Mbe07_t035880.1	Macma4_07_g17110.1	Mba02_g04670	Ms02t027200
					Sobic.004G310100.1.p		Eg02_t022060	ML1h08G02519.1	Mbe02_t010150.1	Macma4_02_g06130.1	Mba07_g15320	
**F3'H**	AT5G07990	XP_003574810.2	AL7G01297	Os10t0317900-02	Sobic.009G162500.1.p	zingiber_officinale042377047.1	Eg03_t018110	ML1h03G02252.1	Mbe06_t019910.1	Macma4_03_g33170.1	Mba01_g02030	Ms03t075990
		XP_010233685.1	AL2G05114	Os10t0320100-01	Sobic.004G200900.1.p	zingiber_officinale042458020.1						
		XP_003562884.2			Sobic.004G201100.1.p							
		XP_003577475.1			Sobic.004G200800.1.p							
					Sobic.004G200833.1.p							

**F3'5'H**	AT4G12300	XP_014753340.1	AL3G03749	Os03t0367101-00	Sobic.001G360800.2.p	zingiber_officinale042422124.1	Eg09_t020640	ML1h07G00690.1	Mbe07_t008510.1	Macma4_08_g08290.1	Mba08_g07950	Ms08t214430
	AT4G12320	XP_003567588.1			Sobic.001G360700.1.p	zingiber_officinale042409260.1	Eg02_t007770	ML1h04G02425.1	Mbe06_t049500.1	Macma4_11_g05110.1	Mba11_g04840	Ms09t255750
	AT4G12310					zingiber_officinale042375298.1	Eg06_t004020	ML1h07G00951.1	Mbe02_t005590.1	Macma4_08_g05480.1	Mba09_g16030	Ms08t211840
	AT4G12330					zingiber_officinale042375301.1	Eg07_t042500	ML1h08G03854.1	Mbe02_t005610.1	Macma4_09_g17660.1	Mba10_g00740	Ms02t024370.1
	AT5G44620					zingiber_officinale042375302.1	Eg02_t010650	ML1h06G02235.1	Mbe02_t006410.1	Macma4_10_g01240.1	Mba02_g01840.1	Ms02t024400.1
	AT4G22690					zingiber_officinale042375303.1	Eg02_t019070	ML1h06G02237.1	Mbe02_t005600.1	Macma4_10_g01260.1	Mba02_g01880.1	Ms02t024410.1
	AT4G22710					zingiber_officinale042375299.1	Eg02_t019100	ML1h07G01736.1	Mbe08_t039500.1	Macma4_02_g03040.1	Mba02_g01890.1	Ms02t024420.1
						zingiber_officinale042410615.1	Eg02_t019110	ML1h07G01737.1	Mbe09_t008950.1	Macma4_02_g03240.1	Mba02_g01900.1	Ms11t312500.1
							Eg02_t019180	ML1h02G01787.1	Mbe09_t008960.1	Macma4_02_g03250.1	Mba02_g01910.1	
							Eg09_t010710	ML1h03G01872.1	Mbe07_t005300.1	Macma4_02_g03260.1	Mba02_g01920.1	
								ML1h07G01741.1		Macma4_02_g03290.1	Mba02_g01940.1	
								ML1h04G03337.1		Macma4_02_g03300.1	Mba02_g01950.1	

										Macma4_11_g15260.1	Mba11_g13650.1	
**FLS**	AT5G08640	XP_003570562.1	AL7G03380	Os02t0767300-01(OsFLS)	Sobic.003G021700.1.p	zingiber_officinale042378807.1	Eg06_t006150	ML1h03G01030.1	Mbe08_t015900.1	Macma4_03_g07220.1	Mba08_g23630	Ms08t229420
	AT5G63580		AL6G04177		Sobic.004G310100.2.p	zingiber_officinale042406407.1	Eg01_t007540	ML1h05G00636.1	Mbe02_t047300.2	Macma4_08_g24690.1	Mba10_g22000	Ms03t052640
	AT5G63590		AL6G04187			zingiber_officinale042406408.1	Eg08_t007020	ML1h05G00635.1	Mbe08_t036240.1		Mba10_g21980	Ms10t292840
	AT5G63595						Eg03_t031640	ML1h06G02043.1	Mbe03_t008100.1		Mba03_g06940	
	AT5G63600							ML1h09G00678.1			Mba09_g14060	
	AT5G43935											
**DFR**	AT5G42800	XP_010232065.1	AL1G03574	Os01t0633500-01	Sobic.004G050200.1.p	zingiber_officinale042421830.1	Eg09_t036940	ML1h04G00849.1	Mbe04_t036630.1	Macma4_04_g33220.1	Mba04_g31800	Ms04t107810
		XP_003569417.2			Sobic.003G230900.1.p		Eg04_t027830	ML1h06G03238.1	Mbe06_t019010.2	Macma4_03_g33910.1	Mba01_g01310	Ms04t087800
					Sobic.003G231000.1.p		Eg03_t018610	ML1h03G02164.1	Mbe04_t011700.1	Macma4_04_g11180.1		Ms03t076720
					Sobic.009G043800.1.p							
**LDOX/ANS**	AT4G22880		AL7G00463	Os01t0372500-00	Sobic.004G000700.1.p	zingiber_officinale042431763.1	Eg05_t014690	ML1h01G01735.1	Mbe03_t031300.1	Macma4_05_g03920.1	Mba05_g13780	Ms05t120140
			AL7G00477	Os06t0626700-00								

**UGT78D2**	AT5G17050	XP_014753234.1	AL5G00921	Os06t0192100-00	Sobic.010G070200.1.p	zingiber_officinale042423608.1	Eg08_t008340	ML1h09G00476.1	Mbe07_t034770.1	Macma4_10_g27580.1	Mba03_g04830	Ms10t291590
		XP_024313830.1		Os07t0148200-01	Sobic.002G369600.1.p	zingiber_officinale042410446.1	Eg06_t008220	ML1h06G01843.1	Mbe02_t045890.1	Macma4_07_g16200.1	Mba10_g20800	Ms07t193970
						zingiber_officinale042388078.1	Eg01_t005290	ML1h05G00763.1	Mbe03_t010580.1	Macma4_03_g04880.1	Mba07_g14580	Ms03t050760
						zingiber_officinale042460585.1						
						zingiber_officinale042458777.1						
						zingiber_officinale042458785.1						
						zingiber_officinale042458786.1						
						zingiber_officinale042458779.1						
						zingiber_officinale042458782.1						
						zingiber_officinale042458775.1						
						zingiber_officinale042458790.1						
						zingiber_officinale042458800.1						

**UGT78D2**	zingiber_officinale042458794.1<br>zingiber_officinale042458797.1<br>zingiber_officinale042458783.1<br>zingiber_officinale042458788.1<br>zingiber_officinale042458780.1<br>zingiber_officinale042458787.1<br>zingiber_officinale042458795.1<br>zingiber_officinale042458796.1<br>zingiber_officinale042458799.1<br>zingiber_officinale042458791.1<br>zingiber_officinale042458798.1<br>zingiber_officinale042458778.1

**UGT78D2**						zingiber_officinale042458789.1<br>zingiber_officinale042458784.1<br>zingiber_officinale042458781.1<br>zingiber_officinale042458793.1						
**UGT75C1**	AT4G14090	XP_003576359.2	AL5G03818	Os06t0593800-01	Sobic.010G179150.1.p	zingiber_officinale042413493.1	Eg08_t003590	ML1h05G00327.1	Mbe02_t051150.1	Macma4_10_g31950.1	Mba10_g24560	Ms06t152710
		XP_024311125.1	AL5G03820	Os01t0179600-01	Sobic.010G179500.1.p	zingiber_officinale042413492.1	Eg08_t037030	ML1h05G03318.1	Mbe01_t034880.1	Macma4_06_g07210.1	Mba06_g06480	Ms06t152720
		XP_003560621.1	AL5G03827	Os05t0179950-00	Sobic.003G042900.1.p	zingiber_officinale042413495.1	Eg08_t037040	ML1h05G03317.1	Mbe01_t034870.1	Macma4_06_g07200.1	Mba06_g06490	Ms06t152730
		XP_003571494.1	AL6G02053	Os06t0593200-01	Sobic.009G066100.1.p	zingiber_officinale042413494.1				Macma4_06_g07190.1		Ms10t295740
		XP_003560622.3	AL6G02057	Os11t0145200-01	Sobic.010G178900.2.p	zingiber_officinale042453821.1						
		XP_010230853.1	AL5G00208	Os02t0203300-00	Sobic.010G179000.1.p	zingiber_officinale042405426.1						
		XP_024315058.1	AL7G04281		Sobic.005G032200.2.p	zingiber_officinale042404506.1						
		XP_003565425.2	AL5G00933		Sobic.006G097800.2.p							

		XP_003565426.1	AL3G02472		Sobic.008G011700.1.p							
**UGT79B1**	AT5G54060	XP_003560487.1	AL3G06005	Os11t0457300-01	Sobic.008G035600.1.p	zingiber_officinale042420251.1	Eg07_t037900	ML1h08G03185.1	Mbe08_t027530.1	Macma4_09_g07100.1		Ms09t246270
		XP_003560486.1			Sobic.005G033900.1.p							
					Sobic.005G033801.1.p							
**LAR**	0	XP_003561802.1	AL3G04509	Os03t0259400-00	0	0	Eg05_t019870	ML1h01G01259.1	Mbe05_t014700.1	Macma4_05_g19560.1	Mba05_g18490	Ms05t133790
**ANR**	AT1G61720	XP_003560368.1	AL4G03119	Os07t0601900-01	Sobic.002G366200.3.p	zingiber_officinale042454473.1	Eg06_t013840	ML1h06G01340.1	Mbe07_t028460.1	Macma4_07_g10640.1	Mba08_g01400	Ms08t207990
		XP_003562747.1	AL4G03124	Os07t0602000-01	Sobic.002G366100.1.p	zingiber_officinale042425557.1	Eg02_t015210	ML1h06G01342.1		Macma4_07_g10660.1		Ms07t188920
		XP_003559994.1	AL4G03120	Os07t0598000-02		zingiber_officinale042425546.1		ML1h06G01341.1		Macma4_07_g10670.1		Ms07t188910
		XP_003562745.1	AL4G03125	Os07t0601100-01		zingiber_officinale042425536.1		ML1h07G01339.1		Macma4_07_g10680.1		
		XP_003580613.1	AL5G00711	Os07t0601000-01						Macma4_08_g01440.1		
		XP_014751318.1	AL4G00442									
		XP_003580612.1	ALContig28G00015									

**ANR**		XP_003580614.1	AL4G00443<br>ALContig28G00016<br>AL3G05634<br>AL3G05652<br>AL4G00441<br>ALContig28G00014<br>AL4G00440<br>ALContig28G00013<br>AL4G00436<br>ALContig28G00010<br>AL4G00439<br>ALContig28G00012									
**TT8**	AT4G09820	XP_024312733.1	AL4G04360	Os07t0211500-01	Sobic.002G076600.4.p	zingiber_officinale042379222.1	Eg04_t020700	ML1h03G03510.1	Mbe06_t034420.1	Macma4_06_g25800.1	Mba06_g23950	MsSC186t326890
		XP_010230011.1					Eg03_t004200	ML1h02G03012.1	Mbe06_t001720.2	Macma4_03_g19880.1	Mba03_g18730	Ms03t064490
		XP_024312732.1										
	AT5G24520	XP_003570109.1	ALContig2G00010	Os02t0682500-01	Sobic.004G280800.1.p	zingiber_officinale042406919.1	Eg05_t009200	ML1h01G02333.1	Mbe03_t038300.1	Macma4_05_g10420.1	Mba05_g07670	Ms04t112120

**GLABRA1(TTG1)**						zingiber_officinale042451789.1<br>zingiber_officinale042377982.1	Eg09_t041330	ML1h04G00438.1	Mbe04_t041380.1	Macma4_04_g37870.1	Mba04_g36060	Ms05t125970
**C1**		XP_003561552.1	AL3G03580	Os06t0205100-02	Sobic.001G340900.1.p	zingiber_officinale042388738.1	Eg07_t019620	ML1h08G01785.1	Mbe01_t033880.1	Macma4_10_g21630.1	Mba10_g15520	Ms09t266370.1
			AL5G00993			zingiber_officinale042387580.1	Eg08_t035970	ML1h05G03234.1	Mbe03_t051550.1	Macma4_09_g29590.1	Mba06_g05630	Ms06t151760.1
						zingiber_officinale042417551.1		ML1h05G01322.1	Mbe02_t039140.1		Mba09_g23680	
						zingiber_officinale042423943.1						
						zingiber_officinale042423006.1						

Table S3a. The information of flavonoid biosynthetic proteins identified in *M. beccarii*

ID	Name	length	MW(Da)	pI	Localizations
Mbe05_t009880.2	MbePAL1	557	60494.9	6.35	Cytoplasm
Mbe06_t040740.1	MbePAL2	713	77095.3	6.6	Cytoplasm
Mbe07_t015550.1	MbePAL3	709	76790	6.56	Cytoplasm
Mbe06_t040740.2	MbePAL4	557	60665	6.57	Cytoplasm
Mbe06_t048840.1	MbePAL5	701	75831.8	5.77	Cytoplasm
Mbe03_t031150.2	MbePAL6	423	46990.1	6.58	Cytoplasm
Mbe01_t022110.1	MbePAL7	713	77124.6	6.26	Cytoplasm
Mbe08_t037020.1	MbePAL8	757	83240.1	5.59	Cytoplasm
Mbe01_t038520.1	MbeC4H1	503	57545.6	9.56	Endoplasmic reticulum
Mbe10_t000860.1	MbeC4H2	503	57405.4	9.66	Endoplasmic reticulum
Mbe02_t033650.1	MbeC4H3	503	57436.4	9.69	Endoplasmic reticulum
Mbe08_t025090.1	MbeC4H4	503	57928	9.36	Endoplasmic reticulum
Mbe06_t016280.1	Mbe4CL1	545	59022.8	6.74	Cytoplasm
Mbe03_t000510.1	Mbe4CL2	650	70159.3	8.85	Peroxisome
Mbe07_t014630.1	Mbe4CL3	549	59243.6	8.73	Peroxisome
Mbe05_t019740.1	Mbe4CL4	625	66173.6	7.81	Peroxisome
Mbe01_t019280.1	Mbe4CL5	555	59588.4	8.79	Peroxisome
Mbe01_t019320.2	Mbe4CL6	503	53550.1	6.65	Cytoplasm
Mbe05_t004680.1	Mbe4CL7	550	58618.9	7.02	Peroxisome
Mbe03_t020730.1	Mbe4CL8	548	59184.8	8.21	Peroxisome
Mbe05_t011100.1	Mbe4CL9	544	58594.2	6.99	Peroxisome
Mbe07_t013640.1	Mbe4CL10	543	58425.9	8.34	Peroxisome

Mbe02_t027270.1	Mbe4CL11	552	59673.4	6.2	Cytoplasm
Mbe06_t040140.1	Mbe4CL12	568	61167.2	5.37	Cytoplasm
Mbe03_t056570.1	Mbe4CL13	543	59202.6	5.65	Cytoplasm
Mbe01_t005810.2	Mbe4CL14	471	50634.8	4.78	Cytoplasm
Mbe03_t000920.1	Mbe4CL15	548	59482.1	5.52	Cytoplasm
Mbe07_t022330.2	Mbe4CL16	400	43002.4	5.63	Cytoplasm
Mbe04_t006230.1	Mbe4CL17	543	59060.5	5.66	Cytoplasm
Mbe04_t013920.2	Mbe4CL18	404	43697	6.01	Cytoplasm
Mbe04_t020690.1	MbeCHI1	255	26702.3	4.84	Cytoplasm
Mbe06_t039860.1	MbeCHI2	209	23445.7	4.81	Cytoplasm
Mbe01_t038010.2	MbeCHS1	520	57925.8	8.55	Nucleus
Mbe01_t038030.1	MbeCHS2	392	43202.4	7.06	Cytoplasm
Mbe01_t041060.1	MbeCHS3	493	53761.4	6.64	Cytoplasm
Mbe01_t047580.1	MbeCHS4	480	52053.9	7.97	Plastid
Mbe11_t002790.1	MbeCHS5	392	42951.2	6.43	Cytoplasm
Mbe07_t035880.1	MbeF3H1	373	41367.6	5.1	Cytoplasm
Mbe06_t019910.1	MbeF3'H	520	57128.1	7.69	Endoplasmic reticulum
Mbe07_t008510.1	MbeF3'5'H1	503	56483.5	9.49	Endoplasmic reticulum
Mbe06_t049500.1	MbeF3'5'H2	505	56211.9	9.25	Endoplasmic reticulum
Mbe02_t005590.1	MbeF3'5'H3	505	56293	8.46	Endoplasmic reticulum
Mbe02_t005610.1	MbeF3'5'H4	370	40785.5	10.29	Endoplasmic reticulum
Mbe02_t006410.1	MbeF3'5'H5	540	59944.2	8.68	Endoplasmic reticulum
Mbe02_t005600.1	MbeF3'5'H6	208	23306.7	6.63	Cytoplasm
Mbe08_t039500.1	MbeF3'5'H7	505	56209.8	7.85	Endoplasmic reticulum
Mbe09_t008950.1	MbeF3'5'H8	507	56012.4	8.47	Endoplasmic reticulum

Mbe09_t008960.1	MbeF3'5'H9	507	56005.4	8.47	Endoplasmic reticulum
Mbe07_t005300.1	MbeF3'5'H10	523	57292.8	8.16	Endoplasmic reticulum
Mbe08_t015900.1	MbeFLS1	398	44295.4	7.57	Nucleus
Mbe02_t047300.2	MbeFLS2	291	32583.2	6.13	Cytoplasm
Mbe08_t036240.1	MbeFLS3	406	45798.9	8.64	Cytoplasm
Mbe03_t008100.1	MbeFLS4	333	37372.4	5.43	Cytoplasm
Mbe04_t036630.1	MbeDFR1	342	38256.6	6	Cytoplasm
Mbe06_t019010.2	MbeDFR2	378	42879.6	6.07	Cytoplasm
Mbe04_t011700.1	MbeDFR3	358	40360.1	6.29	Cytoplasm
Mbe03_t031300.1	MbeLDOX	398	44831.2	5.69	Cytoplasm|Nucleus
Mbe07_t034770.1	MbeUGT78D2.1	458	47917.9	6.45	Cytoplasm
Mbe02_t045890.1	MbeUGT78D2.2	455	47753.5	6.55	Cytoplasm
Mbe03_t010580.1	MbeUGT78D2.3	361	38355	6.05	Cytoplasm
Mbe02_t051150.1	MbeUGT75C1.1	501	54513	5.31	Cytoplasm
Mbe01_t034880.1	MbeUGT75C1.2	579	64964.9	6.66	Cytoplasm
Mbe01_t034870.1	MbeUGT75C1.3	473	52570.3	5.1	Cytoplasm
Mbe08_t027530.1	MbeUGT79B1	471	51052.7	7.07	Cytoplasm
Mbe05_t014700.1	MbeLAR	358	38681.8	6.84	Cytoplasm
Mbe07_t028460.1	MbeANR	341	37711.7	5.43	Cytoplasm
Mbe06_t034420.1	MbeTT8.1	693	77470.9	6.14	Nucleus
Mbe06_t001720.2	MbeTT8.2	643	72324	5.03	Nucleus
Mbe03_t038300.1	MbeTTG1.1	378	41003.5	4.55	Cytoplasm|Nucleus
Mbe04_t041380.1	MbeTTG1.2	340	36953	4.67	Cytoplasm|Nucleus
Mbe01_t033880.1	MbeC1.1	274	30268.9	7.92	Nucleus
Mbe03_t051550.1	MbeC1.2	260	28985.5	7.47	Nucleus

Mbe02_t039140.1	MbeC1.3	278	30575.2	7.71	Nucleus

Table S3b. The information of flavonoid biosynthetic proteins identified in *M. lasiocarpa*

ID	Name	length	MW(Da)	pI	Localizations
ML1h04G02672.1	MlPAL1	713	76972	6.22	Cytoplasm
ML1h01G00856.1	MlPAL2	711	76675	6.43	Cytoplasm
ML1h07G00086.1	MlPAL3	709	77027.1	6.66	Cytoplasm
ML1h04G03282.1	MlPAL4	703	76163.1	5.66	Cytoplasm
ML1h07G01701.1	MlPAL5	711	76896.7	5.98	Cytoplasm
ML1h02G00424.1	MlPAL6	712	77188.7	6.32	Cytoplasm
ML1h01G01720.1	MlPAL7	709	76855.9	6.51	Cytoplasm|Nucleus
ML1h01G01719.1	MlPAL8	709	76908.9	6.63	Cytoplasm|Nucleus
ML1h06G02093.1	MlPAL9	757	83181.1	6.04	Cytoplasm
ML1h05G01754.1	MlC4H1	536	61135.7	9.79	Endoplasmic reticulum
ML1h08G00252.1	MlC4H2	481	55154.7	9.75	Endoplasmic reticulum
ML1h05G04363.1	MlC4H3	503	57631.6	9.67	Endoplasmic reticulum
ML1h08G03392.1	MlC4H4	503	57911.1	9.22	Endoplasmic reticulum
ML1h07G00167.1	Ml4CL1	549	59273.6	8.93	Peroxisome
ML1h08G00140.1	Ml4CL2	563	59603.8	6.52	Peroxisome
ML1h04G04085.1	Ml4CL3	548	59500.3	9.04	Peroxisome
ML1h03G00142.1	Ml4CL4	547	58681.3	7.5	Peroxisome
ML1h02G00664.1	Ml4CL5	557	59598.3	8.22	Peroxisome
ML1h08G03149.1	Ml4CL6	555	58892.2	8.21	Peroxisome
ML1h01G00403.1	Ml4CL7	550	58556.7	6.89	Peroxisome
ML1h01G00405.1	Ml4CL8	550	58810.2	7.2	Peroxisome

ML1h01G00972.1	Ml4CL9	546	59097.7	7.85	Peroxisome
ML1h07G00248.1	Ml4CL10	515	55430.6	6.94	Peroxisome
ML1h04G02613.1	Ml4CL11	568	61229.3	5.37	Cytoplasm
ML1h07G03382.1	Ml4CL12	563	60560.5	5.64	Cytoplasm
ML1h02G02377.1	Ml4CL13	548	59367.9	5.16	Cytoplasm
ML1h09G01309.1	Ml4CL14	548	59368	5.07	Cytoplasm
ML1h08G00557.1	Ml4CL15	543	59144.4	5.1	Cytoplasm
ML1h06G03426.1	Ml4CL16	543	59167.6	5.66	Cytoplasm
ML1h06G02761.1	Ml4CL17	550	59656.4	6.38	Cytoplasm
ML1h09G02144.1	Ml4CL18	548	59473.2	6.4	Cytoplasm|Peroxisome
ML1h09G01419.1	MlCHI1	236	24960.1	5.12	Cytoplasm
ML1h04G02595.1	MlCHI2	211	23702.1	4.79	Cytoplasm
ML1h05G01818.1	MlCHS1	392	42787.9	6.25	Cytoplasm
ML1h05G03634.1	MlCHS2	392	42997.2	6.52	Cytoplasm
ML1h05G04408.1	MlCHS3	777	85121.1	6.34	Cytoplasm
ML1h05G04406.1	MlCHS4	392	43055.3	7.31	Cytoplasm
ML1h07G02004.1	MlF3H1	373	41487	5.52	Cytoplasm
ML1h08G02519.1	MlF3H2	373	41386.7	5.35	Cytoplasm
ML1h03G02252.1	MlF3H3	520	57489.4	6.99	Endoplasmic reticulum
ML1h07G00690.1	MlF3'5'H1	445	49816.4	8.45	Mitochondrion
ML1h04G02425.1	MlF3'5'H2	506	56209.3	8.9	Endoplasmic reticulum
ML1h07G00951.1	MlF3'5'H3	505	55905.5	7.84	Endoplasmic reticulum
ML1h08G03854.1	MlF3'5'H4	507	56073.5	8.08	Endoplasmic reticulum
ML1h06G02235.1	MlF3'5'H5	505	56222.8	7.59	Endoplasmic reticulum
ML1h06G02237.1	MlF3'5'H6	505	56373.9	7.85	Endoplasmic reticulum

ML1h07G01736.1	MlF3'5'H7	505	56088.7	7.72	Endoplasmic reticulum
ML1h07G01737.1	MlF3'5'H8	504	56084.8	8.58	Endoplasmic reticulum
ML1h02G01787.1	MlF3'5'H9	505	56248.9	8.04	Endoplasmic reticulum
ML1h03G01872.1	MlF3'5'H10	505	56131.8	8.59	Endoplasmic reticulum
ML1h07G01741.1	MlF3'5'H11	505	56186.8	9.02	Endoplasmic reticulum
ML1h04G03337.1	MlF3'5'H12	454	50867	7.22	Mitochondrion
ML1h03G01030.1	MlFLS1	333	37327.5	6.64	Cytoplasm
ML1h05G00636.1	MlFLS2	335	37718.1	5.94	Cytoplasm|Nucleus
ML1h05G00635.1	MlFLS3	700	76851.6	8.14	Mitochondrion
ML1h06G02043.1	MlFLS4	348	38764	6.74	Cytoplasm|Nucleus
ML1h09G00678.1	MlFLS5	333	37444.5	5.14	Cytoplasm
ML1h04G00849.1	MlDFR1	342	38190.6	6.29	Cytoplasm
ML1h06G03238.1	MlDFR2	358	40584.3	6.14	Cytoplasm
ML1h03G02164.1	MlDFR3	356	40126.6	4.96	Cytoplasm
ML1h01G01735.1	MlLDOX	360	40563.4	5.66	Cytoplasm
ML1h09G00476.1	MlUGT78D2.1	459	48291.3	6.14	Cytoplasm
ML1h06G01843.1	MlUGT78D2.2	458	47589.5	6.27	Cytoplasm
ML1h05G00763.1	MlUGT78D2.3	455	47828.7	6.61	Cytoplasm
ML1h05G00327.1	MlUGT75C1.1	477	52172.3	4.98	Cytoplasm
ML1h05G03318.1	MlUGT75C1.2	464	51172.8	4.77	Cytoplasm
ML1h05G03317.1	MlUGT75C1.3	466	51540	5.25	Cytoplasm
ML1h08G03185.1	MlUGT79B1	694	75152.1	5.43	Endoplasmic reticulum
ML1h01G01259.1	MlLAR	353	38132.1	6.84	Cytoplasm
ML1h06G01340.1	MlANR1	337	37083.1	5.26	Cytoplasm
ML1h06G01342.1	MlANR2	336	37121.1	5.43	Cytoplasm

ML1h06G01341.1	MlANR3	337	37240.3	5.43	Cytoplasm
ML1h07G01339.1	MlANR4	350	37948.2	6.52	Cytoplasm
ML1h03G03510.1	MlTT8.1	641	71812.2	6.05	Nucleus
ML1h02G03012.1	MlTT8.2	657	73615.5	5.18	Nucleus
ML1h01G02333.1	MlTTG1.1	378	41058.5	4.67	Cytoplasm|Nucleus
ML1h04G00438.1	MlTTG1.2	316	34344.3	5.02	Cytoplasm|Nucleus
ML1h08G01785.1	MlC1.1	260	28947.5	7.82	Nucleus
ML1h05G03234.1	MlC1.2	277	30559.2	7.97	Nucleus
ML1h05G01322.1	MlC1.3	278	30511.3	8.27	Nucleus

Table S3c. The information of flavonoid biosynthetic proteins identified in *E. glaucum*

ID	Name	length	MW(Da)	pI	Localizations
Eg01_t040920	EgPAL1	713	77092.6	6.26	Cytoplasm
Eg05_t014880	EgPAL2	710	76801.6	6.51	Cytoplasm|Nucleus
Eg05_t014870	EgPAL3	682	73719.7	6.59	Cytoplasm|Nucleus
Eg02_t018790	EgPAL4	712	77302.3	6.19	Cytoplasm
Eg09_t011330	EgPAL5	709	76467.5	6.15	Cytoplasm
Eg02_t001010	EgPAL6	710	76853.9	6.61	Cytoplasm
Eg09_t017980	EgPAL7	714	76873	6.39	Cytoplasm
Eg05_t024420	EgPAL8	712	76639.9	6.51	Cytoplasm
Eg06_t005550	EgPAL9	758	83171.1	6.04	Cytoplasm
Eg08_t019660	EgC4H1	504	57589.5	9.6	Endoplasmic reticulum
Eg07_t003130	EgC4H2	504	57346.3	9.44	Endoplasmic reticulum
Eg08_t048930	EgC4H3	504	57389.3	9.57	Endoplasmic reticulum
Eg07_t034110	EgC4H4	504	57849.9	8.66	Endoplasmic reticulum

Eg02_t002700	Eg4CL1.1	544	58412	8.53	Peroxisome
Eg05_t023200	Eg4CL1.2	547	59105.8	8.52	Peroxisome
Eg02_t037340	Eg4CL2.1	564	60678.7	5.83	Cytoplasm
Eg09_t018540	Eg4CL2.2	570	61371.5	5.5	Cytoplasm
Eg09_t002410	Eg4CL3	550	59306.1	8.55	Peroxisome
Eg02_t001820	Eg4CL4	550	59197.5	8.94	Peroxisome
Eg05_t033420	Eg4CL5	551	58611.8	6.69	Peroxisome
Eg01_t038400	Eg4CL7	556	59297.9	8.31	Peroxisome
Eg03_t041750	Eg4CL8	545	58062.5	7.3	Peroxisome
Eg04_t006840	Eg4CL9	549	59476.2	6.51	Cytoplasm|Peroxisome
Eg01_t014520	Eg4CL10	549	59490.3	6.43	Cytoplasm
Eg01_t025930	Eg4CL11	549	59318.9	5.15	Cytoplasm
Eg04_t025780	Eg4CL12	544	59003.4	5.81	Cytoplasm
Eg04_t032900	Eg4CL13	544	58907.4	6.15	Cytoplasm
Eg07_t001640	Eg4CL14	563	59753	6.36	Peroxisome
Eg07_t014740	Eg4CL15	544	59315.6	5.29	Cytoplasm
Eg01_t015550	EgCHI1	237	24932.1	4.81	Cytoplasm
Eg09_t018750	EgCHI2	212	23585.9	4.79	Cytoplasm
Eg08_t046640	EgCHS1	393	42788.9	6.94	Cytoplasm
Eg08_t040870	EgCHS2	393	42822.2	6.94	Cytoplasm
Eg08_t020390	EgCHS3	393	42952.2	6.07	Cytoplasm
Eg08_t049390	EgCHS4	393	43183.4	7.31	Cytoplasm
Eg08_t049400	EgCHS5	393	43094.3	7.06	Cytoplasm
Eg08_t049410	EgCHS6	393	43044.2	7.31	Cytoplasm
Eg07_t027880	EgF3H1	374	41398.8	5.35	Cytoplasm

Eg02_t022060	EgF3H2	374	41633.2	5.36	Cytoplasm
Eg03_t018110	EgF3'H1	521	57527.6	7.48	Endoplasmic reticulum
Eg09_t020640	EgF3'5'H1	508	56310.1	8.83	Endoplasmic reticulum
Eg02_t007770	EgF3'5'H2	504	56228.2	9.53	Endoplasmic reticulum
Eg06_t004020	EgF3'5'H3	506	56223.7	7.39	Endoplasmic reticulum
Eg07_t042500	EgF3'5'H4	508	56049.6	8.47	Endoplasmic reticulum
Eg02_t010650	EgF3'5'H5	506	55952.6	7.81	Endoplasmic reticulum
Eg02_t019070	EgF3'5'H6	506	56107.7	7	Endoplasmic reticulum
Eg02_t019100	EgF3'5'H7	341	37198.9	10.07	Endoplasmic reticulum
Eg02_t019110	EgF3'5'H8	209	23498.9	5.33	Cytoplasm
Eg02_t019180	EgF3'5'H9	506	56483.2	8.33	Endoplasmic reticulum
Eg09_t010710	EgF3'5'H10	506	56233.7	8.59	Endoplasmic reticulum
Eg06_t006150	EgFLS1	302	33251.6	6.35	Cytoplasm|Nucleus
Eg01_t007540	EgFLS2	334	37412.3	5.14	Cytoplasm
Eg08_t007020	EgFLS3	336	37815.4	6.19	Cytoplasm|Nucleus
Eg03_t031640	EgFLS4	334	37332.6	6.63	Cytoplasm
Eg09_t036940	EgDFR1	343	38149.3	5.24	Cytoplasm
Eg04_t027830	EgDFR2	360	40467	5.98	Cytoplasm
Eg03_t018610	EgDFR3	299	34129.2	5.42	Cytoplasm
Eg05_t014690	EgLDOX	361	40632.4	5.66	Cytoplasm
Eg08_t008340	EgUGT78D2.1	457	47910.6	6.65	Cytoplasm
Eg06_t008220	EgUGT78D2.2	459	47656.6	6.39	Cytoplasm
Eg01_t005290	EgUGT78D2.3	460	48329.3	6.29	Cytoplasm
Eg08_t003590	EgUGT75C1.1	477	52031.3	5.1	Cytoplasm
Eg08_t037030	EgUGT75C1.2	495	54906.7	5.88	Cytoplasm

Eg08_t037040	EgUGT75C1.3	474	52377.2	5.17	Cytoplasm
Eg07_t037900	EgUGT79B1	472	51214.8	6.67	Cytoplasm
Eg05_t019870	EgLAR	355	38258.2	6.84	Cytoplasm
Eg06_t013840	EgANR1	333	36445.3	5.92	Cytoplasm
Eg02_t015210	EgANR2	343	37129.4	6.07	Cytoplasm
Eg04_t020700	EgTT8.1	651	73039.9	5.52	Nucleus
Eg03_t004200	EgTT8.2	642	71979.5	6.13	Nucleus
Eg05_t009200	EgTTG1.1	385	41845.3	4.67	Cytoplasm|Nucleus
Eg09_t041330	EgTTG1.2	342	37149.2	4.75	Cytoplasm|Nucleus
Eg07_t019620	EgC1.1	261	28848.4	8.59	Nucleus
Eg08_t035970	EgC1.2	279	30806.5	7.97	Nucleus

Table S3d. The information of flavonoid biosynthetic proteins identified in *M. acuminata.*

ID	Name	length	MW(Da)	pI	Localizations
Macma4_01_g04800.1	MaPAL1	713	77111.5	6.15	Cytoplasm
Macma4_05_g03790.1	MaPAL2	710	76888.9	6.68	Cytoplasm|Nucleus
Macma4_11_g15930.1	MaPAL3	821	89696	6.86	Cytoplasm|Nucleus
Macma4_02_g02760.1	MaPAL4	712	76823.8	6	Cytoplasm
Macma4_05_g21700.1	MaPAL5	712	76936.3	6.39	Cytoplasm
Macma4_11_g22170.1	MaPAL6	714	77034.2	6.3	Cytoplasm
Macma4_08_g14800.1	MaPAL7	710	76913.9	6.18	Cytoplasm
Macma4_09_g16190.1	MaPAL8	783	85950.4	6.31	Plastid
Macma4_10_g16840.1	MaC4H1	504	57549.5	9.6	Endoplasmic reticulum
Macma4_07_g20380.1	MaC4H2	504	57454.5	9.77	Endoplasmic reticulum
Macma4_06_g10450.1	MaC4H3	504	57587.5	9.34	Endoplasmic reticulum

Macma4_09_g04800.1	MaC4H4	504	57918.9	8.66	Endoplasmic reticulum
Macma4_08_g13040.1	Ma4CL1.1	544	58411.8	7.94	Peroxisome
Macma4_05_g22890.1	Ma4CL1.2	545	58653.3	7.58	Peroxisome
Macma4_02_g21360.1	Ma4CL2.1	916	100037.3	6.17	Cytoplasm
Macma4_11_g22750.1	Ma4CL2.2	569	61158.2	5.26	Cytoplasm
Macma4_11_g14090.1	Ma4CL3	341	37269.2	9	Peroxisome
Macma4_08_g13960.1	Ma4CL4	553	59587.1	9.25	Peroxisome
Macma4_05_g28920.1	Ma4CL5	555	59148.5	6.73	Peroxisome
Macma4_11_g06130.1	Ma4CL6	576	61756.7	8.84	Peroxisome
Macma4_01_g07350.1	Ma4CL7	556	59390.9	8.31	Peroxisome
Macma4_03_g14310.1	Ma4CL8	545	58542.2	8.03	Peroxisome
Macma4_06_g39580.1	Ma4CL9	543	58654.2	6.2	Cytoplasm
Macma4_01_g19800.1	Ma4CL10	549	59198.7	5	Cytoplasm
Macma4_03_g13930.1	Ma4CL11	656	71400.8	5.86	Endoplasmic reticulum
Macma4_04_g06110.1	Ma4CL12	544	59039.5	5.94	Cytoplasm
Macma4_04_g13130.1	Ma4CL13	544	59221.7	5.79	Cytoplasm
Macma4_07_g05220.1	Ma4CL14	544	58642.1	5.64	Cytoplasm
Macma4_07_g18890.1	Ma4CL15	563	59412.6	6.42	Peroxisome
Macma4_09_g24760.1	Ma4CL16	544	59345.8	5.28	Cytoplasm
Macma4_04_g18950.1	MaCHI1	237	25003.3	4.72	Cytoplasm
Macma4_11_g22930.1	MaCHI2	212	23695	4.72	Cytoplasm
Macma4_06_g12560.1	MaCHS1	434	47100.1	7.78	Cytoplasm
Macma4_06_g18170.1	MaCHS2	416	45664.5	6.98	Cytoplasm
Macma4_10_g16190.1	MaCHS3	393	42939.3	6.61	Cytoplasm
Macma4_06_g10020.1	MaCHS4	407	44537.1	7.27	Cytoplasm

Macma4_06_g10010.1	MaCHS5	424	46536.2	6.86	Cytoplasm
Macma4_10_g16140.1	MaCHS6	393	42888.2	6.79	Cytoplasm
Macma4_07_g17110.1	MaF3H1	374	41382.5	4.92	Cytoplasm
Macma4_02_g06130.1	MaF3H2	373	41427.9	5.11	Cytoplasm
Macma4_03_g33170.1	MaF3'H	542	59767.2	8.01	Endoplasmic reticulum
Macma4_08_g08290.1	MaF3'5'H1	504	56530.4	9.61	Endoplasmic reticulum
Macma4_11_g05110.1	MaF3'5'H2	505	55839.7	9.38	Endoplasmic reticulum
Macma4_08_g05480.1	MaF3'5'H3	506	56055.8	7.85	Endoplasmic reticulum
Macma4_09_g17660.1	MaF3'5'H4	506	56302.8	6.7	Endoplasmic reticulum
Macma4_10_g01240.1	MaF3'5'H5	453	49898.5	9.01	Endoplasmic reticulum
Macma4_10_g01260.1	MaF3'5'H6	453	49897.4	8.83	Endoplasmic reticulum
Macma4_02_g03040.1	MaF3'5'H7	417	45829.5	8.23	Endoplasmic reticulum
Macma4_02_g03240.1	MaF3'5'H8	506	56237	7.97	Endoplasmic reticulum
Macma4_02_g03250.1	MaF3'5'H9	506	56182.9	7.97	Endoplasmic reticulum
Macma4_02_g03260.1	MaF3'5'H10	209	23585.2	6.16	Cytoplasm
Macma4_02_g03290.1	MaF3'5'H11	506	56011.6	7.7	Endoplasmic reticulum
Macma4_02_g03300.1	MaF3'5'H12	573	63705	8.86	Peroxisome
Macma4_11_g15260.1	MaF3'5'H13	506	56128.6	9.25	Endoplasmic reticulum
Macma4_03_g07220.1	MaFLS1	334	37416.5	5.79	Cytoplasm
Macma4_08_g24690.1	MaFLS2	334	37063	5.93	Cytoplasm
Macma4_04_g33220.1	MaDFR1	353	39360.9	6.13	Cytoplasm
Macma4_03_g33910.1	MaDFR2	357	40207.7	4.89	Cytoplasm
Macma4_04_g11180.1	MaDFR3	359	40477.2	6.35	Cytoplasm
Macma4_05_g03920.1	MaLDOX	361	40674.6	5.68	Cytoplasm
Macma4_10_g27580.1	MaUGT78D2.1	457	47869.8	6.99	Cytoplasm

Macma4_07_g16200.1	MaUGT78D2.2	459	47723.6	6.34	Cytoplasm
Macma4_03_g04880.1	MaUGT78D2.3	460	48317.5	6.29	Cytoplasm
Macma4_10_g31950.1	MaUGT75C1.1	479	52486.7	5.31	Cytoplasm
Macma4_06_g07210.1	MaUGT75C1.2	474	52394.4	5.28	Cytoplasm
Macma4_06_g07200.1	MaUGT75C1.3	505	55611.5	5.62	Cytoplasm
Macma4_06_g07190.1	MaUGT75C1.4	470	52105.7	5.1	Cytoplasm
Macma4_09_g07100.1	MaUGT79B1	468	50614.2	7.2	Cytoplasm
Macma4_05_g19560.1	MaLAR	352	37887.7	6.78	Cytoplasm
Macma4_07_g10640.1	MaANR1	339	37253.2	5.64	Cytoplasm
Macma4_07_g10660.1	MaANR2	336	36926	5.62	Cytoplasm
Macma4_07_g10670.1	MaANR3	339	37358.3	5.29	Cytoplasm
Macma4_07_g10680.1	MaANR4	339	37203.2	5.28	Cytoplasm
Macma4_08_g01440.1	MaANR5	352	37982.2	5.76	Cytoplasm
Macma4_06_g25800.1	MaTT8.1	652	72922.9	5.39	Nucleus
Macma4_03_g19880.1	MaTT8.2	642	71624	5.8	Nucleus
Macma4_05_g10420.1	MaTTG1.1	379	41179.7	4.67	Cytoplasm|Nucleus
Macma4_04_g37870.1	MaTTG1.2	357	38819	4.63	Cytoplasm|Nucleus
Macma4_10_g21630.1	MaC1.1	279	30553.2	7.71	Nucleus
Macma4_09_g29590.1	MaC1.2	261	28854.3	7.14	Nucleus

Table S3e. The information of flavonoid biosynthetic proteins identified in *M. balbisiana.*

ID	Name	length	MW(Da)	pI	Localizations
Mba09_g14610.1	MbPAL1	758	83243.3	5.93	Cytoplasm
Mba11_g20430.1	MbPAL2	784	85216.6	6.68	Cytoplasm
Mba08_g14430.1	MbPAL3	710	76854	6.66	Cytoplasm|Nucleus

Mba02_g01510.1	MbPAL5	495	52879.7	7.7	Cytoplasm
Mba11_g14220.1	MbPAL6	683	73969.3	7.92	Cytoplasm|Nucleus
Mba05_g13940.1	MbPAL7	710	76894.9	6.59	Cytoplasm|Nucleus
Mba01_g14280.1	MbPAL8	713	77137.6	6.36	Cytoplasm
Mba09_g04380.1	MbC4H1	504	57779.6	7.96	Endoplasmic reticulum
Mba10_g11450.1	MbC4H2	470	53564	10.19	Endoplasmic reticulum
Mba06_g09560.1	MbC4H3	504	57550.5	9.34	Endoplasmic reticulum
Mba07_g18380.1	MbC4H4	504	57433.5	9.74	Endoplasmic reticulum
Mba05_g21540.1	Mb4CL1.1	545	58665.3	7.6	Peroxisome
Mba08_g12720.1	Mb4CL1.2	479	51433	9.2	Peroxisome
Mba02_g18560.1	Mb4CL2.1	916	99925.1	6.31	Cytoplasm
Mba11_g20940.1	Mb4CL2.2	569	61081.1	5.49	Cytoplasm
Mba11_g05710.1	Mb4CL3.1	1028	110546.8	8.85	Lysosome/Vacuole
Mba11_g12600.1	Mb4CL3.2	282	30353	8.85	Cytoplasm|Nucleus
Mba08_g13620.1	Mb4CL4	638	69166.8	9.39	Plastid
Mba05_g27160.1	Mb4CL5	551	58825	6.73	Peroxisome
Mba01_g16650.1	Mb4CL7	289	31830.6	9.78	Peroxisome
Mba03_g13490.1	Mb4CL8	590	63467.2	8.48	Peroxisome
Mba06_g36630.1	Mb4CL9	543	58869.5	6.07	Cytoplasm
Mba01_g28370.1	Mb4CL10	549	59225.7	5.07	Cytoplasm
Mba04_g05720.1	Mb4CL11	544	59041.4	6.21	Cytoplasm
Mba04_g12340.1	Mb4CL12	544	59147.6	5.79	Cytoplasm
Mba07_g04890.1	Mb4CL13	512	55129.1	4.98	Cytoplasm
Mba07_g17040.1	Mb4CL14	563	59524.8	6.61	Peroxisome
Mba11_g21110.1	MbCHI1	210	23524.8	4.72	Cytoplasm

Mba04_g18040.1	MbCHI2	205	21682.7	6.02	Cytoplasm
Mba06_g11590.1	MbCHS1	393	42806	6.72	Cytoplasm
Mba06_g16830.1	MbCHS2	393	42950.2	6.72	Cytoplasm
Mba10_g10880.1	MbCHS3	399	43644	6.52	Cytoplasm
Mba06_g09110.1	MbCHS4	270	29676.9	10.08	Cytoplasm
Mba06_g09080.1	MbCHS5	339	37144.6	6.29	Cytoplasm
Mba06_g09090.1	MbCHS6	318	34448.5	6.17	Cytoplasm
Mba06_g09120.1	MbCHS7	342	37079.5	6.11	Cytoplasm
Mba10_g10820.1	MbCHS8	393	42860.1	6.6	Cytoplasm
Mba02_g04670.1	MbF3H1	373	41366.9	5.22	Cytoplasm
Mba07_g15320.1	MbF3H2	374	41371.6	4.92	Cytoplasm
Mba01_g02030.1	MbF3'H	542	59798.1	7.08	Endoplasmic reticulum
Mba08_g07950.1	MbF3'5'H1	504	56368.2	9.37	Endoplasmic reticulum
Mba11_g04840.1	MbF3'5'H2	864	94437.6	8.97	Endoplasmic reticulum
Mba09_g16030.1	MbF3'5'H3	506	56216.6	6.7	Endoplasmic reticulum
Mba10_g00740.1	MbF3'5'H4	457	50366	8.83	Endoplasmic reticulum
Mba02_g01840.1	MbF3'5'H5	506	56169.8	7.68	Endoplasmic reticulum
Mba02_g01880.1	MbF3'5'H6	323	35617.3	10.17	Endoplasmic reticulum
Mba02_g01890.1	MbF3'5'H7	506	56224.9	8.04	Endoplasmic reticulum
Mba02_g01900.1	MbF3'5'H8	506	56288.1	8.24	Endoplasmic reticulum
Mba02_g01910.1	MbF3'5'H9	506	56017.8	9.09	Endoplasmic reticulum
Mba02_g01920.1	MbF3'5'H10	561	62396	7.91	Endoplasmic reticulum
Mba02_g01940.1	MbF3'5'H11	209	23404	6.51	Cytoplasm
Mba02_g01950.1	MbF3'5'H12	1217	134294	9.9	Endoplasmic reticulum
Mba11_g13650.1	MbF3'5'H13	506	56182.6	9.06	Endoplasmic reticulum

Mba08_g23630.1	MbFLS1	302	33559.2	6.26	Cytoplasm|Nucleus
Mba10_g22000.1	MbFLS2	305	33869	5.83	Cytoplasm|Nucleus
Mba10_g21980.1	MbFLS3	336	37799.4	6.35	Cytoplasm
Mba03_g06940.1	MbFLS4	334	37542.6	5.44	Cytoplasm
Mba09_g14060.1	MbFLS5	336	37001.8	5.45	Cytoplasm
Mba04_g31800.1	MbDFR1	322	36000.2	6.24	Cytoplasm
Mba01_g01310.1	MbDFR2	357	40174.7	4.97	Cytoplasm
Mba05_g13780.1	MbLDOX	413	45918.6	5.66	Cytoplasm
Mba03_g04830.1	MbUGT78D2.1	499	52468.3	6.24	Cytoplasm
Mba10_g20800.1	MbUGT78D2.2	456	47887.8	7.47	Cytoplasm
Mba07_g14580.1	MbUGT78D2.3	459	47791.9	6.45	Cytoplasm
Mba10_g24560.1	MbUGT75C1.1	480	52673.9	5.45	Cytoplasm
Mba06_g06480.1	MbUGT75C1.2	470	51986.6	5.08	Cytoplasm
Mba06_g06490.1	MbUGT75C1.3	477	52963.9	5.12	Cytoplasm
Mba05_g18490.1	MbLAR	355	38226.2	6.83	Cytoplasm
Mba08_g01400.1	MbANR	352	38139.4	5.77	Cytoplasm
Mba06_g23950.1	MbTT8.1	197	21951.2	8.92	Cytoplasm|Nucleus
Mba03_g18730.1	MbTT8.2	173	19324.2	8.47	Nucleus
Mba05_g07670.1	MbTTG1.1	402	44000	4.77	Cytoplasm|Nucleus
Mba04_g36060.1	MbTTG1.2	383	41633.2	4.77	Cytoplasm|Nucleus
Mba10_g15520.1	MbC1.1	279	30582.2	7.18	Nucleus
Mba06_g05630.1	MbC1.2	276	30433	8.23	Nucleus
Mba09_g23680.1	MbC1.3	228	24926.7	4.68	Nucleus

Table S3f. The information of flavonoid biosynthetic proteins identified in *M. schizocharpa*

ID	Name	length	MW(Da)	pI	Localizations
Ms01t004140.1	MsPAL1	713	77111.6	6.26	Cytoplasm
Ms05t120010.1	MsPAL2	710	76874.9	6.68	Cytoplasm|Nucleus
Ms11t313180.1	MsPAL3	644	69769	6.36	Cytoplasm|Nucleus
Ms02t024050.1	MsPAL4	712	76767.6	5.77	Cytoplasm
Ms08t220210.1	MsPAL5	710	76899.1	6.56	Cytoplasm
Ms11t319150.1	MsPAL6	714	77060.2	6.39	Cytoplasm
Ms05t135820.1	MsPAL7	712	76757.1	6.47	Cytoplasm
Ms09t254450.1	MsPAL8	758	83149.1	5.7	Cytoplasm
Ms10t281630.1	MsC4H1	505	57631.6	9.49	Endoplasmic reticulum
Ms07t197940.1	MsC4H2	504	57465.5	9.75	Endoplasmic reticulum
Ms09t244050.1	MsC4H3	504	57960.9	8.66	Endoplasmic reticulum
Ms08t218760.1	Ms4CL1.1	544	58385.8	7.63	Peroxisome
Ms05t136800.1	Ms4CL1.2	545	58688.2	7.59	Peroxisome
Ms02t041000.1	Ms4CL2.1	540	58326.1	6.46	Cytoplasm
Ms11t319670.1	Ms4CL2.2	569	61167.2	5.37	Cytoplasm
Ms11t311430.1	Ms4CL3	341	37481.3	9.29	Peroxisome
Ms08t219500.1	Ms4CL4	550	59269.7	9.26	Peroxisome
Ms05t142250.1	Ms4CL5	587	62546.4	7.36	Peroxisome
Ms11t304430.1	Ms4CL6	559	61313.7	9.28	Peroxisome
Ms01t006680.1	Ms4CL7	572	61161.8	7.4	Peroxisome
Ms03t059300.1	Ms4CL8	528	56948.5	8.66	Peroxisome
Ms06t178240.1	Ms4CL9	543	58759.4	6.04	Cytoplasm

Ms01t017810.1	Ms4CL10	549	59239.8	5.16	Cytoplasm
Ms03t058920.1	Ms4CL11	549	59491.1	5.27	Cytoplasm
Ms04t083350.1	Ms4CL12	544	59032.5	6.07	Cytoplasm
Ms04t089570.1	Ms4CL13	544	59155.6	5.51	Cytoplasm
Ms07t183930.1	Ms4CL14	544	58730.2	5.5	Cytoplasm
Ms07t196560.1	Ms4CL15	512	53831.1	6.12	Cytoplasm
Ms09t262230.1	Ms4CL16	544	59429	5.28	Cytoplasm
Ms11t319860.1	MsCHI1	288	32488.4	6.81	Plastid
Ms04t093250.1	MsCHI2	237	25028.4	5.12	Cytoplasm
Ms10t280970.1	MsCHS1	393	42878.1	6.6	Cytoplasm
Ms06t158650.1	MsCHS2	392	42857.1	6.71	Cytoplasm
MsSC51t327370.1	MsCHS3	369	40956.8	6.91	Cytoplasm
MsSC51t327380.1	MsCHS4	410	44989.7	7.93	Cytoplasm
MsSC66t327440.1	MsCHS5	389	41971.8	9.06	Cytoplasm
Ms02t027200.1	MsF3H	373	41440.9	5.1	Cytoplasm
Ms03t075990.1	MsF3'H	523	57540.6	7.28	Endoplasmic reticulum
Ms08t214430.1	MsF3'5'H1	445	49617.4	9.02	Endoplasmic reticulum
Ms09t255750.1	MsF3'5'H2	474	52543.3	6.11	Endoplasmic reticulum
Ms08t211840.1	MsF3'5'H3	506	56115.9	8	Endoplasmic reticulum
Ms02t024370.1	MsF3'5'H4	321	36104.3	5.81	Plastid
Ms02t024400.1	MsF3'5'H5	506	55805.4	8.47	Endoplasmic reticulum
Ms02t024410.1	MsF3'5'H6	421	46389.7	8.65	Endoplasmic reticulum
Ms02t024420.1	MsF3'5'H7	476	52619.6	9.26	Endoplasmic reticulum
Ms11t312500.1	MsF3'5'H8	506	56176.6	9.06	Endoplasmic reticulum
Ms08t229420.1	MsFLS1	334	37144.2	5.71	Cytoplasm

Ms03t052640.1	MsFLS2	334	37487.6	5.61	Cytoplasm
Ms10t292840.1	MsFLS3	336	37491	6.45	Cytoplasm|Nucleus
Ms04t107810.1	MsDFR1	353	39425.1	6.18	Cytoplasm
Ms04t087800.1	MsDFR2	354	39767.3	6.14	Cytoplasm
Ms03t076720.1	MsDFR3	357	40143.6	4.89	Cytoplasm
Ms05t120140.1	MsLDOX	361	40674.6	5.68	Cytoplasm
Ms10t291590.1	MsUGT78D2.1	457	48126.1	6.99	Cytoplasm
Ms07t193970.1	MsUGT78D2.2	455	47311	6.23	Cytoplasm
Ms03t050760.1	MsUGT78D2.3	460	48360.4	5.97	Cytoplasm
Ms06t152710.1	MsUGT75C1.1	470	52002.5	5.09	Cytoplasm
Ms06t152720.1	MsUGT75C1.2	470	51872.5	5.37	Cytoplasm
Ms06t152730.1	MsUGT75C1.3	474	52482.5	5.51	Cytoplasm
Ms10t295740.1	MsUGT75C1.4	471	51659.9	5.55	Cytoplasm
Ms09t246270.1	MsUGT79B1	471	51074.6	6.67	Cytoplasm
Ms05t133790.1	MsLAR	325	35058.6	6.62	Cytoplasm
Ms08t207990.1	MsANR1	354	38292.7	7.42	Cytoplasm
Ms07t188920.1	MsANR2	351	38307.5	5.61	Cytoplasm
Ms07t188910.1	MsANR3	339	37321.2	5.15	Cytoplasm
MsSC186t326890.1	MsTT8.1	385	42678.3	4.39	Cytoplasm|Nucleus
Ms03t064490.1	MsTT8.2	565	62784.4	6.42	Nucleus
Ms04t112120.1	MsTTG1.1	341	36987.1	4.75	Cytoplasm|Nucleus
Ms05t125970.1	MsTTG1.2	356	38547.9	4.63	Cytoplasm|Nucleus
Ms09t266370.1	MsC1.1	261	29000.5	8.07	Nucleus
Ms06t151760.1	MsC1.2	276	30410	8.02	Nucleus

Table S4a. Divergence time, Ka, Ks and Ka/Ks values of orthologous flavonoid biosynthetic genes pairs between *E. glaucum* and *M. acuminata*

Seq_1	Name1	Seq_2	Name2	Ka	Ks	Ka_Ks	Diversity_year
Eg02_t002700	Eg4CL1	Parent=Macma4_08_g13040	Ma4CL1	0.030448156	0.087782748	0.346858085	19.50727735
Eg05_t023200	Eg4CL1.2	Parent=Macma4_05_g22890	Ma4CL1.2	0.028867818	0.102914267	0.280503555	22.86983716
Eg01_t014520	Eg4CL10	Parent=Macma4_01_g19800	Ma4CL10	0.050934779	0.351209955	0.145026581	78.04665669
Eg01_t014520	Eg4CL10	Parent=Macma4_03_g13930	Ma4CL11	0.022718058	0.088092637	0.257888276	19.57614153
Eg01_t025930	Eg4CL11	Parent=Macma4_01_g19800	Ma4CL10	0.012306286	0.089448569	0.137579456	19.87745987
Eg01_t025930	Eg4CL11	Parent=Macma4_03_g13930	Ma4CL11	0.04613291	0.361629763	0.127569452	80.36216945
Eg01_t025930	Eg4CL11	Parent=Macma4_07_g05220	Ma4CL14	0.083495624	0.454381606	0.183756612	100.9736902
Eg04_t025780	Eg4CL12	Parent=Macma4_04_g13130	Ma4CL13	0.013220751	0.08282486	0.159622984	18.40552448
Eg04_t025780	Eg4CL12	Parent=Macma4_04_g06110	Ma4CL12	0.042717676	0.361825668	0.118061485	80.40570393
Eg04_t025780	Eg4CL12	Parent=Macma4_09_g24760	Ma4CL16	0.057442296	0.416504423	0.137915213	92.55653853
Eg04_t032900	Eg4CL13	Parent=Macma4_04_g06110	Ma4CL12	0.015828425	0.091971342	0.172101707	20.43807607
Eg04_t032900	Eg4CL13	Parent=Macma4_04_g13130	Ma4CL13	0.049643453	0.336560674	0.147502239	74.79126088
Eg07_t001640	Eg4CL14	Parent=Macma4_07_g18890	Ma4CL15	0.033652834	0.109779242	0.30655007	24.39538711
Eg07_t014740	Eg4CL15	Parent=Macma4_09_g24760	Ma4CL16	0.020341637	0.095221058	0.213625405	21.16023509
Eg09_t018540	Eg4CL2.2	Parent=Macma4_11_g22750	Ma4CL2.2	0.011830676	0.063646732	0.185880335	14.14371818
Eg09_t002410	Eg4CL3	Parent=Macma4_11_g06130	Ma4CL6	0.046396851	0.103003864	0.450437966	22.88974756
Eg02_t001820	Eg4CL4	Parent=Macma4_08_g13960	Ma4CL4	0.014301169	0.092773033	0.154152224	20.6162295
Eg05_t033420	Eg4CL5	Parent=Macma4_05_g28920	Ma4CL5	0.021000952	0.096247208	0.218198039	21.38826854
Eg01_t038400	Eg4CL7	Parent=Macma4_01_g07350	Ma4CL7	0.022725881	0.120256988	0.188977637	26.72377501
Eg03_t041750	Eg4CL8	Parent=Macma4_03_g14310	Ma4CL8	0.033998936	0.107672474	0.315762565	23.92721635
Eg04_t006840	Eg4CL9	Parent=Macma4_06_g39580	Ma4CL9	0.024011986	0.076482549	0.31395379	16.9961219
Eg06_t013840	EgANR1	Parent=Macma4_07_g10660	MaANR2	0.051307976	0.11652155	0.440330357	25.89367784

Eg02_t015210	EgANR2	Parent=Macma4_08_g01440	MaANR5	0.029759249	0.067715135	0.439477065	15.04780778
Eg07_t019620	EgC1	Parent=Macma4_09_g29590	MaC1.2	0.047662584	0.095228596	0.500507049	21.16191029
Eg08_t019660	EgC4H1	Parent=Macma4_06_g10450	MaC4H3	0.033843676	0.295141444	0.114669344	65.58698754
Eg08_t019660	EgC4H1	Parent=Macma4_10_g16840	MaC4H1	0.007887314	0.075517183	0.104443969	16.78159633
Eg07_t003130	EgC4H2	Parent=Macma4_07_g20380	MaC4H2	0.009631654	0.088445917	0.108898794	19.65464829
Eg08_t048930	EgC4H3	Parent=Macma4_06_g10450	MaC4H3	0.012307969	0.084679048	0.145348452	18.81756618
Eg08_t048930	EgC4H3	Parent=Macma4_10_g16840	MaC4H1	0.038660467	0.355247937	0.108826718	78.94398603
Eg07_t034110	EgC4H4	Parent=Macma4_09_g04800	MaC4H4	0.01400274	0.086319142	0.162220571	19.18203146
Eg01_t015550	EgCHI1	Parent=Macma4_04_g18950	MaCHI1	0.052121717	0.066655272	0.781959408	14.81228265
Eg09_t018750	EgCHI2	Parent=Macma4_11_g22930	MaCHI2	0.01648418	0.105436463	0.156342309	23.4303251
Eg08_t046640	EgCHS1	Parent=Macma4_06_g12560	MaCHS1	0.029252417	0.075016776	0.389945011	16.67039458
Eg08_t040870	EgCHS2	Parent=Macma4_06_g18170	MaCHS2	0.017616114	0.060086713	0.293178197	13.35260298
Eg08_t040870	EgCHS2	Parent=Macma4_06_g10010	MaCHS5	0.106008972	0.350319314	0.302606701	77.84873651
Eg08_t040870	EgCHS2	Parent=Macma4_10_g16140	MaCHS6	0.032099655	0.329646032	0.097376131	73.25467381
Eg08_t020390	EgCHS3	Parent=Macma4_06_g18170	MaCHS2	0.041153619	0.29886016	0.137701924	66.41336893
Eg08_t020390	EgCHS3	Parent=Macma4_10_g16140	MaCHS6	0.011301777	0.069946424	0.161577628	15.5436497
Eg08_t049400	EgCHS5	Parent=Macma4_06_g10010	MaCHS5	0.028067422	0.079020387	0.35519216	17.56008594
Eg08_t049400	EgCHS5	Parent=Macma4_06_g18170	MaCHS2	0.102873973	0.357535564	0.287730743	79.45234746
Eg08_t049400	EgCHS5	Parent=Macma4_10_g16140	MaCHS6	0.107293231	0.373124864	0.287553153	82.91663637
Eg09_t036940	EgDFR1	Parent=Macma4_04_g33220	MaDFR1	0.020420277	0.106454841	0.191821023	23.65663143
Eg04_t027830	EgDFR2	Parent=Macma4_04_g11180	MaDFR3	0.023229241	0.068816148	0.337555086	15.29247727
Eg09_t020640	EgF3'5'H1	Parent=Macma4_11_g05110	MaF3'5'H2	0.04171204	0.104813195	0.397965545	23.29182113
Eg09_t010710	EgF3'5'H10	Parent=Macma4_11_g15260	MaF3'5'H13	0.020904177	0.074203628	0.281713683	16.48969508
Eg02_t007770	EgF3'5'H2	Parent=Macma4_08_g08290	MaF3'5'H1	0.019630833	0.097024186	0.202329272	21.5609302
Eg06_t004020	EgF3'5'H3	Parent=Macma4_09_g17660	MaF3'5'H4	0.023059979	0.113561102	0.203062306	25.23580042

Eg07_t042500	EgF3'5'H4	Parent=Macma4_09_g17660	MaF3'5'H4	0.124106248	0.464327144	0.267281915	103.1838097
Eg07_t042500	EgF3'5'H4	Parent=Macma4_10_g01260	MaF3'5'H6	0.020876099	0.10088637	0.206926854	22.41919342
Eg02_t010650	EgF3'5'H5	Parent=Macma4_08_g05480	MaF3'5'H3	0.02504705	0.072077584	0.347501246	16.01724098
Eg02_t019070	EgF3'5'H6	Parent=Macma4_02_g03040	MaF3'5'H7	0.050319405	0.165735594	0.303612543	36.83013189
Eg02_t019070	EgF3'5'H6	Parent=Macma4_02_g03290	MaF3'5'H11	0.03908655	0.17604095	0.222031012	39.12021122
Eg02_t019070	EgF3'5'H6	Parent=Macma4_02_g03300	MaF3'5'H12	0.17390456	0.592788456	0.293366982	131.7307679
Eg02_t019110	EgF3'5'H8	Parent=Macma4_02_g03260	MaF3'5'H10	0.019112696	0.125522379	0.152265246	27.89386197
Eg07_t027880	EgF3H1	Parent=Macma4_07_g17110	MaF3H1	0.021999199	0.151758777	0.144961625	33.72417276
Eg03_t018110	EgF3'H1	Parent=Macma4_03_g33170	MaF3'H	0.021851509	0.07919438	0.275922472	17.59875122
Eg02_t022060	EgF3H2	Parent=Macma4_02_g06130	MaF3H2	0.012978399	0.089385328	0.145196076	19.86340612
Eg01_t007540	EgFLS2	Parent=Macma4_03_g07220	MaFLS1	0.017187047	0.090779349	0.189327719	20.17318876
Eg03_t031640	EgFLS4	Parent=Macma4_08_g24690	MaFLS2	0.022583065	0.071429408	0.316159215	15.87320167
Eg05_t019870	EgLAR	Parent=Macma4_05_g19560	MaLAR	0.023227663	0.096131395	0.241624111	21.36253224
Eg05_t014690	EgLDOX	Parent=Macma4_05_g03920	MaLDOX	0.020728814	0.067319162	0.307918476	14.95981384
Eg01_t040920	EgPAL1	Parent=Macma4_01_g04800	MaPAL1	0.012837552	0.079218668	0.162052104	17.60414851
Eg05_t014880	EgPAL2	Parent=Macma4_05_g03790	MaPAL2	0.010621858	0.088488942	0.120035996	19.66420934
Eg02_t018790	EgPAL4	Parent=Macma4_02_g02760	MaPAL4	0.021509118	0.105258317	0.204346017	23.39073706
Eg02_t018790	EgPAL4	Parent=Macma4_08_g14800	MaPAL7	0.058940904	0.401943438	0.146639796	89.3207639
Eg09_t011330	EgPAL5	Parent=Macma4_02_g02760	MaPAL4	0.066969936	0.380667201	0.175927782	84.5927114
Eg09_t011330	EgPAL5	Parent=Macma4_08_g14800	MaPAL7	0.081008797	0.385794925	0.209978907	85.73220561
Eg09_t011330	EgPAL5	Parent=Macma4_11_g15930	MaPAL3	0.02180223	0.081532772	0.267404497	18.11839375
Eg02_t001010	EgPAL6	Parent=Macma4_02_g02760	MaPAL4	0.066617058	0.381236394	0.174739502	84.71919863
Eg02_t001010	EgPAL6	Parent=Macma4_08_g14800	MaPAL7	0.014431063	0.058203717	0.247940577	12.93415936
Eg09_t017980	EgPAL7	Parent=Macma4_05_g21700	MaPAL5	0.043020243	0.376043694	0.114402247	83.56526526
Eg09_t017980	EgPAL7	Parent=Macma4_11_g22170	MaPAL6	0.010017366	0.085679392	0.116916866	19.03986485

Eg05_t024420	EgPAL8	Parent=Macma4_05_g21700	MaPAL5	0.013827449	0.063289053	0.218480891	14.06423403
Eg06_t005550	EgPAL9	Parent=Macma4_09_g16190	MaPAL8	0.008264953	0.062411775	0.132426182	13.86928325
Eg04_t020700	EgTT8	Parent=Macma4_06_g25800	MaTT8	0.024548516	0.092329963	0.265878109	20.51776961
Eg03_t004200	EgTT8.2	Parent=Macma4_03_g19880	MaTT8.2	0.03276383	0.132223082	0.247792063	29.38290703
Eg05_t009200	EgTTG1	Parent=Macma4_05_g10420	MaTTG1	0.016593269	0.098783089	0.167976822	21.95179757
Eg09_t041330	EgTTG1.2	Parent=Macma4_04_g37870	MaTTG1.2	0.015821285	0.124125967	0.127461528	27.58354822
Eg08_t037030	EgUGT75C1.2	Parent=Macma4_06_g07200	MaUGT75C1.3	0.07564968	0.159282386	0.474940653	35.39608579
Eg08_t037040	EgUGT75C1.3	Parent=Macma4_06_g07210	MaUGT75C1.2	0.039690535	0.080912142	0.490538682	17.98047597
Eg08_t008340	EgUGT78D2	Parent=Macma4_10_g27580	MaUGT78D2	0.046476545	0.127836889	0.363561295	28.40819753
Eg06_t008220	EgUGT78D2.2	Parent=Macma4_07_g16200	MaUGT78D2.2	0.02597446	0.086340581	0.300837211	19.18679588
Eg01_t005290	EgUGT78D2.3	Parent=Macma4_03_g04880	MaUGT78D2.3	0.03218191	0.100593663	0.319919856	22.35414734
Eg07_t037900	EgUGT79B1	Parent=Macma4_09_g07100	MaUGT79B1	0.029682758	0.075957716	0.390780025	16.87949236

Table S4b. Divergence time, Ka, Ks and Ka/Ks values of orthologous flavonoid biosynthetic genes pairs between *E. glaucum* and *M. balbisiana*

Seq_1	Name1	Seq_2	Name2	Ka	Ks	Ka_Ks	Diversity_year
Eg02_t002700	Eg4CL1	Mba08_g12720	Mb4CL1.2	0.027861033	0.097001086	0.287223923	21.55579698
Eg05_t023200	Eg4CL1.2	Mba05_g21540	Mb4CL1	0.030607937	0.102579665	0.298382108	22.79548109
Eg01_t014520	Eg4CL10	Mba01_g28370	Mb4CL10	0.051097917	0.361717414	0.141264741	80.38164766
Eg01_t025930	Eg4CL11	Mba01_g28370	Mb4CL10	0.011483478	0.105799832	0.108539661	23.51107385
Eg04_t025780	Eg4CL12	Mba04_g12340	Mb4CL12	0.014885748	0.096718544	0.153907901	21.49300975
Eg04_t025780	Eg4CL12	Mba04_g05720	Mb4CL11	0.04620431	0.35410953	0.130480279	78.69100661
Eg04_t032900	Eg4CL13	Mba04_g05720	Mb4CL11	0.020048528	0.075854067	0.264303926	16.85645937
Eg04_t032900	Eg4CL13	Mba04_g12340	Mb4CL12	0.051385593	0.356223906	0.144250828	79.16086793
Eg07_t001640	Eg4CL14	Mba07_g17040	Mb4CL14	0.033655165	0.119994493	0.28047258	26.66544294
Eg07_t014740	Eg4CL15	Mba04_g05720	Mb4CL11	0.06433892	0.57664255	0.111575048	128.1427888

Eg09_t018540	Eg4CL2.2	Mba11_g20940	Mb4CL2.2	0.014224053	0.05596434	0.254162801	12.43651995
Eg02_t001820	Eg4CL4	Mba08_g13620	Mb4CL4	0.020965115	0.113939276	0.184002532	25.31983903
Eg05_t033420	Eg4CL5	Mba05_g27160	Mb4CL5	0.02442664	0.085944481	0.284214172	19.09877361
Eg01_t038400	Eg4CL7	Mba01_g16650	Mb4CL7	0.011060108	0.075662594	0.1461767	16.81390977
Eg03_t041750	Eg4CL8	Mba03_g13490	Mb4CL8	0.054629005	0.126001261	0.433559192	28.00028025
Eg04_t006840	Eg4CL9	Mba06_g36630	Mb4CL9	0.025671841	0.071168367	0.360719826	15.81519269
Eg02_t015210	EgANR2	Mba08_g01400	MbANR	0.027041839	0.063606107	0.425145322	14.13469045
Eg07_t019620	EgC1	Mba09_g23680	MbC1.3	0.181505951	0.262138392	0.692405068	58.25297596
Eg08_t035970	EgC1.2	Mba06_g05630	MbC1.2	0.037411197	0.13693676	0.273200544	30.43039116
Eg07_t003130	EgC4H2	Mba07_g18380	MbC4H4	0.006991021	0.09164931	0.076280123	20.36651334
Eg08_t048930	EgC4H3	Mba06_g09560	MbC4H3	0.011431305	0.096885056	0.117988321	21.53001244
Eg07_t034110	EgC4H4	Mba09_g04380	MbC4H1	0.023783555	0.07691463	0.30922017	17.09213992
Eg01_t015550	EgCHI1	Mba04_g18040	MbCHI2	0.075505527	0.113254769	0.666687394	25.16772652
Eg09_t018750	EgCHI2	Mba11_g21110	MbCHI1	0.018750977	0.106507748	0.1760527	23.6683885
Eg08_t046640	EgCHS1	Mba06_g11590	MbCHS1	0.024013485	0.057842565	0.415152495	12.85390337
Eg08_t040870	EgCHS2	Mba06_g16830	MbCHS2	0.010176355	0.073520709	0.138414811	16.33793541
Eg08_t040870	EgCHS2	Mba10_g10820	MbCHS8	0.028602131	0.334742724	0.085445118	74.38727194
Eg08_t020390	EgCHS3	Mba06_g16830	MbCHS2	0.033281421	0.318481924	0.104500189	70.77376093
Eg08_t020390	EgCHS3	Mba10_g10820	MbCHS8	0.011304971	0.085461979	0.132280713	18.99155085
Eg08_t049400	EgCHS5	Mba06_g09120	MbCHS7	0.020097217	0.100039483	0.20089285	22.23099614
Eg08_t049400	EgCHS5	Mba06_g16830	MbCHS2	0.095795195	0.366288822	0.261529124	81.39751606
Eg08_t049400	EgCHS5	Mba10_g10820	MbCHS8	0.103446314	0.378471643	0.273326458	84.10480954
Eg09_t036940	EgDFR1	Mba04_g31800	MbDFR1	0.017625938	0.103740921	0.169903431	23.05353792
Eg09_t020640	EgF3'5'H1	Mba11_g04840	MbF3'5'H2	0.040891344	0.114974818	0.355654782	25.54995961
Eg09_t010710	EgF3'5'H10	Mba11_g13650	MbF3'5'H13	0.017749682	0.08463644	0.209716785	18.80809786

Eg02_t007770	EgF3'5'H2	Mba08_g07950	MbF3'5'H1	0.015117222	0.088065101	0.171659618	19.57002254
Eg06_t004020	EgF3'5'H3	Mba09_g16030	MbF3'5'H3	0.024002048	0.119205831	0.201349616	26.49018469
Eg07_t042500	EgF3'5'H4	Mba10_g00740	MbF3'5'H4	0.023234681	0.085363352	0.272185664	18.96963374
Eg02_t019070	EgF3'5'H6	Mba02_g01950	MbF3'5'H12	0.094948398	0.521089619	0.182211263	115.7976931
Eg02_t019110	EgF3'5'H8	Mba02_g01940	MbF3'5'H11	0.0420671	0.121290554	0.346829152	26.95345645
Eg07_t027880	EgF3H1	Mba07_g15320	MbF3H2	0.021391824	0.168182034	0.127194469	37.37378523
Eg03_t018110	EgF3'H1	Mba01_g02030	MbF3'H	0.022735638	0.079229271	0.286960076	17.60650473
Eg02_t022060	EgF3H2	Mba02_g04670	MbF3H1	0.012975847	0.072369457	0.179300047	16.08210161
Eg06_t006150	EgFLS1	Mba09_g14060	MbFLS5	0.141119552	0.204327392	0.690654106	45.40608714
Eg01_t007540	EgFLS2	Mba03_g06940	MbFLS4	0.017871778	0.078576468	0.227444401	17.46143742
Eg01_t007540	EgFLS2	Mba09_g14060	MbFLS5	0.152797668	0.501688536	0.304566792	111.4863413
Eg08_t007020	EgFLS3	Mba10_g21980	MbFLS3	0.025120779	0.094755958	0.26511029	21.05687949
Eg03_t031640	EgFLS4	Mba08_g23630	MbFLS1	0.016049245	0.11824346	0.135730506	26.27632435
Eg05_t019870	EgLAR	Mba05_g18490	MbLAR	0.030210936	0.080750184	0.374128379	17.94448542
Eg05_t014690	EgLDOX	Mba05_g13780	MbLDOX	0.026947173	0.085016608	0.316963632	18.89257956
Eg01_t040920	EgPAL1	Mba01_g14280	MbPAL8	0.011264879	0.073850538	0.152536185	16.41123068
Eg05_t014880	EgPAL2	Mba05_g13940	MbPAL7	0.015037313	0.09511955	0.158088559	21.13767777
Eg02_t018790	EgPAL4	Mba02_g01510	MbPAL5	0.052573992	0.130658518	0.402377077	29.03522614
Eg02_t018790	EgPAL4	Mba08_g14430	MbPAL3	0.058947294	0.40177258	0.146718061	89.28279545
Eg09_t011330	EgPAL5	Mba02_g01510	MbPAL5	0.089161023	0.383754621	0.232338631	85.27880467
Eg09_t011330	EgPAL5	Mba11_g14220	MbPAL6	0.030510176	0.104883377	0.2908962	23.30741718
Eg02_t001010	EgPAL6	Mba08_g14430	MbPAL3	0.014432573	0.058184244	0.248049496	12.92983211
Eg09_t017980	EgPAL7	Mba11_g20430	MbPAL2	0.011277819	0.083599292	0.134903286	18.57762038
Eg06_t005550	EgPAL9	Mba09_g14610	MbPAL1	0.008859243	0.070097065	0.126385359	15.57712554
Eg04_t020700	EgTT8	Mba06_g23950	MbTT8	0.082164693	0.167282093	0.491174466	37.17379843

Eg03_t004200	EgTT8.2	Mba03_g18730	MbTT8.2	0.102720217	0.160248606	0.641005374	35.61080123
Eg03_t004200	EgTT8.2	Mba06_g23950	MbTT8	0.106584956	0.417975642	0.255002794	92.88347593
Eg05_t009200	EgTTG1	Mba05_g07670	MbTTG1	0.018999631	0.106847011	0.177820891	23.74378025
Eg09_t041330	EgTTG1.2	Mba04_g36060	MbTTG1.2	0.015776217	0.12833498	0.122929984	28.51888451
Eg08_t037030	EgUGT75C1.2	Mba06_g06480	MbUGT75C1.2	0.063079795	0.113481846	0.555858032	25.21818792
Eg08_t037040	EgUGT75C1.3	Mba06_g06490	MbUGT75C1.3	0.038006966	0.064169735	0.592288025	14.25994113
Eg08_t008340	EgUGT78D2	Mba10_g20800	MbUGT78D2.2	0.037957164	0.098576801	0.385051686	21.90595588
Eg06_t008220	EgUGT78D2.2	Mba07_g14580	MbUGT78D2.3	0.02493788	0.074006256	0.336969898	16.4458346
Eg01_t005290	EgUGT78D2.3	Mba03_g04830	MbUGT78D2	0.02910064	0.110207788	0.264052485	24.49061962

Table S4c. Divergence time, Ka, Ks and Ka/Ks values of orthologous flavonoid biosynthetic genes pairs between *E. glaucum* and *M. schizocharpa*

Seq_1	Name1	Seq_2	Name2	Ka	Ks	Ka_Ks	Diversity_year
Eg02_t002700	Eg4CL1	Ms08t218760	Ms4CL1	0.029593807	0.093045064	0.318058856	20.67668097
Eg05_t023200	Eg4CL1.2	Ms05t136800	Ms4CL1.2	0.025479271	0.105505243	0.24149767	23.44560966
Eg01_t014520	Eg4CL10	Ms01t017810	Ms4CL10	0.050963394	0.362087034	0.140749017	80.46378532
Eg01_t014520	Eg4CL10	Ms03t058920	Ms4CL11	0.023547545	0.093651284	0.251438572	20.81139651
Eg01_t025930	Eg4CL11	Ms01t017810	Ms4CL10	0.01231302	0.108506079	0.113477699	24.11246205
Eg01_t025930	Eg4CL11	Ms03t058920	Ms4CL11	0.045249323	0.366113716	0.123593629	81.35860356
Eg01_t025930	Eg4CL11	Ms07t183930	Ms4CL14	0.083875315	0.458289121	0.183018341	101.842027
Eg04_t025780	Eg4CL12	Ms04t089570	Ms4CL13	0.014889853	0.088318133	0.168593384	19.62625169
Eg04_t025780	Eg4CL12	Ms04t083350	Ms4CL12	0.045744592	0.372479318	0.122811094	82.77318172
Eg04_t032900	Eg4CL13	Ms04t083350	Ms4CL12	0.019193037	0.09212845	0.208329097	20.47298891
Eg04_t032900	Eg4CL13	Ms04t089570	Ms4CL13	0.051400151	0.344218686	0.149324117	76.49304125
Eg07_t001640	Eg4CL14	Ms07t196560	Ms4CL15	0.041898569	0.122895352	0.340928832	27.3100782

Eg07_t014740	Eg4CL15	Ms04t089570	Ms4CL13	0.048195771	0.500649897	0.096266416	111.2555326
Eg07_t014740	Eg4CL15	Ms09t262230	Ms4CL16	0.023704239	0.092585382	0.256025722	20.57452931
Eg02_t037340	Eg4CL2	Ms02t041000	Ms4CL2	0.029372329	0.093308542	0.314787143	20.73523147
Eg09_t018540	Eg4CL2.2	Ms11t319670	Ms4CL2.2	0.011036132	0.063646732	0.173396674	14.14371818
Eg02_t001820	Eg4CL4	Ms08t219500	Ms4CL4	0.015150968	0.100408305	0.15089357	22.31295678
Eg05_t033420	Eg4CL5	Ms05t142250	Ms4CL5	0.024419783	0.093646658	0.260765129	20.81036834
Eg01_t038400	Eg4CL7	Ms01t006680	Ms4CL7	0.019797685	0.108206949	0.18296131	24.04598865
Eg03_t041750	Eg4CL8	Ms03t059300	Ms4CL8	0.040158291	0.101935694	0.3939571	22.65237655
Eg04_t006840	Eg4CL9	Ms06t178240	Ms4CL9	0.025694895	0.087691004	0.293016319	19.48688968
Eg06_t013840	EgANR1	Ms07t188910	MsANR3	0.051590353	0.112628525	0.458057613	25.02856116
Eg02_t015210	EgANR2	Ms08t207990	MsANR1	0.033810409	0.063475789	0.532650471	14.10573086
Eg07_t019620	EgC1	Ms09t266370	MsC1	0.05487863	0.095091209	0.577115702	21.13137979
Eg08_t035970	EgC1.2	Ms06t151760	MsC1.2	0.045822019	0.124285518	0.368683493	27.61900409
Eg08_t019660	EgC4H1	Ms10t281630	MsC4H1	0.009657021	0.081495162	0.118498093	18.11003592
Eg07_t003130	EgC4H2	Ms07t197940	MsC4H2	0.009631654	0.097892382	0.098390228	21.7538626
Eg08_t048930	EgC4H3	Ms10t281630	MsC4H1	0.036387942	0.361054381	0.100782441	80.2343069
Eg07_t034110	EgC4H4	Ms09t244050	MsC4H3	0.015773913	0.083129986	0.189749975	18.47333024
Eg01_t015550	EgCHI1	Ms04t093250	MsCHI2	0.050194071	0.060140597	0.83461211	13.3645771
Eg09_t018750	EgCHI2	Ms11t319860	MsCHI1	0.027678418	0.11903611	0.232521187	26.45246895
Eg08_t046640	EgCHS1	MsSC66t327440	MsCHS5	0.09887286	0.158904877	0.622214133	35.31219485
Eg08_t040870	EgCHS2	Ms06t158650	MsCHS2	0.021703399	0.085435496	0.254032576	18.98566568
Eg08_t040870	EgCHS2	Ms10t280970	MsCHS1	0.032099655	0.340620165	0.094238857	75.69336996
Eg08_t020390	EgCHS3	Ms06t158650	MsCHS2	0.045425972	0.32402875	0.140191179	72.00638892
Eg08_t020390	EgCHS3	Ms10t280970	MsCHS1	0.011301777	0.077703442	0.145447576	17.26743149
Eg08_t049400	EgCHS5	MsSC51t327380	MsCHS4	0.019392715	0.1121488	0.172919508	24.92195566

Eg08_t049400	EgCHS5	Ms06t158650	MsCHS2	0.108974557	0.367529742	0.296505411	81.673276
Eg08_t049400	EgCHS5	Ms10t280970	MsCHS1	0.107293231	0.384724902	0.278882991	85.49442257
Eg09_t036940	EgDFR1	Ms04t107810	MsDFR1	0.021692102	0.092034121	0.235696304	20.45202689
Eg04_t027830	EgDFR2	Ms04t087800	MsDFR2	0.021110665	0.065448478	0.32255395	14.54410629
Eg09_t010710	EgF3'5'H10	Ms11t312500	MsF3'5'H8	0.018194813	0.083203331	0.218678899	18.48962916
Eg02_t007770	EgF3'5'H2	Ms08t214430	MsF3'5'H1	0.023348189	0.096440853	0.242098532	21.43130064
Eg06_t004020	EgF3'5'H3	Ms09t255750	MsF3'5'H2	0.024679914	0.111286276	0.221769611	24.73028347
Eg02_t010650	EgF3'5'H5	Ms08t211840	MsF3'5'H3	0.022321415	0.066295721	0.33669466	14.73238253
Eg02_t019070	EgF3'5'H6	Ms02t024400	MsF3'5'H5	0.039101193	0.172440151	0.226752255	38.32003345
Eg02_t019100	EgF3'5'H7	Ms02t024420	MsF3'5'H7	0.160630791	0.486120138	0.330434348	108.0266973
Eg02_t019110	EgF3'5'H8	Ms02t024370	MsF3'5'H4	0.021300686	0.078363027	0.271820614	17.41400609
Eg02_t019180	EgF3'5'H9	Ms02t024400	MsF3'5'H5	0.089851232	0.362465952	0.24788875	80.54798922
Eg03_t018110	EgF3'H1	Ms03t075990	MsF3'H	0.025866522	0.069406088	0.372683767	15.42357501
Eg02_t022060	EgF3H2	Ms02t027200	MsF3H	0.011791537	0.102373304	0.115181757	22.74962304
Eg01_t007540	EgFLS2	Ms03t052640	MsFLS2	0.019866519	0.090779349	0.218844029	20.17318876
Eg08_t007020	EgFLS3	Ms10t292840	MsFLS3	0.065775128	0.235719582	0.279039727	52.38212934
Eg03_t031640	EgFLS4	Ms08t229420	MsFLS1	0.017185154	0.057763548	0.297508627	12.83634391
Eg05_t019870	EgLAR	Ms05t133790	MsLAR	0.035804646	0.113213008	0.316259118	25.15844618
Eg05_t014690	EgLDOX	Ms05t120140	MsLDOX	0.020733028	0.058525502	0.354256296	13.0056671
Eg01_t040920	EgPAL1	Ms01t004140	MsPAL1	0.012203659	0.075050065	0.16260691	16.67779215
Eg05_t014880	EgPAL2	Ms05t120010	MsPAL2	0.011247868	0.084219714	0.133553855	18.71549207
Eg02_t018790	EgPAL4	Ms02t024050	MsPAL4	0.022144164	0.088160077	0.251181321	19.59112811
Eg02_t018790	EgPAL4	Ms08t220210	MsPAL5	0.060504209	0.428112827	0.141327719	95.13618367
Eg09_t011330	EgPAL5	Ms02t024050	MsPAL4	0.069354685	0.373271387	0.185802307	82.94919717
Eg09_t011330	EgPAL5	Ms08t220210	MsPAL5	0.083072531	0.405778322	0.204723925	90.17296041

Eg09_t011330	EgPAL5	Ms11t313180	MsPAL3	0.021701333	0.079063813	0.274478705	17.56973631
Eg02_t001010	EgPAL6	Ms02t024050	MsPAL4	0.068309675	0.358209276	0.190697671	79.60206123
Eg02_t001010	EgPAL6	Ms05t135820	MsPAL7	0.067189621	0.401239167	0.167455288	89.16425941
Eg02_t001010	EgPAL6	Ms08t220210	MsPAL5	0.016964962	0.068769718	0.246692333	15.28215965
Eg09_t017980	EgPAL7	Ms05t135820	MsPAL7	0.04010364	0.385167969	0.104119873	85.59288196
Eg09_t017980	EgPAL7	Ms11t319150	MsPAL6	0.008760594	0.085594024	0.102350532	19.02089427
Eg05_t024420	EgPAL8	Ms05t135820	MsPAL7	0.011308523	0.075472919	0.149835502	16.77175982
Eg05_t024420	EgPAL8	Ms11t319150	MsPAL6	0.03417666	0.367210948	0.093070918	81.60243296
Eg06_t005550	EgPAL9	Ms09t254450	MsPAL8	0.008266173	0.06429884	0.128558666	14.28863105
Eg03_t004200	EgTT8.2	Ms03t064490	MsTT8.2	0.027791829	0.138726736	0.200335063	30.82816356
Eg05_t009200	EgTTG1	Ms05t125970	MsTTG1.2	0.013921513	0.091322181	0.152443944	20.29381791
Eg09_t041330	EgTTG1.2	Ms04t112120	MsTTG1	0.015821285	0.110290063	0.143451594	24.50890299
Eg08_t037030	EgUGT75C1.2	Ms06t152710	MsUGT75C1	0.058871564	0.120601552	0.488149304	26.80034496
Eg08_t037040	EgUGT75C1.3	Ms06t152730	MsUGT75C1.3	0.037713776	0.077793828	0.484791358	17.28751744
Eg08_t008340	EgUGT78D2	Ms10t291590	MsUGT78D2	0.049072609	0.123049247	0.398804625	27.34427709
Eg06_t008220	EgUGT78D2.2	Ms07t193970	MsUGT78D2.2	0.031696342	0.079762984	0.397381593	17.72510752
Eg01_t005290	EgUGT78D2.3	Ms03t050760	MsUGT78D2.3	0.035211831	0.10109291	0.348311579	22.46509108
Eg07_t037900	EgUGT79B1	Ms09t246270	MsUGT79B1	0.027679762	0.072992315	0.379214742	16.22051446

Table S4d. Divergence time, Ka, Ks and Ka/Ks values of orthologous flavonoid biosynthetic genes pairs between *M. acuminata* and *M. balbisiana*

Seq_1	Name1	Seq_2	Name2	Ka	Ks	Ka_Ks	Diversity_year
Parent=Macma4_08_g13040	Ma4CL1	Mba08_g12720	Mb4CL1.2	0.016183398	0.038593723	0.419327206	8.576382879
Parent=Macma4_05_g22890	Ma4CL1.2	Mba05_g21540	Mb4CL1	0.013224394	0.052863141	0.250162847	11.74736468
Parent=Macma4_01_g19800	Ma4CL10	Mba01_g28370	Mb4CL10	0.00572639	0.044466612	0.128779532	9.881469403

Parent=Macma4_03_g13930	Ma4CL11	Mba01_g28370	Mb4CL10	0.039447019	0.339818038	0.116082771	75.5151196
Parent=Macma4_04_g06110	Ma4CL12	Mba04_g05720	Mb4CL11	0.0107923	0.065299533	0.165273765	14.51100737
Parent=Macma4_04_g06110	Ma4CL12	Mba04_g12340	Mb4CL12	0.04307707	0.382206809	0.11270618	84.93484643
Parent=Macma4_04_g13130	Ma4CL13	Mba04_g12340	Mb4CL12	0.003277798	0.019967902	0.164153362	4.437311452
Parent=Macma4_07_g05220	Ma4CL14	Mba07_g04890	Mb4CL13	0.014573212	0.043576611	0.334427378	9.683691409
Parent=Macma4_07_g18890	Ma4CL15	Mba07_g17040	Mb4CL14	0.010623262	0.050142078	0.211863224	11.14268409
Parent=Macma4_02_g21360	Ma4CL2	Mba02_g18560	Mb4CL2	0.006261325	0.042061637	0.148860699	9.347030536
Parent=Macma4_11_g22750	Ma4CL2.2	Mba11_g20940	Mb4CL2.2	0.005497047	0.031160863	0.176408692	6.924636186
Parent=Macma4_08_g13960	Ma4CL4	Mba08_g13620	Mb4CL4	0.01354341	0.045519034	0.297532902	10.11534098
Parent=Macma4_05_g28920	Ma4CL5	Mba05_g27160	Mb4CL5	0.02014107	0.041838887	0.481395943	9.297530461
Parent=Macma4_01_g07350	Ma4CL7	Mba01_g16650	Mb4CL7	0.012625232	0.060334024	0.2092556	13.40756087
Parent=Macma4_03_g14310	Ma4CL8	Mba03_g13490	Mb4CL8	0.034549	0.068179503	0.506735875	15.15100068
Parent=Macma4_06_g39580	Ma4CL9	Mba06_g36630	Mb4CL9	0.013125848	0.033539552	0.391354293	7.453233823
Parent=Macma4_08_g01440	MaANR5	Mba08_g01400	MbANR	0.012717558	0.043547851	0.292036405	9.677300159
Parent=Macma4_10_g21630	MaC1	Mba10_g15520	MbC1	0.009472564	0.063670558	0.14877463	14.14901298
Parent=Macma4_09_g29590	MaC1.2	Mba09_g23680	MbC1.3	0.17018449	0.251325074	0.677148872	55.85001634
Parent=Macma4_10_g16840	MaC4H1	Mba06_g09560	MbC4H3	0.03574232	0.32611299	0.109601031	72.46955335
Parent=Macma4_07_g20380	MaC4H2	Mba07_g18380	MbC4H4	0.002614572	0.039980914	0.065395493	8.884647598
Parent=Macma4_06_g10450	MaC4H3	Mba06_g09560	MbC4H3	0.00437096	0.045587256	0.095881185	10.13050133
Parent=Macma4_09_g04800	MaC4H4	Mba09_g04380	MbC4H1	0.009588973	0.037598234	0.255037851	8.355163005
Parent=Macma4_04_g18950	MaCHI1	Mba04_g18040	MbCHI2	0.043092791	0.059758558	0.721114983	13.27967948
Parent=Macma4_11_g22930	MaCHI2	Mba11_g21110	MbCHI1	0.006200518	0.043754812	0.141710531	9.723291481
Parent=Macma4_06_g12560	MaCHS1	Mba06_g11590	MbCHS1	0.005637535	0.046932602	0.120119799	10.42946719
Parent=Macma4_06_g18170	MaCHS2	Mba06_g16830	MbCHS2	0.009610081	0.037664818	0.255147408	8.369959497
Parent=Macma4_06_g18170	MaCHS2	Mba06_g09120	MbCHS7	0.125578746	0.320627725	0.391665275	71.25060559

Parent=Macma4_06_g18170	MaCHS2	Mba10_g10820	MbCHS8	0.036427721	0.325308328	0.111979061	72.29073954
Parent=Macma4_06_g10010	MaCHS5	Mba06_g09120	MbCHS7	0.040649522	0.131898935	0.308186884	29.31087435
Parent=Macma4_10_g16140	MaCHS6	Mba06_g16830	MbCHS2	0.031517242	0.332252271	0.094859374	73.8338379
Parent=Macma4_10_g16140	MaCHS6	Mba10_g10820	MbCHS8	0.002246982	0.03968464	0.056620958	8.818808806
Parent=Macma4_04_g33220	MaDFR1	Mba04_g31800	MbDFR1	0.011463473	0.065311185	0.17552083	14.51359659
Parent=Macma4_03_g33910	MaDFR2	Mba01_g01310	MbDFR2	0.009222373	0.026488322	0.348167493	5.886293723
Parent=Macma4_08_g08290	MaF3'5'H1	Mba08_g07950	MbF3'5'H1	0.006184691	0.055710056	0.111015707	12.3800124
Parent=Macma4_02_g03260	MaF3'5'H10	Mba02_g01940	MbF3'5'H11	0.0320733	0.159387344	0.201228648	35.41940979
Parent=Macma4_02_g03290	MaF3'5'H11	Mba02_g01920	MbF3'5'H10	0.034419448	0.153695475	0.223945745	34.15454994
Parent=Macma4_02_g03300	MaF3'5'H12	Mba02_g01950	MbF3'5'H12	0.043827731	0.076625327	0.57197447	17.02785045
Parent=Macma4_11_g15260	MaF3'5'H13	Mba11_g13650	MbF3'5'H13	0.007953532	0.043638756	0.182258451	9.697501319
Parent=Macma4_11_g05110	MaF3'5'H2	Mba11_g04840	MbF3'5'H2	0.029145115	0.048170646	0.605038917	10.70458792
Parent=Macma4_09_g17660	MaF3'5'H4	Mba09_g16030	MbF3'5'H3	0.006156136	0.030065515	0.204757367	6.681225561
Parent=Macma4_10_g01260	MaF3'5'H6	Mba10_g00740	MbF3'5'H4	0.011354504	0.040981362	0.27706506	9.106969332
Parent=Macma4_02_g03040	MaF3'5'H7	Mba02_g01920	MbF3'5'H10	0.074227897	0.117392689	0.632304255	26.08726418
Parent=Macma4_03_g33170	MaF3'H	Mba01_g02030	MbF3'H	0.010826003	0.042282227	0.256041465	9.396050469
Parent=Macma4_07_g17110	MaF3H1	Mba07_g15320	MbF3H2	0.009437797	0.042537172	0.221871745	9.452704907
Parent=Macma4_02_g06130	MaF3H2	Mba02_g04670	MbF3H1	0.007050581	0.031295283	0.225292121	6.954507371
Parent=Macma4_03_g07220	MaFLS1	Mba03_g06940	MbFLS4	0.016535061	0.041616514	0.397319707	9.24811427
Parent=Macma4_03_g07220	MaFLS1	Mba09_g14060	MbFLS5	0.153355877	0.498751411	0.307479584	110.833647
Parent=Macma4_08_g24690	MaFLS2	Mba08_g23630	MbFLS1	0.00724906	0.080377973	0.090187142	17.86177179
Parent=Macma4_05_g19560	MaLAR	Mba05_g18490	MbLAR	0.018251851	0.01936216	0.942655765	4.302702115
Parent=Macma4_05_g03920	MaLDOX	Mba05_g13780	MbLDOX	0.010887288	0.041561879	0.261953697	9.23597319
Parent=Macma4_01_g04800	MaPAL1	Mba01_g14280	MbPAL8	0.005293224	0.040140084	0.131868777	8.920018758
Parent=Macma4_05_g03790	MaPAL2	Mba05_g13940	MbPAL7	0.005601395	0.039936234	0.140258473	8.874718755

Parent=Macma4_11_g15930	MaPAL3	Mba11_g14220	MbPAL6	0.020865022	0.047925308	0.435365414	10.65006852
Parent=Macma4_02_g02760	MaPAL4	Mba02_g01510	MbPAL5	0.050074297	0.088817771	0.563786908	19.73728255
Parent=Macma4_11_g22170	MaPAL6	Mba11_g20430	MbPAL2	0.003110102	0.021128158	0.147201755	4.695146161
Parent=Macma4_08_g14800	MaPAL7	Mba08_g14430	MbPAL3	0.00560925	0.015651041	0.35839471	3.478009049
Parent=Macma4_09_g16190	MaPAL8	Mba09_g14610	MbPAL1	0.005302718	0.039786335	0.13327987	8.841407869
Parent=Macma4_06_g25800	MaTT8	Mba06_g23950	MbTT8	0.072115694	0.098035442	0.735608393	21.78565374
Parent=Macma4_03_g19880	MaTT8.2	Mba03_g18730	MbTT8.2	0.093409853	0.128965766	0.724299601	28.65905911
Parent=Macma4_03_g19880	MaTT8.2	Mba06_g23950	MbTT8	0.113307434	0.421935181	0.268542277	93.76337363
Parent=Macma4_05_g10420	MaTTG1	Mba05_g07670	MbTTG1	0.00942575	0.036603787	0.257507506	8.134174944
Parent=Macma4_04_g37870	MaTTG1.2	Mba04_g36060	MbTTG1.2	0.00623186	0.051268312	0.121553831	11.3929582
Parent=Macma4_10_g31950	MaUGT75C1	Mba10_g24560	MbUGT75C1	0.0159793	0.014068408	1.135828569	3.126312845
Parent=Macma4_06_g07210	MaUGT75C1.2	Mba06_g06490	MbUGT75C1.3	0.011271346	0.03245344	0.347308185	7.211875657
Parent=Macma4_06_g07200	MaUGT75C1.3	Mba06_g06480	MbUGT75C1.2	0.019152014	0.053382209	0.358771482	11.86271308
Parent=Macma4_10_g27580	MaUGT78D2	Mba10_g20800	MbUGT78D2.2	0.030300388	0.059068052	0.51297422	13.12623384
Parent=Macma4_07_g16200	MaUGT78D2.2	Mba07_g14580	MbUGT78D2.3	0.010876921	0.040472246	0.26875012	8.993832482
Parent=Macma4_03_g04880	MaUGT78D2.3	Mba03_g04830	MbUGT78D2	0.014911028	0.051596271	0.288994294	11.465838

Table S4e. Divergence time, Ka, Ks and Ka/Ks values of orthologous flavonoid biosynthetic genes pairs between *M. acuminata* and *M. schizocharpa*

**Seq_1**	**Name1**	**Seq_2**	**Name2**	**Ka**	**Ks**	**Ka_Ks**	**Diversity_year**
Parent=Macma4_08_g13040	Ma4CL1	Ms08t218760	Ms4CL1	0.007492778	0.019208733	0.390071419	4.268607336

Parent=Macma4_05_g22890	Ma4CL1.2	Ms05t136800	Ms4CL1.2	0.004929836	0.022228423	0.221780753	4.939649472
Parent=Macma4_01_g19800	Ma4CL10	Ms01t017810	Ms4CL10	0.001631988	0.024362678	0.0669872	5.413928436
Parent=Macma4_01_g19800	Ma4CL10	Ms03t058920	Ms4CL11	0.038559243	0.333225729	0.115715083	74.05016192
Parent=Macma4_01_g19800	Ma4CL10	Ms07t183930	Ms4CL14	0.075310931	0.449422029	0.167572852	99.87156208
Parent=Macma4_03_g13930	Ma4CL11	Ms01t017810	Ms4CL10	0.039455271	0.347144062	0.113656764	77.14312494
Parent=Macma4_03_g13930	Ma4CL11	Ms03t058920	Ms4CL11	0.002445654	0.019513296	0.125332715	4.336287955
Parent=Macma4_03_g13930	Ma4CL11	Ms07t183930	Ms4CL14	0.071971516	0.480161248	0.149890305	106.7024995
Parent=Macma4_04_g06110	Ma4CL12	Ms04t083350	Ms4CL12	0.00578914	0.031985167	0.180994514	7.107814821
Parent=Macma4_04_g06110	Ma4CL12	Ms04t089570	Ms4CL13	0.044830481	0.37375165	0.119947245	83.05592232
Parent=Macma4_04_g13130	Ma4CL13	Ms04t089570	Ms4CL13	0.001637555	0.004938289	0.331603737	1.097397655
Parent=Macma4_07_g05220	Ma4CL14	Ms01t017810	Ms4CL10	0.076788935	0.427247549	0.179729374	94.94389985
Parent=Macma4_07_g05220	Ma4CL14	Ms03t058920	Ms4CL11	0.072459473	0.490835425	0.147624782	109.0745388
Parent=Macma4_07_g05220	Ma4CL14	Ms07t183930	Ms4CL14	0.007415096	0.027371895	0.270901827	6.082643403
Parent=Macma4_07_g18890	Ma4CL15	Ms07t196560	Ms4CL15	0.009149244	0.024471992	0.373865947	5.438220343
Parent=Macma4_09_g24760	Ma4CL16	Ms09t262230	Ms4CL16	0.004923095	0.022487631	0.218924556	4.9972513
Parent=Macma4_11_g22750	Ma4CL2.2	Ms11t319670	Ms4CL2.2	7.83E-04	0.01184391	0.066074021	2.63197995
Parent=Macma4_11_g14090	Ma4CL3	Ms11t311430	Ms4CL3	0.043415112	0.048132888	0.901984346	8.321008483
Parent=Macma4_08_g13960	Ma4CL4	Ms08t219500	Ms4CL4	8.34E-04	0.020370924	0.040959182	4.526872019
Parent=Macma4_05_g28920	Ma4CL5	Ms05t142250	Ms4CL5	0.010782602	0.020517997	0.525519237	4.559554869
Parent=Macma4_01_g07350	Ma4CL7	Ms01t006680	Ms4CL7	0.008184504	0.025633563	0.319288565	5.696347327
Parent=Macma4_03_g14310	Ma4CL8	Ms03t059300	Ms4CL8	0.010938719	0.015273682	0.716180888	2.34241607
Parent=Macma4_06_g39580	Ma4CL9	Ms06t178240	Ms4CL9	0.004899643	0.025594294	0.191434978	5.68762093
Parent=Macma4_07_g10660	MaANR2	Ms07t188910	MsANR3	0.028232302	0.058915661	0.479198586	13.09236919
Parent=Macma4_08_g01440	MaANR5	Ms08t207990	MsANR1	0.006338512	0.01941856	0.326415147	4.315235655
Parent=Macma4_09_g29590	MaC1.2	Ms09t266370	MsC1	0.010136716	0.010944107	0.926226019	2.432023698

Parent=Macma4_10_g16840	MaC4H1	Ms10t281630	MsC4H1	0.007019648	0.016667353	0.421161524	3.703856131
Parent=Macma4_07_g20380	MaC4H2	Ms07t197940	MsC4H2	0.003488885	0.037026978	0.0942255	8.228217264
Parent=Macma4_06_g10450	MaC4H3	Ms10t281630	MsC4H1	0.034363116	0.315819854	0.108806066	70.18218989
Parent=Macma4_09_g04800	MaC4H4	Ms09t244050	MsC4H3	0.001734606	0.025815168	0.067193293	5.736703981
Parent=Macma4_11_g22930	MaCHI2	Ms11t319860	MsCHI1	0.010952847	0.026182746	0.41832309	5.818388019
Parent=Macma4_06_g12560	MaCHS1	MsSC66t327440	MsCHS5	0.095019137	0.155167236	0.612365982	34.48160796
Parent=Macma4_06_g18170	MaCHS2	Ms06t158650	MsCHS2	0.0142117	0.041464448	0.342744208	9.214321842
Parent=Macma4_06_g18170	MaCHS2	Ms10t280970	MsCHS1	0.038777749	0.331098623	0.117118424	73.57747178
Parent=Macma4_06_g10010	MaCHS5	MsSC51t327380	MsCHS4	0.038761163	0.150595466	0.257385987	33.46565907
Parent=Macma4_06_g10010	MaCHS5	Ms06t158650	MsCHS2	0.117422023	0.388692181	0.302095151	86.37604011
Parent=Macma4_10_g16140	MaCHS6	Ms06t158650	MsCHS2	0.043030158	0.351872673	0.122289002	78.19392732
Parent=Macma4_10_g16140	MaCHS6	Ms10t280970	MsCHS1	0.002246351	0.014201608	0.158175849	3.155912834
Parent=Macma4_04_g33220	MaDFR1	Ms04t107810	MsDFR1	0.00490298	0.043333351	0.11314564	9.629633444
Parent=Macma4_03_g33910	MaDFR2	Ms03t076720	MsDFR3	0.009852358	0.020216857	0.487333822	4.492634998
Parent=Macma4_04_g11180	MaDFR3	Ms04t087800	MsDFR2	0.003678732	0.004204636	0.874922823	0.934363578
Parent=Macma4_08_g08290	MaF3'5'H1	Ms08t214430	MsF3'5'H1	0.007025225	0.030844748	0.227760816	6.854388376
Parent=Macma4_02_g03290	MaF3'5'H11	Ms02t024400	MsF3'5'H5	0.007037659	0.024530917	0.286889349	5.451314916
Parent=Macma4_02_g03300	MaF3'5'H12	Ms02t024420	MsF3'5'H7	0.189036887	0.221518094	0.853369957	49.2262432
Parent=Macma4_11_g15260	MaF3'5'H13	Ms11t312500	MsF3'5'H8	0.004407241	0.029747027	0.148157347	6.610450352
Parent=Macma4_08_g05480	MaF3'5'H3	Ms08t211840	MsF3'5'H3	0.009722461	0.016118821	0.603174447	3.58196012
Parent=Macma4_09_g17660	MaF3'5'H4	Ms09t255750	MsF3'5'H2	0.003748544	0.020315631	0.184515236	4.514584768
Parent=Macma4_02_g03040	MaF3'5'H7	Ms02t024400	MsF3'5'H5	0.093617041	0.195028562	0.48001708	43.33968054
Parent=Macma4_03_g33170	MaF3'H	Ms03t075990	MsF3'H	0.006935964	0.027927915	0.248352395	6.206203247
Parent=Macma4_02_g06130	MaF3H2	Ms02t027200	MsF3H	0.00351838	0.027275733	0.128993056	6.061274028
Parent=Macma4_03_g07220	MaFLS1	Ms03t052640	MsFLS2	0.003931857	0.017291832	0.227382323	3.842629383

Parent=Macma4_08_g24690	MaFLS2	Ms08t229420	MsFLS1	0.00524134	0.030638834	0.171068536	6.80862969
Parent=Macma4_05_g19560	MaLAR	Ms05t133790	MsLAR	0.017349837	0.035796267	0.484682847	7.9547261
Parent=Macma4_05_g03920	MaLDOX	Ms05t120140	MsLDOX	0	0.016360567	0	3.635681542
Parent=Macma4_01_g04800	MaPAL1	Ms01t004140	MsPAL1	6.20E-04	0.011549795	0.053716241	2.566621142
Parent=Macma4_05_g03790	MaPAL2	Ms05t120010	MsPAL2	6.20E-04	0.017717359	0.035006299	3.937190961
Parent=Macma4_11_g15930	MaPAL3	Ms11t313180	MsPAL3	0.002071348	0.01476841	0.140255298	3.281868815
Parent=Macma4_02_g02760	MaPAL4	Ms02t024050	MsPAL4	0.007531444	0.022917123	0.32863828	5.092694108
Parent=Macma4_02_g02760	MaPAL4	Ms08t220210	MsPAL5	0.06130516	0.38841449	0.157834378	86.31433114
Parent=Macma4_05_g21700	MaPAL5	Ms05t135820	MsPAL7	0.003742212	0.019268883	0.194210095	4.281973904
Parent=Macma4_11_g22170	MaPAL6	Ms11t319150	MsPAL6	0.00248808	0.01915813	0.129870727	4.257362175
Parent=Macma4_08_g14800	MaPAL7	Ms02t024050	MsPAL4	0.061075347	0.342676126	0.178230528	76.15025032
Parent=Macma4_08_g14800	MaPAL7	Ms08t220210	MsPAL5	0.006858972	0.019637843	0.349273205	4.363965011
Parent=Macma4_09_g16190	MaPAL8	Ms09t254450	MsPAL8	0.001175261	0.028715935	0.040927137	6.381318806
Parent=Macma4_03_g19880	MaTT8.2	Ms03t064490	MsTT8.2	0.01045853	0.024624242	0.42472494	5.472053777
Parent=Macma4_05_g10420	MaTTG1	Ms05t125970	MsTTG1.2	0.001252871	0.015180784	0.082530089	3.37350754
Parent=Macma4_04_g37870	MaTTG1.2	Ms04t112120	MsTTG1	0	0.019987858	0	4.441746332
Parent=Macma4_10_g31950	MaUGT75C1	Ms10t295740	MsUGT75C1.4	0.006647717	0.011439688	0.58110998	2.542152876
Parent=Macma4_06_g07210	MaUGT75C1.2	Ms06t152730	MsUGT75C1.3	0.003741531	0.017480762	0.214037056	3.88461382
Parent=Macma4_06_g07200	MaUGT75C1.3	Ms06t152710	MsUGT75C1	0.030358276	0.058194728	0.521667114	12.93216181
Parent=Macma4_10_g27580	MaUGT78D2	Ms10t291590	MsUGT78D2	0.015868361	0.035197074	0.450843171	7.821572037
Parent=Macma4_07_g16200	MaUGT78D2.2	Ms07t193970	MsUGT78D2.2	0.004971845	0.034788845	0.142914907	7.73085443
Parent=Macma4_03_g04880	MaUGT78D2.3	Ms03t050760	MsUGT78D2.3	0.008897781	0.014038781	0.6338001	3.119729209
Parent=Macma4_09_g07100	MaUGT79B1	Ms09t246270	MsUGT79B1	0.011530374	0.011442415	1.007687066	2.542758895

Table S4f. Divergence time, Ka, Ks and Ka/Ks values of orthologous flavonoid biosynthetic genes pairs between *M. acuminata* and *M. schizocharpa*

Seq_1	Name1	Seq_2	Name2	Ka	Ks	Ka_Ks	Diversity_year
Mba05_g21540	Mb4CL1	Ms05t136800	Ms4CL1.2	0.011565698	0.055371049	0.208876268	12.30467747
Mba08_g12720	Mb4CL1.2	Ms08t218760	Ms4CL1	0.014261244	0.041427926	0.344242293	9.206205704
Mba01_g28370	Mb4CL10	Ms01t017810	Ms4CL10	0.005729514	0.054625997	0.104886218	12.13911052
Mba01_g28370	Mb4CL10	Ms03t058920	Ms4CL11	0.038572669	0.344127865	0.112088189	76.47285884
Mba01_g28370	Mb4CL10	Ms07t183930	Ms4CL14	0.077155151	0.4443799	0.173624304	98.75108895
Mba04_g05720	Mb4CL11	Ms04t083350	Ms4CL12	0.014136653	0.068042434	0.207762306	15.12054092
Mba04_g05720	Mb4CL11	Ms04t089570	Ms4CL13	0.048321794	0.361929109	0.133511763	80.42869088
Mba04_g12340	Mb4CL12	Ms04t089570	Ms4CL13	0.004922085	0.025044063	0.196536997	5.565347343
Mba04_g12340	Mb4CL12	Ms04t083350	Ms4CL12	0.046100056	0.393246213	0.117229498	87.38804732
Mba07_g04890	Mb4CL13	Ms07t183930	Ms4CL14	0.014559281	0.049176158	0.296063811	10.9280351
Mba07_g17040	Mb4CL14	Ms07t196560	Ms4CL15	0.016540987	0.05761229	0.287108663	12.80273102
Mba11_g20940	Mb4CL2.2	Ms11t319670	Ms4CL2.2	0.004709284	0.033612346	0.140105768	7.46941025
Mba11_g12600	Mb4CL3.2	Ms11t311430	Ms4CL3	0.117634338	0.102305839	1.149830142	22.73463092
Mba08_g13620	Mb4CL4	Ms08t219500	Ms4CL4	0.010561772	0.049382672	0.213876068	10.97392719
Mba05_g27160	Mb4CL5	Ms05t142250	Ms4CL5	0.021847225	0.039436993	0.553977951	8.763776221
Mba01_g16650	Mb4CL7	Ms01t006680	Ms4CL7	0.009448944	0.049935244	0.189223946	11.09672085
Mba03_g13490	Mb4CL8	Ms03t059300	Ms4CL8	0.042106403	0.067845131	0.620625272	15.07669578
Mba06_g36630	Mb4CL9	Ms06t178240	Ms4CL9	0.014781838	0.044178173	0.334595944	9.817371804
Mba08_g01400	MbANR	Ms08t207990	MsANR1	0.016571739	0.039508805	0.419444189	8.779734391
Mba06_g05630	MbC1.2	Ms06t151760	MsC1.2	0.017586737	0.042830548	0.410612002	9.517899556
Mba09_g23680	MbC1.3	Ms09t266370	MsC1	0.182934985	0.250840554	0.729287914	55.74234541

Mba09_g04380	MbC4H1	Ms09t244050	MsC4H3	0.011347321	0.034621531	0.32775331	7.693673627
Mba06_g09560	MbC4H3	Ms10t281630	MsC4H1	0.033477738	0.331756574	0.10091055	73.72368313
Mba07_g18380	MbC4H4	Ms07t197940	MsC4H2	0.002614572	0.051806732	0.050467796	11.51260711
Mba11_g21110	MbCHI1	Ms11t319860	MsCHI1	0.006194116	0.043914982	0.141047911	9.758884792
Mba04_g18040	MbCHI2	Ms04t093250	MsCHI2	0.036279394	0.066520847	0.545383826	14.7824104
Mba06_g11590	MbCHS1	MsSC66t327440	MsCHS5	0.073701728	0.141407869	0.521199624	31.42397099
Mba06_g16830	MbCHS2	Ms06t158650	MsCHS2	0.013635448	0.062173869	0.219311555	13.81641543
Mba06_g16830	MbCHS2	Ms10t280970	MsCHS1	0.031517242	0.343261643	0.091816965	76.28036502
Mba06_g09090	MbCHS6	MsSC51t327370	MsCHS3	0.061634764	0.127288788	0.484212043	28.28639744
Mba06_g09120	MbCHS7	MsSC51t327380	MsCHS4	0.006520363	0.044819851	0.145479349	9.959966903
Mba10_g10820	MbCHS8	Ms06t158650	MsCHS2	0.040662452	0.35711559	0.11386356	79.35901995
Mba10_g10820	MbCHS8	Ms10t280970	MsCHS1	0.002246982	0.047131179	0.047675071	10.47359539
Mba04_g31800	MbDFR1	Ms04t107810	MsDFR1	0.0128093	0.045541144	0.281268737	10.12025429
Mba01_g01310	MbDFR2	Ms03t076720	MsDFR3	0.011696143	0.022382129	0.522566174	4.973806428
Mba08_g07950	MbF3'5'H1	Ms08t214430	MsF3'5'H1	0.00803019	0.050057667	0.160418774	11.1239261
Mba02_g01950	MbF3'5'H12	Ms02t024400	MsF3'5'H5	0.088357805	0.378038601	0.233726938	84.00857802
Mba11_g13650	MbF3'5'H13	Ms11t312500	MsF3'5'H8	0.005290638	0.049344122	0.107219224	10.9653604
Mba09_g16030	MbF3'5'H3	Ms09t255750	MsF3'5'H2	0.003755289	0.023134364	0.162325131	5.140969831
Mba01_g02030	MbF3'H	Ms03t075990	MsF3'H	0.012178593	0.03313344	0.367561983	7.362986769
Mba02_g04670	MbF3H1	Ms02t027200	MsF3H	0.005872021	0.043347585	0.135463617	9.632796608
Mba08_g23630	MbFLS1	Ms08t229420	MsFLS1	0.001442135	0.075472086	0.019108188	16.77157461
Mba10_g21980	MbFLS3	Ms10t292840	MsFLS3	0.065206786	0.254366038	0.256350207	56.52578623
Mba03_g06940	MbFLS4	Ms03t052640	MsFLS2	0.019214832	0.041616514	0.461711708	9.24811427
Mba09_g14060	MbFLS5	Ms03t052640	MsFLS2	0.154976915	0.498751411	0.310729777	110.833647
Mba05_g18490	MbLAR	Ms05t133790	MsLAR	0.029105746	0.04528864	0.642672124	10.06414214

Mba05_g13780	MbLDOX	Ms05t120140	MsLDOX	0.010889483	0.033040442	0.329580439	7.342320523
Mba09_g14610	MbPAL1	Ms09t254450	MsPAL8	0.005303499	0.047231099	0.112288278	10.49579986
Mba11_g20430	MbPAL2	Ms11t319150	MsPAL6	0.00435912	0.025010333	0.17429275	5.557851737
Mba08_g14430	MbPAL3	Ms02t024050	MsPAL4	0.061421772	0.341024262	0.180109682	75.7831693
Mba08_g14430	MbPAL3	Ms08t220210	MsPAL5	0.006546516	0.024620625	0.265895616	5.47125008
Mba02_g01510	MbPAL5	Ms02t024050	MsPAL4	0.04281569	0.063804104	0.671049157	14.17868986
Mba11_g14220	MbPAL6	Ms02t024050	MsPAL4	0.072903193	0.425306377	0.171413356	94.51252818
Mba11_g14220	MbPAL6	Ms11t313180	MsPAL3	0.013682027	0.043727485	0.312893056	9.717218984
Mba05_g13940	MbPAL7	Ms05t120010	MsPAL2	0.006224425	0.035881282	0.17347276	7.973618164
Mba01_g14280	MbPAL8	Ms01t004140	MsPAL1	0.004667581	0.036151576	0.129111401	8.033683636
Mba06_g23950	MbTT8	Ms03t064490	MsTT8.2	0.11189735	0.429698066	0.260409248	95.48845915
Mba03_g18730	MbTT8.2	Ms03t064490	MsTT8.2	0.096555655	0.128765197	0.749858325	28.6144883
Mba05_g07670	MbTTG1	Ms05t125970	MsTTG1.2	0.007551191	0.034566117	0.218456449	7.681359387
Mba04_g36060	MbTTG1.2	Ms04t112120	MsTTG1	0.00655026	0.040550416	0.161533731	9.011203545
Mba10_g24560	MbUGT75C1	Ms10t295740	MsUGT75C1.4	0.015261635	0.014262374	1.070062701	3.169416482
Mba06_g06480	MbUGT75C1.2	Ms06t152710	MsUGT75C1	0.024956313	0.056729344	0.439918947	12.60652098
Mba06_g06490	MbUGT75C1.3	Ms06t152730	MsUGT75C1.3	0.00937732	0.026479828	0.354130696	5.884406236
Mba03_g04830	MbUGT78D2	Ms03t050760	MsUGT78D2.3	0.015889373	0.048867856	0.325149783	10.85952361
Mba10_g20800	MbUGT78D2.2	Ms10t291590	MsUGT78D2	0.029768135	0.057634703	0.516496715	12.80771168
Mba07_g14580	MbUGT78D2.3	Ms07t193970	MsUGT78D2.2	0.016013953	0.03487311	0.459206333	7.749579944

Table S4g. Divergence time, Ka, Ks and Ka/Ks values of orthologous flavonoid biosynthetic genes pairs between *M. beccarii* and *E. glaucum*

Seq_1	Name1	Seq_2	Name2	Ka	Ks	Ka_Ks	Diversity_year
Mbe06_t016280	Mbe4CL1	Eg04_t006840	Eg4CL9	0.028156771	0.127216837	0.221328962	28.27040831
Mbe07_t013640	Mbe4CL10	Eg02_t002700	Eg4CL1	0.03826276	0.123142796	0.310718622	27.36506571

Mbe02_t027270	Mbe4CL11	Eg02_t037340	Eg4CL2	0.032789868	0.1019178	0.321728569	22.64839991
Mbe06_t040140	Mbe4CL12	Eg09_t018540	Eg4CL2.2	0.014219371	0.108387028	0.131190711	24.08600627
Mbe03_t056570	Mbe4CL13	Eg04_t025780	Eg4CL12	0.050913726	0.424796606	0.119854361	94.39924582
Mbe03_t056570	Mbe4CL13	Eg07_t014740	Eg4CL15	0.015330967	0.092024666	0.166596278	20.44992568
Mbe01_t005810	Mbe4CL14	Eg01_t025930	Eg4CL11	0.015210168	0.101697954	0.149562181	22.59954532
Mbe01_t005810	Mbe4CL14	Eg01_t014520	Eg4CL10	0.052253646	0.376333647	0.138849253	83.62969941
Mbe03_t000920	Mbe4CL15	Eg01_t014520	Eg4CL10	0.021055463	0.082535626	0.255107569	18.34125019
Mbe03_t000920	Mbe4CL15	Eg01_t025930	Eg4CL11	0.047028135	0.356907932	0.131765453	79.31287385
Mbe07_t022330	Mbe4CL16	Eg01_t025930	Eg4CL11	0.075289271	0.445490417	0.169003122	98.99787054
Mbe04_t006230	Mbe4CL17	Eg04_t032900	Eg4CL13	0.021725513	0.073383394	0.296054893	16.30742093
Mbe04_t006230	Mbe4CL17	Eg04_t025780	Eg4CL12	0.04427457	0.348093526	0.127191594	77.35411695
Mbe04_t013920	Mbe4CL18	Eg04_t025780	Eg4CL12	0.014054838	0.105043564	0.133800084	23.34301431
Mbe04_t013920	Mbe4CL18	Eg04_t032900	Eg4CL13	0.047016097	0.36371296	0.129267037	80.82510232
Mbe03_t000510	Mbe4CL2	Eg03_t041750	Eg4CL8	0.04547516	0.094217396	0.482662032	20.93719918
Mbe07_t014630	Mbe4CL3	Eg02_t001820	Eg4CL4	0.016003976	0.100368254	0.159452573	22.30405655
Mbe05_t019740	Mbe4CL4	Eg07_t001640	Eg4CL14	0.030728815	0.108062268	0.284362117	24.01383736
Mbe01_t019320	Mbe4CL6	Eg01_t038400	Eg4CL7	0.031831154	0.107281695	0.29670629	23.8403766
Mbe05_t004680	Mbe4CL7	Eg05_t033420	Eg4CL5	0.02315161	0.10765073	0.215062268	23.9223845
Mbe03_t020730	Mbe4CL8	Eg09_t002410	Eg4CL3	0.044630582	0.073087895	0.610642596	16.24175436
Mbe05_t011100	Mbe4CL9	Eg05_t023200	Eg4CL1.2	0.031036019	0.084705308	0.366399927	18.82340186
Mbe07_t028460	MbeANR	Eg06_t013840	EgANR1	0.045245013	0.124314848	0.363955022	27.62552174
Mbe01_t033880	MbeC1	Eg08_t035970	EgC1.2	0.038287124	0.14582585	0.262553749	32.40574448
Mbe03_t051550	MbeC1	Eg07_t019620	EgC1	0.049504291	0.119846005	0.413065848	26.63244545
Mbe01_t038520	MbeC4H1	Eg08_t048930	EgC4H3	0.009658434	0.096980096	0.099591925	21.55113255
Mbe10_t000860	MbeC4H2	Eg07_t003130	EgC4H2	0.00787696	0.075847915	0.103852025	16.85509226

Mbe02_t033650	MbeC4H3	Eg08_t019660	EgC4H1	0.010536502	0.08469968	0.124398365	18.82215106
Mbe02_t033650	MbeC4H3	Eg08_t048930	EgC4H3	0.03866624	0.363923178	0.106248358	80.87181734
Mbe08_t025090	MbeC4H4	Eg07_t034110	EgC4H4	0.017547225	0.089435856	0.196198997	19.87463469
Mbe04_t020690	MbeCHI1	Eg01_t015550	EgCHI1	0.055726329	0.080869124	0.689092778	17.97091648
Mbe06_t039860	MbeCHI2	Eg09_t018750	EgCHI2	0.031561459	0.114143862	0.276505969	25.36530256
Mbe01_t038030	MbeCHS2	Eg08_t049400	EgCHS5	0.023030421	0.111966071	0.205691069	24.88134906
Mbe01_t038030	MbeCHS2	Eg08_t040870	EgCHS2	0.104952554	0.327177575	0.320781626	72.7061278
Mbe01_t038030	MbeCHS2	Eg08_t020390	EgCHS3	0.115305009	0.384043822	0.300239197	85.34307159
Mbe01_t041060	MbeCHS3	Eg08_t046640	EgCHS1	0.026331045	0.065476271	0.402146379	14.55028248
Mbe01_t047580	MbeCHS4	Eg08_t040870	EgCHS2	0.014734135	0.062154915	0.237055018	13.81220342
Mbe01_t047580	MbeCHS4	Eg08_t020390	EgCHS3	0.035020233	0.316611117	0.110609613	70.3580259
Mbe01_t047580	MbeCHS4	Eg08_t049400	EgCHS5	0.1014938	0.347533796	0.292040088	77.22973239
Mbe11_t002790	MbeCHS5	Eg08_t049400	EgCHS5	0.103976596	0.359949685	0.288864249	79.98881897
Mbe11_t002790	MbeCHS5	Eg08_t020390	EgCHS3	0.013572472	0.066260109	0.20483625	14.7244686
Mbe11_t002790	MbeCHS5	Eg08_t040870	EgCHS2	0.031490171	0.306411099	0.102770986	68.09135532
Mbe04_t036630	MbeDFR1	Eg09_t036940	EgDFR1	0.026225889	0.094631686	0.277136445	21.0292636
Mbe04_t011700	MbeDFR3	Eg04_t027830	EgDFR2	0.01954906	0.055070601	0.354981784	12.23791143
Mbe07_t008510	MbeF3'5'H1	Eg02_t007770	EgF3'5'H2	0.022786563	0.068771849	0.331335613	15.28263311
Mbe07_t005300	MbeF3'5'H10	Eg02_t010650	EgF3'5'H5	0.046562661	0.097605274	0.477050668	21.690061
Mbe06_t049500	MbeF3'5'H2	Eg09_t010710	EgF3'5'H10	0.01414094	0.076157433	0.18568037	16.92387401
Mbe02_t005590	MbeF3'5'H3	Eg02_t019070	EgF3'5'H6	0.095564557	0.474424643	0.201432531	105.4276985
Mbe02_t005590	MbeF3'5'H3	Eg02_t019180	EgF3'5'H9	0.016849659	0.09976735	0.168889514	22.17052223
Mbe02_t005610	MbeF3'5'H4	Eg02_t019100	EgF3'5'H7	0.155747086	0.316963641	0.491372089	70.43636456
Mbe02_t006410	MbeF3'5'H5	Eg02_t019070	EgF3'5'H6	0.026726902	0.128562607	0.207890169	28.56946812
Mbe02_t006410	MbeF3'5'H5	Eg02_t019180	EgF3'5'H9	0.096886161	0.431106961	0.224738104	95.80154696

Mbe02_t005600	MbeF3'5'H6	Eg02_t019110	EgF3'5'H8	0.0612697	0.132616347	0.462007147	29.47029925
Mbe08_t039500	MbeF3'5'H7	Eg06_t004020	EgF3'5'H3	0.036770421	0.128562607	0.28601179	28.56946812
Mbe09_t008950	MbeF3'5'H8	Eg07_t042500	EgF3'5'H4	0.020826979	0.103686455	0.200864992	23.04143442
Mbe06_t019910	MbeF3'H	Eg03_t018110	EgF3'H1	0.04026328	0.114778749	0.350790367	25.50638878
Mbe07_t035880	MbeF3H1	Eg07_t027880	EgF3H1	0.020195241	0.149354476	0.135216845	33.1898836
Mbe08_t015900	MbeFLS1	Eg03_t031640	EgFLS4	0.025268347	0.066909126	0.377651734	14.8686946
Mbe02_t047300	MbeFLS2	Eg08_t007020	EgFLS3	0.031877308	0.104397848	0.305344494	23.19952169
Mbe03_t008100	MbeFLS4	Eg01_t007540	EgFLS2	0.007886162	0.100377354	0.078565156	22.3060787
Mbe05_t014700	MbeLAR	Eg05_t019870	EgLAR	0.030955495	0.117054756	0.264453119	26.012168
Mbe03_t031300	MbeLDOX	Eg05_t014690	EgLDOX	0.025669648	0.076391062	0.336029461	16.97579166
Mbe05_t009880	MbePAL1	Eg02_t001010	EgPAL6	0.06394699	0.412667672	0.154960019	91.70392722
Mbe05_t009880	MbePAL1	Eg05_t024420	EgPAL8	0.00815609	0.067006623	0.121720658	14.89036062
Mbe05_t009880	MbePAL1	Eg09_t017980	EgPAL7	0.037499799	0.396747313	0.094518093	88.16606957
Mbe06_t040740	MbePAL2	Eg05_t024420	EgPAL8	0.034974975	0.362222832	0.096556518	80.49396268
Mbe06_t040740	MbePAL2	Eg09_t017980	EgPAL7	0.013826724	0.091656191	0.150854234	20.36804247
Mbe07_t015550	MbePAL3	Eg02_t001010	EgPAL6	0.01826862	0.072612932	0.251589067	16.13620707
Mbe07_t015550	MbePAL3	Eg02_t018790	EgPAL4	0.058359751	0.370536095	0.157500852	82.34135434
Mbe06_t048840	MbePAL5	Eg02_t001010	EgPAL6	0.085861938	0.386316199	0.222258186	85.84804432
Mbe06_t048840	MbePAL5	Eg02_t018790	EgPAL4	0.06818482	0.427384155	0.159539888	94.97425675
Mbe06_t048840	MbePAL5	Eg09_t011330	EgPAL5	0.025059889	0.059962	0.417929497	13.32488898
Mbe01_t022110	MbePAL7	Eg01_t040920	EgPAL1	0.011582637	0.07695211	0.150517476	17.10046898
Mbe08_t037020	MbePAL8	Eg06_t005550	EgPAL9	0.007673456	0.058548459	0.131061621	13.01076877
Mbe06_t034420	MbeTT8	Eg03_t004200	EgTT8.2	0.029540166	0.137254456	0.215221911	30.50099024
Mbe06_t001720	MbeTT8	Eg04_t020700	EgTT8	0.035710727	0.128057456	0.278864877	28.45721249
Mbe03_t038300	MbeTTG1	Eg05_t009200	EgTTG1	0.014790462	0.130086288	0.113697314	28.90806402

Mbe04_t041380	MbeTTG1	Eg09_t041330	EgTTG1.2	0.015819547	0.110329134	0.143385039	24.51758544
Mbe01_t034870	MbeUGT75C1.3	Eg08_t037030	EgUGT75C1.2	0.071319721	0.1107188	0.644151862	24.60417784
Mbe02_t045890	MbeUGT78D2	Eg08_t008340	EgUGT78D2	0.037937959	0.115138777	0.329497676	25.58639478
Mbe07_t034770	MbeUGT78D2	Eg06_t008220	EgUGT78D2.2	0.024917166	0.067976597	0.366555062	15.10591048
Mbe03_t010580	MbeUGT78D2.3	Eg01_t005290	EgUGT78D2.3	0.030374287	0.104516641	0.290616755	23.22592015
Mbe08_t027530	MbeUGT79B1	Eg07_t037900	EgUGT79B1	0.036338449	0.081561768	0.445532874	18.12483734

Table S4h. Divergence time, Ka, Ks and Ka/Ks values of orthologous flavonoid biosynthetic genes pairs between *M. beccarii* and *M. acuminata*

**Seq_1**	**Name1**	**Seq_2**	**Name2**	**Ka**	**Ks**	**Ka_Ks**	**Diversity_year**
Mbe06_t016280	Mbe4CL1	Parent=Macma4_06_g39580	Ma4CL9	0.022346481	0.095960332	0.232872065	21.32451819
Mbe07_t013640	Mbe4CL10	Parent=Macma4_08_g13040	Ma4CL1	0.028703559	0.077375288	0.370965457	17.19450855
Mbe06_t040140	Mbe4CL12	Parent=Macma4_11_g22750	Ma4CL2.2	0.011031059	0.087126445	0.126609768	19.36143223
Mbe03_t056570	Mbe4CL13	Parent=Macma4_09_g24760	Ma4CL16	0.018246805	0.058516634	0.311822531	13.00369652
Mbe01_t005810	Mbe4CL14	Parent=Macma4_01_g19800	Ma4CL10	0.009017118	0.073028919	0.12347325	16.22864863
Mbe01_t005810	Mbe4CL14	Parent=Macma4_03_g13930	Ma4CL11	0.040157357	0.352052587	0.11406636	78.2339082
Mbe01_t005810	Mbe4CL14	Parent=Macma4_07_g05220	Ma4CL14	0.079952596	0.474385963	0.168539126	105.4191029
Mbe03_t000920	Mbe4CL15	Parent=Macma4_01_g19800	Ma4CL10	0.040174166	0.328863615	0.122160569	73.08080324
Mbe03_t000920	Mbe4CL15	Parent=Macma4_03_g13930	Ma4CL11	0.012321449	0.04961511	0.248340655	11.02558009
Mbe03_t000920	Mbe4CL15	Parent=Macma4_07_g05220	Ma4CL14	0.076772329	0.485633162	0.158087082	107.9184804
Mbe07_t022330	Mbe4CL16	Parent=Macma4_01_g19800	Ma4CL10	0.066804516	0.415380401	0.160827318	92.30675578
Mbe07_t022330	Mbe4CL16	Parent=Macma4_03_g13930	Ma4CL11	0.067533619	0.48300948	0.139818413	107.33544
Mbe07_t022330	Mbe4CL16	Parent=Macma4_07_g05220	Ma4CL14	0.023335216	0.063543488	0.367232214	14.12077501
Mbe04_t006230	Mbe4CL17	Parent=Macma4_04_g06110	Ma4CL12	0.014132735	0.084110961	0.168024896	18.69132468
Mbe04_t006230	Mbe4CL17	Parent=Macma4_04_g13130	Ma4CL13	0.044644302	0.336495894	0.132674136	74.77686538
Mbe04_t013920	Mbe4CL18	Parent=Macma4_04_g13130	Ma4CL13	0.010708584	0.061520381	0.17406563	13.67119579

Mbe04_t013920	Mbe4CL18	Parent=Macma4_04_g06110	Ma4CL12	0.040918279	0.396103457	0.103301999	88.02299046
Mbe03_t000510	Mbe4CL2	Parent=Macma4_03_g14310	Ma4CL8	0.036871087	0.080043579	0.460637661	17.78746206
Mbe07_t014630	Mbe4CL3	Parent=Macma4_08_g13960	Ma4CL4	0.010075718	0.067697559	0.148834292	15.04390206
Mbe05_t019740	Mbe4CL4	Parent=Macma4_07_g18890	Ma4CL15	0.020592961	0.08111312	0.253879531	18.02513779
Mbe01_t019320	Mbe4CL6	Parent=Macma4_01_g07350	Ma4CL7	0.027588841	0.104692896	0.26352161	23.26508808
Mbe05_t004680	Mbe4CL7	Parent=Macma4_05_g28920	Ma4CL5	0.016328884	0.064835163	0.251852294	14.40781393
Mbe03_t020730	Mbe4CL8	Parent=Macma4_11_g06130	Ma4CL6	0.034417316	0.075575831	0.455401085	16.79462915
Mbe05_t011100	Mbe4CL9	Parent=Macma4_05_g22890	Ma4CL1.2	0.018244283	0.055475815	0.328869127	12.32795893
Mbe07_t028460	MbeANR	Parent=Macma4_07_g10660	MaANR2	0.039643374	0.093468426	0.424136532	20.77076125
Mbe03_t051550	MbeC1	Parent=Macma4_09_g29590	MaC1.2	0.029100784	0.080037796	0.363588017	17.78617687
Mbe02_t039140	MbeC1.3	Parent=Macma4_10_g21630	MaC1	0.018993365	0.135069213	0.140619501	30.01538064
Mbe01_t038520	MbeC4H1	Parent=Macma4_06_g10450	MaC4H3	0.00788386	0.048576841	0.162296673	10.79485364
Mbe10_t000860	MbeC4H2	Parent=Macma4_07_g20380	MaC4H2	0.005243249	0.06362194	0.082412591	14.13820879
Mbe02_t033650	MbeC4H3	Parent=Macma4_06_g10450	MaC4H3	0.036633501	0.310074958	0.118144015	68.90554629
Mbe02_t033650	MbeC4H3	Parent=Macma4_10_g16840	MaC4H1	0.011435495	0.045361012	0.252099644	10.08022486
Mbe08_t025090	MbeC4H4	Parent=Macma4_09_g04800	MaC4H4	0.008713429	0.043532527	0.200159052	9.673894942
Mbe04_t020690	MbeCHI1	Parent=Macma4_04_g18950	MaCHI1	0.041691182	0.066778156	0.62432365	14.83959027
Mbe06_t039860	MbeCHI2	Parent=Macma4_11_g22930	MaCHI2	0.023022387	0.097760208	0.235498545	21.72449057
Mbe01_t038030	MbeCHS2	Parent=Macma4_06_g10010	MaCHS5	0.037056423	0.132735585	0.279174746	29.49679669
Mbe01_t038030	MbeCHS2	Parent=Macma4_06_g18170	MaCHS2	0.112781267	0.343201107	0.328615686	76.26691266
Mbe01_t038030	MbeCHS2	Parent=Macma4_10_g16140	MaCHS6	0.111797989	0.382389646	0.292366674	84.97547684
Mbe01_t041060	MbeCHS3	Parent=Macma4_06_g12560	MaCHS1	0.012454291	0.065875907	0.189056847	14.63909042
Mbe01_t047580	MbeCHS4	Parent=Macma4_06_g18170	MaCHS2	0.014166065	0.04143954	0.34184899	9.20878656
Mbe01_t047580	MbeCHS4	Parent=Macma4_06_g10010	MaCHS5	0.10983443	0.373831173	0.293807574	83.07359409
Mbe01_t047580	MbeCHS4	Parent=Macma4_10_g16140	MaCHS6	0.0326668	0.338612226	0.096472595	75.24716144

Mbe11_t002790	MbeCHS5	Parent=Macma4_06_g18170	MaCHS2	0.039338811	0.292112523	0.134670061	64.91389408
Mbe11_t002790	MbeCHS5	Parent=Macma4_10_g16140	MaCHS6	0.009019274	0.036108056	0.249785656	8.02401241
Mbe04_t036630	MbeDFR1	Parent=Macma4_04_g33220	MaDFR1	0.017789906	0.082779185	0.214907968	18.39537452
Mbe06_t019010	MbeDFR2	Parent=Macma4_03_g33910	MaDFR2	0.018615227	0.10633488	0.175062281	23.62997325
Mbe04_t011700	MbeDFR3	Parent=Macma4_04_g11180	MaDFR3	0.013374215	0.073071347	0.183029534	16.23807712
Mbe07_t008510	MbeF3'5'H1	Parent=Macma4_08_g08290	MaF3'5'H1	0.016903377	0.070358562	0.240246198	15.63523591
Mbe07_t005300	MbeF3'5'H10	Parent=Macma4_08_g05480	MaF3'5'H3	0.051141161	0.104782026	0.488071891	23.28489459
Mbe06_t049500	MbeF3'5'H2	Parent=Macma4_11_g15260	MaF3'5'H13	0.013726307	0.065364483	0.209996419	14.52544069
Mbe02_t005590	MbeF3'5'H3	Parent=Macma4_02_g03290	MaF3'5'H11	0.084989209	0.344417286	0.246762323	76.53717464
Mbe02_t005610	MbeF3'5'H4	Parent=Macma4_02_g03300	MaF3'5'H12	0.26455997	0.587838341	0.450055655	130.6307424
Mbe02_t006410	MbeF3'5'H5	Parent=Macma4_02_g03040	MaF3'5'H7	0.077005053	0.138666714	0.555324712	30.81482538
Mbe02_t005600	MbeF3'5'H6	Parent=Macma4_02_g03260	MaF3'5'H10	0.048718422	0.162695314	0.299445761	36.15451433
Mbe08_t039500	MbeF3'5'H7	Parent=Macma4_09_g17660	MaF3'5'H4	0.024037666	0.049787861	0.482801737	11.06396911
Mbe09_t008950	MbeF3'5'H8	Parent=Macma4_09_g17660	MaF3'5'H4	0.117104106	0.440303758	0.265962087	97.84527949
Mbe09_t008950	MbeF3'5'H8	Parent=Macma4_10_g01260	MaF3'5'H6	0.011843325	0.058476157	0.20253255	12.99470165
Mbe06_t019910	MbeF3'H	Parent=Macma4_03_g33170	MaF3'H	0.03306928	0.099925827	0.330938267	22.2057394
Mbe07_t035880	MbeF3H1	Parent=Macma4_07_g17110	MaF3H1	0.017801469	0.066710615	0.266846119	14.824581
Mbe08_t015900	MbeFLS1	Parent=Macma4_08_g24690	MaFLS2	0.021202825	0.057806378	0.366790418	12.84586172
Mbe03_t008100	MbeFLS4	Parent=Macma4_03_g07220	MaFLS1	0.014523504	0.076327492	0.190278801	16.96166487
Mbe05_t014700	MbeLAR	Parent=Macma4_05_g19560	MaLAR	0.020608788	0.083446878	0.246968955	18.5437507
Mbe03_t031300	MbeLDOX	Parent=Macma4_05_g03920	MaLDOX	0.01209216	0.033200441	0.364216857	7.377875842
Mbe05_t009880	MbePAL1	Parent=Macma4_05_g21700	MaPAL5	0.010975423	0.043972214	0.249599059	9.77160309
Mbe05_t009880	MbePAL1	Parent=Macma4_11_g22170	MaPAL6	0.035791444	0.34818076	0.102795583	77.37350225
Mbe06_t040740	MbePAL2	Parent=Macma4_05_g21700	MaPAL5	0.041128139	0.342568802	0.12005804	76.12640042
Mbe06_t040740	MbePAL2	Parent=Macma4_11_g22170	MaPAL6	0.007496548	0.058824846	0.12743847	13.07218806

Mbe07_t015550	MbePAL3	Parent=Macma4_02_g02760	MaPAL4	0.059828397	0.352447054	0.169751447	78.32156746
Mbe07_t015550	MbePAL3	Parent=Macma4_08_g14800	MaPAL7	0.010014753	0.064382671	0.155550449	14.30726031
Mbe06_t048840	MbePAL5	Parent=Macma4_02_g02760	MaPAL4	0.063233637	0.392271547	0.161198633	87.17145493
Mbe06_t048840	MbePAL5	Parent=Macma4_11_g15930	MaPAL3	0.019412277	0.056611572	0.342902991	12.58034939
Mbe01_t022110	MbePAL7	Parent=Macma4_01_g04800	MaPAL1	0.013464509	0.066710615	0.201834588	14.824581
Mbe08_t037020	MbePAL8	Parent=Macma4_09_g16190	MaPAL8	0.004121901	0.043483875	0.094791473	9.663083325
Mbe06_t034420	MbeTT8	Parent=Macma4_03_g19880	MaTT8.2	0.021164221	0.092312077	0.229268177	20.51379479
Mbe06_t001720	MbeTT8	Parent=Macma4_06_g25800	MaTT8	0.047226502	0.058162737	0.811971791	12.92505272
Mbe03_t038300	MbeTTG1	Parent=Macma4_05_g10420	MaTTG1	0.010604006	0.067397986	0.157334165	14.97733015
Mbe04_t041380	MbeTTG1	Parent=Macma4_04_g37870	MaTTG1.2	0.014488432	0.061657434	0.234982728	13.70165207
Mbe01_t034880	MbeUGT75C1	Parent=Macma4_06_g07210	MaUGT75C1.2	0.031445654	0.063021309	0.498968589	14.00473542
Mbe02_t051150	MbeUGT75C1	Parent=Macma4_10_g31950	MaUGT75C1	0.027471695	0.048926478	0.561489326	10.87255073
Mbe01_t034870	MbeUGT75C1.3	Parent=Macma4_06_g07200	MaUGT75C1.3	0.049253963	0.077184987	0.638128807	17.15221924
Mbe02_t045890	MbeUGT78D2	Parent=Macma4_10_g27580	MaUGT78D2	0.042160138	0.125638289	0.335567588	27.91961973
Mbe07_t034770	MbeUGT78D2	Parent=Macma4_07_g16200	MaUGT78D2.2	0.016863002	0.077181239	0.218485759	17.15138653
Mbe03_t010580	MbeUGT78D2.3	Parent=Macma4_03_g04880	MaUGT78D2.3	0.020112235	0.072002237	0.279327917	16.00049704
Mbe08_t027530	MbeUGT79B1	Parent=Macma4_09_g07100	MaUGT79B1	0.02523285	0.074355047	0.339356259	16.52334371

Table S4i. Divergence time, Ka, Ks and Ka/Ks values of orthologous flavonoid biosynthetic genes pairs between *M. beccarii* and *M. balbisiana*

Seq_1	Name1	Seq_2	Name2	Ka	Ks	Ka_Ks	Diversity_year
Mbe06_t016280	Mbe4CL1	Mba06_g36630	Mb4CL9	0.02400536	0.090575557	0.265031326	20.12790153
Mbe07_t013640	Mbe4CL10	Mba08_g12720	Mb4CL1.2	0.026878576	0.073057245	0.367911168	16.23494336
Mbe06_t040140	Mbe4CL12	Mba11_g20940	Mb4CL2.2	0.015015319	0.073966689	0.203001093	16.43704205
Mbe01_t005810	Mbe4CL14	Mba01_g28370	Mb4CL10	0.008195683	0.083595506	0.098039759	18.57677914
Mbe03_t000920	Mbe4CL15	Mba01_g28370	Mb4CL10	0.040331547	0.335289628	0.12028868	74.50880628

Mbe07_t022330	Mbe4CL16	Mba01_g28370	Mb4CL10	0.068626634	0.410607407	0.167134427	91.24609038
Mbe07_t022330	Mbe4CL16	Mba07_g04890	Mb4CL13	0.025348889	0.071306185	0.355493549	15.84581889
Mbe04_t006230	Mbe4CL17	Mba04_g05720	Mb4CL11	0.018339341	0.070759511	0.25917846	15.72433576
Mbe04_t006230	Mbe4CL17	Mba04_g12340	Mb4CL12	0.044631727	0.360149382	0.123925597	80.03319599
Mbe04_t013920	Mbe4CL18	Mba04_g12340	Mb4CL12	0.012366315	0.075062594	0.164746702	16.68057643
Mbe04_t013920	Mbe4CL18	Mba04_g05720	Mb4CL11	0.044391654	0.375855239	0.118108381	83.52338634
Mbe03_t000510	Mbe4CL2	Mba03_g13490	Mb4CL8	0.091801832	0.158323822	0.579835871	35.18307164
Mbe07_t014630	Mbe4CL3	Mba08_g13620	Mb4CL4	0.017738111	0.084032105	0.211087316	18.67380104
Mbe05_t019740	Mbe4CL4	Mba07_g17040	Mb4CL14	0.01975965	0.095924944	0.205990739	21.31665417
Mbe01_t019320	Mbe4CL6	Mba01_g16650	Mb4CL7	0.028358011	0.077087997	0.367865448	17.13066601
Mbe05_t004680	Mbe4CL7	Mba05_g27160	Mb4CL5	0.02356931	0.075909095	0.310493886	16.86868781
Mbe05_t011100	Mbe4CL9	Mba05_g21540	Mb4CL1	0.016584424	0.055301424	0.29989144	12.28920531
Mbe01_t033880	MbeC1	Mba06_g05630	MbC1.2	0.025815939	0.082728407	0.312056528	18.38409034
Mbe03_t051550	MbeC1	Mba09_g23680	MbC1.3	0.147611107	0.271515804	0.543655672	60.33684525
Mbe02_t039140	MbeC1.3	Mba10_g15520	MbC1	0.019020964	0.110239313	0.172542476	24.49762501
Mbe01_t038520	MbeC4H1	Mba06_g09560	MbC4H3	0.007009397	0.066325612	0.105681599	14.73902499
Mbe10_t000860	MbeC4H2	Mba07_g18380	MbC4H4	0.002616472	0.069733232	0.037521162	15.4962737
Mbe02_t033650	MbeC4H3	Mba06_g09560	MbC4H3	0.033006664	0.334478731	0.098680906	74.32860678
Mbe08_t025090	MbeC4H4	Mba09_g04380	MbC4H1	0.016643019	0.046526543	0.357710192	10.33923182
Mbe04_t020690	MbeCHI1	Mba04_g18040	MbCHI2	0.057765709	0.102431651	0.563943942	22.76258917
Mbe06_t039860	MbeCHI2	Mba11_g21110	MbCHI1	0.020885443	0.114289091	0.182742226	25.39757572
Mbe01_t038030	MbeCHS2	Mba06_g09120	MbCHS7	0.012214802	0.104002719	0.117446945	23.11171528
Mbe01_t038030	MbeCHS2	Mba06_g16830	MbCHS2	0.104317132	0.363048407	0.287336701	80.6774238
Mbe01_t041060	MbeCHS3	Mba06_g11590	MbCHS1	0.006769508	0.050682729	0.133566371	11.26282859
Mbe01_t047580	MbeCHS4	Mba06_g16830	MbCHS2	0.006765056	0.05455297	0.124008936	12.1228822

Mbe01_t047580	MbeCHS4	Mba10_g10820	MbCHS8	0.03033607	0.33823646	0.08968894	75.16365782
Mbe11_t002790	MbeCHS5	Mba10_g10820	MbCHS8	0.006756168	0.047245408	0.143001588	10.49897962
Mbe04_t036630	MbeDFR1	Mba04_g31800	MbDFR1	0.0128093	0.086160207	0.1486684	19.14671267
Mbe06_t019010	MbeDFR2	Mba01_g01310	MbDFR2	0.017332867	0.093241603	0.185891986	20.72035621
Mbe07_t008510	MbeF3'5'H1	Mba08_g07950	MbF3'5'H1	0.012412548	0.067508649	0.183866037	15.00192205
Mbe06_t049500	MbeF3'5'H2	Mba11_g13650	MbF3'5'H13	0.012384182	0.072716435	0.170307887	16.15920768
Mbe02_t005590	MbeF3'5'H3	Mba02_g01950	MbF3'5'H12	0.012376883	0.105940449	0.116828684	23.54232193
Mbe02_t005610	MbeF3'5'H4	Mba02_g01880	MbF3'5'H6	0.157999701	0.255337944	0.618786609	56.74176537
Mbe02_t006410	MbeF3'5'H5	Mba02_g01950	MbF3'5'H12	0.110202853	0.509436164	0.216323185	113.2080365
Mbe02_t005600	MbeF3'5'H6	Mba02_g01940	MbF3'5'H11	0.032142037	0.063755179	0.504147861	14.16781747
Mbe08_t039500	MbeF3'5'H7	Mba09_g16030	MbF3'5'H3	0.019561011	0.052374891	0.373480702	11.63886461
Mbe09_t008950	MbeF3'5'H8	Mba10_g00740	MbF3'5'H4	0.016218176	0.056558492	0.286750511	12.56855372
Mbe06_t019910	MbeF3'H	Mba01_g02030	MbF3'H	0.038946803	0.092874685	0.419347886	20.63881888
Mbe07_t035880	MbeF3H1	Mba07_g15320	MbF3H2	0.017797948	0.087574515	0.203232048	19.46100335
Mbe08_t015900	MbeFLS1	Mba08_g23630	MbFLS1	0.016029754	0.091290257	0.175591071	20.28672382
Mbe02_t047300	MbeFLS2	Mba10_g21980	MbFLS3	0.025850344	0.068253888	0.378738045	15.16753065
Mbe08_t036240	MbeFLS3	Mba09_g14060	MbFLS5	0.083368337	0.145369652	0.573492028	32.30436722
Mbe03_t008100	MbeFLS4	Mba03_g06940	MbFLS4	0.014536299	0.080825798	0.17984727	17.96128843
Mbe03_t008100	MbeFLS4	Mba09_g14060	MbFLS5	0.149641669	0.517019962	0.289431124	114.8933249
Mbe05_t014700	MbeLAR	Mba05_g18490	MbLAR	0.028868641	0.068092097	0.42396464	15.13157713
Mbe03_t031300	MbeLDOX	Mba05_g13780	MbLDOX	0.062273669	0.126456267	0.492452218	28.10139275
Mbe06_t040740	MbePAL2	Mba11_g20430	MbPAL2	0.009381474	0.056806388	0.165148227	12.62364178
Mbe07_t015550	MbePAL3	Mba08_g14430	MbPAL3	0.009071115	0.065411864	0.138676901	14.53596987
Mbe06_t048840	MbePAL5	Mba11_g14220	MbPAL6	0.030104148	0.073469868	0.409748221	16.32663741
Mbe01_t022110	MbePAL7	Mba01_g14280	MbPAL8	0.011891352	0.067662693	0.17574458	15.03615408

Mbe08_t037020	MbePAL8	Mba09_g14610	MbPAL1	0.005895677	0.047216799	0.124863979	10.49262203
Mbe06_t034420	MbeTT8	Mba03_g18730	MbTT8.2	0.104261625	0.155083736	0.672292451	34.4630524
Mbe06_t034420	MbeTT8	Mba06_g23950	MbTT8	0.110492037	0.46406338	0.238096868	103.1251956
Mbe03_t038300	MbeTTG1	Mba05_g07670	MbTTG1	0.012984782	0.075203837	0.172661168	16.71196375
Mbe04_t041380	MbeTTG1	Mba04_g36060	MbTTG1.2	0.011830676	0.065999274	0.179254637	14.66650523
Mbe02_t051150	MbeUGT75C1	Mba10_g24560	MbUGT75C1	0.024611768	0.039914406	0.616613664	8.869868045
Mbe01_t034870	MbeUGT75C1.3	Mba06_g06490	MbUGT75C1.3	0.133288396	0.328032643	0.406326623	72.89614281
Mbe02_t045890	MbeUGT78D2	Mba10_g20800	MbUGT78D2.2	0.030631872	0.096217156	0.318361857	21.3815902
Mbe07_t034770	MbeUGT78D2	Mba07_g14580	MbUGT78D2.3	0.015847404	0.068026895	0.232957916	15.11708779
Mbe03_t010580	MbeUGT78D2.3	Mba03_g04830	MbUGT78D2	0.015036527	0.096193127	0.156316026	21.37625054

Table S4j. Divergence time, Ka, Ks and Ka/Ks values of orthologous flavonoid biosynthetic genes pairs between *M. beccarii* and *M. schizocarpa*

**Seq_1**	**Name1**	**Seq_2**	**Name2**	**Ka**	**Ks**	**Ka_Ks**	**Diversity_year**
Mbe06_t016280	Mbe4CL1	Ms06t178240	Ms4CL9	0.023185452	0.110339622	0.210128074	24.51991593
Mbe07_t013640	Mbe4CL10	Ms08t218760	Ms4CL1	0.029572966	0.072075286	0.410306613	16.01673022
Mbe02_t027270	Mbe4CL11	Ms02t041000	Ms4CL2	0.023896204	0.078202793	0.305567146	17.37839844
Mbe06_t040140	Mbe4CL12	Ms11t319670	Ms4CL2.2	0.010237723	0.089770784	0.114042926	19.94906322
Mbe03_t056570	Mbe4CL13	Ms04t089570	Ms4CL13	0.052154378	0.45144524	0.115527584	100.3211643
Mbe03_t056570	Mbe4CL13	Ms09t262230	Ms4CL16	0.021602158	0.055958148	0.386041332	12.43514397
Mbe01_t005810	Mbe4CL14	Ms01t017810	Ms4CL10	0.009022049	0.08622531	0.104633413	19.16118001
Mbe01_t005810	Mbe4CL14	Ms03t058920	Ms4CL11	0.039711163	0.354519054	0.112014184	78.7820121
Mbe01_t005810	Mbe4CL14	Ms07t183930	Ms4CL14	0.080334157	0.487716512	0.16471486	108.3814471
Mbe03_t000920	Mbe4CL15	Ms01t017810	Ms4CL10	0.0401966	0.34698525	0.115845271	77.10783333
Mbe03_t000920	Mbe4CL15	Ms03t058920	Ms4CL11	0.011488975	0.054830303	0.209536963	12.18451188

Mbe03_t000920	Mbe4CL15	Ms07t183930	Ms4CL14	0.075338023	0.485108771	0.155301302	107.801949
Mbe07_t022330	Mbe4CL16	Ms01t017810	Ms4CL10	0.068641396	0.406092075	0.169029143	90.24268324
Mbe07_t022330	Mbe4CL16	Ms03t058920	Ms4CL11	0.066607248	0.493183151	0.135055807	109.5962557
Mbe07_t022330	Mbe4CL16	Ms07t183930	Ms4CL14	0.024160367	0.077139221	0.313204711	17.14204914
Mbe04_t006230	Mbe4CL17	Ms04t083350	Ms4CL12	0.016645388	0.08967803	0.185612777	19.92845104
Mbe04_t006230	Mbe4CL17	Ms04t089570	Ms4CL13	0.046387644	0.344175142	0.134779183	76.48336493
Mbe04_t013920	Mbe4CL18	Ms04t089570	Ms4CL13	0.012369714	0.066882334	0.184947405	14.86274093
Mbe04_t013920	Mbe4CL18	Ms04t083350	Ms4CL12	0.04393457	0.403182143	0.108969533	89.59603168
Mbe03_t000510	Mbe4CL2	Ms03t059300	Ms4CL8	0.043272474	0.07875672	0.549444844	17.50149334
Mbe07_t014630	Mbe4CL3	Ms08t219500	Ms4CL4	0.010921504	0.075062594	0.145498616	16.68057643
Mbe05_t019740	Mbe4CL4	Ms07t196560	Ms4CL15	0.025948534	0.083595506	0.310405849	18.57677914
Mbe01_t019320	Mbe4CL6	Ms01t006680	Ms4CL7	0.027170122	0.087766283	0.30957357	19.50361849
Mbe05_t004680	Mbe4CL7	Ms05t142250	Ms4CL5	0.019725555	0.072230891	0.273090285	16.05130919
Mbe05_t011100	Mbe4CL9	Ms05t136800	Ms4CL1.2	0.01657755	0.057987711	0.285880404	12.88615793
Mbe07_t028460	MbeANR	Ms07t188910	MsANR3	0.039327929	0.105907211	0.371343263	23.53493571
Mbe01_t033880	MbeC1	Ms06t151760	MsC1.2	0.02747412	0.076779313	0.357832318	17.0620696
Mbe03_t051550	MbeC1	Ms09t266370	MsC1	0.039664415	0.067971013	0.58354897	15.10466958
Mbe01_t038520	MbeC4H1	Ms10t281630	MsC4H1	0.033011573	0.321501004	0.102679533	71.44466753
Mbe10_t000860	MbeC4H2	Ms07t197940	MsC4H2	0.005243249	0.078866367	0.066482698	17.52585929
Mbe02_t033650	MbeC4H3	Ms10t281630	MsC4H1	0.011442205	0.045275413	0.252724478	10.06120298
Mbe08_t025090	MbeC4H4	Ms09t244050	MsC4H3	0.010469852	0.046503998	0.225138743	10.33422172
Mbe04_t020690	MbeCHI1	Ms04t093250	MsCHI2	0.035650275	0.072784342	0.489806934	16.17429819
Mbe06_t039860	MbeCHI2	Ms11t319860	MsCHI1	0.022998318	0.114434691	0.200973302	25.42993127
Mbe01_t038030	MbeCHS2	MsSC51t327380	MsCHS4	0.011532872	0.091712473	0.125750306	20.38054955
Mbe01_t038030	MbeCHS2	Ms06t158650	MsCHS2	0.116787417	0.345525927	0.337998998	76.78353943

Mbe01_t038030	MbeCHS2	Ms10t280970	MsCHS1	0.110495764	0.394148828	0.280340207	87.58862853
Mbe01_t041060	MbeCHS3	MsSC66t327440	MsCHS5	0.081540411	0.168281506	0.484547667	37.39589029
Mbe01_t047580	MbeCHS4	Ms06t158650	MsCHS2	0.018224131	0.062306872	0.292489907	13.84597162
Mbe01_t047580	MbeCHS4	Ms10t280970	MsCHS1	0.0326668	0.349738995	0.093403369	77.71977672
Mbe11_t002790	MbeCHS5	Ms06t158650	MsCHS2	0.043592535	0.322505347	0.13516841	71.66785494
Mbe11_t002790	MbeCHS5	Ms10t280970	MsCHS1	0.009019274	0.043543061	0.207134598	9.676235771
Mbe04_t036630	MbeDFR1	Ms04t107810	MsDFR1	0.017771087	0.073396948	0.242122976	16.31043281
Mbe06_t019010	MbeDFR2	Ms03t076720	MsDFR3	0.0198646	0.101923357	0.194897427	22.64963493
Mbe04_t011700	MbeDFR3	Ms04t087800	MsDFR2	0.012358673	0.069767041	0.177141997	15.50378695
Mbe07_t008510	MbeF3'5'H1	Ms08t214430	MsF3'5'H1	0.020746811	0.068120671	0.3045597	15.13792688
Mbe07_t005300	MbeF3'5'H10	Ms08t211840	MsF3'5'H3	0.04936988	0.10118761	0.487904394	22.48613562
Mbe06_t049500	MbeF3'5'H2	Ms11t312500	MsF3'5'H8	0.012827441	0.074238302	0.172787374	16.49740053
Mbe02_t005590	MbeF3'5'H3	Ms02t024400	MsF3'5'H5	0.084531185	0.363179019	0.232753493	80.70644865
Mbe02_t005610	MbeF3'5'H4	Ms02t024420	MsF3'5'H7	0.233654738	0.56302065	0.415002074	125.1157001
Mbe02_t006410	MbeF3'5'H5	Ms02t024400	MsF3'5'H5	0.038613944	0.134931273	0.286174905	29.98472725
Mbe08_t039500	MbeF3'5'H7	Ms09t255750	MsF3'5'H2	0.021869864	0.043882854	0.498369234	9.751745292
Mbe06_t019910	MbeF3'H	Ms03t075990	MsF3'H	0.039275796	0.088940851	0.441594556	19.76463367
Mbe08_t015900	MbeFLS1	Ms08t229420	MsFLS1	0.015824763	0.044260629	0.357535882	9.83569524
Mbe02_t047300	MbeFLS2	Ms10t292840	MsFLS3	0.07091356	0.208223802	0.340564139	46.2719561
Mbe03_t008100	MbeFLS4	Ms03t052640	MsFLS2	0.017194625	0.081068717	0.212099386	18.01527038
Mbe05_t014700	MbeLAR	Ms05t133790	MsLAR	0.035800456	0.113254989	0.316104893	25.16777539
Mbe03_t031300	MbeLDOX	Ms05t120140	MsLDOX	0.012094598	0.033177489	0.364542288	7.372775439
Mbe05_t009880	MbePAL1	Ms05t135820	MsPAL7	0.008147996	0.05694589	0.14308313	12.65464217
Mbe05_t009880	MbePAL1	Ms11t319150	MsPAL6	0.034821266	0.349267872	0.099697878	77.61508264
Mbe06_t040740	MbePAL2	Ms05t135820	MsPAL7	0.039533435	0.354459876	0.111531482	78.76886136

Mbe06_t040740	MbePAL2	Ms11t319150	MsPAL6	0.007498891	0.058767201	0.127603335	13.0593779
Mbe07_t015550	MbePAL3	Ms02t024050	MsPAL4	0.061168463	0.331768271	0.184371046	73.7262824
Mbe07_t015550	MbePAL3	Ms08t220210	MsPAL5	0.01127311	0.077160124	0.14610021	17.14669423
Mbe06_t048840	MbePAL5	Ms02t024050	MsPAL4	0.067020691	0.391149273	0.171343002	86.92206069
Mbe06_t048840	MbePAL5	Ms11t313180	MsPAL3	0.017656183	0.054529823	0.323789474	12.11773837
Mbe01_t022110	MbePAL7	Ms01t004140	MsPAL1	0.012830187	0.064673788	0.198383102	14.3719529
Mbe08_t037020	MbePAL8	Ms09t254450	MsPAL8	0.004122507	0.045331016	0.090942314	10.07355903
Mbe06_t034420	MbeTT8	Ms03t064490	MsTT8.2	0.019450037	0.092697813	0.209821958	20.5995139
Mbe03_t038300	MbeTTG1	Ms05t125970	MsTTG1.2	0.006282759	0.070965572	0.088532497	15.77012706
Mbe04_t041380	MbeTTG1	Ms04t112120	MsTTG1	0.014488432	0.048913765	0.296203577	10.86972553
Mbe02_t051150	MbeUGT75C1	Ms10t295740	MsUGT75C1.4	0.026007903	0.049665257	0.52366392	11.03672382
Mbe01_t034870	MbeUGT75C1.3	Ms06t152730	MsUGT75C1.3	0.132854542	0.349272971	0.380374529	77.61621581
Mbe02_t045890	MbeUGT78D2	Ms10t291590	MsUGT78D2	0.041611839	0.124202882	0.335031188	27.60064037
Mbe07_t034770	MbeUGT78D2	Ms07t193970	MsUGT78D2.2	0.02208995	0.074763043	0.295466172	16.61400961
Mbe03_t010580	MbeUGT78D2.3	Ms03t050760	MsUGT78D2.3	0.020070185	0.072460248	0.276982003	16.10227742
Mbe08_t027530	MbeUGT79B1	Ms09t246270	MsUGT79B1	0.024228869	0.074521414	0.325126269	16.56031433

Table S4k. Ka/Ks values of orthologous flavonoid biosynthetic genes pairs between *M. lasiocarpa* and *E. glaucum*

**Seq_1**	**Name1**	**Seq_2**	**Name2**	**Ka**	**Ks**	**Ka/Ks**	**Divergence (Mya)**
ML1h07G00167	Ml4CL1	Eg02_t001820	Eg4CL4	0.010912336	0.062974035	0.173283096	13.99423002
ML1h07G00248	Ml4CL10	Eg02_t002700	Eg4CL1	0.028479506	0.062549797	0.455309333	13.8999548
ML1h04G02613	Ml4CL11	Eg09_t018540	Eg4CL2.2	0.007076915	0.048452768	0.146058014	10.76728184
ML1h07G03382	Ml4CL12	Eg02_t037340	Eg4CL2	0.021146424	0.061016831	0.346567061	13.55929576
ML1h02G02377	Ml4CL13	Eg01_t025930	Eg4CL11	0.009006044	0.049779826	0.180917545	11.06218358
ML1h02G02377	Ml4CL13	Eg01_t014520	Eg4CL10	0.053560305	0.354882672	0.150923978	78.86281603

ML1h09G01309	Ml4CL14	Eg01_t014520	Eg4CL10	0.017696294	0.061540782	0.287553938	13.67572934
ML1h09G01309	Ml4CL14	Eg01_t025930	Eg4CL11	0.050453313	0.369960986	0.136374687	82.21355235
ML1h08G00557	Ml4CL15	Eg07_t014740	Eg4CL15	0.010733636	0.058429874	0.183701164	12.98441635
ML1h06G03426	Ml4CL16	Eg04_t025780	Eg4CL12	0.009074147	0.053130744	0.170789	11.80683192
ML1h06G03426	Ml4CL16	Eg04_t032900	Eg4CL13	0.04663177	0.344725008	0.135272375	76.60555743
ML1h06G02761	Ml4CL17	Eg04_t032900	Eg4CL13	0.018480001	0.044288728	0.41726196	9.841939445
ML1h06G02761	Ml4CL17	Eg04_t025780	Eg4CL12	0.054798151	0.348272923	0.157342553	77.39398278
ML1h09G02144	Ml4CL18	Eg04_t006840	Eg4CL9	0.013819657	0.046109268	0.299715378	10.246504
ML1h08G00140	Ml4CL2	Eg07_t001640	Eg4CL14	0.024419318	0.079353915	0.307726693	17.63420344
ML1h04G04085	Ml4CL3	Eg09_t002410	Eg4CL3	0.02720826	0.055816111	0.487462478	12.40358021
ML1h03G00142	Ml4CL4	Eg03_t041750	Eg4CL8	0.019625693	0.065769815	0.298399702	14.61551446
ML1h02G00664	Ml4CL5	Eg01_t038400	Eg4CL7	0.011481124	0.059614287	0.192590136	13.24761924
ML1h01G00405	Ml4CL8	Eg05_t033420	Eg4CL5	0.018452147	0.068497612	0.269383796	15.22169163
ML1h01G00972	Ml4CL9	Eg05_t023200	Eg4CL1.2	0.011529978	0.052466756	0.219757784	11.65927905
ML1h06G01342	MlANR2	Eg06_t013840	EgANR1	0.037270463	0.079574011	0.468374818	17.68311346
ML1h07G01339	MlANR4	Eg02_t015210	EgANR2	0.019034056	0.038289039	0.497115004	8.508675389
ML1h08G01785	MlC1	Eg07_t019620	EgC1	0.029138198	0.067778924	0.429900564	15.06198303
ML1h05G03234	MlC1	Eg08_t035970	EgC1.2	0.019109314	0.086896354	0.219909272	19.31030091
ML1h05G01754	MlC4H1	Eg08_t019660	EgC4H1	0.004370323	0.045608921	0.095821679	10.13531575
ML1h05G01754	MlC4H1	Eg08_t048930	EgC4H3	0.036355405	0.353455818	0.102857	78.54573723
ML1h08G00252	MlC4H2	Eg07_t003130	EgC4H2	0.008209264	0.057955651	0.141647344	12.87903362
ML1h05G04363	MlC4H3	Eg08_t048930	EgC4H3	0.008780374	0.060132358	0.146017455	13.36274633
ML1h05G04363	MlC4H3	Eg08_t019660	EgC4H1	0.033425509	0.295940509	0.112946718	65.76455764
ML1h08G03392	MlC4H4	Eg07_t034110	EgC4H4	0.013124651	0.061422859	0.213676987	13.64952412
ML1h09G01419	MlCHI1	Eg01_t015550	EgCHI1	0.020913903	0.053382209	0.391776644	11.86271308

ML1h04G02595	MlCHI2	Eg09_t018750	EgCHI2	0.010254817	0.043464182	0.235937192	9.658707121
ML1h05G01818	MlCHS1	Eg08_t049400	EgCHS5	0.106062297	0.401538611	0.264139722	89.23080248
ML1h05G01818	MlCHS1	Eg08_t020390	EgCHS3	0.011308167	0.058352626	0.193790208	12.96725019
ML1h05G01818	MlCHS1	Eg08_t040870	EgCHS2	0.033290974	0.323508417	0.102906053	71.89075935
ML1h05G03634	MlCHS2	Eg08_t040870	EgCHS2	0.011310299	0.035913503	0.314931657	7.980778545
ML1h05G03634	MlCHS2	Eg08_t049400	EgCHS5	0.102801707	0.358470458	0.286778742	79.66010169
ML1h05G03634	MlCHS2	Eg08_t020390	EgCHS3	0.033265512	0.313707975	0.106039739	69.71288336
ML1h05G04408	MlCHS3	Eg08_t049400	EgCHS5	0.030391162	0.102804521	0.295620873	22.84544902
ML1h05G04408	MlCHS3	Eg08_t020390	EgCHS3	0.128648066	0.366730391	0.350797395	81.49564242
ML1h05G04408	MlCHS3	Eg08_t040870	EgCHS2	0.120098339	0.324407833	0.370207891	72.09062946
ML1h04G00849	MlDFR1	Eg09_t036940	EgDFR1	0.010144929	0.06288755	0.161318562	13.97501103
ML1h06G03238	MlDFR2	Eg04_t027830	EgDFR2	0.015187317	0.04458334	0.340650043	9.907408801
ML1h07G00690	MlF3'5'H1	Eg02_t007770	EgF3'5'H2	0.01291102	0.045162796	0.285877339	10.03617679
ML1h04G03337	MlF3'5'H12	Eg09_t010710	EgF3'5'H10	0.022583935	0.04069856	0.554907474	9.044124427
ML1h04G02425	MlF3'5'H2	Eg09_t020640	EgF3'5'H1	0.024096666	0.068966544	0.34939646	15.32589862
ML1h07G00951	MlF3'5'H3	Eg02_t010650	EgF3'5'H5	0.008861423	0.032277505	0.274538678	7.172778783
ML1h08G03854	MlF3'5'H4	Eg07_t042500	EgF3'5'H4	0.017689499	0.069429744	0.254782723	15.42883202
ML1h06G02237	MlF3'5'H6	Eg06_t004020	EgF3'5'H3	0.022611436	0.099521676	0.22720112	22.11592797
ML1h07G01736	MlF3'5'H7	Eg02_t019070	EgF3'5'H6	0.023118078	0.097209379	0.237817364	21.60208421
ML1h07G01736	MlF3'5'H7	Eg02_t019180	EgF3'5'H9	0.095935569	0.430326585	0.222936653	95.62813004
ML1h07G02004	MlF3H1	Eg02_t022060	EgF3H2	0.010571827	0.063783435	0.165745656	14.17409675
ML1h08G02519	MlF3H2	Eg07_t027880	EgF3H1	0.011200837	0.103192792	0.108542824	22.93173148
ML1h03G02252	MlF3H3	Eg03_t018110	EgF3'H1	0.013015511	0.046707697	0.278658804	10.37948825
ML1h03G01030	MlFLS1	Eg03_t031640	EgFLS4	0.015877109	0.057423179	0.276493034	12.76070637
ML1h06G02043	MlFLS4	Eg01_t007540	EgFLS2	0.085980299	0.354362068	0.242634037	78.74712612

ML1h06G02043	MlFLS4	Eg06_t006150	EgFLS1	0.072917831	0.116560578	0.625578836	25.90235065
ML1h09G00678	MlFLS5	Eg01_t007540	EgFLS2	0.00854055	0.046355318	0.184240998	10.3011817
ML1h01G01259	MlLAR	Eg05_t019870	EgLAR	0.019209246	0.054129765	0.35487399	12.02883656
ML1h01G01735	MlLDOX	Eg05_t014690	EgLDOX	0.019529175	0.045343477	0.430694255	10.07632817
ML1h04G02672	MlPAL1	Eg05_t024420	EgPAL8	0.036274381	0.368810564	0.098355049	81.95790315
ML1h04G02672	MlPAL1	Eg09_t017980	EgPAL7	0.00437046	0.052482546	0.08327454	11.6627879
ML1h01G00856	MlPAL2	Eg05_t024420	EgPAL8	0.006260797	0.032839625	0.190647639	7.297694506
ML1h01G00856	MlPAL2	Eg09_t017980	EgPAL7	0.03735832	0.395393758	0.094483838	87.86527959
ML1h07G00086	MlPAL3	Eg02_t001010	EgPAL6	0.010654033	0.051744654	0.205896312	11.49881191
ML1h07G00086	MlPAL3	Eg02_t018790	EgPAL4	0.060040726	0.452258101	0.132757657	100.5018002
ML1h07G00086	MlPAL3	Eg09_t011330	EgPAL5	0.08367939	0.43435677	0.192651285	96.52372669
ML1h04G03282	MlPAL4	Eg09_t011330	EgPAL5	0.016562941	0.057739944	0.286854119	12.83109873
ML1h07G01701	MlPAL5	Eg02_t018790	EgPAL4	0.015103557	0.052971838	0.285124281	11.77151959
ML1h07G01701	MlPAL5	Eg02_t001010	EgPAL6	0.067895942	0.405963074	0.167246595	90.2140164
ML1h07G01701	MlPAL5	Eg09_t011330	EgPAL5	0.067114369	0.420507428	0.159603289	93.44609515
ML1h02G00424	MlPAL6	Eg01_t040920	EgPAL1	0.011894453	0.057274075	0.207676047	12.72757223
ML1h01G01719	MlPAL8	Eg05_t014880	EgPAL2	0.009368291	0.048032416	0.195041009	10.67387018
ML1h06G02093	MlPAL9	Eg06_t005550	EgPAL9	0.002943056	0.045296783	0.064972732	10.06595171
ML1h02G03012	MlTT8	Eg04_t020700	EgTT8	0.018037727	0.078340838	0.230246802	17.40907505
ML1h03G03510	MlTT8	Eg03_t004200	EgTT8.2	0.023274927	0.09188372	0.253308497	20.41860437
ML1h01G02333	MlTTG1	Eg05_t009200	EgTTG1	0.004714217	0.074272141	0.063472211	16.50492028
ML1h04G00438	MlTTG1	Eg09_t041330	EgTTG1.2	0.009913988	0.079986938	0.123945091	17.77487519
ML1h05G03318	MlUGT75C1	Eg08_t037040	EgUGT75C1.3	0.038625976	0.068466729	0.564156881	15.21482861
ML1h05G03317	MlUGT75C1.3	Eg08_t037030	EgUGT75C1.2	0.036043461	0.075969715	0.474445129	16.88215887
ML1h09G00476	MlUGT78D2	Eg01_t005290	EgUGT78D2.3	0.021946703	0.063775726	0.344123137	14.17238367

ML1h06G01843	MlUGT78D2	Eg06_t008220	EgUGT78D2.2	0.008881683	0.049532559	0.179309991	11.00723539
ML1h05G00763	MlUGT78D2.3	Eg08_t008340	EgUGT78D2	0.028141648	0.068516976	0.410725194	15.22599461
ML1h08G03185	MlUGT79B1	Eg07_t037900	EgUGT79B1	0.018758493	0.048063033	0.390289415	10.68067389

Table S4l. Divergence time, Ka, Ks and Ka/Ks values of orthologous flavonoid biosynthetic genes pairs between *M. lasiocarpa* and *M. acuminata*

**Seq_1**	**Name1**	**Seq_2**	**Name2**	**Ka**	**Ks**	**Ka_Ks**	**Diversity_year**
ML1h07G00167	Ml4CL1	Parent=Macma4_08_g13960	Ma4CL4	0.008386418	0.082516897	0.101632732	18.33708813
ML1h07G00248	Ml4CL10	Parent=Macma4_08_g13040	Ma4CL1	0.029365358	0.079211822	0.37071939	17.60262712
ML1h04G02613	Ml4CL11	Parent=Macma4_11_g22750	Ma4CL2.2	0.010239067	0.066273671	0.154496752	14.72748243
ML1h02G02377	Ml4CL13	Parent=Macma4_01_g19800	Ma4CL10	0.008192326	0.06777183	0.120880993	15.06040668
ML1h02G02377	Ml4CL13	Parent=Macma4_03_g13930	Ma4CL11	0.041444695	0.338817297	0.122321662	75.29273258
ML1h02G02377	Ml4CL13	Parent=Macma4_07_g05220	Ma4CL14	0.078577548	0.415155625	0.189272513	92.25680564
ML1h09G01309	Ml4CL14	Parent=Macma4_01_g19800	Ma4CL10	0.043578549	0.34511291	0.126273307	76.69175779
ML1h09G01309	Ml4CL14	Parent=Macma4_03_g13930	Ma4CL11	0.018958355	0.075934134	0.249668421	16.87425206
ML1h09G01309	Ml4CL14	Parent=Macma4_07_g05220	Ma4CL14	0.07836337	0.460836246	0.170046021	102.4080547
ML1h08G00557	Ml4CL15	Parent=Macma4_09_g24760	Ma4CL16	0.019915428	0.055981891	0.355747686	12.44042022
ML1h06G03426	Ml4CL16	Parent=Macma4_04_g13130	Ma4CL13	0.010720358	0.091178917	0.117574971	20.26198145
ML1h06G03426	Ml4CL16	Parent=Macma4_04_g06110	Ma4CL12	0.040096755	0.37818499	0.106024184	84.04110878
ML1h06G02761	Ml4CL17	Parent=Macma4_04_g06110	Ma4CL12	0.024268207	0.108834688	0.222982286	24.18548613
ML1h06G02761	Ml4CL17	Parent=Macma4_04_g13130	Ma4CL13	0.056483251	0.338582284	0.16682282	75.24050748
ML1h09G02144	Ml4CL18	Parent=Macma4_06_g39580	Ma4CL9	0.023578393	0.053338465	0.442052337	11.85299212
ML1h08G00140	Ml4CL2	Parent=Macma4_07_g18890	Ma4CL15	0.025643524	0.103207052	0.248466776	22.93490053
ML1h04G04085	Ml4CL3	Parent=Macma4_11_g06130	Ma4CL6	0.040373156	0.105896704	0.381250354	23.53260087
ML1h03G00142	Ml4CL4	Parent=Macma4_03_g14310	Ma4CL8	0.027443393	0.08570551	0.320205703	19.04566898

ML1h02G00664	Ml4CL5	Parent=Macma4_01_g07350	Ma4CL7	0.021879777	0.112347627	0.194750678	24.96613939
ML1h01G00405	Ml4CL8	Parent=Macma4_05_g28920	Ma4CL5	0.018436683	0.099018175	0.186194934	22.00403889
ML1h01G00972	Ml4CL9	Parent=Macma4_05_g22890	Ma4CL1.2	0.025044063	0.082203667	0.304658709	18.26748154
ML1h06G01342	MlANR2	Parent=Macma4_07_g10660	MaANR2	0.043325898	0.104110832	0.416151686	23.1357404
ML1h07G01339	MlANR4	Parent=Macma4_08_g01440	MaANR5	0.027053169	0.072735285	0.371940098	16.16339666
ML1h08G01785	MlC1	Parent=Macma4_09_g29590	MaC1.2	0.03876832	0.07100853	0.545967082	15.77967333
ML1h05G01322	MlC1.3	Parent=Macma4_10_g21630	MaC1	0.035234662	0.12844674	0.274313397	28.54372005
ML1h05G01754	MlC4H1	Parent=Macma4_06_g10450	MaC4H3	0.034332467	0.312700142	0.109793577	69.48892037
ML1h05G01754	MlC4H1	Parent=Macma4_10_g16840	MaC4H1	0.007013494	0.072240593	0.097085215	16.05346522
ML1h08G00252	MlC4H2	Parent=Macma4_07_g20380	MaC4H2	0.009129059	0.08042723	0.113507069	17.87271771
ML1h05G04363	MlC4H3	Parent=Macma4_06_g10450	MaC4H3	0.008770107	0.063362198	0.138412283	14.08048844
ML1h05G04363	MlC4H3	Parent=Macma4_10_g16840	MaC4H1	0.036210847	0.319391298	0.113374556	70.97584397
ML1h08G03392	MlC4H4	Parent=Macma4_09_g04800	MaC4H4	0.014876882	0.089681225	0.165886254	19.9291611
ML1h09G01419	MlCHI1	Parent=Macma4_04_g18950	MaCHI1	0.04247556	0.071803674	0.591551356	15.95637191
ML1h04G02595	MlCHI2	Parent=Macma4_11_g22930	MaCHI2	0.010258324	0.105502785	0.097232729	23.44506344
ML1h05G01818	MlCHS1	Parent=Macma4_06_g18170	MaCHS2	0.041169457	0.319572413	0.128826694	71.0160917
ML1h05G01818	MlCHS1	Parent=Macma4_10_g16140	MaCHS6	0.013587841	0.077607636	0.175083815	17.2461413
ML1h05G03634	MlCHS2	Parent=Macma4_06_g18170	MaCHS2	0.017609443	0.063965686	0.275295145	14.21459693
ML1h05G03634	MlCHS2	Parent=Macma4_06_g10010	MaCHS5	0.111157805	0.373548322	0.29757276	83.01073828
ML1h05G03634	MlCHS2	Parent=Macma4_10_g16140	MaCHS6	0.030917458	0.335608062	0.092123706	74.57956924
ML1h05G04408	MlCHS3	Parent=Macma4_06_g10010	MaCHS5	0.043947439	0.146382931	0.300222427	32.52954024
ML1h05G04408	MlCHS3	Parent=Macma4_06_g18170	MaCHS2	0.126755524	0.329464642	0.384731798	73.21436497
ML1h05G04408	MlCHS3	Parent=Macma4_10_g16140	MaCHS6	0.127290951	0.361301944	0.352311834	80.28932087
ML1h04G00849	MlDFR1	Parent=Macma4_04_g33220	MaDFR1	0.015251532	0.096834218	0.15750147	21.51871514
ML1h06G03238	MlDFR2	Parent=Macma4_04_g11180	MaDFR3	0.017016835	0.082963447	0.205112433	18.43632156

ML1h03G02164	MlDFR3	Parent=Macma4_03_g33910	MaDFR2	0.016017036	0.071987515	0.222497411	15.99722564
ML1h07G00690	MlF3'5'H1	Parent=Macma4_08_g08290	MaF3'5'H1	0.016920349	0.058677754	0.28836055	13.03950085
ML1h04G03337	MlF3'5'H12	Parent=Macma4_11_g15260	MaF3'5'H13	0.025649849	0.079629213	0.322116066	17.69538059
ML1h04G02425	MlF3'5'H2	Parent=Macma4_11_g05110	MaF3'5'H2	0.032383984	0.090292082	0.358658074	20.06490704
ML1h07G00951	MlF3'5'H3	Parent=Macma4_08_g05480	MaF3'5'H3	0.021407727	0.054894178	0.389981735	12.19870612
ML1h08G03854	MlF3'5'H4	Parent=Macma4_10_g01260	MaF3'5'H6	0.016837947	0.104599596	0.16097526	23.24435469
ML1h06G02237	MlF3'5'H6	Parent=Macma4_09_g17660	MaF3'5'H4	0.029881321	0.057330485	0.521211724	12.74010784
ML1h07G01736	MlF3'5'H7	Parent=Macma4_02_g03290	MaF3'5'H11	0.037235725	0.139733036	0.266477603	31.05178586
ML1h07G01736	MlF3'5'H7	Parent=Macma4_02_g03040	MaF3'5'H7	0.073799377	0.145464074	0.507337478	32.32534988
ML1h07G02004	MlF3H1	Parent=Macma4_02_g06130	MaF3H2	0.017761067	0.08069583	0.220098941	17.93240672
ML1h08G02519	MlF3H2	Parent=Macma4_07_g17110	MaF3H1	0.01657526	0.083752264	0.197908206	18.61161414
ML1h03G02252	MlF3H3	Parent=Macma4_03_g33170	MaF3'H	0.020962063	0.079229271	0.264574731	17.60650473
ML1h03G01030	MlFLS1	Parent=Macma4_08_g24690	MaFLS2	0.01988188	0.095366382	0.208478917	21.19252925
ML1h06G02043	MlFLS4	Parent=Macma4_03_g07220	MaFLS1	0.084518182	0.354203423	0.238614809	78.71187174
ML1h09G00678	MlFLS5	Parent=Macma4_03_g07220	MaFLS1	0.023221669	0.064772372	0.358511947	14.39386047
ML1h01G01259	MlLAR	Parent=Macma4_05_g19560	MaLAR	0.02062207	0.087487352	0.235714876	19.44163375
ML1h01G01735	MlLDOX	Parent=Macma4_05_g03920	MaLDOX	0.020728814	0.054227921	0.382253524	12.05064907
ML1h04G02672	MlPAL1	Parent=Macma4_05_g21700	MaPAL5	0.043754161	0.339985024	0.128694378	75.55222761
ML1h04G02672	MlPAL1	Parent=Macma4_11_g22170	MaPAL6	0.010639031	0.054809967	0.1941076	12.1799926
ML1h01G00856	MlPAL2	Parent=Macma4_05_g21700	MaPAL5	0.016351135	0.045096889	0.362577896	10.0215308
ML1h07G00086	MlPAL3	Parent=Macma4_02_g02760	MaPAL4	0.061181789	0.413009578	0.148136489	91.77990625
ML1h07G00086	MlPAL3	Parent=Macma4_08_g14800	MaPAL7	0.00938392	0.074986467	0.12514151	16.66365939
ML1h04G03282	MlPAL4	Parent=Macma4_02_g02760	MaPAL4	0.073162606	0.389712726	0.187734712	86.6028279
ML1h04G03282	MlPAL4	Parent=Macma4_11_g15930	MaPAL3	0.026905808	0.074487676	0.361211537	16.55281681
ML1h07G01701	MlPAL5	Parent=Macma4_02_g02760	MaPAL4	0.020881918	0.09426511	0.221523302	20.94780214

ML1h07G01701	MlPAL5	Parent=Macma4_08_g14800	MaPAL7	0.059995105	0.389509319	0.154027392	86.55762639
ML1h02G00424	MlPAL6	Parent=Macma4_01_g04800	MaPAL1	0.014724784	0.068864831	0.213821533	15.30329581
ML1h01G01719	MlPAL8	Parent=Macma4_05_g03790	MaPAL2	0.008736448	0.092870115	0.094071685	20.63780344
ML1h06G02093	MlPAL9	Parent=Macma4_09_g16190	MaPAL8	0.008863605	0.056596185	0.156611356	12.57692995
ML1h02G03012	MlTT8	Parent=Macma4_06_g25800	MaTT8	0.015649846	0.053509215	0.292470115	11.89093677
ML1h03G03510	MlTT8	Parent=Macma4_03_g19880	MaTT8.2	0.029538475	0.137283085	0.215164708	30.50735232
ML1h01G02333	MlTTG1	Parent=Macma4_05_g10420	MaTTG1	0.015397304	0.043968633	0.350188364	9.770807225
ML1h04G00438	MlTTG1	Parent=Macma4_04_g37870	MaTTG1.2	0.012807142	0.08982685	0.142575872	19.96152227
ML1h05G03318	MlUGT75C1	Parent=Macma4_06_g07210	MaUGT75C1.2	0.056531459	0.103420116	0.54661956	22.98224806
ML1h05G00327	MlUGT75C1	Parent=Macma4_10_g31950	MaUGT75C1	0.029492504	0.057873964	0.509598817	12.86088095
ML1h05G03317	MlUGT75C1.3	Parent=Macma4_06_g07200	MaUGT75C1.3	0.04952077	0.103683777	0.477613481	23.04083943
ML1h09G00476	MlUGT78D2	Parent=Macma4_03_g04880	MaUGT78D2.3	0.026516018	0.080604581	0.328964158	17.91212921
ML1h06G01843	MlUGT78D2	Parent=Macma4_07_g16200	MaUGT78D2.2	0.020902074	0.077009636	0.271421546	17.11325234
ML1h05G00763	MlUGT78D2.3	Parent=Macma4_10_g27580	MaUGT78D2	0.03869547	0.11667959	0.331638721	25.92879775
ML1h08G03185	MlUGT79B1	Parent=Macma4_09_g07100	MaUGT79B1	0.027207258	0.083815005	0.32461083	18.6255566

Table S4m. Divergence time, Ka, Ks and Ka/Ks values of orthologous flavonoid biosynthetic genes pairs between *M. lasiocarpa* and *M. balbisiana*

Seq_1	Name1	Seq_2	Name2	Ka	Ks	Ka_Ks	Diversity_year
ML1h07G00167	Ml4CL1	Mba08_g13620	Mb4CL4	0.01553557	0.100872186	0.154012422	22.41604138
ML1h07G00248	Ml4CL10	Mba08_g12720	Mb4CL1.2	0.02800495	0.092752812	0.301931014	20.61173596
ML1h04G02613	Ml4CL11	Mba11_g20940	Mb4CL2.2	0.014219371	0.056021163	0.253821423	12.44914734
ML1h02G02377	Ml4CL13	Mba01_g28370	Mb4CL10	0.00737157	0.07827151	0.094179476	17.39366889
ML1h09G01309	Ml4CL14	Mba01_g28370	Mb4CL10	0.043737653	0.355532462	0.12302014	79.00721369
ML1h08G00557	Ml4CL15	Mba04_g12340	Mb4CL12	0.052984387	0.485221546	0.109196278	107.8270102

ML1h06G03426	Ml4CL16	Mba04_g12340	Mb4CL12	0.012379923	0.105251203	0.117622631	23.38915632
ML1h06G03426	Ml4CL16	Mba04_g05720	Mb4CL11	0.043570186	0.366331946	0.118936353	81.40709917
ML1h06G02761	Ml4CL17	Mba04_g05720	Mb4CL11	0.028530919	0.09230584	0.309091157	20.51240887
ML1h06G02761	Ml4CL17	Mba04_g12340	Mb4CL12	0.058237564	0.358366037	0.162508603	79.63689714
ML1h09G02144	Ml4CL18	Mba06_g36630	Mb4CL9	0.02481616	0.052149585	0.475864952	11.58879662
ML1h08G00140	Ml4CL2	Mba07_g17040	Mb4CL14	0.026487338	0.120995616	0.218911549	26.88791474
ML1h03G00142	Ml4CL4	Mba03_g13490	Mb4CL8	0.047463818	0.109910569	0.431840345	24.42457085
ML1h02G00664	Ml4CL5	Mba01_g16650	Mb4CL7	0.011057196	0.081057297	0.136412104	18.01273272
ML1h01G00405	Ml4CL8	Mba05_g27160	Mb4CL5	0.022700346	0.098898578	0.229531569	21.97746173
ML1h01G00972	Ml4CL9	Mba05_g21540	Mb4CL1	0.026771751	0.081940287	0.326722693	18.20895275
ML1h07G01339	MlANR4	Mba08_g01400	MbANR	0.025709458	0.06878132	0.373785472	15.28473775
ML1h08G01785	MlC1	Mba09_g23680	MbC1.3	0.173514535	0.216601891	0.801075812	48.13375346
ML1h05G03234	MlC1	Mba06_g05630	MbC1.2	0.029110243	0.100381601	0.289995806	22.3070225
ML1h05G01322	MlC1.3	Mba10_g15520	MbC1	0.035286476	0.140004964	0.252037318	31.11221419
ML1h05G01754	MlC4H1	Mba06_g09560	MbC4H3	0.031625826	0.332907993	0.094998697	73.97955411
ML1h08G00252	MlC4H2	Mba07_g18380	MbC4H4	0.006377202	0.083764649	0.07613238	18.61436655
ML1h05G04363	MlC4H3	Mba06_g09560	MbC4H3	0.00789481	0.075280111	0.10487245	16.72891363
ML1h08G03392	MlC4H4	Mba09_g04380	MbC4H1	0.022873996	0.086512485	0.264401101	19.22499668
ML1h09G01419	MlCHI1	Mba04_g18040	MbCHI2	0.066382369	0.119540262	0.555313901	26.56450256
ML1h04G02595	MlCHI2	Mba11_g21110	MbCHI1	0.012446267	0.106575428	0.116783642	23.68342846
ML1h05G01818	MlCHS1	Mba06_g16830	MbCHS2	0.033881359	0.331520521	0.102199885	73.67122693
ML1h05G01818	MlCHS1	Mba10_g10820	MbCHS8	0.011309233	0.093258753	0.121267258	20.72416727
ML1h05G03634	MlCHS2	Mba06_g16830	MbCHS2	0.010172521	0.077488211	0.131278306	17.21960234
ML1h05G03634	MlCHS2	Mba10_g10820	MbCHS8	0.028591226	0.335236646	0.085286698	74.49703249
ML1h05G04408	MlCHS3	Mba06_g09120	MbCHS7	0.038595901	0.098214943	0.39297382	21.82554278

ML1h05G04408	MlCHS3	Mba06_g16830	MbCHS2	0.119451815	0.337905479	0.353506593	75.09010649
ML1h05G04408	MlCHS3	Mba10_g10820	MbCHS8	0.123350065	0.366592145	0.336477655	81.46492119
ML1h04G00849	MlDFR1	Mba04_g31800	MbDFR1	0.013507794	0.093563779	0.144369909	20.79195083
ML1h03G02164	MlDFR3	Mba01_g01310	MbDFR2	0.01350973	0.054703524	0.246962696	12.15633861
ML1h07G00690	MlF3'5'H1	Mba08_g07950	MbF3'5'H1	0.013901929	0.065561783	0.212043179	14.56928508
ML1h04G03337	MlF3'5'H12	Mba11_g13650	MbF3'5'H13	0.022643229	0.096648269	0.234284892	21.47739308
ML1h04G02425	MlF3'5'H2	Mba11_g04840	MbF3'5'H2	0.030395221	0.12074783	0.25172478	26.83285111
ML1h08G03854	MlF3'5'H4	Mba10_g00740	MbF3'5'H4	0.018212544	0.088935235	0.204784346	19.76338545
ML1h06G02237	MlF3'5'H6	Mba09_g16030	MbF3'5'H3	0.029021478	0.062827412	0.461923813	13.96164718
ML1h07G01736	MlF3'5'H7	Mba02_g01950	MbF3'5'H12	0.092941402	0.445007983	0.208853336	98.89066294
ML1h07G02004	MlF3H1	Mba02_g04670	MbF3H1	0.015365454	0.063889622	0.240500002	14.19769374
ML1h08G02519	MlF3H2	Mba07_g15320	MbF3H2	0.017769834	0.10522276	0.168878237	23.38283552
ML1h03G02252	MlF3H3	Mba01_g02030	MbF3'H	0.021845144	0.076482549	0.285622596	16.9961219
ML1h03G01030	MlFLS1	Mba08_g23630	MbFLS1	0.017525441	0.129463865	0.135369367	28.76974782
ML1h06G02043	MlFLS4	Mba03_g06940	MbFLS4	0.086070159	0.346259135	0.248571518	76.94647449
ML1h06G02043	MlFLS4	Mba09_g14060	MbFLS5	0.070790543	0.151310838	0.46784846	33.62463072
ML1h09G00678	MlFLS5	Mba03_g06940	MbFLS4	0.019877489	0.048475018	0.410056342	10.77222622
ML1h09G00678	MlFLS5	Mba09_g14060	MbFLS5	0.160871935	0.47849146	0.336206492	106.3314356
ML1h01G01259	MlLAR	Mba05_g18490	MbLAR	0.028887597	0.072151723	0.400372934	16.03371623
ML1h01G01735	MlLDOX	Mba05_g13780	MbLDOX	0.026947173	0.071633353	0.376181927	15.91852295
ML1h04G02672	MlPAL1	Mba11_g20430	MbPAL2	0.01189942	0.052799121	0.225371553	11.73313789
ML1h07G00086	MlPAL3	Mba08_g14430	MbPAL3	0.009384898	0.074961126	0.125196869	16.658028
ML1h04G03282	MlPAL4	Mba11_g14220	MbPAL6	0.036644975	0.09750796	0.375815215	21.66843551
ML1h07G01701	MlPAL5	Mba02_g01510	MbPAL5	0.054050785	0.110011401	0.491319847	24.44697801
ML1h07G01701	MlPAL5	Mba08_g14430	MbPAL3	0.060001618	0.389345547	0.15410891	86.5212327

ML1h07G01701	MlPAL5	Mba11_g14220	MbPAL6	0.068781133	0.460907277	0.149229869	102.4238393
ML1h02G00424	MlPAL6	Mba01_g14280	MbPAL8	0.012518546	0.061501724	0.203547895	13.66704973
ML1h01G01719	MlPAL8	Mba05_g13940	MbPAL7	0.014404701	0.09511955	0.151437864	21.13767777
ML1h06G02093	MlPAL9	Mba09_g14610	MbPAL1	0.009458719	0.064210315	0.147308411	14.26895879
ML1h02G03012	MlTT8	Mba06_g23950	MbTT8	0.079639792	0.131859868	0.603972939	29.30219288
ML1h03G03510	MlTT8	Mba03_g18730	MbTT8.2	0.10088637	0.167042755	0.60395538	37.12061218
ML1h03G03510	MlTT8	Mba06_g23950	MbTT8	0.110357306	0.439747898	0.250955846	97.72175511
ML1h01G02333	MlTTG1	Mba05_g07670	MbTTG1	0.017799708	0.051506596	0.345581139	11.44591026
ML1h04G00438	MlTTG1	Mba04_g36060	MbTTG1.2	0.012767775	0.0942591	0.13545403	20.9464667
ML1h05G03318	MlUGT75C1	Mba06_g06490	MbUGT75C1.3	0.0581719	0.090908254	0.639896793	20.20183416
ML1h05G00327	MlUGT75C1	Mba10_g24560	MbUGT75C1	0.029042029	0.050209949	0.578411838	11.15776644
ML1h09G00476	MlUGT78D2	Mba03_g04830	MbUGT78D2	0.022449298	0.08998993	0.249464558	19.9977623
ML1h06G01843	MlUGT78D2	Mba07_g14580	MbUGT78D2.3	0.01987694	0.064793764	0.306772418	14.39861429
ML1h05G00763	MlUGT78D2.3	Mba10_g20800	MbUGT78D2.2	0.028071107	0.084917665	0.330568523	18.87059224

Table S4n. Divergence time, Ka, Ks and Ka/Ks values of orthologous flavonoid biosynthetic genes pairs between *M. lasiocarpa* and *M. beccarii*

Seq_1	Name1	Seq_2	Name2	Ka	Ks	Ka_Ks	Diversity_year
ML1h07G00167	Ml4CL1	Mbe07_t014630	Mbe4CL3	0.010076423	0.09251595	0.108915526	20.55910003
ML1h07G00248	Ml4CL10	Mbe07_t013640	Mbe4CL10	0.03940911	0.104750957	0.376217182	23.27799037
ML1h04G02613	Ml4CL11	Mbe06_t040140	Mbe4CL12	0.015012353	0.097719325	0.153627266	21.71540549
ML1h07G03382	Ml4CL12	Mbe02_t027270	Mbe4CL11	0.031972054	0.098930023	0.323178477	21.98444948
ML1h02G02377	Ml4CL13	Mbe01_t005810	Mbe4CL14	0.010669765	0.070359151	0.15164715	15.63536697
ML1h02G02377	Ml4CL13	Mbe03_t000920	Mbe4CL15	0.04276394	0.336168407	0.127209871	74.70409045
ML1h02G02377	Ml4CL13	Mbe07_t022330	Mbe4CL16	0.070413997	0.402578179	0.174907634	89.46181758
ML1h09G01309	Ml4CL14	Mbe01_t005810	Mbe4CL14	0.044883393	0.370013245	0.121302126	82.22516554

ML1h09G01309	Ml4CL14	Mbe03_t000920	Mbe4CL15	0.013145619	0.081125862	0.16203981	18.02796941
ML1h09G01309	Ml4CL14	Mbe07_t022330	Mbe4CL16	0.070658987	0.463091322	0.152581107	102.9091826
ML1h08G00557	Ml4CL15	Mbe03_t056570	Mbe4CL13	0.014908352	0.066327453	0.224768951	14.73943396
ML1h08G00557	Ml4CL15	Mbe04_t013920	Mbe4CL18	0.049930312	0.48232639	0.103519759	107.1836422
ML1h06G03426	Ml4CL16	Mbe04_t013920	Mbe4CL18	0.009890253	0.113673574	0.087005738	25.26079414
ML1h06G03426	Ml4CL16	Mbe04_t006230	Mbe4CL17	0.041650014	0.356321178	0.116888966	79.18248398
ML1h06G02761	Ml4CL17	Mbe04_t006230	Mbe4CL17	0.030218103	0.092543426	0.326528899	20.56520588
ML1h06G02761	Ml4CL17	Mbe04_t013920	Mbe4CL18	0.052074284	0.369884371	0.140785304	82.19652681
ML1h09G02144	Ml4CL18	Mbe06_t016280	Mbe4CL1	0.028985114	0.109919794	0.263693309	24.42662081
ML1h08G00140	Ml4CL2	Mbe05_t019740	Mbe4CL4	0.023156687	0.105292316	0.219927611	23.39829241
ML1h04G04085	Ml4CL3	Mbe03_t020730	Mbe4CL8	0.040761295	0.077205453	0.527958761	17.15676723
ML1h03G00142	Ml4CL4	Mbe03_t000510	Mbe4CL2	0.034655288	0.083689314	0.414094534	18.59762536
ML1h02G00664	Ml4CL5	Mbe01_t019320	Mbe4CL6	0.03139534	0.09560869	0.328373286	21.24637554
ML1h01G00405	Ml4CL8	Mbe05_t004680	Mbe4CL7	0.021428529	0.110480097	0.19395827	24.5511327
ML1h01G00972	Ml4CL9	Mbe05_t011100	Mbe4CL9	0.027623964	0.057975524	0.476476309	12.88344976
ML1h06G01342	MlANR2	Mbe07_t028460	MbeANR	0.03809047	0.133636416	0.285030618	29.69698136
ML1h08G01785	MlC1	Mbe03_t051550	MbeC1	0.044136742	0.07077383	0.623630828	15.72751775
ML1h05G03234	MlC1	Mbe01_t033880	MbeC1	0.023385848	0.115017234	0.203324732	25.55938543
ML1h05G01322	MlC1.3	Mbe02_t039140	MbeC1.3	0.022176906	0.12341204	0.179698075	27.42489784
ML1h05G01754	MlC4H1	Mbe01_t038520	MbeC4H1	0.032982141	0.326864996	0.100904477	72.63666589
ML1h05G01754	MlC4H1	Mbe02_t033650	MbeC4H3	0.007895964	0.078294106	0.100850046	17.39869011
ML1h08G00252	MlC4H2	Mbe10_t000860	MbeC4H2	0.007299883	0.060931741	0.11980427	13.54038692
ML1h05G04363	MlC4H3	Mbe01_t038520	MbeC4H1	0.006131421	0.075352898	0.081369412	16.74508839
ML1h05G04363	MlC4H3	Mbe02_t033650	MbeC4H3	0.036216244	0.327677441	0.11052407	72.81720905
ML1h08G03392	MlC4H4	Mbe08_t025090	MbeC4H4	0.016649095	0.09281701	0.179375478	20.62600221

ML1h09G01419	MlCHI1	Mbe04_t020690	MbeCHI1	0.045762577	0.086204466	0.530860854	19.15654808
ML1h04G02595	MlCHI2	Mbe06_t039860	MbeCHI2	0.02513763	0.10603639	0.237066065	23.56364215
ML1h05G01818	MlCHS1	Mbe01_t047580	MbeCHS4	0.035033644	0.337861541	0.103692311	75.08034245
ML1h05G01818	MlCHS1	Mbe01_t038030	MbeCHS2	0.112735691	0.407157206	0.276884922	90.47937908
ML1h05G01818	MlCHS1	Mbe11_t002790	MbeCHS5	0.013577591	0.0739059	0.183714578	16.4235334
ML1h05G03634	MlCHS2	Mbe01_t047580	MbeCHS4	0.012443724	0.06605734	0.188377618	14.67940887
ML1h05G03634	MlCHS2	Mbe01_t038030	MbeCHS2	0.109445378	0.352444381	0.310532339	78.32097359
ML1h05G03634	MlCHS2	Mbe11_t002790	MbeCHS5	0.031478154	0.312177109	0.100834279	69.37269091
ML1h05G04408	MlCHS3	Mbe01_t038030	MbeCHS2	0.035270178	0.139237302	0.253309834	30.94162258
ML1h05G04408	MlCHS3	Mbe01_t047580	MbeCHS4	0.123324989	0.344314185	0.358175743	76.51426331
ML1h05G04408	MlCHS3	Mbe11_t002790	MbeCHS5	0.123873307	0.348262531	0.355689447	77.39167362
ML1h04G00849	MlDFR1	Mbe04_t036630	MbeDFR1	0.019729606	0.075405922	0.261645313	16.75687154
ML1h06G03238	MlDFR2	Mbe04_t011700	MbeDFR3	0.017047918	0.077851607	0.218979656	17.30035715
ML1h03G02164	MlDFR3	Mbe06_t019010	MbeDFR2	0.021045326	0.126661846	0.16615363	28.14707693
ML1h07G00690	MlF3'5'H1	Mbe07_t008510	MbeF3'5'H1	0.018928449	0.045296783	0.417876244	10.06595171
ML1h04G03337	MlF3'5'H12	Mbe06_t049500	MbeF3'5'H2	0.026599406	0.076785167	0.346413334	17.06337046
ML1h07G00951	MlF3'5'H3	Mbe07_t005300	MbeF3'5'H10	0.048966095	0.088483743	0.553390869	19.66305394
ML1h08G03854	MlF3'5'H4	Mbe09_t008950	MbeF3'5'H8	0.019460073	0.102504679	0.189845704	22.77881766
ML1h06G02237	MlF3'5'H6	Mbe08_t039500	MbeF3'5'H7	0.040042495	0.068595224	0.583750485	15.243383
ML1h06G02237	MlF3'5'H6	Mbe09_t008950	MbeF3'5'H8	0.12945665	0.450429109	0.287407379	100.0953575
ML1h07G01736	MlF3'5'H7	Mbe02_t005590	MbeF3'5'H3	0.093553434	0.402936374	0.232179173	89.54141643
ML1h07G01736	MlF3'5'H7	Mbe02_t006410	MbeF3'5'H5	0.023102671	0.091324072	0.252974602	20.29423811
ML1h08G02519	MlF3H2	Mbe07_t035880	MbeF3H1	0.016578532	0.083696573	0.19807898	18.59923837
ML1h03G02252	MlF3H3	Mbe06_t019910	MbeF3'H	0.039803002	0.116291814	0.342268304	25.8426253
ML1h03G01030	MlFLS1	Mbe08_t015900	MbeFLS1	0.02525715	0.081160188	0.31120122	18.03559744

ML1h06G02043	MlFLS4	Mbe03_t008100	MbeFLS4	0.083812345	0.370742318	0.226066303	82.38718168
ML1h06G02043	MlFLS4	Mbe08_t036240	MbeFLS3	0.071015216	0.151376519	0.469129667	33.63922653
ML1h09G00678	MlFLS5	Mbe03_t008100	MbeFLS4	0.010529953	0.076441995	0.137750885	16.98711006
ML1h01G01259	MlLAR	Mbe05_t014700	MbeLAR	0.028341715	0.099615685	0.284510566	22.13681884
ML1h01G01735	MlLDOX	Mbe03_t031300	MbeLDOX	0.025669648	0.063106582	0.406766562	14.02368496
ML1h04G02672	MlPAL1	Mbe05_t009880	MbePAL1	0.03823436	0.353889393	0.108040425	78.64208744
ML1h04G02672	MlPAL1	Mbe06_t040740	MbePAL2	0.013181634	0.060678659	0.217236747	13.48414634
ML1h01G00856	MlPAL2	Mbe05_t009880	MbePAL1	0.010671869	0.052893976	0.201759626	11.75421678
ML1h01G00856	MlPAL2	Mbe06_t040740	MbePAL2	0.035481209	0.346049955	0.102532042	76.89999001
ML1h07G00086	MlPAL3	Mbe05_t009880	MbePAL1	0.058552059	0.438930994	0.133396956	97.54022078
ML1h07G00086	MlPAL3	Mbe06_t048840	MbePAL5	0.084132775	0.41693586	0.201788292	92.65241337
ML1h07G00086	MlPAL3	Mbe07_t015550	MbePAL3	0.013189914	0.089628387	0.147162236	19.91741943
ML1h04G03282	MlPAL4	Mbe06_t048840	MbePAL5	0.028331068	0.049645911	0.570662669	11.03242478
ML1h07G01701	MlPAL5	Mbe07_t015550	MbePAL3	0.061111894	0.363372944	0.16817954	80.749543
ML1h02G00424	MlPAL6	Mbe01_t022110	MbePAL7	0.013468727	0.068727549	0.195972752	15.27278869
ML1h06G02093	MlPAL9	Mbe08_t037020	MbePAL8	0.008271464	0.056553144	0.146260017	12.56736541
ML1h02G03012	MlTT8	Mbe06_t001720	MbeTT8	0.04835441	0.097771363	0.494566181	21.72696964
ML1h03G03510	MlTT8	Mbe06_t034420	MbeTT8	0.025297219	0.143805131	0.175913191	31.95669587
ML1h01G02333	MlTTG1	Mbe03_t038300	MbeTTG1	0.013597771	0.080829318	0.168228206	17.96207073
ML1h04G00438	MlTTG1	Mbe04_t041380	MbeTTG1	0.011371931	0.085102379	0.133626473	18.91163975
ML1h05G03318	MlUGT75C1	Mbe01_t034870	MbeUGT75C1.3	0.155528597	0.406098334	0.382982603	90.24407426
ML1h05G00327	MlUGT75C1	Mbe02_t051150	MbeUGT75C1	0.028561281	0.072766674	0.392504966	16.17037195
ML1h09G00476	MlUGT78D2	Mbe03_t010580	MbeUGT78D2.3	0.01817701	0.086440298	0.210283982	19.20895504
ML1h06G01843	MlUGT78D2	Mbe07_t034770	MbeUGT78D2	0.020363804	0.054236077	0.375466014	12.05246159
ML1h05G00763	MlUGT78D2.3	Mbe02_t045890	MbeUGT78D2	0.030101858	0.09475826	0.317670019	21.057391

ML1h08G03185	MlUGT79B1	Mbe08_t027530	MbeUGT79B1	0.034016681	0.086598694	0.392808247	19.24415425

Table S4h. Divergence time, Ka, Ks and Ka/Ks values of orthologous flavonoid biosynthetic genes pairs between *M. lasiocarpa* and *M. schizocarpa*

Seq_1	Name1	Seq_2	Name2	Ka	Ks	Ka_Ks	Diversity_year
ML1h07G00167	Ml4CL1	Ms08t219500	Ms4CL4	0.00923024	0.090031048	0.102522857	20.00689963
ML1h07G00248	Ml4CL10	Ms08t218760	Ms4CL1	0.030282406	0.079142102	0.382633336	17.58713375
ML1h04G02613	Ml4CL11	Ms11t319670	Ms4CL2.2	0.011032508	0.063711756	0.173162829	14.1581679
ML1h07G03382	Ml4CL12	Ms02t041000	Ms4CL2	0.025954871	0.084858624	0.305860146	18.8574721
ML1h02G02377	Ml4CL13	Ms01t017810	Ms4CL10	0.008196803	0.080886391	0.101337231	17.97475353
ML1h02G02377	Ml4CL13	Ms03t058920	Ms4CL11	0.040567379	0.343122745	0.118229933	76.24949899
ML1h02G02377	Ml4CL13	Ms07t183930	Ms4CL14	0.078959606	0.42731524	0.184780693	94.95894222
ML1h09G01309	Ml4CL14	Ms01t017810	Ms4CL10	0.043602916	0.359772179	0.121195909	79.94937311
ML1h09G01309	Ml4CL14	Ms03t058920	Ms4CL11	0.018116574	0.081384019	0.222606041	18.08533755
ML1h09G01309	Ml4CL14	Ms07t183930	Ms4CL14	0.077380379	0.458010428	0.168948945	101.7800951
ML1h08G00557	Ml4CL15	Ms04t089570	Ms4CL13	0.052121214	0.457085002	0.114029587	101.5744449
ML1h08G00557	Ml4CL15	Ms09t262230	Ms4CL16	0.023275401	0.053423722	0.435675395	11.87193821
ML1h06G03426	Ml4CL16	Ms04t089570	Ms4CL13	0.01238333	0.096739646	0.128006777	21.49769919
ML1h06G03426	Ml4CL16	Ms04t083350	Ms4CL12	0.043113495	0.38911356	0.110799262	86.46968005
ML1h06G02761	Ml4CL17	Ms04t083350	Ms4CL12	0.027662561	0.111822524	0.247379154	24.84944978
ML1h06G02761	Ml4CL17	Ms04t089570	Ms4CL13	0.058254122	0.346286182	0.168225371	76.95248487
ML1h09G02144	Ml4CL18	Ms06t178240	Ms4CL9	0.025259443	0.064217331	0.393343088	14.2705181
ML1h08G00140	Ml4CL2	Ms07t196560	Ms4CL15	0.033545964	0.116311659	0.288414458	25.84703538
ML1h03G00142	Ml4CL4	Ms03t059300	Ms4CL8	0.031069531	0.080762548	0.384702218	17.94723285
ML1h02G00664	Ml4CL5	Ms01t006680	Ms4CL7	0.018955751	0.097798566	0.193824427	21.73301475

ML1h01G00405	Ml4CL8	Ms05t142250	Ms4CL5	0.020989197	0.096402062	0.217725601	21.42268035
ML1h01G00972	Ml4CL9	Ms05t136800	Ms4CL1.2	0.022515762	0.084759757	0.265642127	18.8355016
ML1h06G01342	MlANR2	Ms07t188910	MsANR3	0.041563768	0.102780639	0.404392968	22.84014204
ML1h07G01339	MlANR4	Ms08t207990	MsANR1	0.030992143	0.068550033	0.452109826	15.23334062
ML1h08G01785	MlC1	Ms09t266370	MsC1	0.045895142	0.070907754	0.647251383	15.75727861
ML1h05G03234	MlC1	Ms06t151760	MsC1.2	0.030777702	0.088377986	0.348250778	19.63955236
ML1h05G01754	MlC4H1	Ms10t281630	MsC4H1	0.008782302	0.078180649	0.112333446	17.37347761
ML1h08G00252	MlC4H2	Ms07t197940	MsC4H2	0.009129059	0.083699665	0.109069243	18.59992549
ML1h05G04363	MlC4H3	Ms10t281630	MsC4H1	0.033031223	0.324991547	0.101637177	72.22034377
ML1h08G03392	MlC4H4	Ms09t244050	MsC4H3	0.016649095	0.086469445	0.192543106	19.21543221
ML1h09G01419	MlCHI1	Ms04t093250	MsCHI2	0.040556944	0.071447141	0.567649637	15.87714247
ML1h04G02595	MlCHI2	Ms11t319860	MsCHI1	0.021357434	0.119111687	0.179305946	26.46926375
ML1h05G01818	MlCHS1	Ms06t158650	MsCHS2	0.045443547	0.35108469	0.129437563	78.01881991
ML1h05G01818	MlCHS1	Ms10t280970	MsCHS1	0.013587841	0.085435496	0.159042104	18.98566568
ML1h05G03634	MlCHS2	Ms06t158650	MsCHS2	0.021695138	0.089491504	0.242426788	19.88700081
ML1h05G03634	MlCHS2	Ms10t280970	MsCHS1	0.030917458	0.346683482	0.08918065	77.04077379
ML1h05G04408	MlCHS3	MsSC51t327380	MsCHS4	0.030420239	0.122722068	0.247879124	27.27157063
ML1h05G04408	MlCHS3	Ms06t158650	MsCHS2	0.130421122	0.344569991	0.378504006	76.57110904
ML1h05G04408	MlCHS3	Ms10t280970	MsCHS1	0.125962439	0.372756191	0.337921789	82.8347092
ML1h04G00849	MlDFR1	Ms04t107810	MsDFR1	0.016519092	0.082525913	0.200168547	18.33909185
ML1h06G03238	MlDFR2	Ms04t087800	MsDFR2	0.014813772	0.079750456	0.18575157	17.72232354
ML1h03G02164	MlDFR3	Ms03t076720	MsDFR3	0.018504228	0.067651466	0.273522941	15.0336592
ML1h07G00690	MlF3'5'H1	Ms08t214430	MsF3'5'H1	0.019552574	0.060069614	0.325498572	13.3488032
ML1h04G03337	MlF3'5'H12	Ms11t312500	MsF3'5'H8	0.022633944	0.09334291	0.242481663	20.74286885
ML1h07G00951	MlF3'5'H3	Ms08t211840	MsF3'5'H3	0.018696717	0.049231614	0.379770549	10.9403587

ML1h06G02237	MlF3'5'H6	Ms09t255750	MsF3'5'H2	0.029069712	0.054920671	0.529303666	12.20459354
ML1h07G01736	MlF3'5'H7	Ms02t024400	MsF3'5'H5	0.039092406	0.129935677	0.300859681	28.87459479
ML1h07G01736	MlF3'5'H7	Ms11t312500	MsF3'5'H8	0.148718793	0.639189586	0.232667735	142.0421301
ML1h07G02004	MlF3H1	Ms02t027200	MsF3H	0.015969061	0.095690424	0.166882536	21.26453865
ML1h03G02252	MlF3H3	Ms03t075990	MsF3'H	0.024525943	0.070810361	0.346360936	15.7356357
ML1h03G01030	MlFLS1	Ms08t229420	MsFLS1	0.017177584	0.090951571	0.188865173	20.21146019
ML1h06G02043	MlFLS4	Ms03t052640	MsFLS2	0.085990274	0.354203423	0.242770873	78.71187174
ML1h09G00678	MlFLS5	Ms03t052640	MsFLS2	0.025921942	0.064772372	0.4002006	14.39386047
ML1h01G01259	MlLAR	Ms05t133790	MsLAR	0.035825609	0.094112368	0.380668452	20.9138596
ML1h01G01735	MlLDOX	Ms05t120140	MsLDOX	0.020733028	0.045594147	0.454730023	10.1320327
ML1h04G02672	MlPAL1	Ms05t135820	MsPAL7	0.040838047	0.345826936	0.118088103	76.85043015
ML1h04G02672	MlPAL1	Ms11t319150	MsPAL6	0.009382452	0.05475635	0.171349118	12.1680778
ML1h01G00856	MlPAL2	Ms05t135820	MsPAL7	0.013829622	0.057095449	0.242219336	12.6878776
ML1h01G00856	MlPAL2	Ms11t319150	MsPAL6	0.034682727	0.350899187	0.098839575	77.97759704
ML1h07G00086	MlPAL3	Ms02t024050	MsPAL4	0.062183002	0.392279352	0.158517143	87.17318925
ML1h07G00086	MlPAL3	Ms05t135820	MsPAL7	0.062454791	0.417082356	0.149742108	92.68496804
ML1h07G00086	MlPAL3	Ms08t220210	MsPAL5	0.011903147	0.085793141	0.138742412	19.06514241
ML1h04G03282	MlPAL4	Ms02t024050	MsPAL4	0.074883672	0.382188697	0.195933769	84.93082147
ML1h04G03282	MlPAL4	Ms11t313180	MsPAL3	0.027277344	0.078423409	0.347821446	17.42742432
ML1h07G01701	MlPAL5	Ms02t024050	MsPAL4	0.021517059	0.077436879	0.277865779	17.20819535
ML1h07G01701	MlPAL5	Ms08t220210	MsPAL5	0.062240548	0.415180286	0.1499121	92.26228588
ML1h02G00424	MlPAL6	Ms01t004140	MsPAL1	0.014089331	0.064738241	0.217635369	14.38627586
ML1h01G01719	MlPAL8	Ms05t120010	MsPAL2	0.00936147	0.088580192	0.105683555	19.68448705
ML1h06G02093	MlPAL9	Ms09t254450	MsPAL8	0.008864915	0.060370836	0.146841015	13.4157413
ML1h03G03510	MlTT8	Ms03t064490	MsTT8.2	0.02817784	0.14049816	0.200556645	31.22181324

ML1h01G02333	MlTTG1	Ms05t125970	MsTTG1.2	0.011363854	0.046346789	0.245191822	10.29928654
ML1h04G00438	MlTTG1	Ms04t112120	MsTTG1	0.012807142	0.075647617	0.169299999	16.81058163
ML1h05G03318	MlUGT75C1	Ms06t152730	MsUGT75C1.3	0.054437732	0.103530107	0.525815472	23.00669043
ML1h05G00327	MlUGT75C1	Ms10t295740	MsUGT75C1.4	0.028494373	0.057058946	0.499384843	12.67976571
ML1h09G00476	MlUGT78D2	Ms03t050760	MsUGT78D2.3	0.029528418	0.081000663	0.364545382	18.00014729
ML1h06G01843	MlUGT78D2	Ms07t193970	MsUGT78D2.2	0.026196937	0.068347186	0.383292112	15.1882636
ML1h05G00763	MlUGT78D2.3	Ms10t291590	MsUGT78D2	0.039014596	0.115802655	0.336905886	25.73392325
ML1h08G03185	MlUGT79B1	Ms09t246270	MsUGT79B1	0.025212458	0.08083589	0.311896831	17.96353121

Table S5a. Tandem duplication FBG pairs in Musaceae

Gene1	Name1	Gene2	Name2	Gene3	Name3	Gene4	Name4
M. beccarii							
Mbe01_t034870	***MbeUGT75C1.3***	Mbe01_t034880	***MbeUGT75C1.2***				
Mbe02_t005590	***MbeF3'5'H3***	Mbe02_t005600	***MbeF3'5'H6***				
Mbe09_t008950	***MbeF3'5'H8***	Mbe09_t008960	***MbeF3'5'H9***				
M. lasiocarpa							
ML1h01G01720	***MlPAL7***	ML1h01G01719	***MlPAL8***				
ML1h05G00635	***MlFLS3***	ML1h05G00636	***MlFLS2***				
ML1h05G03317	***MlUGT75C1.3***	ML1h05G03318	***MlUGT75C1.2***				
ML1h06G01340	***MlANR1***	ML1h06G01342	***MlANR2***	ML1h06G01341	***MlANR3***		
ML1h07G01736	***MlF3'5'H7***	ML1h07G01737	***MlF3'5'H8***				
E. glaucum							
Eg05_t014870	***EgPAL3***	Eg05_t014880	***EgPAL2***				
Eg08_t037030	***EgUGT75C1.2***	Eg08_t037040	***EgUGT75C1.3***				
Eg08_t049390	***EgCHS4***	Eg08_t049400	***EgCHS5***	Eg08_t049410	***EgCHS6***		
M. acuminata							
Macma4_06_g07190.1	***MaUGT75C1.4***	Macma4_06_g07200.1	***MaUGT75C1.3***	Macma4_06_g07210.1	***MaUGT75C1.2***		
Macma4_06_g10010.1	***MaCHS5***	Macma4_06_g10020.1	***MaCHS4***				
Macma4_07_g10660.1	***MaANR2***	Macma4_07_g10670.1	***MaANR3***	Macma4_07_g10680.1	***MaANR4***		
Macma4_02_g03240.1	***MaF3'5'H8***	Macma4_02_g03250.1	***MaF3'5'H9***	Macma4_02_g03260.1	***MaF3'5'H10***		
Macma4_02_g03290.1	***MaF3'5'H11***	Macma4_02_g03300.1	***MaF3'5'H12***				
M. balbisiana							
Mba06_g06480.1	***MbUGT75C1.2***	Mba06_g06490.1	***MbUGT75C1.3***				

Mba06_g09080.1	***MbCHS5***	Mba06_g09090.1	***MbCHS6***				
Mba06_g09110.1	***MbCHS4***	Mba06_g09120.1	***MbCHS7***				
Mba02_g01890.1	***MbF3'5'H7***	Mba02_g01900.1	***MbF3'5'H8***	Mba02_g01910.1	***MbF3'5'H9***	Mba02_g01920.1	***MbF3'5'H10***
M. schizocarpa							
Ms02t024400	***MsF3'5'H5***	Ms02t024410	***MsF3'5'H6***				
Ms06t152710	***MsUGT75C1***.1	Ms06t152720	***MsUGT75C1.2***	Ms06t152730	***MsUGT75C1.3***		
Ms07t188910	***MsANR3***	Ms07t188920	***MsANR2***				
MsSC51t327370	***MsCHS3***	MsSC51t327380	***MsCHS4***				

Table S5b. Segmental duplication FBG pairs in Musaceae.

Gene1	Name1	Gene2	Name2	
Mbe02_t027270	***Mbe4CL11***	Mbe06_t040140	***Mbe4CL12***	
Mbe03_t056570	***Mbe4CL13***	Mbe04_t006230	***Mbe4CL17***	
Mbe01_t005810	***Mbe4CL14***	Mbe03_t000920	***Mbe4CL15***	
Mbe01_t005810	***Mbe4CL14***	Mbe07_t022330	***Mbe4CL16***	*
Mbe03_t000920	***Mbe4CL15***	Mbe07_t022330	***Mbe4CL16***	
Mbe05_t011100	***Mbe4CL9***	Mbe07_t013640	***Mbe4CL10***	
Mbe01_t038520	***MbeC4H1***	Mbe02_t033650	***MbeC4H3***	
Mbe01_t038520	***MbeC4H1***	Mbe10_t000860	***MbeC4H2***	
Mbe02_t033650	***MbeC4H3***	Mbe08_t025090	***MbeC4H4***	*
Mbe01_t038010	***MbeCHS1***	Mbe01_t047580	***MbeCHS4***	
Mbe01_t038010	***MbeCHS1***	Mbe11_t002790	***MbeCHS5***	
Mbe01_t047580	***MbeCHS4***	Mbe11_t002790	***MbeCHS5***	
Mbe04_t011700	***MbeDFR3***	Mbe06_t019010	***MbeDFR2***	
Mbe02_t005590	***MbeF3'5'H3***	Mbe06_t049500	***MbeF3'5'H2***	

Mbe08_t039500	***MbeF3'5'H7***	Mbe09_t008950	***MbeF3'5'H8***	
Mbe02_t047300	***MbeFLS2***	Mbe03_t008100	***MbeFLS4***	*
Mbe02_t047300	***MbeFLS2***	Mbe08_t036240	***MbeFLS3***	
Mbe05_t009880	***MbePAL1***	Mbe06_t040740	***MbePAL2***	
Mbe05_t009880	***MbePAL1***	Mbe07_t015550	***MbePAL3***	*
Mbe06_t040740	***MbePAL2***	Mbe07_t015550	***MbePAL3***	
Mbe06_t048840	***MbePAL5***	Mbe07_t015550	***MbePAL3***	
Mbe06_t001720	***MbeTT8.2***	Mbe06_t034420	***MbeTT8.1***	
Mbe02_t045890	***MbeUGT78D2.2***	Mbe03_t010580	***MbeUGT78D2.3***	
Mbe02_t045890	***MbeUGT78D2.2***	Mbe07_t034770	***MbeUGT78D2.1***	*
Mbe03_t010580	***MbeUGT78D2.3***	Mbe07_t034770	***MbeUGT78D2.1***	
ML1h04G02613	***Ml4CL11***	ML1h07G03382	***Ml4CL12***	
ML1h02G02377	***Ml4CL13***	ML1h09G01309	***Ml4CL14***	
ML1h06G02761	***Ml4CL17***	ML1h08G00557	***Ml4CL15***	
ML1h02G00664	***Ml4CL5***	ML1h08G03149	***Ml4CL6***	*
ML1h01G00405	***Ml4CL8***	ML1h08G03149	***Ml4CL6***	
ML1h01G00972	***Ml4CL9***	ML1h07G00248	***Ml4CL10***	
ML1h05G01754	***MlC4H1***	ML1h05G04363	***MlC4H3***	*
ML1h05G01754	***MlC4H1***	ML1h08G03392	***MlC4H4***	
ML1h05G01818	***MlCHS1***	ML1h05G03634	***MlCHS2***	
ML1h05G01818	***MlCHS1***	ML1h05G04406	***MlCHS4***	*
ML1h05G03634	***MlCHS2***	ML1h05G04406	***MlCHS4***	
ML1h03G02164	***MlDFR3***	ML1h06G03238	***MlDFR2***	
ML1h06G02237	***MlF3'5'H6***	ML1h08G03854	***MlF3'5'H4***	
ML1h07G02004	***MlF3H1***	ML1h08G02519	***MlF3H2***	

ML1h05G00635	***MlFLS3***	ML1h06G02043	***MlFLS4***	*
ML1h05G00635	***MlFLS3***	ML1h09G00678	***MlFLS5***	
ML1h04G02672	***MlPAL1***	ML1h07G00086	***MlPAL3***	
ML1h01G00856	***MlPAL2***	ML1h04G02672	***MlPAL1***	
ML1h01G00856	***MlPAL2***	ML1h07G00086	***MlPAL3***	*
ML1h07G00086	***MlPAL3***	ML1h07G01701	***MlPAL5***	
ML1h04G03282	***MlPAL4***	ML1h07G00086	***MlPAL3***	
ML1h04G03282	***MlPAL4***	ML1h07G01701	***MlPAL5***	
ML1h06G01843	***MlUGT78D2.2***	ML1h09G00476	***MlUGT78D2.1***	
ML1h05G00763	***MlUGT78D2.3***	ML1h06G01843	***MlUGT78D2.2***	*
ML1h05G00763	***MlUGT78D2.3***	ML1h09G00476	***MlUGT78D2.1***	
Eg02_t018790	***EgPAL4***	Eg09_t011330	***EgPAL5***	
Eg02_t001010	***EgPAL6***	Eg09_t011330	***EgPAL5***	
Eg02_t001010	***EgPAL6***	Eg09_t017980	***EgPAL7***	*
Eg02_t001010	***EgPAL6***	Eg05_t024420	***EgPAL8***	
Eg05_t024420	***EgPAL8***	Eg09_t017980	***EgPAL7***	
Eg08_t019660	***EgC4H1***	Eg08_t048930	***EgC4H3***	
Eg07_t034110	***EgC4H4***	Eg08_t019660	***EgC4H1***	*
Eg07_t003130	***EgC4H2***	Eg08_t048930	***EgC4H3***	
Eg02_t002700	***Eg4CL1.1***	Eg05_t023200	***Eg4CL1.2***	
Eg02_t037340	***Eg4CL2.1***	Eg09_t018540	***Eg4CL2.2***	
Eg01_t014520	***Eg4CL10***	Eg01_t025930	***Eg4CL11***	
Eg04_t032900	***Eg4CL13***	Eg07_t014740	***Eg4CL15***	
Eg08_t020390	***EgCHS3***	Eg08_t040870	***EgCHS2***	*
Eg08_t040870	***EgCHS2***	Eg08_t049390	***EgCHS4***	

Eg08_t020390	***EgCHS3***	Eg08_t049390	***EgCHS4***	
Eg01_t007540	***EgFLS2***	Eg08_t007020	***EgFLS3***	
Eg06_t008220	***EgUGT78D2.2***	Eg08_t008340	***EgUGT78D2.1***	
Eg01_t005290	***EgUGT78D2.3***	Eg08_t008340	***EgUGT78D2.1***	*
Eg01_t005290	***EgUGT78D2.3***	Eg06_t008220	***EgUGT78D2.2***	
Eg03_t004200	***EgTT8.2***	Eg04_t020700	***EgTT8.1***	
Macma4_08_g14800.1	***MaPAL7***	Macma4_11_g15930.1	***MaPAL3***	
Macma4_02_g02760.1	***MaPAL4***	Macma4_08_g14800.1	***MaPAL7***	
Macma4_05_g21700.1	***MaPAL5***	Macma4_11_g22170.1	***MaPAL6***	*
Macma4_05_g21700.1	***MaPAL5***	Macma4_08_g14800.1	***MaPAL7***	
Macma4_08_g14800.1	***MaPAL7***	Macma4_11_g22170.1	***MaPAL6***	
Macma4_06_g10450.1	***MaC4H3***	Macma4_10_g16840.1	***MaC4H1***	
Macma4_09_g04800.1	***MaC4H4***	Macma4_10_g16840.1	***MaC4H1***	*
Macma4_06_g10450.1	***MaC4H3***	Macma4_07_g20380.1	***MaC4H2***	
Macma4_05_g22890.1	***Ma4CL1.2***	Macma4_08_g13040.1	***Ma4CL1.1***	
Macma4_02_g21360.1	***Ma4CL2.1***	Macma4_11_g22750.1	***Ma4CL2.2***	
Macma4_01_g19800	***Ma4CL10***	Macma4_03_g13930	***Ma4CL11***	
Macma4_01_g19800	***Ma4CL10***	Macma4_07_g05220	***Ma4CL14***	*
Macma4_03_g13930	***Ma4CL11***	Macma4_07_g05220	***Ma4CL14***	
Macma4_04_g06110	***Ma4CL12***	Macma4_09_g24760	***Ma4CL16***	
Macma4_06_g10010.1	***MaCHS5***	Macma4_06_g18170.1	***MaCHS2***	
Macma4_06_g18170.1	***MaCHS2***	Macma4_10_g16140.1	***MaCHS6***	*
Macma4_06_g10010.1	***MaCHS5***	Macma4_10_g16140.1	***MaCHS6***	
Macma4_07_g16200.1	***MaUGT78D2.2***	Macma4_10_g27580.1	***MaUGT78D2.1***	*
Macma4_03_g04880.1	***MaUGT78D2.3***	Macma4_10_g27580.1	***MaUGT78D2.1***	

Macma4_03_g04880.1	***MaUGT78D2.3***	Macma4_07_g16200.1	***MaUGT78D2.2***	
Macma4_03_g19880.1	***MaTT8.2***	Macma4_06_g25800.1	***MaTT8.1***	
Macma4_09_g17660.1	***MaF3'5'H4***	Macma4_10_g01240.1	***MaF3'5'H5***	*
Macma4_02_g03240.1	***MaF3'5'H8***	Macma4_10_g01240.1	***MaF3'5'H5***	
Mba08_g14430	***MbPAL3***	Mba11_g20430	***MbPAL2***	*
Mba02_g01510	***MbPAL5***	Mba08_g14430	***MbPAL3***	
Mba09_g04380	***MbC4H1***	Mba10_g11450	***MbC4H2***	*
Mba06_g09560	***MbC4H3***	Mba10_g11450	***MbC4H2***	
Mba02_g18560	***Mb4CL2.1***	Mba11_g20940	***Mb4CL2.2***	
Mba01_g28370	***Mb4CL10***	Mba07_g04890	***Mb4CL13***	
Mba04_g05720	***Mb4CL11***	Mba04_g12340	***Mb4CL12***	
Mba06_g09080	***MbCHS5***	Mba06_g16830	***MbCHS2***	
Mba06_g16830	***MbCHS2***	Mba10_g10820	***MbCHS8***	*
Mba06_g09080	***MbCHS5***	Mba10_g10820	***MbCHS8***	
Mba03_g06940	***MbFLS4***	Mba10_g21980	***MbFLS3***	
Mba03_g04830	***MbUGT78D2.1***	Mba10_g20800	***MbUGT78D2.2***	
Mba03_g04830	***MbUGT78D2.1***	Mba07_g14580	***MbUGT78D2.3***	*
Mba07_g14580	***MbUGT78D2.3***	Mba10_g20800	***MbUGT78D2.2***	
Mba03_g18730	***MbTT8.2***	Mba06_g23950	***MbTT8.1***	
Mba09_g16030	***MbF3'5'H3***	Mba10_g00740	***MbF3'5'H4***	
Mba02_g01840	***MbF3'5'H5***	Mba11_g13650	***MbF3'5'H13***	
Ms02t024050	***MsPAL4***	Ms11t313180	***MsPAL3***	
Ms08t220210	***MsPAL5***	Ms11t313180	***MsPAL3***	*
Ms05t135820	***MsPAL7***	Ms11t313180	***MsPAL3***	
Ms02t024050	***MsPAL4***	Ms08t220210	***MsPAL5***	

Ms08t220210	***MsPAL5***	Ms11t319150	***MsPAL6***	
Ms05t135820	***MsPAL7***	Ms08t220210	***MsPAL5***	
Ms05t135820	***MsPAL7***	Ms11t319150	***MsPAL6***	
Ms09t244050	***MsC4H3***	Ms10t281630	***MsC4H1***	
Ms05t136800	***Ms4CL1.2***	Ms08t218760	***Ms4CL1.1***	
Ms02t041000	***Ms4CL2.1***	Ms11t319670	***Ms4CL2.2***	
Ms01t017810	***Ms4CL10***	Ms03t058920	***Ms4CL11***	
Ms01t017810	***Ms4CL10***	Ms07t183930	***Ms4CL14***	*
Ms03t058920	***Ms4CL11***	Ms07t183930	***Ms4CL14***	
Ms04t083350	***Ms4CL12***	Ms04t089570	***Ms4CL13***	*
Ms04t083350	***Ms4CL12***	Ms09t262230	***Ms4CL16***	
Ms06t158650	***MsCHS2***	Ms10t280970	***MsCHS1***	
Ms10t280970	***MsCHS1***	MsSC51t327370	***MsCHS3***	*
Ms06t158650	***MsCHS2***	MsSC51t327370	***MsCHS3***	
Ms03t076720	***MsDFR3***	Ms04t087800	***MsDFR2***	
Ms07t193970	***MsUGT78D2.2***	Ms10t291590	***MsUGT78D2.1***	
Ms03t050760	***MsUGT78D2.3***	Ms10t291590	***MsUGT78D2.1***	*
Ms03t050760	***MsUGT78D2.3***	Ms07t193970	***MsUGT78D2.2***	

Table S6 Expression of some flavonoid biosynthetic genes in *Musella lasiocarpa*

Gene ID	Ynormal	Ycold	Rnormal	Rcold
TPM value < 1				
Ml4CL12	0.01	0	0	0
Ml4CL18	0	0	0.69	0.39
Ml4CL2	0.02	0.03	0.01	0.01
Ml4CL4	0	0.07	0.04	0.37
Ml4CL5	0.01	0.02	0.05	0.01
Ml4CL6	0	0	0	0
Ml4CL9	0	0	0.03	0.01
MlCHS4	0.02	0.1	0.05	0
MlDFR1	0.01	0	0	0
MlF3'5'H10	0.01	0	0.07	0.32
MlF3'5'H11	0.02	0	0	0
MlF3'5'H12	0.34	0.25	0.82	0.3
MlF3'5'H3	0.31	0.63	0.11	0.03
MlF3'5'H4	0.18	0	0.49	0.04
MlF3'5'H6	0.04	0.03	0.1	0.05
MlF3'5'H9	0.04	0.01	0.22	0.5
MlPAL3	0	0.04	0	0
MlUGT78D2.2	0.79	0.37	0.29	0
TPM value > 100				
MlC4H1	193.72	112.9	101.28	118.84
MlCHI1	140.98	273.35	311.89	539.02
MlCHI2	361.34	830.13	604.17	313.68
MlCHS1	388.34	175.86	279.61	423.57
MlCHS2	386.32	180.7	307.1	212.48
MlDFR2	152.89	259.08	432.97	637.54
MlDFR3	143.32	435.19	278.77	864.45
MlF3H1	320.16	395.44	332.26	179.85
MlF3'H	112.96	136.87	179.36	192.79
MlLDOX	266.93	135.57	291.3	373.07
Expression up-regulated in yellow bracts down-regulated in red bracts				
Ml4CL3	0.81	1.38	0.96	0.9
Ml4CL5	0.01	0.02	0.05	0.01
Ml4CL7	15.56	15.89	26.11	17.71
Ml4CL8	24.07	43.86	54.52	35.97
MlANR1	22.44	26.88	37.29	0.64
MlANR2	18.16	25.09	3.56	0.07
MlANR3	9.63	14.51	9.71	0.18
MlC1.2	2.03	2.1	4.62	0.23
MlCHI2	361.34	830.13	604.17	313.68
MlCHS3	0.05	0.55	2.86	0.21
MlF3'5'H1	8.98	17.61	20.12	9.54
MlF3'5'H3	0.31	0.63	0.11	0.03
MlF3H1	320.16	395.44	332.26	179.85
MlFLS5	56.93	101.99	38.37	33.75
MlPAL4	1.64	5.03	23.52	21.03

MlTTG1.1	4.99	5.45	6.07	2.99
Expression down-regulated in yellow bracts up-regulated in red bracts				
Ml4CL13	201.08	91.43	54.24	74.48
Ml4CL14	55.06	28.74	30.21	72.25
Ml4CL16	10.88	4.86	13.61	14.44
MlC4H1	193.72	112.9	101.28	118.84
MlC4H3	178.58	72.5	135.23	192.92
MlCHS1	388.34	175.86	279.61	423.57
MlF3'5'H2	3.15	0.83	1.26	4.25
MlF3'5'H7	0.1	0.03	0.41	1.67
MlF3'5'H8	0.29	0.09	0.7	3.35
MlF3'5'H9	0.04	0.01	0.22	0.5
MlF3H2	23.41	22.36	31.47	33
MlFLS2	61.55	19.45	63.6	66.77
MlFLS3	71.84	71.34	46.98	52.68
MlFLS4	4.76	3.88	3.46	6.61
MlLDOX	266.93	135.57	291.3	373.07
MlPAL1	132.91	101.54	35.1	80.72
MlPAL5	21.79	15.4	15.3	22.55
MlUGT75C1.2	6.23	1.31	15.3	70.95
MlUGT75C1.3	13.83	6.38	25.55	84.71

Table S7. Expression of flavonoid biosynthetic gene in *Musella lasiocarpa*

Gene Name	Gene ID	Ynormal	Ycold	Rnormal	Rcold
Ml4CL1	*ML1h07G00167*	29.45	17.6	23.92	19.86
Ml4CL10	*ML1h07G00248*	7.1	9.44	25.94	70.84
Ml4CL11	*ML1h04G02613*	15.48	16.05	40.02	50.1
Ml4CL12	*ML1h07G03382*	0.01	0	0	0
Ml4CL13	*ML1h02G02377*	201.08	91.43	54.24	74.48
Ml4CL14	*ML1h09G01309*	55.06	28.74	30.21	72.25
Ml4CL15	*ML1h08G00557*	7.4	6.81	6.91	5.2
Ml4CL16	*ML1h06G03426*	10.88	4.86	13.61	14.44
Ml4CL17	*ML1h06G02761*	4.65	1.01	7.35	1.88
Ml4CL18	*ML1h09G02144*	0	0	0.69	0.39
Ml4CL2	*ML1h08G00140*	0.02	0.03	0.01	0.01
Ml4CL3	*ML1h04G04085*	0.81	1.38	0.96	0.9
Ml4CL4	*ML1h03G00142*	0	0.07	0.04	0.37
Ml4CL5	*ML1h02G00664*	0.01	0.02	0.05	0.01
Ml4CL6	*ML1h08G03149*	0	0	0	0
Ml4CL7	*ML1h01G00403*	15.56	15.89	26.11	17.71
Ml4CL8	*ML1h01G00405*	24.07	43.86	54.52	35.97
Ml4CL9	*ML1h01G00972*	0	0	0.03	0.01
MlANR1	*ML1h06G01340*	22.44	26.88	37.29	0.64
MlANR2	*ML1h06G01342*	18.16	25.09	3.56	0.07
MlANR3	*ML1h06G01341*	9.63	14.51	9.71	0.18
MlANR4	*ML1h07G01339*	15.45	28.97	7.61	0.01

MlC1.1	*ML1h08G01785*	0.95	1.06	2.6	0
MlC1.2	*ML1h05G03234*	2.03	2.1	4.62	0.23
MlC1.3	*ML1h05G01322*	0.67	0.38	1.44	1
MlC4H1	*ML1h05G01754*	193.72	112.9	101.28	118.84
MlC4H2	*ML1h08G00252*	38.38	19.29	57.22	15.25
MlC4H3	*ML1h05G04363*	178.58	72.5	135.23	192.92
MlC4H4	*ML1h08G03392*	54.17	49.53	28.4	26.69
MlCHI1	*ML1h09G01419*	140.98	273.35	311.89	539.02
MlCHI2	*ML1h04G02595*	361.34	830.13	604.17	313.68
MlCHS1	*ML1h05G01818*	388.34	175.86	279.61	423.57
MlCHS2	*ML1h05G03634*	386.32	180.7	307.1	212.48
MlCHS3	*ML1h05G04408*	0.05	0.55	2.86	0.21
MlCHS4	*ML1h05G04406*	0.02	0.1	0.05	0
MlDFR1	*ML1h04G00849*	0.01	0	0	0
MlDFR2	*ML1h06G03238*	152.89	259.08	432.97	637.54
MlDFR3	*ML1h03G02164*	143.32	435.19	278.77	864.45
MlF3'5'H1	*ML1h07G00690*	8.98	17.61	20.12	9.54
MlF3'5'H10	*ML1h03G01872*	0.01	0	0.07	0.32
MlF3'5'H11	*ML1h07G01741*	0.02	0	0	0
MlF3'5'H12	*ML1h04G03337*	0.34	0.25	0.82	0.3
MlF3'5'H2	*ML1h04G02425*	3.15	0.83	1.26	4.25
MlF3'5'H3	*ML1h07G00951*	0.31	0.63	0.11	0.03
MlF3'5'H4	*ML1h08G03854*	0.18	0	0.49	0.04
MlF3'5'H5	*ML1h06G02235*	4.86	2.11	9.73	6.15
MlF3'5'H6	*ML1h06G02237*	0.04	0.03	0.1	0.05

MlF3'5'H7	*ML1h07G01736*	0.1	0.03	0.41	1.67
MlF3'5'H8	*ML1h07G01737*	0.29	0.09	0.7	3.35
MlF3'5'H9	*ML1h02G01787*	0.04	0.01	0.22	0.5
MlF3H1	*ML1h07G02004*	320.16	395.44	332.26	179.85
MlF3H2	*ML1h08G02519*	23.41	22.36	31.47	33
MlF3'H1	*ML1h03G02252*	112.96	136.87	179.36	192.79
MlFLS1	*ML1h03G01030*	0	0	3.05	0.01
MlFLS2	*ML1h05G00636*	61.55	19.45	63.6	66.77
MlFLS3	*ML1h05G00635*	71.84	71.34	46.98	52.68
MlFLS4	*ML1h06G02043*	4.76	3.88	3.46	6.61
MlFLS5	*ML1h09G00678*	56.93	101.99	38.37	33.75
MlLAR	*ML1h01G01259*	133.44	69.33	107.16	50.07
MlLDOX	*ML1h01G01735*	266.93	135.57	291.3	373.07
MlPAL1	*ML1h04G02672*	132.91	101.54	35.1	80.72
MlPAL2	*ML1h01G00856*	37.45	53.39	71.38	187.42
MlPAL3	*ML1h07G00086*	0	0.04	0	0
MlPAL4	*ML1h04G03282*	1.64	5.03	23.52	21.03
MlPAL5	*ML1h07G01701*	21.79	15.4	15.3	22.55
MlPAL6	*ML1h02G00424*	18.04	28.33	18.5	40.44
MlPAL7	*ML1h01G01720*	10.86	6.53	12.86	0.6
MlPAL8	*ML1h01G01719*	19.68	10.08	14.6	0.62
MlPAL9	*ML1h06G02093*	31.66	15.86	56.84	36.63
MlTT8.1	*ML1h03G03510*	0.2	0.81	0.81	1.04
MlTT8.2	*ML1h02G03012*	12.19	29.59	66.44	77.94
MlTTG1.1	*ML1h01G02333*	4.99	5.45	6.07	2.99

MlTTG1.2	*ML1h04G00438*	11.75	8.39	16.26	12.93
MlUGT75C1.1	*ML1h05G00327*	1.97	2.17	7.32	14.64
MlUGT75C1.2	*ML1h05G03318*	6.23	1.31	15.3	70.95
MlUGT75C1.3	*ML1h05G03317*	13.83	6.38	25.55	84.71
MlUGT78D2.1	*ML1h09G00476*	26.23	36.56	17.09	36.09
MlUGT78D2.2	*ML1h06G01843*	0.79	0.37	0.29	0
MlUGT78D2.3	*ML1h05G00763*	9.66	46.16	33.86	36.52
MlUGT79B1	*ML1h08G03185*	30.36	30.41	38.5	40.98

The differentially expressed genes (DEGs) were highlighted by red (Red bracts) and yellow (yellow bracts).
